# Nanoindentation dataset of silicon and hafnia doped silicon coatings produced by magnetron sputtering

**DOI:** 10.1016/j.dib.2020.105800

**Published:** 2020-06-03

**Authors:** Ronja Anton, Vito Leisner, Uwe Schulz

**Affiliations:** German Aerospace Center (DLR), Institute of Materials Research, Cologne 51147, Germany

**Keywords:** Physical vapour deposition, Silicon, Hafnia, Nanoindentation, Hardness, Environmental barrier coating

## Abstract

Mechanical properties of three different coatings comprising of pure silicon, 36 mol% HfO_2_-doped silicon and 60 mol% HfO_2_-doped silicon on SiC substrate material were obtained by nanoindentation. The coatings aim at oxidation protective layers in environmental barrier coating systems for SiC/SiC ceramic matrix composites (CMC). The examined coatings were produced by physical vapour deposition (magnetron sputtering) and have been tested under cycling conditions between room temperature and 1523 K until 100 h accumulated hot time. In order to measure the hardness and the reduced Young's modulus, a Berkovich tip has been used with a constant depth modulus of 100 nm and 160 nm. Two depth moduli have been chosen to investigate a possible volume impact on the mechanical properties. Six successfully produced indents have been averaged to examine the hardness and reduced Young's modulus. The testing has been done on polished cross sections thereby minimising the impact of the substrate material on the measured values. This article provides data related to “Hafnia-doped Silicon Bond Coats manufactured by PVD for SiC/SiC CMCs”.

Specifications table

**Table:** 

Subject	Materials Science/Surfaces, Coatings and Films
Specific subject area	Mechanical testing of silicon containing bond coats for applications in environmental barrier coatings for protection of SiC/SiC CMCs
Type of data	Image Graph Figure Table
How data were acquired	Data were obtained by means of characterisation techniques such as nanoindentation and atomic force microscopy (Hysitron Tribo-Indenter TI 900)
Data format	Raw Analysed Filtered
Parameters for data collection	With an indent depth of 100 nm, silicon and 36 mol% HfO_2_-doped silicon have been tested in the state of as-coated, after 10 h and 100 h of furnace cycle test at 1523 K. With an indent depth of 160 nm, all compositions have been tested after 100 h of furnace cycle test at 1523 K
Description of data collection	The testing has been done on cross sections of the coatings, thereby minimising the impact of the SiC substrate material. AFM helped to obtain the right position of the indent.
Data source location	Saarbrücken/Saarland, Cologne/Nordrhein-Westfalen Germany
Data accessibility	With the article
Related research article	Ronja Anton, Vito Leisner, Philipp Watermeyer, Michael Engstler, Uwe Schulz Hafnia-doped Silicon Bond Coats manufactured by PVD for SiC/SiC CMCs Acta Materialia doi:10.1016/j.actamat.2019.10.050

## Value of the data

• A characterisation of the coatings towards their mechanical stability is important to further understand the strengthening and crack-suppression mechanisms attributed to the hafnia/hafnon within the silicon matrix.
• The data are beneficial for further improvements of the coatings with the focus on crack suppression and mechanical strengthening.
• By understanding the mechanical properties further improvements of the dopant amount can be predicted.
• Data may be useful for future research

## 1 Data

This article provides two main data sets from nanoindentation measurements. One has been obtained with a constant depth mode of 100 nm and compares a pure silicon coating as benchmark with a 36 mol% HfO_2_-doped silicon coating; see Figs. 2 and 4 over testing time up to 100 h at 1523 K. The other set, tested with a constant depth mode of 160 nm, compares the silicon coating with the 36 mol% HfO_2_-doped and the 60 mol% HfO_2_-doped silicon coating after 100 h at 1523 K, see Figs. 5 and 6. The diagrams represent the mean hardness as well as the mean reduced Young's modulus of the tested conditions. The error bars represent the scattering of the data. The coatings have been applied using magnetron sputtering. The coatings are dense after oxidation treatment. The HfO_2_ particles are roundly shaped and homogenously distributed within the silicon coating. The size of the particles is about 69 nm for the 36 mol% HfO_2_-doped silicon coating and about 93 nm for the 60 mol% HfO_2_-doped silicon coating [1].

## 2 Experimental design, materials, and methods

Three different compositions of coatings have been tested using nanoindentation. The compositions consist of pure silicon, 36 mol% HfO_2_-doped silicon and 60 mol% HfO_2_-doped silicon. They have been investigated in the conditions as-coated, after 10 h of furnace cycle test and after 100 h furnace cycle test. Physical vapour deposition has been used to manufacture the specimen shown here. A detailed description about the process, the furnace cycle test and the microstructural development of the coatings can be found in Anton et al. [1]. The measurement was carried out at the Tribo-Indenter TI 900 of the company Hysitron with a Berkovich tip. The tip radius is about 100 nm in its original condition [2]. However, since indentation causes ablation, the tip had a diameter of about 400 nm when these measurements were carried out. In order to obtain the same measurement hysteresis, it was necessary to identify surfaces with a low roughness. The built-in atomic force microscope (AFM) made it possible to determine the exact positions of the indents in the coating and to examine them after indentation. An image section from 25 µm to 25 µm has been taken, see Fig. 3. The indent measurements were performed with a constant penetration depth of 100 nm and 160 nm.

**Fig. 1:**
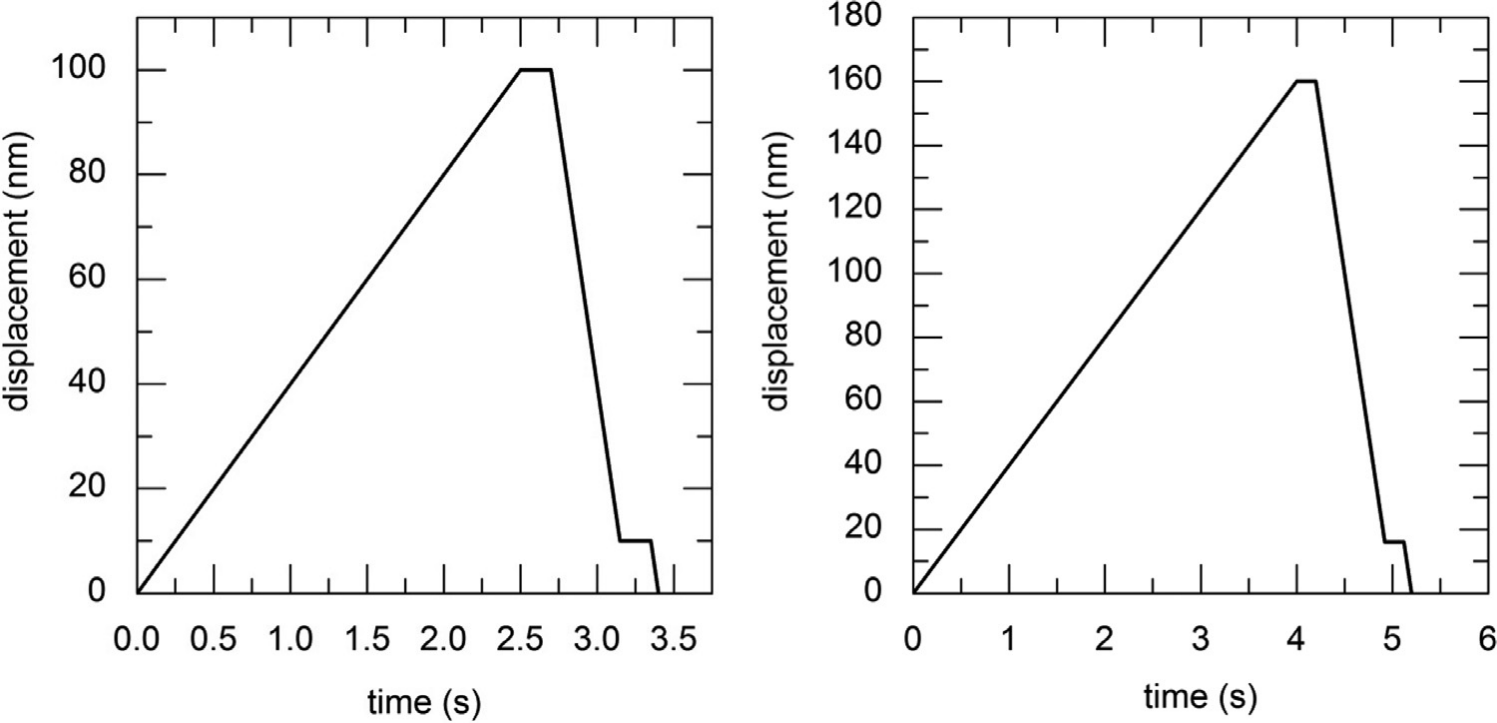
Measure path of the two used test constant depth modi; left: 100 nm; right: 160 nm.

Since the samples were inhomogeneous on a nanoscale, the influence of the volume was checked with the different indent-depths. The curves shown in Fig. 1 represent the indented path travelled and the measurement method at the same time. The visible plateaus were necessary to avoid drifting of the sample. The software automatically calculated the force penetration depth hysteresis by the recorded force, hardness and the reduced Young's modulus.

**Fig. 2:**
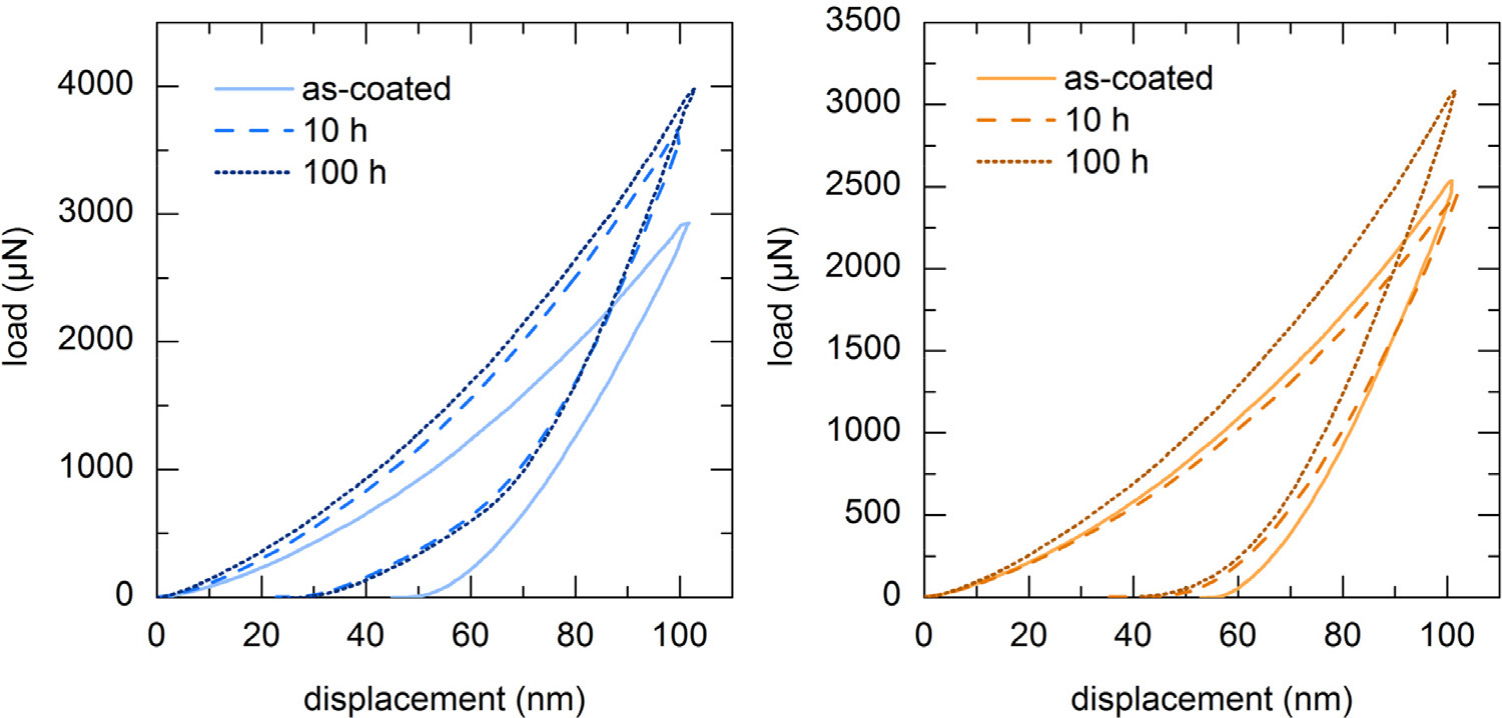
Berkovich hysteresis with a constant depth of 100 nm; left: silicon; right: 36 mol% HfO_2_ doped silicon.

Fig. 2 summarises the analysed hystereses of the silicon coating and the 36 mol% HfO2-doped silicon coating as mean value curves. The data points can be found in Table 1 at the end of the article. They are divided into the three different conditions before and after FCT. The hystereses show that the 100 h sample could resist the greatest load followed by the 10 h sample and the as-coated specimen. The maximum load at the final penetration depth of 100 nm is proportional to the hardness. The shape of the hysteresis indicates the elastic and plastic deformation of the material.

**Table 1:** Poisson number of the tested materials [3,4].

Material	Poisson number (*v_i_*)
Silicon	0.22
HfO_2_	0.3

**Fig. 3:**
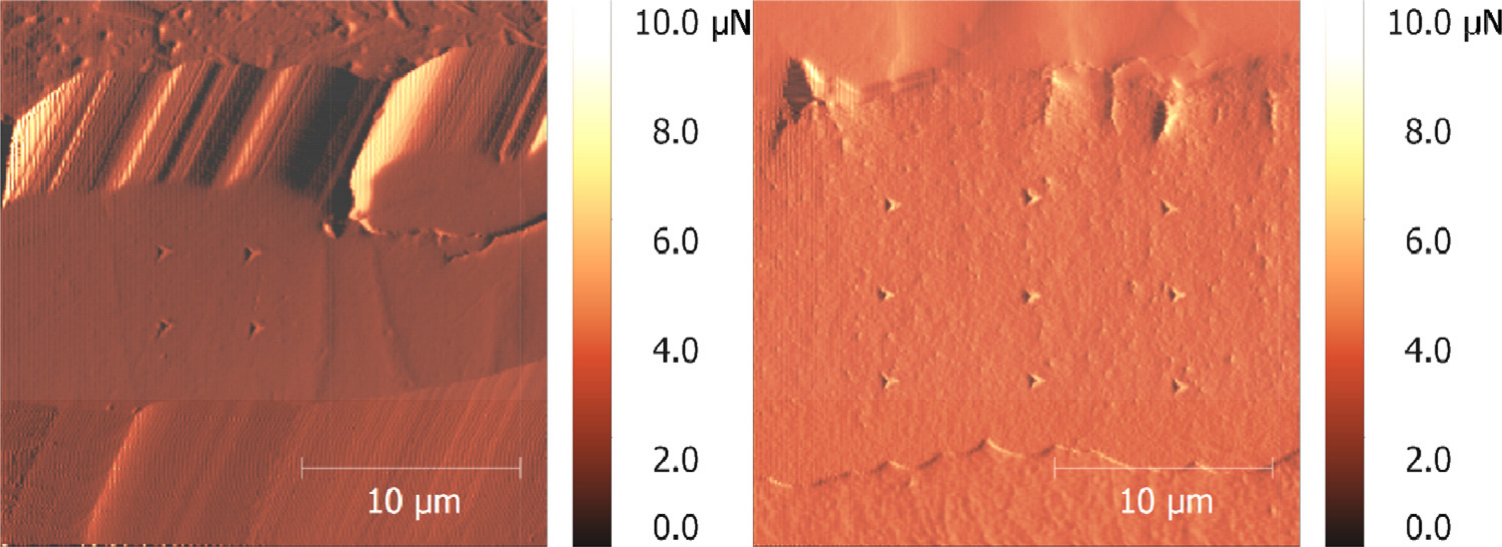
AFM pictures after indentation; left: silicon; right: 36 mol% HfO_2_ doped silicon.

The irreversible plastic deformations in the material are visualised by images of the AFM. The AFM also serves as an orientation aid on the sample. Fig. 3 shows an example of an image taken after indentation.

**Fig. 4:**
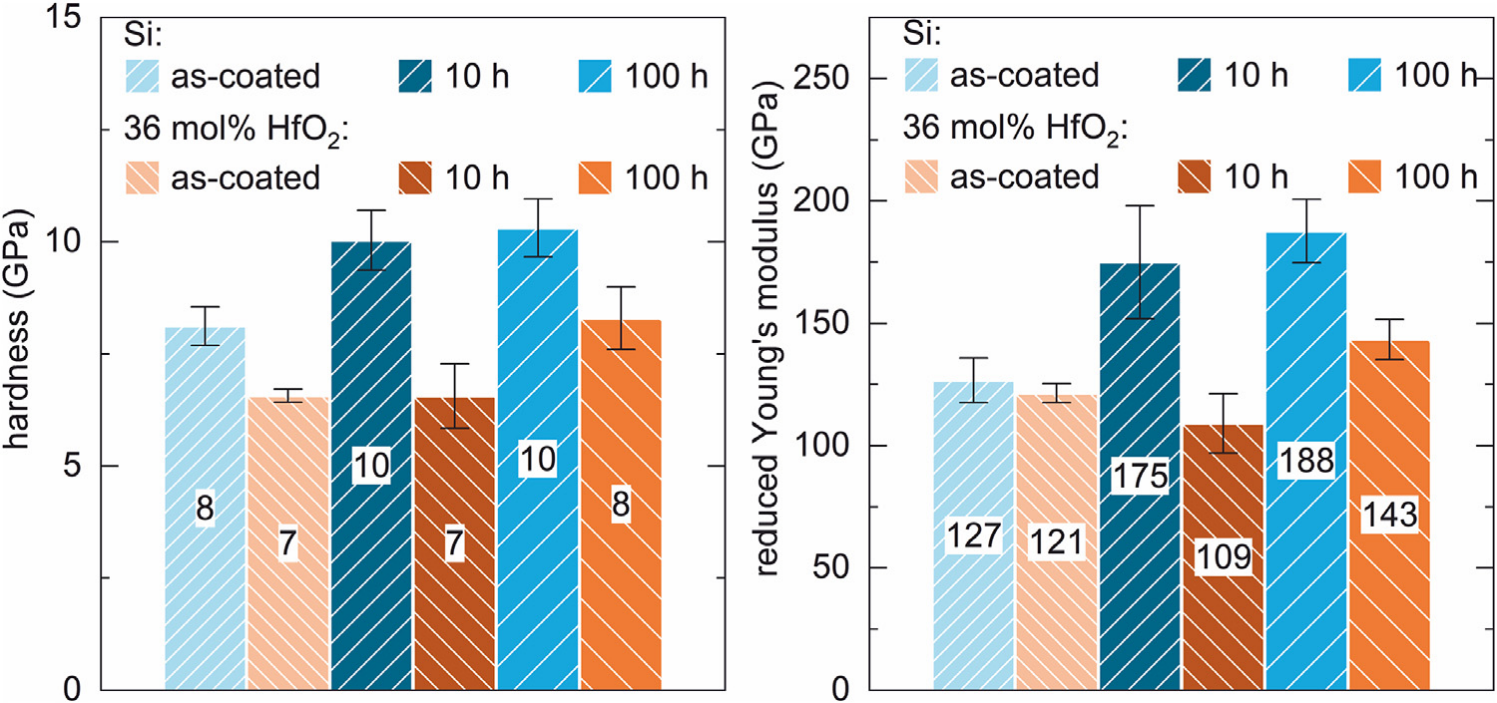
Nanoindentation of samples in the conditions as-coated and after 10 h furnace cycle test with a Berkovich tip and a constant depth of 100 nm; left: hardness; right: reduced Young's modulus; the numbers in the bars represent the average measured values in GPa).

The silicon coating showed a higher hardness and higher reduced Young's modulus for all tested conditions compared to the lower hafnia-doped coating, see Fig. 4. There, the specific test data are displayed in the bars in the unit GPa. Furthermore, hardness and reduced Young's modulus increased with crystallisation of the Si coating. In contrast the low content hafnia coating stayed about the same in hardness for as-coated and after 10 h FCT, then it increased after 100 h furnace cycle test. The reduced Young's modulus decreased slightly from as-coated to after 10 h furnace cycle test, followed by an increase after 100 h furnace cycle test.

**Fig. 5:**
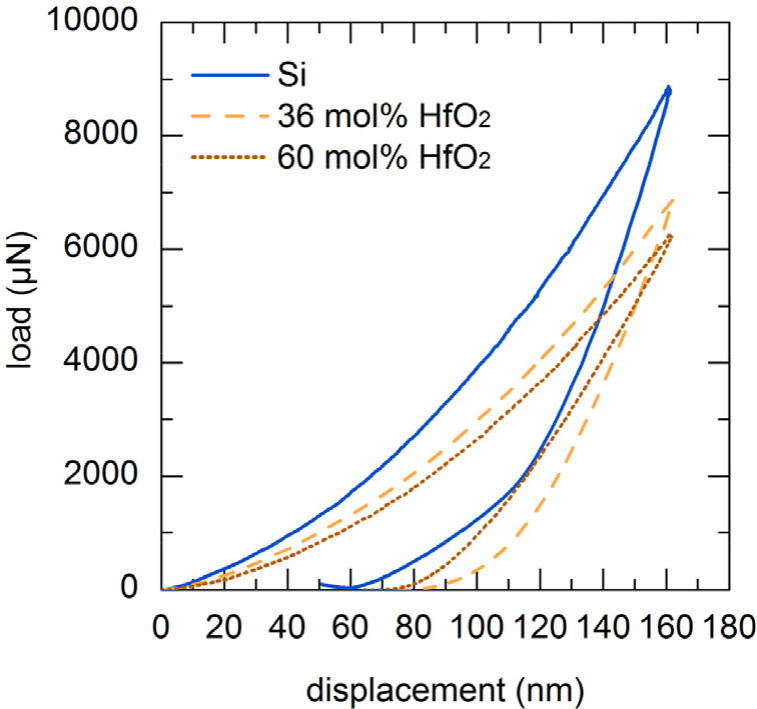
Comparison of the hysterses of silicon, 36 mol% HfO_2_-doped silicon and 60 mol% HfO_2_-doped silicon at 160 nm constant depth mode.

**Fig. 6:**
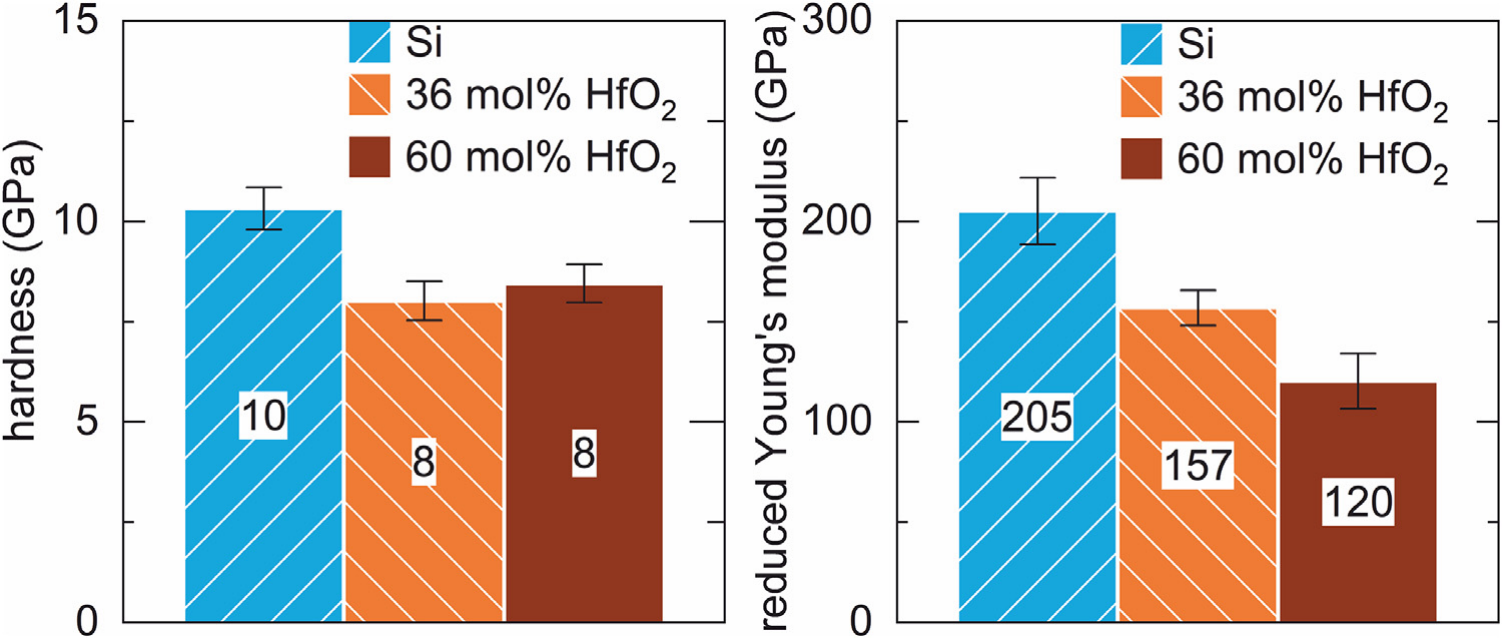
Nanoindentation of all varaiants after 100 h FCT with a Berkovich tip and a constant depth of 160 nm; left: hardness; right: reduced Young's modulus; (the numbers in the bars represent the average measured values in GPa).

The data in Figs. 5 and 6 was also obtained from samples tested for 100 h furnace cycle test at 1523 K. Here, the mechanical testing has been conducted with a constant depth mode of 160 nm. The reference sample of pure silicon exhibited the highest hardness and the highest modulus of elasticity. Both hafnia-doped coatings showed a similar hardness which was lower than that of the pure silicon coating. The lower hafnia-doped coating displayed a higher modulus of elasticity than the higher hafnia-doped coating, but both were lower than that of pure silicon.

Lastly, the reduced Young's modulus has been converted into the Young's modulus with the following formula:

$$E=\frac{1-\left(x_{HfO2}\nu_{HfO2}^{2}+x_{Si}\nu_{Si}^{2}\right)}{\frac{1}{E_{r}}-\frac{1-0,07^{2}}{1141\;\text{GPa}}}$$

*x_i_* represents the volume fraction of the phase (silicon or hafnia). *v_i_* is the Poisson number and the reduced Young's modulus is represented with *E_r_*.

The passion number of the diamond indent is 0.07 and the Young's modulus has a value of 1141 GPa. The volume fractions of the phases have been calculated with the data from the serial sectioning analysis. The methods can be found in more detail in Anton et al. [1].

The data has been summed up in Table 2.

**Table 2:** Data of the calculated Young's modulus for each coating tested until 100 h at 1523 K.

Variant	*x_HfO_*_2_	*x_Si_*	*E_r_* [GPa]	*E* [GPa] @ 160 nm
Si	0	1	205/188	195 (179 @100 nm)
60 mol% HfO_2_	0,32	0,68	120	113
36 mol% HfO_2_	0,2	0,8	157	148

## Declaration of Competing Interest

The authors declare that they have no known competing financial interests or personal relationships that could have appeared to influence the work reported in this paper.

## Appendix Supplementary materials

Image, application 1
