# Supplementary material for: Nanoindentation dataset of silicon and hafnia doped silicon coatings produced by magnetron sputtering
Source: Data Brief. 2020 Jun 3;31:105800. doi: 10.1016/j.dib.2020.105800 (PMC7306585; doi:10.1016/j.dib.2020.105800)
Supplement: Supplementary file 1 [file mmc1.docx]

Table 3. Raw data of Fig. 2.

| silicon | | | | | | 36 mol% HfO_2_ doped silicon | | | | | |
| --- | --- | --- | --- | --- | --- | --- | --- | --- | --- | --- | --- |
| as coated | | 10 h | | 100 h | | as coated | | 10 h | | 100 h | |
| depth (nm) | load (µN) | depth (nm) | load (µN) | depth (nm) | load (µN) | depth (nm) | load (µN) | depth (nm) | load (µN) | depth (nm) | load (µN) |
| -10.265 | 9.344 | -11.085 | -69.663 | -7.803 | 1.990 | -9.568 | 0.725 | -9.634 | -8.258 | -9.364 | 6.593 |
| -10.153 | 9.242 | -10.972 | -68.640 | -7.693 | 2.000 | -9.456 | 0.697 | -9.522 | -8.101 | -9.253 | 6.294 |
| -10.040 | 9.137 | -10.858 | -67.617 | -7.582 | 2.009 | -9.344 | 0.664 | -9.410 | -7.949 | -9.142 | 5.981 |
| -9.927 | 9.031 | -10.745 | -66.608 | -7.471 | 2.018 | -9.232 | 0.639 | -9.298 | -7.799 | -9.031 | 5.669 |
| -9.815 | 8.936 | -10.632 | -65.570 | -7.360 | 2.028 | -9.120 | 0.628 | -9.186 | -7.642 | -8.920 | 5.337 |
| -9.702 | 8.828 | -10.519 | -64.570 | -7.250 | 2.040 | -9.008 | 0.593 | -9.074 | -7.496 | -8.809 | 5.029 |
| -9.590 | 8.714 | -10.405 | -63.528 | -7.139 | 2.056 | -8.896 | 0.569 | -8.962 | -7.314 | -8.697 | 4.711 |
| -9.477 | 8.621 | -10.292 | -62.521 | -7.028 | 2.060 | -8.784 | 0.555 | -8.850 | -7.132 | -8.586 | 4.393 |
| -9.365 | 8.536 | -10.179 | -61.479 | -6.918 | 2.082 | -8.672 | 0.515 | -8.738 | -6.919 | -8.475 | 4.077 |
| -9.252 | 8.424 | -10.065 | -60.465 | -6.807 | 2.096 | -8.560 | 0.508 | -8.626 | -6.760 | -8.364 | 3.775 |
| -9.139 | 8.300 | -9.952 | -59.428 | -6.696 | 2.122 | -8.448 | 0.472 | -8.514 | -6.566 | -8.253 | 3.456 |
| -9.027 | 8.267 | -9.839 | -58.399 | -6.586 | 2.127 | -8.336 | 0.455 | -8.402 | -6.365 | -8.142 | 3.174 |
| -8.914 | 8.136 | -9.725 | -57.385 | -6.475 | 2.170 | -8.224 | 0.424 | -8.290 | -6.183 | -8.031 | 2.826 |
| -8.802 | 8.058 | -9.612 | -56.369 | -6.364 | 2.179 | -8.112 | 0.398 | -8.178 | -5.939 | -7.920 | 2.554 |
| -8.689 | 7.954 | -9.499 | -55.356 | -6.253 | 2.200 | -8.000 | 0.400 | -8.066 | -5.780 | -7.808 | 2.283 |
| -8.577 | 7.831 | -9.385 | -54.355 | -6.143 | 2.179 | -7.888 | 0.359 | -7.955 | -5.564 | -7.697 | 2.035 |
| -8.464 | 7.736 | -9.272 | -53.326 | -6.032 | 2.216 | -7.776 | 0.314 | -7.843 | -5.372 | -7.586 | 1.740 |
| -8.351 | 7.647 | -9.159 | -52.345 | -5.921 | 2.234 | -7.664 | 0.317 | -7.731 | -5.183 | -7.475 | 1.485 |
| -8.239 | 7.557 | -9.045 | -51.326 | -5.811 | 2.221 | -7.552 | 0.306 | -7.619 | -4.947 | -7.364 | 1.186 |
| -8.126 | 7.453 | -8.932 | -50.295 | -5.700 | 2.283 | -7.440 | 0.259 | -7.507 | -4.751 | -7.253 | 0.948 |
| -8.014 | 7.361 | -8.819 | -49.289 | -5.589 | 2.276 | -7.328 | 0.194 | -7.395 | -4.501 | -7.142 | 0.665 |
| -7.901 | 7.266 | -8.705 | -48.280 | -5.479 | 2.290 | -7.217 | 0.218 | -7.283 | -4.302 | -7.030 | 0.386 |
| -7.789 | 7.152 | -8.592 | -47.275 | -5.368 | 2.266 | -7.105 | 0.219 | -7.171 | -3.982 | -6.919 | 0.147 |
| -7.676 | 7.086 | -8.479 | -46.204 | -5.257 | 2.235 | -6.993 | 0.155 | -7.059 | -3.816 | -6.808 | -0.164 |
| -7.563 | 7.016 | -8.365 | -45.227 | -5.147 | 2.134 | -6.881 | 0.197 | -6.947 | -3.617 | -6.697 | -0.256 |
| -7.451 | 6.903 | -8.252 | -44.205 | -5.036 | 2.040 | -6.769 | 0.157 | -6.835 | -3.345 | -6.586 | -0.286 |
| -7.338 | 6.793 | -8.139 | -43.224 | -4.925 | 1.992 | -6.657 | 0.064 | -6.723 | -3.135 | -6.475 | -0.276 |
| -7.226 | 6.719 | -8.025 | -42.214 | -4.814 | 1.877 | -6.545 | 0.050 | -6.611 | -2.920 | -6.364 | -0.273 |
| -7.113 | 6.571 | -7.912 | -41.197 | -4.704 | 1.770 | -6.433 | 0.115 | -6.499 | -2.662 | -6.253 | -0.259 |
| -7.001 | 6.518 | -7.799 | -40.199 | -4.593 | 1.752 | -6.321 | 0.037 | -6.387 | -2.432 | -6.141 | -0.237 |
| -6.888 | 6.412 | -7.685 | -39.137 | -4.482 | 1.726 | -6.209 | 0.074 | -6.275 | -2.164 | -6.030 | -0.218 |
| -6.775 | 6.348 | -7.572 | -38.137 | -4.372 | 1.625 | -6.097 | 0.029 | -6.164 | -1.980 | -5.919 | -0.257 |
| -6.663 | 6.235 | -7.459 | -37.112 | -4.261 | 1.542 | -5.985 | 0.018 | -6.052 | -1.730 | -5.808 | -0.264 |
| -6.550 | 6.084 | -7.345 | -36.117 | -4.150 | 1.512 | -5.873 | -0.037 | -5.940 | -1.465 | -5.697 | -0.225 |
| -6.438 | 5.947 | -7.232 | -35.090 | -4.040 | 1.386 | -5.761 | -0.019 | -5.828 | -1.274 | -5.586 | -0.184 |
| -6.325 | 5.871 | -7.119 | -34.052 | -3.929 | 1.343 | -5.649 | -0.062 | -5.716 | -1.038 | -5.475 | -0.159 |
| -6.213 | 5.723 | -7.006 | -32.989 | -3.818 | 1.208 | -5.537 | -0.063 | -5.604 | -0.783 | -5.363 | -0.186 |
| -6.100 | 5.582 | -6.892 | -32.018 | -3.707 | 1.200 | -5.425 | -0.012 | -5.492 | -0.537 | -5.252 | -0.163 |
| -5.987 | 5.480 | -6.779 | -30.956 | -3.597 | 1.111 | -5.313 | -0.108 | -5.380 | -0.380 | -5.141 | -0.171 |
| -5.875 | 5.285 | -6.666 | -29.869 | -3.486 | 1.056 | -5.201 | -0.054 | -5.268 | -0.354 | -5.030 | -0.194 |
| -5.762 | 5.306 | -6.552 | -28.863 | -3.375 | 0.928 | -5.089 | -0.073 | -5.156 | -0.379 | -4.919 | -0.153 |
| -5.650 | 5.142 | -6.439 | -27.807 | -3.265 | 0.935 | -4.977 | -0.067 | -5.044 | -0.400 | -4.808 | -0.134 |
| -5.537 | 5.062 | -6.326 | -26.784 | -3.154 | 0.850 | -4.865 | -0.094 | -4.932 | -0.347 | -4.697 | -0.104 |
| -5.425 | 4.917 | -6.212 | -25.770 | -3.043 | 0.770 | -4.753 | -0.038 | -4.820 | -0.337 | -4.586 | -0.155 |
| -5.312 | 4.837 | -6.099 | -24.755 | -2.933 | 0.683 | -4.641 | -0.095 | -4.708 | -0.397 | -4.474 | -0.127 |
| -5.199 | 4.782 | -5.986 | -23.700 | -2.822 | 0.671 | -4.529 | -0.056 | -4.596 | -0.352 | -4.363 | -0.065 |
| -5.087 | 4.680 | -5.872 | -22.598 | -2.711 | 0.589 | -4.417 | -0.072 | -4.484 | -0.352 | -4.252 | -0.045 |
| -4.974 | 4.571 | -5.759 | -21.649 | -2.601 | 0.459 | -4.305 | -0.097 | -4.373 | -0.377 | -4.141 | -0.097 |
| -4.862 | 4.505 | -5.646 | -20.664 | -2.490 | 0.391 | -4.193 | -0.105 | -4.261 | -0.371 | -4.030 | -0.106 |
| -4.749 | 4.341 | -5.532 | -19.570 | -2.379 | 0.228 | -4.081 | -0.085 | -4.149 | -0.312 | -3.919 | -0.067 |
| -4.637 | 4.278 | -5.419 | -18.513 | -2.268 | 0.280 | -3.969 | -0.129 | -4.037 | -0.352 | -3.808 | -0.057 |
| -4.524 | 4.207 | -5.306 | -17.472 | -2.158 | 0.180 | -3.857 | -0.080 | -3.925 | -0.319 | -3.696 | -0.053 |
| -4.411 | 4.097 | -5.192 | -16.371 | -2.047 | 0.129 | -3.745 | -0.128 | -3.813 | -0.408 | -3.585 | -0.104 |
| -4.299 | 3.871 | -5.079 | -15.358 | -1.936 | 0.097 | -3.633 | -0.067 | -3.701 | -0.283 | -3.474 | -0.094 |
| -4.186 | 3.826 | -4.966 | -14.318 | -1.826 | 0.042 | -3.521 | -0.174 | -3.589 | -0.315 | -3.363 | -0.171 |
| -4.074 | 3.740 | -4.852 | -13.257 | -1.715 | -0.071 | -3.409 | -0.104 | -3.477 | -0.301 | -3.252 | -0.122 |
| -3.961 | 3.604 | -4.739 | -12.242 | -1.604 | -0.068 | -3.297 | -0.127 | -3.365 | -0.268 | -3.141 | -0.115 |
| -3.849 | 3.520 | -4.626 | -11.196 | -1.494 | -0.115 | -3.185 | -0.133 | -3.253 | -0.227 | -3.030 | -0.092 |
| -3.736 | 3.375 | -4.512 | -10.049 | -1.383 | -0.172 | -3.073 | -0.187 | -3.141 | -0.254 | -2.919 | -0.076 |
| -3.623 | 3.389 | -4.399 | -9.148 | -1.272 | -0.071 | -2.961 | -0.152 | -3.029 | -0.205 | -2.807 | -0.117 |
| -3.511 | 3.161 | -4.286 | -8.033 | -1.161 | -0.155 | -2.849 | -0.153 | -2.917 | -0.234 | -2.696 | -0.146 |
| -3.398 | 3.054 | -4.172 | -6.995 | -1.051 | -0.235 | -2.737 | -0.199 | -2.805 | -0.217 | -2.585 | -0.071 |
| -3.286 | 2.919 | -4.059 | -5.921 | -0.940 | -0.175 | -2.625 | -0.124 | -2.693 | -0.268 | -2.474 | -0.171 |
| -3.173 | 2.859 | -3.946 | -5.030 | -0.829 | -0.108 | -2.513 | -0.158 | -2.582 | -0.186 | -2.363 | -0.124 |
| -3.061 | 2.769 | -3.833 | -3.843 | -0.719 | -0.061 | -2.401 | -0.153 | -2.470 | -0.199 | -2.252 | -0.099 |
| -2.948 | 2.590 | -3.719 | -2.918 | -0.608 | -0.056 | -2.289 | -0.169 | -2.358 | -0.161 | -2.141 | -0.147 |
| -2.835 | 2.505 | -3.606 | -1.799 | -0.497 | -0.025 | -2.178 | -0.161 | -2.246 | -0.208 | -2.029 | -0.169 |
| -2.723 | 2.502 | -3.493 | -0.762 | -0.387 | 0.209 | -2.066 | -0.168 | -2.134 | -0.146 | -1.918 | -0.163 |
| -2.610 | 2.397 | -3.379 | -0.086 | -0.276 | 0.251 | -1.954 | -0.134 | -2.022 | -0.160 | -1.807 | -0.154 |
| -2.498 | 2.154 | -3.266 | -0.095 | -0.165 | 0.632 | -1.842 | -0.193 | -1.910 | -0.153 | -1.696 | -0.140 |
| -2.385 | 2.149 | -3.153 | -0.090 | -0.055 | 0.606 | -1.730 | -0.114 | -1.798 | -0.095 | -1.585 | -0.090 |
| -2.273 | 1.980 | -3.039 | -0.151 | 0.056 | 0.757 | -1.618 | -0.181 | -1.686 | -0.164 | -1.474 | -0.154 |
| -2.160 | 1.917 | -2.926 | -0.132 | 0.167 | 1.184 | -1.506 | -0.118 | -1.574 | -0.045 | -1.363 | -0.180 |
| -2.047 | 1.734 | -2.813 | -0.149 | 0.278 | 1.241 | -1.394 | -0.060 | -1.462 | -0.084 | -1.252 | -0.247 |
| -1.935 | 1.717 | -2.699 | -0.050 | 0.388 | 1.879 | -1.282 | -0.051 | -1.350 | -0.135 | -1.140 | -0.201 |
| -1.822 | 1.633 | -2.586 | -0.095 | 0.499 | 1.765 | -1.170 | 0.014 | -1.238 | 0.051 | -1.029 | -0.134 |
| -1.710 | 1.505 | -2.473 | -0.185 | 0.610 | 2.243 | -1.058 | 0.082 | -1.126 | 0.095 | -0.918 | -0.141 |
| -1.597 | 1.449 | -2.359 | -0.116 | 0.720 | 2.880 | -0.946 | 0.101 | -1.014 | 0.076 | -0.807 | -0.141 |
| -1.485 | 1.348 | -2.246 | -0.161 | 0.831 | 3.573 | -0.834 | 0.316 | -0.902 | 0.134 | -0.696 | -0.124 |
| -1.372 | 1.202 | -2.133 | -0.138 | 0.942 | 3.779 | -0.722 | 0.106 | -0.791 | 0.279 | -0.585 | -0.061 |
| -1.259 | 1.101 | -2.019 | -0.203 | 1.052 | 6.141 | -0.610 | 0.457 | -0.679 | 0.312 | -0.474 | -0.135 |
| -1.147 | 1.099 | -1.906 | -0.072 | 1.163 | 6.492 | -0.498 | 0.577 | -0.567 | 0.403 | -0.362 | 0.003 |
| -1.034 | 0.915 | -1.793 | -0.080 | 1.274 | 6.096 | -0.386 | 0.693 | -0.455 | 0.549 | -0.251 | 0.233 |
| -0.922 | 0.738 | -1.679 | -0.011 | 1.385 | 5.829 | -0.274 | 0.395 | -0.343 | 0.502 | -0.140 | 0.215 |
| -0.809 | 0.679 | -1.566 | 0.036 | 1.495 | 7.438 | -0.162 | 0.864 | -0.231 | 0.613 | -0.029 | 0.302 |
| -0.697 | 0.911 | -1.453 | 0.059 | 1.606 | 8.605 | -0.050 | 1.232 | -0.119 | 1.026 | 0.082 | 0.802 |
| -0.584 | 0.612 | -1.339 | 0.030 | 1.717 | 8.604 | 0.062 | 1.688 | -0.007 | 1.274 | 0.193 | 0.652 |
| -0.471 | 0.793 | -1.226 | 0.069 | 1.827 | 9.357 | 0.174 | 1.950 | 0.105 | 1.459 | 0.304 | 1.267 |
| -0.359 | 0.665 | -1.113 | 0.203 | 1.938 | 11.087 | 0.286 | 2.262 | 0.217 | 1.583 | 0.415 | 1.618 |
| -0.246 | 0.783 | -0.999 | 0.297 | 2.049 | 12.668 | 0.398 | 2.536 | 0.329 | 1.949 | 0.527 | 2.205 |
| -0.134 | 0.931 | -0.886 | 0.415 | 2.159 | 15.539 | 0.510 | 2.921 | 0.441 | 1.977 | 0.638 | 2.030 |
| -0.021 | 0.878 | -0.773 | 0.391 | 2.270 | 17.726 | 0.622 | 2.728 | 0.553 | 2.144 | 0.749 | 2.598 |
| 0.091 | 0.805 | -0.660 | 0.713 | 2.381 | 18.844 | 0.734 | 3.534 | 0.665 | 2.874 | 0.860 | 3.173 |
| 0.204 | 0.537 | -0.546 | 0.473 | 2.492 | 20.419 | 0.846 | 3.934 | 0.777 | 3.088 | 0.971 | 3.674 |
| 0.317 | 1.035 | -0.433 | 1.271 | 2.602 | 21.988 | 0.958 | 4.298 | 0.889 | 3.806 | 1.082 | 3.308 |
| 0.429 | 1.033 | -0.320 | 1.100 | 2.713 | 21.529 | 1.070 | 5.149 | 1.000 | 3.610 | 1.193 | 4.019 |
| 0.542 | 1.342 | -0.206 | 1.649 | 2.824 | 23.760 | 1.182 | 5.932 | 1.112 | 4.671 | 1.305 | 4.644 |
| 0.654 | 2.039 | -0.093 | 1.588 | 2.934 | 24.533 | 1.294 | 6.694 | 1.224 | 5.683 | 1.416 | 5.133 |
| 0.767 | 2.174 | 0.020 | 2.737 | 3.045 | 25.962 | 1.406 | 7.288 | 1.336 | 5.936 | 1.527 | 6.155 |
| 0.879 | 2.689 | 0.134 | 2.955 | 3.156 | 27.651 | 1.518 | 7.889 | 1.448 | 6.561 | 1.638 | 6.364 |
| 0.992 | 2.756 | 0.247 | 3.300 | 3.266 | 28.504 | 1.630 | 8.438 | 1.560 | 7.469 | 1.749 | 7.962 |
| 1.105 | 3.605 | 0.360 | 3.328 | 3.377 | 31.255 | 1.742 | 8.922 | 1.672 | 7.521 | 1.860 | 8.266 |
| 1.217 | 3.711 | 0.474 | 4.141 | 3.488 | 33.234 | 1.854 | 9.641 | 1.784 | 8.481 | 1.971 | 8.832 |
| 1.330 | 4.239 | 0.587 | 4.092 | 3.598 | 35.011 | 1.966 | 10.264 | 1.896 | 9.007 | 2.082 | 9.945 |
| 1.442 | 4.803 | 0.700 | 5.158 | 3.709 | 36.317 | 2.078 | 10.982 | 2.008 | 9.326 | 2.194 | 11.039 |
| 1.555 | 5.555 | 0.814 | 5.513 | 3.820 | 38.061 | 2.190 | 12.003 | 2.120 | 10.649 | 2.305 | 12.150 |
| 1.667 | 5.693 | 0.927 | 5.781 | 3.931 | 39.918 | 2.302 | 12.629 | 2.232 | 11.310 | 2.416 | 12.482 |
| 1.780 | 7.053 | 1.040 | 5.583 | 4.041 | 40.967 | 2.414 | 13.152 | 2.344 | 11.864 | 2.527 | 13.661 |
| 1.892 | 7.745 | 1.154 | 7.203 | 4.152 | 41.838 | 2.526 | 13.998 | 2.456 | 12.489 | 2.638 | 15.015 |
| 2.005 | 7.878 | 1.267 | 7.186 | 4.263 | 43.589 | 2.638 | 14.399 | 2.568 | 13.066 | 2.749 | 15.571 |
| 2.118 | 8.430 | 1.380 | 8.545 | 4.373 | 45.222 | 2.750 | 14.948 | 2.680 | 13.753 | 2.860 | 16.801 |
| 2.230 | 9.591 | 1.494 | 8.248 | 4.484 | 46.083 | 2.862 | 15.713 | 2.791 | 14.401 | 2.972 | 17.462 |
| 2.343 | 9.346 | 1.607 | 9.307 | 4.595 | 48.589 | 2.973 | 16.358 | 2.903 | 15.259 | 3.083 | 18.359 |
| 2.455 | 10.836 | 1.720 | 10.492 | 4.705 | 49.523 | 3.085 | 16.955 | 3.015 | 16.299 | 3.194 | 19.034 |
| 2.568 | 11.420 | 1.834 | 11.114 | 4.816 | 52.017 | 3.197 | 18.032 | 3.127 | 16.984 | 3.305 | 20.345 |
| 2.680 | 11.960 | 1.947 | 12.735 | 4.927 | 54.290 | 3.309 | 18.693 | 3.239 | 17.701 | 3.416 | 20.910 |
| 2.793 | 12.807 | 2.060 | 13.046 | 5.038 | 55.197 | 3.421 | 19.447 | 3.351 | 18.599 | 3.527 | 21.762 |
| 2.906 | 14.060 | 2.174 | 13.395 | 5.148 | 56.051 | 3.533 | 20.366 | 3.463 | 19.187 | 3.638 | 23.181 |
| 3.018 | 14.440 | 2.287 | 14.200 | 5.259 | 57.475 | 3.645 | 21.286 | 3.575 | 20.120 | 3.749 | 23.950 |
| 3.131 | 14.872 | 2.400 | 15.794 | 5.370 | 59.080 | 3.757 | 22.548 | 3.687 | 20.836 | 3.861 | 25.076 |
| 3.243 | 15.959 | 2.514 | 16.533 | 5.480 | 62.031 | 3.869 | 23.297 | 3.799 | 21.605 | 3.972 | 25.894 |
| 3.356 | 17.762 | 2.627 | 17.462 | 5.591 | 62.388 | 3.981 | 24.120 | 3.911 | 22.523 | 4.083 | 26.485 |
| 3.468 | 18.229 | 2.740 | 17.863 | 5.702 | 63.821 | 4.093 | 24.972 | 4.023 | 23.153 | 4.194 | 27.630 |
| 3.581 | 18.522 | 2.853 | 18.244 | 5.812 | 65.567 | 4.205 | 26.000 | 4.135 | 24.139 | 4.305 | 28.771 |
| 3.694 | 19.454 | 2.967 | 19.723 | 5.923 | 67.284 | 4.317 | 26.798 | 4.247 | 25.100 | 4.416 | 30.177 |
| 3.806 | 20.240 | 3.080 | 21.440 | 6.034 | 69.301 | 4.429 | 27.435 | 4.359 | 25.643 | 4.527 | 31.308 |
| 3.919 | 21.012 | 3.193 | 22.206 | 6.144 | 70.889 | 4.541 | 28.231 | 4.471 | 26.520 | 4.638 | 32.160 |
| 4.031 | 22.046 | 3.307 | 23.709 | 6.255 | 72.575 | 4.653 | 29.115 | 4.582 | 27.716 | 4.750 | 32.809 |
| 4.144 | 22.463 | 3.420 | 24.436 | 6.366 | 74.424 | 4.765 | 30.289 | 4.694 | 28.775 | 4.861 | 34.336 |
| 4.256 | 23.350 | 3.533 | 25.807 | 6.477 | 75.340 | 4.877 | 30.986 | 4.806 | 29.105 | 4.972 | 36.018 |
| 4.369 | 25.042 | 3.647 | 26.533 | 6.587 | 77.329 | 4.989 | 32.228 | 4.918 | 30.222 | 5.083 | 36.995 |
| 4.482 | 26.118 | 3.760 | 27.139 | 6.698 | 79.216 | 5.101 | 33.260 | 5.030 | 31.119 | 5.194 | 37.838 |
| 4.594 | 27.097 | 3.873 | 27.818 | 6.809 | 81.424 | 5.213 | 34.125 | 5.142 | 32.011 | 5.305 | 38.719 |
| 4.707 | 27.754 | 3.987 | 29.037 | 6.919 | 83.343 | 5.325 | 34.915 | 5.254 | 33.038 | 5.416 | 39.961 |
| 4.819 | 28.998 | 4.100 | 30.870 | 7.030 | 85.362 | 5.437 | 35.872 | 5.366 | 33.917 | 5.528 | 41.823 |
| 4.932 | 30.557 | 4.213 | 32.193 | 7.141 | 87.651 | 5.549 | 36.857 | 5.478 | 35.079 | 5.639 | 41.939 |
| 5.044 | 31.650 | 4.327 | 33.537 | 7.251 | 88.901 | 5.661 | 37.997 | 5.590 | 36.024 | 5.750 | 43.739 |
| 5.157 | 32.577 | 4.440 | 34.440 | 7.362 | 90.636 | 5.773 | 39.591 | 5.702 | 36.856 | 5.861 | 45.232 |
| 5.270 | 33.210 | 4.553 | 35.204 | 7.473 | 93.178 | 5.885 | 40.675 | 5.814 | 37.780 | 5.972 | 46.374 |
| 5.382 | 33.853 | 4.667 | 36.450 | 7.584 | 94.883 | 5.997 | 41.866 | 5.926 | 39.054 | 6.083 | 47.105 |
| 5.495 | 34.635 | 4.780 | 38.634 | 7.694 | 96.731 | 6.109 | 43.236 | 6.038 | 40.193 | 6.194 | 48.208 |
| 5.607 | 35.757 | 4.893 | 39.539 | 7.805 | 97.735 | 6.221 | 44.301 | 6.150 | 41.201 | 6.305 | 49.679 |
| 5.720 | 36.401 | 5.007 | 40.665 | 7.916 | 99.857 | 6.333 | 44.603 | 6.262 | 41.880 | 6.417 | 51.335 |
| 5.832 | 37.479 | 5.120 | 42.760 | 8.026 | 101.574 | 6.445 | 45.392 | 6.373 | 42.794 | 6.528 | 52.858 |
| 5.945 | 38.454 | 5.233 | 43.722 | 8.137 | 103.880 | 6.557 | 46.388 | 6.485 | 43.575 | 6.639 | 53.728 |
| 6.058 | 39.673 | 5.347 | 44.445 | 8.248 | 106.536 | 6.669 | 47.602 | 6.597 | 44.468 | 6.750 | 54.853 |
| 6.170 | 41.189 | 5.460 | 46.063 | 8.358 | 108.735 | 6.781 | 48.861 | 6.709 | 45.469 | 6.861 | 56.162 |
| 6.283 | 42.104 | 5.573 | 47.439 | 8.469 | 110.631 | 6.893 | 50.103 | 6.821 | 46.991 | 6.972 | 57.246 |
| 6.395 | 43.116 | 5.687 | 49.058 | 8.580 | 112.368 | 7.005 | 51.708 | 6.933 | 48.318 | 7.083 | 58.463 |
| 6.508 | 44.589 | 5.800 | 50.325 | 8.690 | 114.157 | 7.117 | 52.992 | 7.045 | 49.154 | 7.195 | 60.039 |
| 6.620 | 45.961 | 5.913 | 51.005 | 8.801 | 116.240 | 7.229 | 53.871 | 7.157 | 50.356 | 7.306 | 61.037 |
| 6.733 | 47.006 | 6.026 | 52.217 | 8.912 | 118.327 | 7.341 | 54.575 | 7.269 | 51.567 | 7.417 | 62.091 |
| 6.846 | 47.980 | 6.140 | 53.517 | 9.023 | 120.115 | 7.453 | 55.708 | 7.381 | 52.945 | 7.528 | 63.161 |
| 6.958 | 49.004 | 6.253 | 55.051 | 9.133 | 122.348 | 7.565 | 56.859 | 7.493 | 54.098 | 7.639 | 65.213 |
| 7.071 | 51.102 | 6.366 | 55.593 | 9.244 | 124.321 | 7.677 | 58.034 | 7.605 | 55.117 | 7.750 | 66.758 |
| 7.183 | 52.426 | 6.480 | 57.424 | 9.355 | 126.144 | 7.789 | 58.987 | 7.717 | 56.012 | 7.861 | 68.387 |
| 7.296 | 53.756 | 6.593 | 59.321 | 9.465 | 129.108 | 7.901 | 60.049 | 7.829 | 56.886 | 7.972 | 69.930 |
| 7.408 | 54.816 | 6.706 | 60.797 | 9.576 | 131.057 | 8.012 | 60.997 | 7.941 | 57.845 | 8.084 | 71.007 |
| 7.521 | 56.115 | 6.820 | 61.907 | 9.687 | 133.479 | 8.124 | 62.248 | 8.053 | 58.757 | 8.195 | 71.781 |
| 7.634 | 57.370 | 6.933 | 63.966 | 9.797 | 135.713 | 8.236 | 63.583 | 8.164 | 60.061 | 8.306 | 73.082 |
| 7.746 | 58.504 | 7.046 | 65.405 | 9.908 | 137.614 | 8.348 | 65.039 | 8.276 | 61.339 | 8.417 | 74.424 |
| 7.859 | 59.564 | 7.160 | 66.981 | 10.019 | 138.952 | 8.460 | 66.049 | 8.388 | 62.201 | 8.528 | 75.906 |
| 7.971 | 60.899 | 7.273 | 69.048 | 10.130 | 141.204 | 8.572 | 67.064 | 8.500 | 63.039 | 8.639 | 77.128 |
| 8.084 | 61.830 | 7.386 | 69.426 | 10.240 | 143.483 | 8.684 | 68.257 | 8.612 | 64.052 | 8.750 | 78.762 |
| 8.196 | 62.681 | 7.500 | 71.959 | 10.351 | 145.763 | 8.796 | 69.474 | 8.724 | 65.152 | 8.862 | 80.252 |
| 8.309 | 63.966 | 7.613 | 72.630 | 10.462 | 148.434 | 8.908 | 70.490 | 8.836 | 66.016 | 8.973 | 81.547 |
| 8.422 | 65.227 | 7.726 | 73.887 | 10.572 | 150.200 | 9.020 | 71.695 | 8.948 | 67.073 | 9.084 | 82.615 |
| 8.534 | 66.693 | 7.840 | 74.034 | 10.683 | 152.194 | 9.132 | 72.895 | 9.060 | 68.372 | 9.195 | 84.063 |
| 8.647 | 67.566 | 7.953 | 76.996 | 10.794 | 154.046 | 9.244 | 73.985 | 9.172 | 69.565 | 9.306 | 85.654 |
| 8.759 | 68.656 | 8.066 | 78.793 | 10.904 | 156.308 | 9.356 | 75.560 | 9.284 | 70.756 | 9.417 | 86.992 |
| 8.872 | 70.058 | 8.180 | 81.404 | 11.015 | 158.793 | 9.468 | 76.867 | 9.396 | 71.653 | 9.528 | 88.183 |
| 8.984 | 71.932 | 8.293 | 83.774 | 11.126 | 161.080 | 9.580 | 78.059 | 9.508 | 72.677 | 9.639 | 89.572 |
| 9.097 | 73.092 | 8.406 | 84.478 | 11.236 | 163.452 | 9.692 | 79.193 | 9.620 | 73.836 | 9.751 | 90.915 |
| 9.210 | 74.386 | 8.520 | 86.662 | 11.347 | 166.140 | 9.804 | 80.618 | 9.732 | 75.131 | 9.862 | 92.208 |
| 9.322 | 75.989 | 8.633 | 88.865 | 11.458 | 167.799 | 9.916 | 81.676 | 9.844 | 76.182 | 9.973 | 93.566 |
| 9.435 | 77.378 | 8.746 | 89.228 | 11.569 | 169.287 | 10.028 | 82.655 | 9.955 | 77.167 | 10.084 | 95.085 |
| 9.547 | 78.235 | 8.860 | 91.588 | 11.679 | 171.140 | 10.140 | 84.099 | 10.067 | 78.173 | 10.195 | 96.902 |
| 9.660 | 79.672 | 8.973 | 93.191 | 11.790 | 173.714 | 10.252 | 85.462 | 10.179 | 79.130 | 10.306 | 98.859 |
| 9.772 | 80.870 | 9.086 | 94.205 | 11.901 | 176.382 | 10.364 | 86.870 | 10.291 | 80.477 | 10.417 | 100.163 |
| 9.885 | 81.845 | 9.199 | 95.568 | 12.011 | 179.082 | 10.476 | 88.150 | 10.403 | 81.713 | 10.529 | 101.230 |
| 9.998 | 83.707 | 9.313 | 96.303 | 12.122 | 181.208 | 10.588 | 89.462 | 10.515 | 83.077 | 10.640 | 102.604 |
| 10.110 | 85.378 | 9.426 | 97.723 | 12.233 | 183.314 | 10.700 | 90.656 | 10.627 | 84.189 | 10.751 | 104.729 |
| 10.223 | 86.741 | 9.539 | 99.857 | 12.343 | 185.799 | 10.812 | 91.690 | 10.739 | 85.533 | 10.862 | 106.115 |
| 10.335 | 87.778 | 9.653 | 101.429 | 12.454 | 187.958 | 10.924 | 92.753 | 10.851 | 87.024 | 10.973 | 107.419 |
| 10.448 | 89.002 | 9.766 | 102.775 | 12.565 | 191.726 | 11.036 | 93.881 | 10.963 | 88.377 | 11.084 | 109.008 |
| 10.560 | 90.853 | 9.879 | 104.521 | 12.676 | 194.437 | 11.148 | 95.345 | 11.075 | 89.603 | 11.195 | 110.505 |
| 10.673 | 92.538 | 9.993 | 106.280 | 12.786 | 196.560 | 11.260 | 96.340 | 11.187 | 90.845 | 11.306 | 111.985 |
| 10.786 | 93.709 | 10.106 | 107.464 | 12.897 | 198.645 | 11.372 | 97.658 | 11.299 | 92.026 | 11.418 | 113.476 |
| 10.898 | 94.635 | 10.219 | 109.725 | 13.008 | 200.617 | 11.484 | 99.031 | 11.411 | 93.027 | 11.529 | 115.202 |
| 11.011 | 96.562 | 10.333 | 111.255 | 13.118 | 203.206 | 11.596 | 100.151 | 11.523 | 94.126 | 11.640 | 116.723 |
| 11.123 | 98.000 | 10.446 | 112.908 | 13.229 | 205.672 | 11.708 | 101.470 | 11.635 | 95.224 | 11.751 | 118.197 |
| 11.236 | 99.033 | 10.559 | 114.673 | 13.340 | 208.118 | 11.820 | 103.045 | 11.746 | 96.599 | 11.862 | 119.490 |
| 11.348 | 100.473 | 10.673 | 116.654 | 13.450 | 211.557 | 11.932 | 104.653 | 11.858 | 98.060 | 11.973 | 121.006 |
| 11.461 | 102.021 | 10.786 | 119.349 | 13.561 | 214.674 | 12.044 | 105.981 | 11.970 | 99.229 | 12.084 | 122.443 |
| 11.574 | 104.363 | 10.899 | 121.478 | 13.672 | 216.923 | 12.156 | 107.089 | 12.082 | 100.811 | 12.196 | 123.863 |
| 11.686 | 105.816 | 11.013 | 123.158 | 13.782 | 218.470 | 12.268 | 108.281 | 12.194 | 102.151 | 12.307 | 125.686 |
| 11.799 | 107.242 | 11.126 | 124.678 | 13.893 | 220.587 | 12.380 | 109.529 | 12.306 | 103.419 | 12.418 | 127.244 |
| 11.911 | 107.992 | 11.239 | 126.231 | 14.004 | 222.505 | 12.492 | 110.892 | 12.418 | 104.336 | 12.529 | 128.865 |
| 12.024 | 109.643 | 11.353 | 128.429 | 14.115 | 225.294 | 12.604 | 112.031 | 12.530 | 105.787 | 12.640 | 130.087 |
| 12.136 | 111.524 | 11.466 | 131.116 | 14.225 | 227.372 | 12.716 | 113.178 | 12.642 | 106.871 | 12.751 | 131.535 |
| 12.249 | 113.263 | 11.579 | 132.387 | 14.336 | 229.868 | 12.828 | 114.284 | 12.754 | 108.589 | 12.862 | 133.127 |
| 12.362 | 114.352 | 11.693 | 134.689 | 14.447 | 232.395 | 12.940 | 115.655 | 12.866 | 110.107 | 12.973 | 134.922 |
| 12.474 | 115.693 | 11.806 | 137.951 | 14.557 | 234.369 | 13.051 | 116.800 | 12.978 | 111.607 | 13.085 | 137.270 |
| 12.587 | 117.577 | 11.919 | 138.148 | 14.668 | 236.487 | 13.163 | 118.101 | 13.090 | 112.998 | 13.196 | 139.331 |
| 12.699 | 118.926 | 12.033 | 139.985 | 14.779 | 238.105 | 13.275 | 119.500 | 13.202 | 114.207 | 13.307 | 140.736 |
| 12.812 | 120.477 | 12.146 | 141.845 | 14.889 | 240.886 | 13.387 | 120.587 | 13.314 | 115.599 | 13.418 | 141.829 |
| 12.924 | 122.075 | 12.259 | 143.744 | 15.000 | 243.570 | 13.499 | 122.193 | 13.426 | 116.520 | 13.529 | 143.776 |
| 13.037 | 123.374 | 12.372 | 145.503 | 15.111 | 245.860 | 13.611 | 123.568 | 13.537 | 117.886 | 13.640 | 145.867 |
| 13.150 | 124.422 | 12.486 | 146.917 | 15.222 | 248.330 | 13.723 | 124.887 | 13.649 | 119.372 | 13.751 | 146.227 |
| 13.262 | 125.078 | 12.599 | 149.237 | 15.332 | 251.633 | 13.835 | 126.349 | 13.761 | 120.838 | 13.863 | 148.128 |
| 13.375 | 126.230 | 12.712 | 152.121 | 15.443 | 253.265 | 13.947 | 127.370 | 13.873 | 122.085 | 13.974 | 150.476 |
| 13.487 | 128.451 | 12.826 | 154.536 | 15.554 | 255.925 | 14.059 | 128.972 | 13.985 | 123.344 | 14.085 | 152.935 |
| 13.600 | 129.908 | 12.939 | 156.144 | 15.664 | 258.394 | 14.171 | 130.808 | 14.097 | 124.605 | 14.196 | 155.330 |
| 13.712 | 131.500 | 13.052 | 158.065 | 15.775 | 260.482 | 14.283 | 132.336 | 14.209 | 126.145 | 14.307 | 157.140 |
| 13.825 | 133.128 | 13.166 | 159.793 | 15.886 | 262.443 | 14.395 | 133.092 | 14.321 | 127.399 | 14.418 | 158.752 |
| 13.937 | 134.464 | 13.279 | 161.828 | 15.996 | 265.249 | 14.507 | 134.599 | 14.433 | 128.715 | 14.529 | 160.489 |
| 14.050 | 136.252 | 13.392 | 163.515 | 16.107 | 267.885 | 14.619 | 136.240 | 14.545 | 130.276 | 14.640 | 161.916 |
| 14.163 | 137.588 | 13.506 | 165.636 | 16.218 | 270.016 | 14.731 | 138.090 | 14.657 | 131.663 | 14.752 | 163.690 |
| 14.275 | 138.867 | 13.619 | 168.452 | 16.329 | 272.195 | 14.843 | 139.779 | 14.769 | 132.927 | 14.863 | 165.512 |
| 14.388 | 140.503 | 13.732 | 170.290 | 16.439 | 273.660 | 14.955 | 140.738 | 14.881 | 134.200 | 14.974 | 167.088 |
| 14.500 | 141.998 | 13.846 | 172.971 | 16.550 | 275.217 | 15.067 | 141.958 | 14.993 | 135.403 | 15.085 | 168.530 |
| 14.613 | 143.836 | 13.959 | 174.508 | 16.661 | 278.568 | 15.179 | 143.306 | 15.105 | 136.637 | 15.196 | 170.125 |
| 14.725 | 144.791 | 14.072 | 176.868 | 16.771 | 282.215 | 15.291 | 144.830 | 15.217 | 138.040 | 15.307 | 172.308 |
| 14.838 | 146.615 | 14.186 | 178.588 | 16.882 | 285.823 | 15.403 | 146.310 | 15.328 | 139.360 | 15.418 | 174.393 |
| 14.951 | 148.457 | 14.299 | 180.227 | 16.993 | 289.863 | 15.515 | 147.701 | 15.440 | 140.554 | 15.530 | 176.611 |
| 15.063 | 149.939 | 14.412 | 181.560 | 17.103 | 291.620 | 15.627 | 149.115 | 15.552 | 141.826 | 15.641 | 178.488 |
| 15.176 | 151.193 | 14.526 | 183.871 | 17.214 | 294.008 | 15.739 | 150.999 | 15.664 | 143.393 | 15.752 | 180.393 |
| 15.288 | 152.273 | 14.639 | 185.734 | 17.325 | 296.343 | 15.851 | 152.859 | 15.776 | 144.801 | 15.863 | 181.897 |
| 15.401 | 154.031 | 14.752 | 188.556 | 17.435 | 298.684 | 15.963 | 154.063 | 15.888 | 146.083 | 15.974 | 183.746 |
| 15.513 | 155.944 | 14.866 | 190.491 | 17.546 | 300.852 | 16.075 | 155.405 | 16.000 | 148.133 | 16.085 | 185.396 |
| 15.626 | 157.513 | 14.979 | 193.212 | 17.657 | 302.942 | 16.187 | 156.697 | 16.112 | 149.486 | 16.196 | 187.212 |
| 15.739 | 159.156 | 15.092 | 194.382 | 17.768 | 305.246 | 16.299 | 158.187 | 16.224 | 150.811 | 16.307 | 189.578 |
| 15.851 | 160.971 | 15.206 | 196.925 | 17.878 | 307.301 | 16.411 | 159.878 | 16.336 | 152.468 | 16.419 | 191.328 |
| 15.964 | 162.777 | 15.319 | 199.631 | 17.989 | 310.525 | 16.523 | 161.490 | 16.448 | 154.051 | 16.530 | 193.378 |
| 16.076 | 164.282 | 15.432 | 201.805 | 18.100 | 313.667 | 16.635 | 162.898 | 16.560 | 155.481 | 16.641 | 195.431 |
| 16.189 | 166.517 | 15.546 | 203.708 | 18.210 | 315.278 | 16.747 | 164.264 | 16.672 | 156.802 | 16.752 | 197.400 |
| 16.301 | 168.692 | 15.659 | 205.564 | 18.321 | 317.922 | 16.859 | 165.792 | 16.784 | 158.476 | 16.863 | 199.520 |
| 16.414 | 170.754 | 15.772 | 208.184 | 18.432 | 319.838 | 16.971 | 167.703 | 16.896 | 159.922 | 16.974 | 201.730 |
| 16.527 | 172.919 | 15.885 | 211.046 | 18.542 | 321.718 | 17.083 | 169.450 | 17.008 | 162.218 | 17.085 | 203.323 |
| 16.639 | 174.490 | 15.999 | 213.671 | 18.653 | 323.896 | 17.195 | 170.864 | 17.119 | 163.969 | 17.196 | 205.242 |
| 16.752 | 175.903 | 16.112 | 216.203 | 18.764 | 327.165 | 17.307 | 172.230 | 17.231 | 165.579 | 17.308 | 207.329 |
| 16.864 | 177.147 | 16.225 | 217.701 | 18.875 | 329.473 | 17.419 | 173.665 | 17.343 | 166.864 | 17.419 | 209.463 |
| 16.977 | 178.156 | 16.339 | 219.203 | 18.985 | 331.885 | 17.531 | 175.526 | 17.455 | 168.345 | 17.530 | 211.821 |
| 17.089 | 180.165 | 16.452 | 222.337 | 19.096 | 334.617 | 17.643 | 176.886 | 17.567 | 169.727 | 17.641 | 213.223 |
| 17.202 | 182.400 | 16.565 | 225.243 | 19.207 | 338.322 | 17.755 | 178.288 | 17.679 | 171.296 | 17.752 | 215.182 |
| 17.315 | 184.049 | 16.679 | 227.765 | 19.317 | 341.129 | 17.867 | 180.469 | 17.791 | 172.673 | 17.863 | 217.427 |
| 17.427 | 186.305 | 16.792 | 230.624 | 19.428 | 343.691 | 17.979 | 182.124 | 17.903 | 174.105 | 17.974 | 219.102 |
| 17.540 | 187.997 | 16.905 | 233.249 | 19.539 | 347.228 | 18.090 | 183.884 | 18.015 | 175.526 | 18.086 | 220.447 |
| 17.652 | 189.728 | 17.019 | 234.308 | 19.649 | 349.201 | 18.202 | 185.162 | 18.127 | 176.971 | 18.197 | 222.681 |
| 17.765 | 191.639 | 17.132 | 235.906 | 19.760 | 351.710 | 18.314 | 186.795 | 18.239 | 178.367 | 18.308 | 225.216 |
| 17.877 | 193.880 | 17.245 | 237.993 | 19.871 | 353.559 | 18.426 | 188.289 | 18.351 | 179.389 | 18.419 | 227.450 |
| 17.990 | 195.671 | 17.359 | 240.413 | 19.981 | 355.673 | 18.538 | 189.435 | 18.463 | 180.622 | 18.530 | 229.392 |
| 18.103 | 197.283 | 17.472 | 243.274 | 20.092 | 358.112 | 18.650 | 191.284 | 18.575 | 182.409 | 18.641 | 231.480 |
| 18.215 | 199.411 | 17.585 | 245.459 | 20.203 | 360.680 | 18.762 | 192.441 | 18.687 | 184.019 | 18.752 | 233.635 |
| 18.328 | 201.430 | 17.699 | 247.969 | 20.314 | 364.505 | 18.874 | 194.203 | 18.799 | 185.386 | 18.863 | 235.010 |
| 18.440 | 203.405 | 17.812 | 251.476 | 20.424 | 367.769 | 18.986 | 195.886 | 18.910 | 187.057 | 18.975 | 237.247 |
| 18.553 | 205.347 | 17.925 | 253.727 | 20.535 | 370.330 | 19.098 | 197.202 | 19.022 | 188.842 | 19.086 | 239.599 |
| 18.665 | 207.536 | 18.039 | 255.368 | 20.646 | 372.875 | 19.210 | 198.306 | 19.134 | 190.600 | 19.197 | 241.644 |
| 18.778 | 209.080 | 18.152 | 257.876 | 20.756 | 375.813 | 19.322 | 199.971 | 19.246 | 192.252 | 19.308 | 243.584 |
| 18.891 | 210.583 | 18.265 | 259.094 | 20.867 | 378.836 | 19.434 | 202.088 | 19.358 | 193.816 | 19.419 | 245.491 |
| 19.003 | 212.267 | 18.379 | 262.616 | 20.978 | 381.684 | 19.546 | 203.775 | 19.470 | 195.573 | 19.530 | 246.986 |
| 19.116 | 214.453 | 18.492 | 264.734 | 21.088 | 384.309 | 19.658 | 205.402 | 19.582 | 197.093 | 19.641 | 248.798 |
| 19.228 | 216.818 | 18.605 | 267.138 | 21.199 | 388.255 | 19.770 | 207.162 | 19.694 | 198.545 | 19.753 | 251.218 |
| 19.341 | 219.588 | 18.719 | 269.731 | 21.310 | 391.499 | 19.882 | 209.041 | 19.806 | 200.057 | 19.864 | 253.368 |
| 19.453 | 222.296 | 18.832 | 272.980 | 21.421 | 394.277 | 19.994 | 211.181 | 19.918 | 201.716 | 19.975 | 254.977 |
| 19.566 | 223.952 | 18.945 | 275.840 | 21.531 | 396.822 | 20.106 | 212.922 | 20.030 | 203.726 | 20.086 | 257.212 |
| 19.679 | 225.230 | 19.058 | 278.960 | 21.642 | 398.804 | 20.218 | 214.513 | 20.142 | 205.645 | 20.197 | 259.754 |
| 19.791 | 227.215 | 19.172 | 281.252 | 21.753 | 400.831 | 20.330 | 216.105 | 20.254 | 206.938 | 20.308 | 262.279 |
| 19.904 | 229.138 | 19.285 | 283.250 | 21.863 | 403.351 | 20.442 | 218.280 | 20.366 | 208.335 | 20.419 | 264.852 |
| 20.016 | 230.944 | 19.398 | 285.835 | 21.974 | 406.211 | 20.554 | 219.811 | 20.478 | 209.867 | 20.530 | 266.517 |
| 20.129 | 232.332 | 19.512 | 287.997 | 22.085 | 407.713 | 20.666 | 221.681 | 20.590 | 211.450 | 20.642 | 268.073 |
| 20.241 | 234.685 | 19.625 | 289.424 | 22.195 | 410.129 | 20.778 | 223.286 | 20.701 | 213.393 | 20.753 | 270.351 |
| 20.354 | 236.224 | 19.738 | 292.949 | 22.306 | 412.720 | 20.890 | 224.751 | 20.813 | 214.866 | 20.864 | 272.574 |
| 20.467 | 239.001 | 19.852 | 295.323 | 22.417 | 417.598 | 21.002 | 226.660 | 20.925 | 216.319 | 20.975 | 274.521 |
| 20.579 | 240.923 | 19.965 | 298.063 | 22.527 | 420.655 | 21.114 | 228.375 | 21.037 | 217.665 | 21.086 | 277.040 |
| 20.692 | 242.545 | 20.078 | 301.038 | 22.638 | 423.520 | 21.226 | 230.191 | 21.149 | 219.292 | 21.197 | 279.201 |
| 20.804 | 244.263 | 20.192 | 302.866 | 22.749 | 427.271 | 21.338 | 231.938 | 21.261 | 220.936 | 21.308 | 281.132 |
| 20.917 | 246.039 | 20.305 | 305.384 | 22.860 | 430.110 | 21.450 | 233.613 | 21.373 | 222.264 | 21.420 | 283.025 |
| 21.029 | 247.632 | 20.418 | 307.590 | 22.970 | 432.269 | 21.562 | 235.317 | 21.485 | 224.102 | 21.531 | 284.861 |
| 21.142 | 248.133 | 20.532 | 311.502 | 23.081 | 434.361 | 21.674 | 236.862 | 21.597 | 226.078 | 21.642 | 286.647 |
| 21.255 | 249.812 | 20.645 | 314.314 | 23.192 | 436.815 | 21.786 | 239.074 | 21.709 | 227.642 | 21.753 | 288.975 |
| 21.367 | 252.086 | 20.758 | 317.304 | 23.302 | 439.332 | 21.898 | 241.140 | 21.821 | 229.361 | 21.864 | 291.193 |
| 21.480 | 253.680 | 20.872 | 319.909 | 23.413 | 441.515 | 22.010 | 242.901 | 21.933 | 231.642 | 21.975 | 293.807 |
| 21.592 | 256.203 | 20.985 | 322.431 | 23.524 | 444.074 | 22.122 | 244.081 | 22.045 | 233.286 | 22.086 | 296.273 |
| 21.705 | 259.701 | 21.098 | 325.290 | 23.634 | 446.218 | 22.234 | 245.957 | 22.157 | 235.153 | 22.197 | 298.269 |
| 21.817 | 262.234 | 21.212 | 328.271 | 23.745 | 449.028 | 22.346 | 247.954 | 22.269 | 236.976 | 22.309 | 300.713 |
| 21.930 | 264.751 | 21.325 | 330.302 | 23.856 | 452.403 | 22.458 | 249.500 | 22.381 | 238.544 | 22.420 | 302.926 |
| 22.043 | 266.454 | 21.438 | 332.412 | 23.967 | 454.883 | 22.570 | 250.622 | 22.492 | 240.169 | 22.531 | 305.285 |
| 22.155 | 268.172 | 21.552 | 335.129 | 24.077 | 460.334 | 22.682 | 252.711 | 22.604 | 241.863 | 22.642 | 307.253 |
| 22.268 | 269.831 | 21.665 | 337.656 | 24.188 | 463.108 | 22.794 | 254.869 | 22.716 | 243.548 | 22.753 | 308.896 |
| 22.380 | 271.279 | 21.778 | 340.225 | 24.299 | 466.364 | 22.906 | 257.087 | 22.828 | 245.397 | 22.864 | 310.767 |
| 22.493 | 273.468 | 21.892 | 343.810 | 24.409 | 468.772 | 23.018 | 258.830 | 22.940 | 246.864 | 22.975 | 313.293 |
| 22.605 | 275.867 | 22.005 | 347.251 | 24.520 | 470.787 | 23.129 | 260.477 | 23.052 | 248.533 | 23.087 | 315.802 |
| 22.718 | 277.674 | 22.118 | 349.149 | 24.631 | 473.085 | 23.241 | 262.352 | 23.164 | 250.686 | 23.198 | 318.258 |
| 22.831 | 278.884 | 22.231 | 350.967 | 24.741 | 476.849 | 23.353 | 264.159 | 23.276 | 252.065 | 23.309 | 320.476 |
| 22.943 | 281.956 | 22.345 | 352.970 | 24.852 | 478.747 | 23.465 | 265.311 | 23.388 | 253.267 | 23.420 | 322.875 |
| 23.056 | 284.545 | 22.458 | 354.991 | 24.963 | 481.251 | 23.577 | 267.313 | 23.500 | 254.922 | 23.531 | 325.229 |
| 23.168 | 287.078 | 22.571 | 358.141 | 25.073 | 485.201 | 23.689 | 268.772 | 23.612 | 256.280 | 23.642 | 327.116 |
| 23.281 | 288.585 | 22.685 | 359.927 | 25.184 | 488.466 | 23.801 | 270.373 | 23.724 | 257.738 | 23.753 | 329.676 |
| 23.393 | 290.599 | 22.798 | 361.713 | 25.295 | 491.112 | 23.913 | 272.728 | 23.836 | 259.507 | 23.864 | 331.940 |
| 23.506 | 292.247 | 22.911 | 363.919 | 25.406 | 494.623 | 24.025 | 275.043 | 23.948 | 261.254 | 23.976 | 333.982 |
| 23.619 | 294.597 | 23.025 | 366.418 | 25.516 | 498.390 | 24.137 | 276.941 | 24.060 | 262.219 | 24.087 | 336.362 |
| 23.731 | 296.729 | 23.138 | 368.743 | 25.627 | 500.674 | 24.249 | 278.648 | 24.172 | 264.302 | 24.198 | 337.966 |
| 23.844 | 299.315 | 23.251 | 371.188 | 25.738 | 502.252 | 24.361 | 279.910 | 24.283 | 265.971 | 24.309 | 339.772 |
| 23.956 | 301.196 | 23.365 | 373.258 | 25.848 | 505.408 | 24.473 | 281.812 | 24.395 | 268.155 | 24.420 | 341.698 |
| 24.069 | 303.404 | 23.478 | 376.175 | 25.959 | 508.443 | 24.585 | 283.534 | 24.507 | 269.945 | 24.531 | 343.583 |
| 24.181 | 305.084 | 23.591 | 378.338 | 26.070 | 510.946 | 24.697 | 285.372 | 24.619 | 271.532 | 24.642 | 345.618 |
| 24.294 | 306.972 | 23.705 | 380.182 | 26.180 | 513.815 | 24.809 | 287.484 | 24.731 | 273.211 | 24.754 | 348.884 |
| 24.407 | 308.980 | 23.818 | 383.283 | 26.291 | 515.508 | 24.921 | 289.159 | 24.843 | 275.209 | 24.865 | 350.010 |
| 24.519 | 311.675 | 23.931 | 385.320 | 26.402 | 518.033 | 25.033 | 291.449 | 24.955 | 276.785 | 24.976 | 352.594 |
| 24.632 | 314.401 | 24.045 | 388.621 | 26.513 | 520.143 | 25.145 | 293.098 | 25.067 | 278.429 | 25.087 | 355.096 |
| 24.744 | 315.819 | 24.158 | 391.649 | 26.623 | 523.171 | 25.257 | 294.875 | 25.179 | 280.068 | 25.198 | 358.012 |
| 24.857 | 317.541 | 24.271 | 394.948 | 26.734 | 526.641 | 25.369 | 296.740 | 25.291 | 281.787 | 25.309 | 360.191 |
| 24.969 | 319.548 | 24.385 | 399.127 | 26.845 | 529.715 | 25.481 | 298.406 | 25.403 | 283.734 | 25.420 | 362.218 |
| 25.082 | 321.902 | 24.498 | 402.722 | 26.955 | 534.205 | 25.593 | 300.142 | 25.515 | 286.077 | 25.531 | 364.231 |
| 25.195 | 324.191 | 24.611 | 403.664 | 27.066 | 537.262 | 25.705 | 302.031 | 25.627 | 288.072 | 25.643 | 366.298 |
| 25.307 | 326.507 | 24.725 | 405.527 | 27.177 | 539.407 | 25.817 | 303.829 | 25.739 | 290.137 | 25.754 | 368.386 |
| 25.420 | 329.593 | 24.838 | 407.773 | 27.287 | 542.287 | 25.929 | 306.001 | 25.851 | 292.128 | 25.865 | 370.127 |
| 25.532 | 332.419 | 24.951 | 410.210 | 27.398 | 544.330 | 26.041 | 307.878 | 25.963 | 294.296 | 25.976 | 372.109 |
| 25.645 | 334.990 | 25.065 | 413.286 | 27.509 | 548.792 | 26.153 | 309.528 | 26.074 | 295.918 | 26.087 | 374.693 |
| 25.757 | 338.095 | 25.178 | 417.403 | 27.619 | 553.024 | 26.265 | 311.050 | 26.186 | 297.371 | 26.198 | 377.588 |
| 25.870 | 340.605 | 25.291 | 420.176 | 27.730 | 554.874 | 26.377 | 312.748 | 26.298 | 298.962 | 26.309 | 380.136 |
| 25.982 | 342.709 | 25.405 | 424.261 | 27.841 | 557.412 | 26.489 | 314.739 | 26.410 | 300.706 | 26.421 | 382.538 |
| 26.095 | 344.026 | 25.518 | 426.019 | 27.952 | 562.018 | 26.601 | 317.069 | 26.522 | 302.477 | 26.532 | 385.076 |
| 26.208 | 345.836 | 25.631 | 428.154 | 28.062 | 565.055 | 26.713 | 318.751 | 26.634 | 304.489 | 26.643 | 386.878 |
| 26.320 | 347.667 | 25.744 | 432.056 | 28.173 | 568.978 | 26.825 | 320.958 | 26.746 | 306.246 | 26.754 | 389.427 |
| 26.433 | 349.823 | 25.858 | 436.614 | 28.284 | 573.260 | 26.937 | 322.204 | 26.858 | 308.160 | 26.865 | 391.344 |
| 26.545 | 353.298 | 25.971 | 439.756 | 28.394 | 577.154 | 27.049 | 324.408 | 26.970 | 309.893 | 26.976 | 392.841 |
| 26.658 | 356.891 | 26.084 | 440.887 | 28.505 | 578.035 | 27.161 | 326.946 | 27.082 | 311.798 | 27.087 | 394.180 |
| 26.770 | 357.834 | 26.198 | 444.403 | 28.616 | 579.183 | 27.273 | 328.693 | 27.194 | 313.344 | 27.198 | 398.342 |
| 26.883 | 360.441 | 26.311 | 445.932 | 28.726 | 582.621 | 27.385 | 330.573 | 27.306 | 314.887 | 27.310 | 400.494 |
| 26.996 | 363.176 | 26.424 | 448.617 | 28.837 | 586.881 | 27.497 | 332.067 | 27.418 | 317.223 | 27.421 | 402.634 |
| 27.108 | 365.106 | 26.538 | 451.271 | 28.948 | 588.624 | 27.609 | 334.122 | 27.530 | 319.246 | 27.532 | 404.913 |
| 27.221 | 366.977 | 26.651 | 453.973 | 29.059 | 594.836 | 27.721 | 336.443 | 27.642 | 321.116 | 27.643 | 406.601 |
| 27.333 | 369.723 | 26.764 | 457.067 | 29.169 | 598.059 | 27.833 | 338.820 | 27.754 | 323.110 | 27.754 | 408.301 |
| 27.446 | 372.275 | 26.878 | 459.765 | 29.280 | 602.338 | 27.945 | 341.044 | 27.865 | 324.625 | 27.865 | 410.644 |
| 27.558 | 374.274 | 26.991 | 463.586 | 29.391 | 605.599 | 28.057 | 343.050 | 27.977 | 326.228 | 27.976 | 412.782 |
| 27.671 | 376.623 | 27.104 | 466.677 | 29.501 | 608.583 | 28.168 | 344.901 | 28.089 | 327.927 | 28.087 | 415.593 |
| 27.784 | 379.239 | 27.218 | 469.767 | 29.612 | 611.591 | 28.280 | 346.340 | 28.201 | 329.740 | 28.199 | 417.799 |
| 27.896 | 382.377 | 27.331 | 472.991 | 29.723 | 614.236 | 28.392 | 348.100 | 28.313 | 331.633 | 28.310 | 419.748 |
| 28.009 | 383.551 | 27.444 | 476.722 | 29.833 | 618.242 | 28.504 | 349.726 | 28.425 | 333.263 | 28.421 | 421.639 |
| 28.121 | 385.435 | 27.558 | 479.598 | 29.944 | 618.275 | 28.616 | 351.725 | 28.537 | 334.724 | 28.532 | 423.750 |
| 28.234 | 388.364 | 27.671 | 482.438 | 30.055 | 622.378 | 28.728 | 353.779 | 28.649 | 336.408 | 28.643 | 426.586 |
| 28.346 | 391.444 | 27.784 | 485.158 | 30.165 | 624.854 | 28.840 | 355.768 | 28.761 | 338.314 | 28.754 | 428.917 |
| 28.459 | 393.533 | 27.898 | 487.929 | 30.276 | 628.312 | 28.952 | 357.369 | 28.873 | 340.234 | 28.865 | 431.631 |
| 28.572 | 394.404 | 28.011 | 491.675 | 30.387 | 631.903 | 29.064 | 359.217 | 28.985 | 342.037 | 28.977 | 434.230 |
| 28.684 | 396.431 | 28.124 | 494.137 | 30.498 | 634.910 | 29.176 | 361.824 | 29.097 | 343.867 | 29.088 | 436.819 |
| 28.797 | 398.571 | 28.238 | 497.193 | 30.608 | 638.971 | 29.288 | 363.709 | 29.209 | 346.310 | 29.199 | 440.037 |
| 28.909 | 401.789 | 28.351 | 500.371 | 30.719 | 641.869 | 29.400 | 366.112 | 29.321 | 348.644 | 29.310 | 442.482 |
| 29.022 | 402.924 | 28.464 | 502.732 | 30.830 | 645.540 | 29.512 | 368.437 | 29.433 | 350.564 | 29.421 | 444.403 |
| 29.134 | 404.979 | 28.578 | 507.456 | 30.940 | 649.209 | 29.624 | 370.988 | 29.545 | 351.255 | 29.532 | 446.612 |
| 29.247 | 407.483 | 28.691 | 509.517 | 31.051 | 651.683 | 29.736 | 372.183 | 29.656 | 352.725 | 29.643 | 448.053 |
| 29.360 | 409.118 | 28.804 | 513.812 | 31.162 | 654.941 | 29.848 | 374.138 | 29.768 | 355.485 | 29.754 | 450.301 |
| 29.472 | 411.671 | 28.917 | 516.562 | 31.272 | 659.646 | 29.960 | 375.999 | 29.880 | 357.425 | 29.866 | 453.684 |
| 29.585 | 414.642 | 29.031 | 519.210 | 31.383 | 664.537 | 30.072 | 377.899 | 29.992 | 359.166 | 29.977 | 456.474 |
| 29.697 | 418.310 | 29.144 | 523.106 | 31.494 | 666.382 | 30.184 | 380.103 | 30.104 | 361.057 | 30.088 | 458.673 |
| 29.810 | 420.509 | 29.257 | 525.549 | 31.605 | 667.873 | 30.296 | 382.333 | 30.216 | 363.153 | 30.199 | 462.548 |
| 29.922 | 422.404 | 29.371 | 529.932 | 31.715 | 671.876 | 30.408 | 384.522 | 30.328 | 365.707 | 30.310 | 465.289 |
| 30.035 | 424.354 | 29.484 | 532.877 | 31.826 | 674.714 | 30.520 | 386.728 | 30.440 | 367.563 | 30.421 | 467.215 |
| 30.148 | 426.476 | 29.597 | 536.798 | 31.937 | 680.090 | 30.632 | 389.026 | 30.552 | 369.704 | 30.532 | 469.340 |
| 30.260 | 428.900 | 29.711 | 539.518 | 32.047 | 683.861 | 30.744 | 391.342 | 30.664 | 371.910 | 30.644 | 471.921 |
| 30.373 | 431.193 | 29.824 | 541.745 | 32.158 | 687.622 | 30.856 | 393.124 | 30.776 | 373.815 | 30.755 | 474.928 |
| 30.485 | 434.284 | 29.937 | 543.883 | 32.269 | 689.875 | 30.968 | 395.084 | 30.888 | 376.086 | 30.866 | 477.776 |
| 30.598 | 435.797 | 30.051 | 545.984 | 32.379 | 691.140 | 31.080 | 397.023 | 31.000 | 378.255 | 30.977 | 479.954 |
| 30.710 | 438.449 | 30.164 | 548.069 | 32.490 | 695.115 | 31.192 | 399.223 | 31.112 | 379.530 | 31.088 | 482.562 |
| 30.823 | 441.106 | 30.277 | 549.836 | 32.601 | 698.550 | 31.304 | 401.032 | 31.224 | 381.089 | 31.199 | 484.264 |
| 30.936 | 442.521 | 30.391 | 552.788 | 32.711 | 701.898 | 31.416 | 403.397 | 31.336 | 382.760 | 31.310 | 487.127 |
| 31.048 | 444.671 | 30.504 | 556.633 | 32.822 | 704.683 | 31.528 | 404.924 | 31.447 | 384.385 | 31.421 | 488.654 |
| 31.161 | 447.044 | 30.617 | 560.840 | 32.933 | 709.097 | 31.640 | 407.423 | 31.559 | 386.791 | 31.533 | 491.622 |
| 31.273 | 449.167 | 30.731 | 564.732 | 33.044 | 712.991 | 31.752 | 409.517 | 31.671 | 388.755 | 31.644 | 493.954 |
| 31.386 | 452.860 | 30.844 | 567.516 | 33.154 | 716.877 | 31.864 | 412.495 | 31.783 | 390.857 | 31.755 | 496.600 |
| 31.498 | 455.655 | 30.957 | 571.265 | 33.265 | 720.931 | 31.976 | 414.625 | 31.895 | 393.490 | 31.866 | 499.955 |
| 31.611 | 457.560 | 31.071 | 574.844 | 33.376 | 725.666 | 32.088 | 416.996 | 32.007 | 395.562 | 31.977 | 502.917 |
| 31.724 | 459.923 | 31.184 | 577.605 | 33.486 | 729.119 | 32.200 | 418.822 | 32.119 | 397.198 | 32.088 | 505.431 |
| 31.836 | 462.712 | 31.297 | 581.566 | 33.597 | 732.603 | 32.312 | 421.029 | 32.231 | 398.788 | 32.199 | 508.063 |
| 31.949 | 465.479 | 31.411 | 585.106 | 33.708 | 735.623 | 32.424 | 422.888 | 32.343 | 400.974 | 32.311 | 510.914 |
| 32.061 | 468.652 | 31.524 | 586.390 | 33.818 | 738.844 | 32.536 | 425.046 | 32.455 | 402.802 | 32.422 | 513.611 |
| 32.174 | 470.994 | 31.637 | 589.674 | 33.929 | 742.696 | 32.648 | 427.681 | 32.567 | 404.753 | 32.533 | 516.201 |
| 32.286 | 472.624 | 31.751 | 593.023 | 34.040 | 746.047 | 32.760 | 429.617 | 32.679 | 406.249 | 32.644 | 519.719 |
| 32.399 | 475.268 | 31.864 | 595.907 | 34.151 | 749.163 | 32.872 | 432.177 | 32.791 | 408.808 | 32.755 | 522.362 |
| 32.512 | 477.966 | 31.977 | 598.660 | 34.261 | 752.699 | 32.984 | 434.246 | 32.903 | 410.898 | 32.866 | 525.669 |
| 32.624 | 480.178 | 32.090 | 601.554 | 34.372 | 754.715 | 33.096 | 436.119 | 33.015 | 413.300 | 32.977 | 528.290 |
| 32.737 | 482.931 | 32.204 | 605.022 | 34.483 | 759.816 | 33.207 | 437.933 | 33.127 | 415.431 | 33.088 | 530.408 |
| 32.849 | 485.801 | 32.317 | 609.341 | 34.593 | 762.489 | 33.319 | 439.608 | 33.238 | 418.157 | 33.200 | 532.739 |
| 32.962 | 487.825 | 32.430 | 612.992 | 34.704 | 765.567 | 33.431 | 441.757 | 33.350 | 419.639 | 33.311 | 534.887 |
| 33.074 | 491.342 | 32.544 | 616.339 | 34.815 | 769.445 | 33.543 | 443.901 | 33.462 | 421.681 | 33.422 | 536.929 |
| 33.187 | 493.578 | 32.657 | 619.505 | 34.925 | 772.695 | 33.655 | 446.207 | 33.574 | 423.823 | 33.533 | 540.148 |
| 33.300 | 495.911 | 32.770 | 623.337 | 35.036 | 775.564 | 33.767 | 449.175 | 33.686 | 425.938 | 33.644 | 542.554 |
| 33.412 | 498.732 | 32.884 | 625.789 | 35.147 | 780.201 | 33.879 | 451.519 | 33.798 | 427.451 | 33.755 | 545.461 |
| 33.525 | 500.041 | 32.997 | 627.987 | 35.258 | 784.341 | 33.991 | 454.278 | 33.910 | 428.785 | 33.866 | 546.925 |
| 33.637 | 503.162 | 33.110 | 633.442 | 35.368 | 788.697 | 34.103 | 456.645 | 34.022 | 431.252 | 33.978 | 548.984 |
| 33.750 | 506.204 | 33.224 | 636.981 | 35.479 | 790.127 | 34.215 | 459.196 | 34.134 | 433.690 | 34.089 | 551.370 |
| 33.862 | 509.353 | 33.337 | 639.781 | 35.590 | 794.669 | 34.327 | 461.678 | 34.246 | 436.520 | 34.200 | 553.706 |
| 33.975 | 510.440 | 33.450 | 642.348 | 35.700 | 798.579 | 34.439 | 464.507 | 34.358 | 437.912 | 34.311 | 556.097 |
| 34.088 | 513.461 | 33.564 | 646.397 | 35.811 | 801.431 | 34.551 | 466.709 | 34.470 | 439.683 | 34.422 | 559.233 |
| 34.200 | 517.394 | 33.677 | 649.339 | 35.922 | 805.977 | 34.663 | 469.109 | 34.582 | 442.554 | 34.533 | 561.720 |
| 34.313 | 520.256 | 33.790 | 651.410 | 36.032 | 806.930 | 34.775 | 471.847 | 34.694 | 444.841 | 34.644 | 564.960 |
| 34.425 | 523.188 | 33.904 | 654.420 | 36.143 | 810.741 | 34.887 | 474.023 | 34.806 | 445.789 | 34.755 | 567.311 |
| 34.538 | 525.490 | 34.017 | 657.147 | 36.254 | 814.988 | 34.999 | 475.868 | 34.918 | 447.912 | 34.867 | 569.760 |
| 34.650 | 527.044 | 34.130 | 660.749 | 36.364 | 818.766 | 35.111 | 478.535 | 35.030 | 449.641 | 34.978 | 573.805 |
| 34.763 | 529.173 | 34.244 | 666.269 | 36.475 | 821.713 | 35.223 | 480.279 | 35.141 | 452.503 | 35.089 | 576.437 |
| 34.876 | 532.579 | 34.357 | 669.267 | 36.586 | 824.265 | 35.335 | 482.588 | 35.253 | 455.586 | 35.200 | 579.001 |
| 34.988 | 535.601 | 34.470 | 672.385 | 36.697 | 827.216 | 35.447 | 483.516 | 35.365 | 457.244 | 35.311 | 581.104 |
| 35.101 | 536.868 | 34.584 | 675.884 | 36.807 | 831.267 | 35.559 | 486.178 | 35.477 | 459.185 | 35.422 | 583.704 |
| 35.213 | 538.882 | 34.697 | 678.613 | 36.918 | 834.220 | 35.671 | 488.551 | 35.589 | 461.438 | 35.533 | 586.430 |
| 35.326 | 541.430 | 34.810 | 680.642 | 37.029 | 837.777 | 35.783 | 491.677 | 35.701 | 464.017 | 35.645 | 588.821 |
| 35.438 | 544.797 | 34.924 | 684.113 | 37.139 | 841.049 | 35.895 | 493.189 | 35.813 | 466.597 | 35.756 | 591.787 |
| 35.551 | 545.769 | 35.037 | 688.420 | 37.250 | 843.073 | 36.007 | 496.087 | 35.925 | 468.485 | 35.867 | 594.217 |
| 35.664 | 549.042 | 35.150 | 693.108 | 37.361 | 846.044 | 36.119 | 497.485 | 36.037 | 470.437 | 35.978 | 596.742 |
| 35.776 | 551.827 | 35.263 | 696.629 | 37.471 | 849.960 | 36.231 | 499.916 | 36.149 | 472.398 | 36.089 | 599.282 |
| 35.889 | 553.488 | 35.377 | 699.130 | 37.582 | 855.052 | 36.343 | 501.930 | 36.261 | 473.281 | 36.200 | 601.179 |
| 36.001 | 555.018 | 35.490 | 701.680 | 37.693 | 857.576 | 36.455 | 504.114 | 36.373 | 475.860 | 36.311 | 604.081 |
| 36.114 | 557.181 | 35.603 | 705.162 | 37.804 | 860.195 | 36.567 | 506.410 | 36.485 | 478.093 | 36.422 | 606.474 |
| 36.226 | 559.205 | 35.717 | 708.982 | 37.914 | 862.855 | 36.679 | 508.641 | 36.597 | 481.007 | 36.534 | 608.969 |
| 36.339 | 562.341 | 35.830 | 710.930 | 38.025 | 865.548 | 36.791 | 510.525 | 36.709 | 483.773 | 36.645 | 611.768 |
| 36.452 | 564.854 | 35.943 | 713.529 | 38.136 | 867.689 | 36.903 | 512.329 | 36.821 | 485.987 | 36.756 | 614.798 |
| 36.564 | 566.503 | 36.057 | 716.784 | 38.246 | 870.686 | 37.015 | 514.705 | 36.932 | 488.473 | 36.867 | 616.804 |
| 36.677 | 568.461 | 36.170 | 720.588 | 38.357 | 874.754 | 37.127 | 517.380 | 37.044 | 490.591 | 36.978 | 618.956 |
| 36.789 | 571.361 | 36.283 | 724.893 | 38.468 | 880.261 | 37.239 | 520.365 | 37.156 | 492.620 | 37.089 | 621.576 |
| 36.902 | 574.549 | 36.397 | 728.782 | 38.578 | 885.364 | 37.351 | 523.871 | 37.268 | 495.219 | 37.200 | 624.698 |
| 37.014 | 575.918 | 36.510 | 732.711 | 38.689 | 889.199 | 37.463 | 526.205 | 37.380 | 496.884 | 37.312 | 627.677 |
| 37.127 | 578.887 | 36.623 | 736.029 | 38.800 | 889.319 | 37.575 | 528.203 | 37.492 | 499.702 | 37.423 | 629.706 |
| 37.240 | 581.700 | 36.737 | 739.562 | 38.910 | 893.220 | 37.687 | 530.226 | 37.604 | 501.969 | 37.534 | 632.023 |
| 37.352 | 583.691 | 36.850 | 740.902 | 39.021 | 897.271 | 37.799 | 532.292 | 37.716 | 504.398 | 37.645 | 635.189 |
| 37.465 | 586.009 | 36.963 | 744.894 | 39.132 | 898.373 | 37.911 | 534.608 | 37.828 | 506.803 | 37.756 | 637.175 |
| 37.577 | 589.553 | 37.077 | 748.251 | 39.243 | 906.020 | 38.023 | 537.086 | 37.940 | 508.544 | 37.867 | 641.153 |
| 37.690 | 592.948 | 37.190 | 751.997 | 39.353 | 908.944 | 38.135 | 539.862 | 38.052 | 510.429 | 37.978 | 643.710 |
| 37.802 | 596.013 | 37.303 | 756.194 | 39.464 | 912.370 | 38.246 | 541.903 | 38.164 | 512.269 | 38.089 | 646.346 |
| 37.915 | 598.618 | 37.417 | 758.816 | 39.575 | 914.977 | 38.358 | 544.619 | 38.276 | 514.036 | 38.201 | 648.951 |
| 38.028 | 600.682 | 37.530 | 761.527 | 39.685 | 919.417 | 38.470 | 546.640 | 38.388 | 516.753 | 38.312 | 651.192 |
| 38.140 | 603.205 | 37.643 | 765.634 | 39.796 | 922.909 | 38.582 | 549.592 | 38.500 | 518.989 | 38.423 | 653.657 |
| 38.253 | 606.966 | 37.757 | 769.513 | 39.907 | 927.069 | 38.694 | 552.768 | 38.612 | 521.056 | 38.534 | 656.082 |
| 38.365 | 609.314 | 37.870 | 771.053 | 40.017 | 931.609 | 38.806 | 555.517 | 38.723 | 523.108 | 38.645 | 659.482 |
| 38.478 | 611.202 | 37.983 | 774.653 | 40.128 | 933.392 | 38.918 | 557.582 | 38.835 | 525.368 | 38.756 | 662.140 |
| 38.590 | 615.098 | 38.097 | 777.876 | 40.239 | 936.808 | 39.030 | 560.168 | 38.947 | 527.489 | 38.867 | 664.508 |
| 38.703 | 617.443 | 38.210 | 781.928 | 40.350 | 940.707 | 39.142 | 562.831 | 39.059 | 529.110 | 38.978 | 667.472 |
| 38.815 | 618.967 | 38.323 | 784.829 | 40.460 | 944.499 | 39.254 | 566.084 | 39.171 | 531.706 | 39.090 | 670.424 |
| 38.928 | 622.484 | 38.437 | 786.627 | 40.571 | 949.986 | 39.366 | 568.096 | 39.283 | 534.522 | 39.201 | 673.112 |
| 39.041 | 625.915 | 38.550 | 788.751 | 40.682 | 953.781 | 39.478 | 571.141 | 39.395 | 536.824 | 39.312 | 674.997 |
| 39.153 | 629.489 | 38.663 | 792.447 | 40.792 | 954.949 | 39.590 | 573.417 | 39.507 | 540.027 | 39.423 | 677.809 |
| 39.266 | 631.908 | 38.776 | 795.686 | 40.903 | 956.665 | 39.702 | 575.492 | 39.619 | 541.779 | 39.534 | 681.341 |
| 39.378 | 633.438 | 38.890 | 798.752 | 41.014 | 962.404 | 39.814 | 577.276 | 39.731 | 543.504 | 39.645 | 685.050 |
| 39.491 | 636.560 | 39.003 | 801.904 | 41.124 | 966.824 | 39.926 | 579.093 | 39.843 | 545.335 | 39.756 | 687.435 |
| 39.603 | 639.697 | 39.116 | 805.362 | 41.235 | 970.650 | 40.038 | 581.638 | 39.955 | 546.955 | 39.868 | 690.108 |
| 39.716 | 642.919 | 39.230 | 808.839 | 41.346 | 975.075 | 40.150 | 584.157 | 40.067 | 549.094 | 39.979 | 693.130 |
| 39.829 | 647.478 | 39.343 | 810.328 | 41.456 | 978.340 | 40.262 | 586.616 | 40.179 | 551.742 | 40.090 | 695.794 |
| 39.941 | 650.902 | 39.456 | 814.333 | 41.567 | 981.545 | 40.374 | 589.155 | 40.291 | 554.510 | 40.201 | 698.188 |
| 40.054 | 652.698 | 39.570 | 818.197 | 41.678 | 984.151 | 40.486 | 591.481 | 40.403 | 557.615 | 40.312 | 700.623 |
| 40.166 | 655.637 | 39.683 | 821.118 | 41.789 | 987.795 | 40.598 | 593.984 | 40.514 | 559.581 | 40.423 | 703.614 |
| 40.279 | 657.981 | 39.796 | 825.988 | 41.899 | 991.086 | 40.710 | 596.060 | 40.626 | 561.765 | 40.534 | 706.708 |
| 40.391 | 663.947 | 39.910 | 830.160 | 42.010 | 994.505 | 40.822 | 597.959 | 40.738 | 564.068 | 40.645 | 710.395 |
| 40.504 | 666.568 | 40.023 | 833.863 | 42.121 | 998.009 | 40.934 | 600.152 | 40.850 | 566.755 | 40.757 | 713.589 |
| 40.617 | 668.617 | 40.136 | 837.187 | 42.231 | 1002.002 | 41.046 | 603.077 | 40.962 | 569.392 | 40.868 | 716.237 |
| 40.729 | 671.433 | 40.250 | 840.426 | 42.342 | 1005.029 | 41.158 | 605.234 | 41.074 | 571.936 | 40.979 | 719.367 |
| 40.842 | 674.339 | 40.363 | 842.279 | 42.453 | 1008.649 | 41.270 | 608.113 | 41.186 | 574.300 | 41.090 | 721.064 |
| 40.954 | 676.648 | 40.476 | 843.975 | 42.563 | 1012.397 | 41.382 | 609.945 | 41.298 | 576.175 | 41.201 | 724.222 |
| 41.067 | 677.303 | 40.590 | 847.037 | 42.674 | 1016.573 | 41.494 | 613.504 | 41.410 | 577.921 | 41.312 | 727.549 |
| 41.179 | 680.608 | 40.703 | 851.436 | 42.785 | 1019.516 | 41.606 | 617.201 | 41.522 | 579.860 | 41.423 | 729.737 |
| 41.292 | 684.707 | 40.816 | 856.363 | 42.896 | 1022.193 | 41.718 | 618.745 | 41.634 | 582.307 | 41.535 | 732.306 |
| 41.405 | 687.365 | 40.930 | 862.220 | 43.006 | 1026.459 | 41.830 | 620.661 | 41.746 | 584.800 | 41.646 | 735.805 |
| 41.517 | 691.265 | 41.043 | 863.389 | 43.117 | 1031.384 | 41.942 | 623.542 | 41.858 | 587.614 | 41.757 | 738.629 |
| 41.630 | 695.231 | 41.156 | 867.079 | 43.228 | 1034.875 | 42.054 | 627.230 | 41.970 | 589.512 | 41.868 | 743.278 |
| 41.742 | 696.842 | 41.270 | 869.492 | 43.338 | 1037.353 | 42.166 | 630.119 | 42.082 | 592.118 | 41.979 | 745.611 |
| 41.855 | 699.577 | 41.383 | 872.386 | 43.449 | 1041.576 | 42.278 | 632.306 | 42.194 | 594.927 | 42.090 | 745.807 |
| 41.967 | 702.173 | 41.496 | 877.640 | 43.560 | 1043.310 | 42.390 | 635.135 | 42.305 | 597.238 | 42.201 | 749.395 |
| 42.080 | 704.084 | 41.610 | 882.145 | 43.670 | 1047.830 | 42.502 | 637.447 | 42.417 | 599.878 | 42.312 | 752.604 |
| 42.193 | 707.822 | 41.723 | 885.742 | 43.781 | 1050.609 | 42.614 | 640.084 | 42.529 | 602.393 | 42.424 | 755.314 |
| 42.305 | 711.693 | 41.836 | 890.993 | 43.892 | 1053.497 | 42.726 | 642.968 | 42.641 | 603.893 | 42.535 | 757.015 |
| 42.418 | 713.994 | 41.949 | 894.163 | 44.002 | 1057.492 | 42.838 | 645.186 | 42.753 | 605.725 | 42.646 | 760.906 |
| 42.530 | 716.280 | 42.063 | 898.055 | 44.113 | 1060.983 | 42.950 | 647.497 | 42.865 | 608.499 | 42.757 | 764.549 |
| 42.643 | 719.409 | 42.176 | 902.362 | 44.224 | 1066.531 | 43.062 | 649.932 | 42.977 | 610.794 | 42.868 | 767.053 |
| 42.755 | 722.597 | 42.289 | 906.429 | 44.335 | 1069.915 | 43.174 | 652.447 | 43.089 | 614.132 | 42.979 | 769.609 |
| 42.868 | 725.976 | 42.403 | 911.199 | 44.445 | 1074.220 | 43.285 | 655.966 | 43.201 | 615.787 | 43.090 | 773.480 |
| 42.981 | 728.947 | 42.516 | 912.872 | 44.556 | 1079.156 | 43.397 | 658.514 | 43.313 | 617.176 | 43.202 | 777.329 |
| 43.093 | 730.676 | 42.629 | 918.227 | 44.667 | 1080.947 | 43.509 | 661.294 | 43.425 | 619.628 | 43.313 | 780.578 |
| 43.206 | 734.390 | 42.743 | 920.791 | 44.777 | 1086.695 | 43.621 | 662.331 | 43.537 | 622.252 | 43.424 | 783.832 |
| 43.318 | 738.096 | 42.856 | 924.195 | 44.888 | 1090.537 | 43.733 | 664.964 | 43.649 | 624.708 | 43.535 | 787.506 |
| 43.431 | 741.326 | 42.969 | 927.606 | 44.999 | 1095.622 | 43.845 | 667.465 | 43.761 | 626.886 | 43.646 | 789.414 |
| 43.543 | 743.709 | 43.083 | 931.912 | 45.109 | 1100.989 | 43.957 | 669.522 | 43.873 | 629.099 | 43.757 | 793.090 |
| 43.656 | 745.756 | 43.196 | 935.990 | 45.220 | 1104.511 | 44.069 | 672.567 | 43.985 | 631.185 | 43.868 | 796.017 |
| 43.769 | 746.975 | 43.309 | 938.894 | 45.331 | 1108.447 | 44.181 | 675.228 | 44.096 | 633.888 | 43.979 | 798.225 |
| 43.881 | 749.657 | 43.423 | 943.264 | 45.442 | 1110.802 | 44.293 | 677.417 | 44.208 | 635.801 | 44.091 | 800.291 |
| 43.994 | 752.734 | 43.536 | 946.833 | 45.552 | 1114.495 | 44.405 | 679.700 | 44.320 | 638.155 | 44.202 | 801.965 |
| 44.106 | 755.389 | 43.649 | 951.126 | 45.663 | 1118.813 | 44.517 | 682.233 | 44.432 | 640.904 | 44.313 | 804.455 |
| 44.219 | 759.529 | 43.763 | 955.791 | 45.774 | 1123.628 | 44.629 | 684.793 | 44.544 | 643.636 | 44.424 | 807.447 |
| 44.331 | 762.694 | 43.876 | 959.987 | 45.884 | 1127.747 | 44.741 | 687.430 | 44.656 | 646.128 | 44.535 | 810.213 |
| 44.444 | 765.739 | 43.989 | 962.245 | 45.995 | 1131.322 | 44.853 | 690.041 | 44.768 | 648.316 | 44.646 | 813.881 |
| 44.557 | 767.240 | 44.103 | 964.742 | 46.106 | 1136.599 | 44.965 | 692.207 | 44.880 | 650.903 | 44.757 | 816.608 |
| 44.669 | 767.992 | 44.216 | 968.402 | 46.216 | 1139.204 | 45.077 | 695.069 | 44.992 | 653.389 | 44.869 | 819.513 |
| 44.782 | 772.374 | 44.329 | 973.833 | 46.327 | 1143.781 | 45.189 | 697.758 | 45.104 | 656.230 | 44.980 | 822.392 |
| 44.894 | 775.239 | 44.443 | 979.434 | 46.438 | 1148.778 | 45.301 | 700.013 | 45.216 | 659.234 | 45.091 | 826.200 |
| 45.007 | 778.645 | 44.556 | 984.106 | 46.548 | 1151.477 | 45.413 | 702.840 | 45.328 | 661.258 | 45.202 | 828.614 |
| 45.119 | 780.915 | 44.669 | 988.359 | 46.659 | 1154.995 | 45.525 | 706.199 | 45.440 | 662.469 | 45.313 | 831.534 |
| 45.232 | 784.482 | 44.783 | 989.398 | 46.770 | 1159.466 | 45.637 | 708.344 | 45.552 | 664.959 | 45.424 | 835.157 |
| 45.345 | 786.609 | 44.896 | 992.449 | 46.881 | 1163.465 | 45.749 | 711.640 | 45.664 | 668.615 | 45.535 | 839.469 |
| 45.457 | 788.724 | 45.009 | 994.862 | 46.991 | 1166.669 | 45.861 | 714.651 | 45.776 | 670.665 | 45.646 | 842.269 |
| 45.570 | 790.457 | 45.122 | 997.668 | 47.102 | 1170.915 | 45.973 | 718.090 | 45.887 | 671.595 | 45.758 | 845.956 |
| 45.682 | 795.431 | 45.236 | 1000.872 | 47.213 | 1175.290 | 46.085 | 719.920 | 45.999 | 675.380 | 45.869 | 848.745 |
| 45.795 | 797.610 | 45.349 | 1005.784 | 47.323 | 1177.495 | 46.197 | 723.095 | 46.111 | 677.656 | 45.980 | 851.448 |
| 45.907 | 800.961 | 45.462 | 1009.333 | 47.434 | 1180.869 | 46.309 | 725.443 | 46.223 | 679.602 | 46.091 | 854.265 |
| 46.020 | 806.066 | 45.576 | 1013.913 | 47.545 | 1183.142 | 46.421 | 727.281 | 46.335 | 681.907 | 46.202 | 858.271 |
| 46.133 | 808.137 | 45.689 | 1018.952 | 47.655 | 1188.114 | 46.533 | 729.679 | 46.447 | 684.812 | 46.313 | 860.122 |
| 46.245 | 810.888 | 45.802 | 1021.985 | 47.766 | 1191.986 | 46.645 | 732.076 | 46.559 | 687.317 | 46.424 | 865.387 |
| 46.358 | 813.714 | 45.916 | 1026.226 | 47.877 | 1195.716 | 46.757 | 734.198 | 46.671 | 690.214 | 46.536 | 867.313 |
| 46.470 | 820.690 | 46.029 | 1031.524 | 47.988 | 1200.572 | 46.869 | 737.592 | 46.783 | 692.296 | 46.647 | 871.977 |
| 46.583 | 823.792 | 46.142 | 1034.257 | 48.098 | 1203.422 | 46.981 | 740.356 | 46.895 | 695.308 | 46.758 | 875.719 |
| 46.695 | 825.318 | 46.256 | 1037.979 | 48.209 | 1207.567 | 47.093 | 742.265 | 47.007 | 697.804 | 46.869 | 879.032 |
| 46.808 | 829.410 | 46.369 | 1042.070 | 48.320 | 1212.936 | 47.205 | 744.588 | 47.119 | 700.595 | 46.980 | 883.063 |
| 46.921 | 831.825 | 46.482 | 1046.703 | 48.430 | 1219.467 | 47.317 | 748.219 | 47.231 | 703.450 | 47.091 | 885.722 |
| 47.033 | 834.975 | 46.596 | 1050.587 | 48.541 | 1223.575 | 47.429 | 751.444 | 47.343 | 706.541 | 47.202 | 888.399 |
| 47.146 | 840.607 | 46.709 | 1055.232 | 48.652 | 1226.910 | 47.541 | 754.366 | 47.455 | 709.562 | 47.313 | 891.266 |
| 47.258 | 844.125 | 46.822 | 1056.779 | 48.762 | 1230.682 | 47.653 | 757.678 | 47.567 | 712.889 | 47.425 | 893.704 |
| 47.371 | 847.304 | 46.936 | 1059.275 | 48.873 | 1234.297 | 47.765 | 759.638 | 47.678 | 715.692 | 47.536 | 895.984 |
| 47.483 | 850.511 | 47.049 | 1064.201 | 48.984 | 1238.849 | 47.877 | 764.209 | 47.790 | 718.636 | 47.647 | 900.171 |
| 47.596 | 853.665 | 47.162 | 1070.848 | 49.094 | 1243.683 | 47.989 | 767.084 | 47.902 | 721.131 | 47.758 | 903.168 |
| 47.709 | 855.494 | 47.276 | 1074.974 | 49.205 | 1250.468 | 48.101 | 770.404 | 48.014 | 722.433 | 47.869 | 906.219 |
| 47.821 | 859.221 | 47.389 | 1078.581 | 49.316 | 1255.242 | 48.213 | 772.708 | 48.126 | 724.951 | 47.980 | 909.565 |
| 47.934 | 861.291 | 47.502 | 1081.957 | 49.427 | 1258.721 | 48.325 | 774.784 | 48.238 | 728.178 | 48.091 | 912.440 |
| 48.046 | 864.532 | 47.616 | 1084.305 | 49.537 | 1263.132 | 48.436 | 777.919 | 48.350 | 731.101 | 48.203 | 915.250 |
| 48.159 | 866.673 | 47.729 | 1086.734 | 49.648 | 1266.425 | 48.548 | 779.523 | 48.462 | 733.696 | 48.314 | 919.145 |
| 48.271 | 869.597 | 47.842 | 1091.675 | 49.759 | 1269.210 | 48.660 | 782.250 | 48.574 | 736.503 | 48.425 | 922.077 |
| 48.384 | 872.718 | 47.956 | 1093.897 | 49.869 | 1272.016 | 48.772 | 785.291 | 48.686 | 738.997 | 48.536 | 925.016 |
| 48.497 | 876.398 | 48.069 | 1099.151 | 49.980 | 1274.929 | 48.884 | 789.191 | 48.798 | 741.245 | 48.647 | 928.882 |
| 48.609 | 881.395 | 48.182 | 1101.159 | 50.091 | 1279.232 | 48.996 | 791.443 | 48.910 | 743.994 | 48.758 | 930.848 |
| 48.722 | 882.982 | 48.295 | 1104.178 | 50.201 | 1284.972 | 49.108 | 794.889 | 49.022 | 745.906 | 48.869 | 934.451 |
| 48.834 | 885.986 | 48.409 | 1108.795 | 50.312 | 1290.466 | 49.220 | 797.904 | 49.134 | 748.009 | 48.980 | 938.505 |
| 48.947 | 889.985 | 48.522 | 1111.836 | 50.423 | 1295.392 | 49.332 | 800.995 | 49.246 | 750.553 | 49.092 | 940.938 |
| 49.059 | 893.114 | 48.635 | 1115.346 | 50.534 | 1300.514 | 49.444 | 804.097 | 49.358 | 753.712 | 49.203 | 943.308 |
| 49.172 | 895.940 | 48.749 | 1119.110 | 50.644 | 1304.358 | 49.556 | 806.365 | 49.469 | 756.916 | 49.314 | 946.571 |
| 49.285 | 899.247 | 48.862 | 1122.574 | 50.755 | 1307.165 | 49.668 | 809.147 | 49.581 | 759.453 | 49.425 | 949.963 |
| 49.397 | 902.810 | 48.975 | 1126.331 | 50.866 | 1310.583 | 49.780 | 811.736 | 49.693 | 762.361 | 49.536 | 954.207 |
| 49.510 | 905.303 | 49.089 | 1129.832 | 50.976 | 1314.114 | 49.892 | 815.186 | 49.805 | 764.460 | 49.647 | 957.834 |
| 49.622 | 907.869 | 49.202 | 1132.984 | 51.087 | 1317.139 | 50.004 | 817.649 | 49.917 | 766.815 | 49.758 | 960.158 |
| 49.735 | 909.972 | 49.315 | 1134.601 | 51.198 | 1322.576 | 50.116 | 820.678 | 50.029 | 769.519 | 49.870 | 964.001 |
| 49.847 | 913.201 | 49.429 | 1139.508 | 51.308 | 1327.403 | 50.228 | 823.127 | 50.141 | 771.576 | 49.981 | 966.720 |
| 49.960 | 916.932 | 49.542 | 1142.727 | 51.419 | 1332.367 | 50.340 | 825.768 | 50.253 | 774.100 | 50.092 | 970.648 |
| 50.073 | 920.624 | 49.655 | 1147.871 | 51.530 | 1337.515 | 50.452 | 828.702 | 50.365 | 776.753 | 50.203 | 974.164 |
| 50.185 | 925.725 | 49.769 | 1151.842 | 51.641 | 1342.153 | 50.564 | 831.630 | 50.477 | 778.974 | 50.314 | 977.817 |
| 50.298 | 928.763 | 49.882 | 1155.134 | 51.751 | 1347.678 | 50.676 | 834.414 | 50.589 | 781.405 | 50.425 | 980.140 |
| 50.410 | 931.629 | 49.995 | 1160.741 | 51.862 | 1352.190 | 50.788 | 836.111 | 50.701 | 784.091 | 50.536 | 984.257 |
| 50.523 | 934.332 | 50.109 | 1164.502 | 51.973 | 1355.155 | 50.900 | 839.242 | 50.813 | 787.266 | 50.647 | 988.142 |
| 50.635 | 936.678 | 50.222 | 1165.462 | 52.083 | 1358.274 | 51.012 | 843.162 | 50.925 | 790.394 | 50.759 | 989.773 |
| 50.748 | 940.529 | 50.335 | 1171.312 | 52.194 | 1363.336 | 51.124 | 846.645 | 51.037 | 793.278 | 50.870 | 993.477 |
| 50.860 | 943.007 | 50.449 | 1175.536 | 52.305 | 1368.535 | 51.236 | 849.798 | 51.149 | 795.679 | 50.981 | 997.236 |
| 50.973 | 946.416 | 50.562 | 1179.705 | 52.415 | 1372.352 | 51.348 | 851.767 | 51.260 | 797.875 | 51.092 | 1000.167 |
| 51.086 | 952.220 | 50.675 | 1184.302 | 52.526 | 1375.744 | 51.460 | 854.631 | 51.372 | 800.991 | 51.203 | 1003.609 |
| 51.198 | 954.832 | 50.789 | 1188.035 | 52.637 | 1378.853 | 51.572 | 858.027 | 51.484 | 804.319 | 51.314 | 1006.627 |
| 51.311 | 956.739 | 50.902 | 1192.404 | 52.747 | 1382.836 | 51.684 | 860.894 | 51.596 | 807.288 | 51.425 | 1009.597 |
| 51.423 | 958.861 | 51.015 | 1194.371 | 52.858 | 1386.866 | 51.796 | 863.795 | 51.708 | 809.326 | 51.536 | 1013.432 |
| 51.536 | 961.908 | 51.129 | 1198.419 | 52.969 | 1389.554 | 51.908 | 866.820 | 51.820 | 811.644 | 51.648 | 1017.527 |
| 51.648 | 965.719 | 51.242 | 1205.027 | 53.080 | 1394.689 | 52.020 | 869.972 | 51.932 | 815.208 | 51.759 | 1021.397 |
| 51.761 | 969.576 | 51.355 | 1206.967 | 53.190 | 1399.224 | 52.132 | 873.794 | 52.044 | 818.144 | 51.870 | 1026.258 |
| 51.874 | 972.293 | 51.469 | 1212.437 | 53.301 | 1404.091 | 52.244 | 876.892 | 52.156 | 821.264 | 51.981 | 1029.791 |
| 51.986 | 976.470 | 51.582 | 1218.102 | 53.412 | 1408.978 | 52.356 | 879.588 | 52.268 | 824.048 | 52.092 | 1032.430 |
| 52.099 | 979.649 | 51.695 | 1222.089 | 53.522 | 1413.336 | 52.468 | 881.440 | 52.380 | 826.744 | 52.203 | 1035.060 |
| 52.211 | 981.858 | 51.808 | 1225.371 | 53.633 | 1418.160 | 52.580 | 884.594 | 52.492 | 829.178 | 52.314 | 1038.086 |
| 52.324 | 984.525 | 51.922 | 1228.990 | 53.744 | 1423.042 | 52.692 | 888.322 | 52.604 | 831.503 | 52.426 | 1041.735 |
| 52.436 | 989.109 | 52.035 | 1233.476 | 53.854 | 1426.146 | 52.804 | 892.032 | 52.716 | 834.264 | 52.537 | 1045.112 |
| 52.549 | 992.506 | 52.148 | 1238.515 | 53.965 | 1431.531 | 52.916 | 894.672 | 52.828 | 837.160 | 52.648 | 1048.381 |
| 52.662 | 998.593 | 52.262 | 1242.083 | 54.076 | 1435.922 | 53.028 | 896.343 | 52.940 | 839.547 | 52.759 | 1051.542 |
| 52.774 | 999.993 | 52.375 | 1247.329 | 54.187 | 1437.616 | 53.140 | 898.709 | 53.051 | 841.729 | 52.870 | 1054.732 |
| 52.887 | 1001.791 | 52.488 | 1253.490 | 54.297 | 1441.206 | 53.252 | 901.741 | 53.163 | 843.930 | 52.981 | 1057.618 |
| 52.999 | 1003.615 | 52.602 | 1254.350 | 54.408 | 1446.877 | 53.364 | 904.414 | 53.275 | 846.737 | 53.092 | 1060.575 |
| 53.112 | 1008.176 | 52.715 | 1256.694 | 54.519 | 1452.878 | 53.475 | 907.834 | 53.387 | 849.309 | 53.203 | 1062.364 |
| 53.224 | 1012.430 | 52.828 | 1261.521 | 54.629 | 1456.687 | 53.587 | 910.862 | 53.499 | 851.742 | 53.315 | 1067.209 |
| 53.337 | 1014.996 | 52.942 | 1267.008 | 54.740 | 1458.734 | 53.699 | 913.577 | 53.611 | 854.499 | 53.426 | 1071.710 |
| 53.450 | 1020.005 | 53.055 | 1270.467 | 54.851 | 1461.861 | 53.811 | 916.860 | 53.723 | 858.388 | 53.537 | 1074.001 |
| 53.562 | 1024.630 | 53.168 | 1277.184 | 54.961 | 1466.365 | 53.923 | 919.379 | 53.835 | 861.318 | 53.648 | 1078.100 |
| 53.675 | 1027.464 | 53.282 | 1281.353 | 55.072 | 1470.455 | 54.035 | 922.523 | 53.947 | 864.084 | 53.759 | 1081.062 |
| 53.787 | 1030.607 | 53.395 | 1285.076 | 55.183 | 1476.143 | 54.147 | 924.888 | 54.059 | 866.333 | 53.870 | 1085.086 |
| 53.900 | 1033.363 | 53.508 | 1289.009 | 55.293 | 1482.813 | 54.259 | 927.521 | 54.171 | 869.687 | 53.981 | 1088.402 |
| 54.012 | 1036.738 | 53.622 | 1290.576 | 55.404 | 1487.776 | 54.371 | 929.898 | 54.283 | 871.517 | 54.093 | 1090.821 |
| 54.125 | 1038.738 | 53.735 | 1296.072 | 55.515 | 1491.946 | 54.483 | 932.513 | 54.395 | 874.876 | 54.204 | 1093.203 |
| 54.238 | 1042.481 | 53.848 | 1303.520 | 55.626 | 1497.088 | 54.595 | 935.630 | 54.507 | 877.986 | 54.315 | 1096.107 |
| 54.350 | 1046.094 | 53.962 | 1309.630 | 55.736 | 1501.165 | 54.707 | 939.215 | 54.619 | 880.872 | 54.426 | 1098.494 |
| 54.463 | 1050.464 | 54.075 | 1313.105 | 55.847 | 1505.176 | 54.819 | 941.834 | 54.731 | 883.244 | 54.537 | 1102.351 |
| 54.575 | 1053.624 | 54.188 | 1317.356 | 55.958 | 1510.934 | 54.931 | 944.353 | 54.842 | 886.493 | 54.648 | 1105.387 |
| 54.688 | 1056.874 | 54.302 | 1322.956 | 56.068 | 1519.645 | 55.043 | 947.210 | 54.954 | 889.618 | 54.759 | 1108.737 |
| 54.800 | 1060.533 | 54.415 | 1330.500 | 56.179 | 1522.264 | 55.155 | 950.028 | 55.066 | 892.513 | 54.870 | 1111.806 |
| 54.913 | 1064.576 | 54.528 | 1331.656 | 56.290 | 1525.506 | 55.267 | 952.885 | 55.178 | 895.638 | 54.982 | 1114.944 |
| 55.026 | 1066.525 | 54.642 | 1333.800 | 56.400 | 1529.519 | 55.379 | 955.141 | 55.290 | 898.615 | 55.093 | 1118.454 |
| 55.138 | 1069.094 | 54.755 | 1342.656 | 56.511 | 1534.970 | 55.491 | 958.719 | 55.402 | 899.970 | 55.204 | 1122.978 |
| 55.251 | 1072.938 | 54.868 | 1344.877 | 56.622 | 1539.810 | 55.603 | 962.774 | 55.514 | 902.418 | 55.315 | 1125.919 |
| 55.363 | 1078.785 | 54.981 | 1351.017 | 56.733 | 1544.219 | 55.715 | 966.082 | 55.626 | 905.771 | 55.426 | 1129.144 |
| 55.476 | 1082.235 | 55.095 | 1352.183 | 56.843 | 1548.101 | 55.827 | 969.201 | 55.738 | 909.618 | 55.537 | 1131.925 |
| 55.588 | 1086.570 | 55.208 | 1355.910 | 56.954 | 1552.800 | 55.939 | 972.624 | 55.850 | 912.665 | 55.648 | 1135.827 |
| 55.701 | 1088.225 | 55.321 | 1366.477 | 57.065 | 1554.909 | 56.051 | 975.925 | 55.962 | 915.354 | 55.760 | 1138.760 |
| 55.814 | 1091.734 | 55.435 | 1368.826 | 57.175 | 1560.148 | 56.163 | 977.886 | 56.074 | 917.662 | 55.871 | 1142.913 |
| 55.926 | 1093.844 | 55.548 | 1372.511 | 57.286 | 1564.923 | 56.275 | 980.552 | 56.186 | 920.667 | 55.982 | 1146.661 |
| 56.039 | 1097.243 | 55.661 | 1377.199 | 57.397 | 1568.392 | 56.387 | 983.829 | 56.298 | 923.870 | 56.093 | 1150.536 |
| 56.151 | 1101.183 | 55.775 | 1380.855 | 57.507 | 1571.988 | 56.499 | 987.613 | 56.410 | 927.288 | 56.204 | 1154.039 |
| 56.264 | 1107.073 | 55.888 | 1387.929 | 57.618 | 1577.804 | 56.611 | 990.012 | 56.522 | 930.963 | 56.315 | 1158.341 |
| 56.376 | 1110.215 | 56.001 | 1391.675 | 57.729 | 1581.483 | 56.723 | 992.631 | 56.633 | 933.518 | 56.426 | 1160.273 |
| 56.489 | 1109.776 | 56.115 | 1398.038 | 57.839 | 1588.369 | 56.835 | 995.639 | 56.745 | 935.825 | 56.537 | 1164.471 |
| 56.602 | 1112.219 | 56.228 | 1403.497 | 57.950 | 1593.838 | 56.947 | 999.177 | 56.857 | 938.512 | 56.649 | 1169.123 |
| 56.714 | 1116.382 | 56.341 | 1409.383 | 58.061 | 1596.474 | 57.059 | 1002.922 | 56.969 | 941.459 | 56.760 | 1173.102 |
| 56.827 | 1119.374 | 56.455 | 1411.453 | 58.172 | 1602.279 | 57.171 | 1005.854 | 57.081 | 944.936 | 56.871 | 1178.886 |
| 56.939 | 1124.088 | 56.568 | 1415.452 | 58.282 | 1607.059 | 57.283 | 1008.658 | 57.193 | 947.967 | 56.982 | 1181.238 |
| 57.052 | 1127.387 | 56.681 | 1423.982 | 58.393 | 1612.159 | 57.395 | 1011.394 | 57.305 | 950.747 | 57.093 | 1183.541 |
| 57.164 | 1132.189 | 56.795 | 1426.332 | 58.504 | 1616.344 | 57.507 | 1014.269 | 57.417 | 953.877 | 57.204 | 1187.076 |
| 57.277 | 1136.061 | 56.908 | 1428.309 | 58.614 | 1620.979 | 57.619 | 1017.027 | 57.529 | 957.873 | 57.315 | 1191.442 |
| 57.390 | 1139.336 | 57.021 | 1434.321 | 58.725 | 1623.376 | 57.731 | 1021.032 | 57.641 | 961.388 | 57.427 | 1197.384 |
| 57.502 | 1143.331 | 57.135 | 1440.786 | 58.836 | 1627.454 | 57.843 | 1024.965 | 57.753 | 963.130 | 57.538 | 1201.963 |
| 57.615 | 1147.842 | 57.248 | 1444.060 | 58.946 | 1636.923 | 57.955 | 1029.193 | 57.865 | 965.678 | 57.649 | 1205.721 |
| 57.727 | 1150.138 | 57.361 | 1447.808 | 59.057 | 1641.137 | 58.067 | 1031.608 | 57.977 | 968.627 | 57.760 | 1207.295 |
| 57.840 | 1153.110 | 57.475 | 1451.648 | 59.168 | 1645.069 | 58.179 | 1034.398 | 58.089 | 971.721 | 57.871 | 1210.141 |
| 57.952 | 1158.498 | 57.588 | 1456.329 | 59.279 | 1651.923 | 58.291 | 1037.540 | 58.201 | 974.508 | 57.982 | 1214.594 |
| 58.065 | 1163.486 | 57.701 | 1464.578 | 59.389 | 1654.480 | 58.403 | 1040.826 | 58.313 | 977.665 | 58.093 | 1218.421 |
| 58.178 | 1168.445 | 57.815 | 1467.604 | 59.500 | 1657.673 | 58.514 | 1043.983 | 58.424 | 981.360 | 58.204 | 1224.251 |
| 58.290 | 1170.976 | 57.928 | 1470.772 | 59.611 | 1664.607 | 58.626 | 1047.822 | 58.536 | 984.758 | 58.316 | 1227.231 |
| 58.403 | 1175.680 | 58.041 | 1472.488 | 59.721 | 1671.508 | 58.738 | 1051.978 | 58.648 | 986.714 | 58.427 | 1230.959 |
| 58.515 | 1180.259 | 58.154 | 1481.117 | 59.832 | 1677.924 | 58.850 | 1054.379 | 58.760 | 989.613 | 58.538 | 1234.671 |
| 58.628 | 1181.153 | 58.268 | 1485.991 | 59.943 | 1680.688 | 58.962 | 1056.442 | 58.872 | 992.700 | 58.649 | 1238.392 |
| 58.740 | 1184.511 | 58.381 | 1489.723 | 60.053 | 1683.853 | 59.074 | 1060.363 | 58.984 | 995.555 | 58.760 | 1241.092 |
| 58.853 | 1188.264 | 58.494 | 1494.911 | 60.164 | 1689.700 | 59.186 | 1064.010 | 59.096 | 998.904 | 58.871 | 1246.411 |
| 58.966 | 1194.116 | 58.608 | 1499.835 | 60.275 | 1692.146 | 59.298 | 1067.683 | 59.208 | 1002.347 | 58.982 | 1249.294 |
| 59.078 | 1199.274 | 58.721 | 1504.769 | 60.385 | 1696.055 | 59.410 | 1070.957 | 59.320 | 1005.928 | 59.094 | 1252.489 |
| 59.191 | 1202.196 | 58.834 | 1509.441 | 60.496 | 1702.626 | 59.522 | 1073.883 | 59.432 | 1007.695 | 59.205 | 1255.705 |
| 59.303 | 1207.703 | 58.948 | 1514.017 | 60.607 | 1708.469 | 59.634 | 1077.577 | 59.544 | 1010.856 | 59.316 | 1259.968 |
| 59.416 | 1211.673 | 59.061 | 1518.473 | 60.718 | 1712.689 | 59.746 | 1080.031 | 59.656 | 1013.427 | 59.427 | 1264.941 |
| 59.528 | 1213.818 | 59.174 | 1523.930 | 60.828 | 1716.975 | 59.858 | 1083.066 | 59.768 | 1016.313 | 59.538 | 1271.043 |
| 59.641 | 1217.392 | 59.288 | 1528.100 | 60.939 | 1721.236 | 59.970 | 1086.656 | 59.880 | 1018.978 | 59.649 | 1275.628 |
| 59.754 | 1221.436 | 59.401 | 1532.344 | 61.050 | 1725.916 | 60.082 | 1090.209 | 59.992 | 1022.620 | 59.760 | 1281.789 |
| 59.866 | 1225.833 | 59.514 | 1536.035 | 61.160 | 1729.647 | 60.194 | 1092.331 | 60.104 | 1025.644 | 59.871 | 1285.707 |
| 59.979 | 1231.160 | 59.628 | 1539.875 | 61.271 | 1733.359 | 60.306 | 1096.152 | 60.215 | 1029.322 | 59.983 | 1288.208 |
| 60.091 | 1234.505 | 59.741 | 1545.644 | 61.382 | 1736.281 | 60.418 | 1099.876 | 60.327 | 1032.842 | 60.094 | 1291.827 |
| 60.204 | 1236.874 | 59.854 | 1550.557 | 61.492 | 1740.773 | 60.530 | 1103.146 | 60.439 | 1035.741 | 60.205 | 1295.130 |
| 60.316 | 1239.538 | 59.968 | 1553.943 | 61.603 | 1745.424 | 60.642 | 1107.198 | 60.551 | 1038.741 | 60.316 | 1297.825 |
| 60.429 | 1245.568 | 60.081 | 1556.517 | 61.714 | 1750.489 | 60.754 | 1110.529 | 60.663 | 1041.673 | 60.427 | 1300.592 |
| 60.542 | 1250.967 | 60.194 | 1561.681 | 61.825 | 1756.010 | 60.866 | 1114.228 | 60.775 | 1044.539 | 60.538 | 1304.427 |
| 60.654 | 1256.690 | 60.308 | 1565.883 | 61.935 | 1761.747 | 60.978 | 1116.967 | 60.887 | 1046.294 | 60.649 | 1308.139 |
| 60.767 | 1258.341 | 60.421 | 1569.384 | 62.046 | 1766.739 | 61.090 | 1121.408 | 60.999 | 1049.476 | 60.761 | 1312.570 |
| 60.879 | 1261.584 | 60.534 | 1573.660 | 62.157 | 1771.067 | 61.202 | 1125.120 | 61.111 | 1052.930 | 60.872 | 1316.122 |
| 60.992 | 1265.300 | 60.648 | 1578.272 | 62.267 | 1775.830 | 61.314 | 1127.607 | 61.223 | 1056.541 | 60.983 | 1319.168 |
| 61.104 | 1266.815 | 60.761 | 1584.238 | 62.378 | 1780.206 | 61.426 | 1130.696 | 61.335 | 1058.781 | 61.094 | 1322.586 |
| 61.217 | 1269.519 | 60.874 | 1588.850 | 62.489 | 1784.448 | 61.538 | 1133.339 | 61.447 | 1062.056 | 61.205 | 1327.923 |
| 61.330 | 1276.439 | 60.988 | 1594.057 | 62.599 | 1787.165 | 61.650 | 1137.217 | 61.559 | 1066.209 | 61.316 | 1331.289 |
| 61.442 | 1280.256 | 61.101 | 1600.118 | 62.710 | 1793.007 | 61.762 | 1140.398 | 61.671 | 1069.378 | 61.427 | 1335.042 |
| 61.555 | 1284.354 | 61.214 | 1603.829 | 62.821 | 1797.256 | 61.874 | 1142.951 | 61.783 | 1071.447 | 61.538 | 1338.735 |
| 61.667 | 1291.257 | 61.327 | 1609.522 | 62.931 | 1801.329 | 61.986 | 1144.796 | 61.895 | 1074.573 | 61.650 | 1341.684 |
| 61.780 | 1294.672 | 61.441 | 1614.932 | 63.042 | 1806.005 | 62.098 | 1149.122 | 62.006 | 1078.153 | 61.761 | 1346.728 |
| 61.892 | 1297.001 | 61.554 | 1622.241 | 63.153 | 1808.795 | 62.210 | 1152.563 | 62.118 | 1081.516 | 61.872 | 1351.350 |
| 62.005 | 1299.630 | 61.667 | 1627.384 | 63.264 | 1816.358 | 62.322 | 1156.023 | 62.230 | 1083.959 | 61.983 | 1354.548 |
| 62.118 | 1301.917 | 61.781 | 1631.809 | 63.374 | 1819.522 | 62.434 | 1159.579 | 62.342 | 1086.279 | 62.094 | 1357.946 |
| 62.230 | 1306.095 | 61.894 | 1637.557 | 63.485 | 1824.956 | 62.546 | 1162.736 | 62.454 | 1088.864 | 62.205 | 1363.280 |
| 62.343 | 1311.950 | 62.007 | 1642.076 | 63.596 | 1829.720 | 62.658 | 1165.202 | 62.566 | 1092.799 | 62.316 | 1367.197 |
| 62.455 | 1315.023 | 62.121 | 1646.766 | 63.706 | 1836.061 | 62.770 | 1167.563 | 62.678 | 1096.062 | 62.427 | 1372.220 |
| 62.568 | 1319.845 | 62.234 | 1651.530 | 63.817 | 1841.478 | 62.882 | 1170.399 | 62.790 | 1098.494 | 62.539 | 1375.475 |
| 62.680 | 1323.930 | 62.347 | 1655.130 | 63.928 | 1846.777 | 62.994 | 1173.095 | 62.902 | 1101.553 | 62.650 | 1378.633 |
| 62.793 | 1326.682 | 62.461 | 1659.432 | 64.038 | 1852.839 | 63.106 | 1176.311 | 63.014 | 1104.999 | 62.761 | 1381.837 |
| 62.905 | 1331.176 | 62.574 | 1664.968 | 64.149 | 1860.054 | 63.218 | 1179.388 | 63.126 | 1107.469 | 62.872 | 1386.514 |
| 63.018 | 1334.606 | 62.687 | 1668.278 | 64.260 | 1863.683 | 63.330 | 1182.556 | 63.238 | 1110.859 | 62.983 | 1390.088 |
| 63.131 | 1339.021 | 62.801 | 1674.101 | 64.371 | 1868.485 | 63.442 | 1186.336 | 63.350 | 1113.336 | 63.094 | 1393.514 |
| 63.243 | 1343.148 | 62.914 | 1677.626 | 64.481 | 1873.854 | 63.553 | 1189.643 | 63.462 | 1115.924 | 63.205 | 1397.396 |
| 63.356 | 1346.977 | 63.027 | 1682.604 | 64.592 | 1878.298 | 63.665 | 1193.381 | 63.574 | 1118.824 | 63.317 | 1401.355 |
| 63.468 | 1350.895 | 63.141 | 1688.226 | 64.703 | 1883.768 | 63.777 | 1196.038 | 63.686 | 1121.757 | 63.428 | 1406.010 |
| 63.581 | 1354.643 | 63.254 | 1693.915 | 64.813 | 1891.140 | 63.889 | 1199.422 | 63.797 | 1126.186 | 63.539 | 1409.257 |
| 63.693 | 1357.852 | 63.367 | 1699.732 | 64.924 | 1896.281 | 64.001 | 1203.565 | 63.909 | 1129.365 | 63.650 | 1412.608 |
| 63.806 | 1362.655 | 63.481 | 1706.833 | 65.035 | 1898.569 | 64.113 | 1205.835 | 64.021 | 1132.519 | 63.761 | 1416.513 |
| 63.919 | 1366.521 | 63.594 | 1712.294 | 65.145 | 1901.712 | 64.225 | 1208.816 | 64.133 | 1135.740 | 63.872 | 1420.904 |
| 64.031 | 1369.608 | 63.707 | 1715.009 | 65.256 | 1905.541 | 64.337 | 1213.330 | 64.245 | 1138.643 | 63.983 | 1425.498 |
| 64.144 | 1373.343 | 63.821 | 1719.364 | 65.367 | 1912.108 | 64.449 | 1216.360 | 64.357 | 1141.841 | 64.094 | 1429.180 |
| 64.256 | 1376.864 | 63.934 | 1726.003 | 65.477 | 1917.878 | 64.561 | 1220.289 | 64.469 | 1145.117 | 64.206 | 1431.592 |
| 64.369 | 1380.530 | 64.047 | 1731.138 | 65.588 | 1922.676 | 64.673 | 1223.153 | 64.581 | 1148.984 | 64.317 | 1437.219 |
| 64.481 | 1384.530 | 64.161 | 1734.084 | 65.699 | 1928.297 | 64.785 | 1226.281 | 64.693 | 1151.609 | 64.428 | 1440.459 |
| 64.594 | 1388.244 | 64.274 | 1739.607 | 65.810 | 1934.128 | 64.897 | 1230.161 | 64.805 | 1154.121 | 64.539 | 1444.670 |
| 64.707 | 1391.761 | 64.387 | 1745.443 | 65.920 | 1940.775 | 65.009 | 1232.964 | 64.917 | 1156.931 | 64.650 | 1448.547 |
| 64.819 | 1394.899 | 64.501 | 1752.202 | 66.031 | 1945.980 | 65.121 | 1235.369 | 65.029 | 1159.256 | 64.761 | 1453.517 |
| 64.932 | 1399.596 | 64.614 | 1757.924 | 66.142 | 1950.671 | 65.233 | 1237.624 | 65.141 | 1161.906 | 64.872 | 1457.431 |
| 65.044 | 1400.089 | 64.727 | 1763.441 | 66.252 | 1954.778 | 65.345 | 1241.990 | 65.253 | 1165.692 | 64.984 | 1460.766 |
| 65.157 | 1404.651 | 64.840 | 1767.475 | 66.363 | 1958.496 | 65.457 | 1245.372 | 65.365 | 1169.011 | 65.095 | 1464.179 |
| 65.269 | 1409.835 | 64.954 | 1774.161 | 66.474 | 1962.899 | 65.569 | 1248.958 | 65.477 | 1173.052 | 65.206 | 1468.148 |
| 65.382 | 1415.086 | 65.067 | 1780.298 | 66.584 | 1968.022 | 65.681 | 1252.885 | 65.588 | 1176.504 | 65.317 | 1473.200 |
| 65.495 | 1418.914 | 65.180 | 1785.442 | 66.695 | 1973.551 | 65.793 | 1257.069 | 65.700 | 1179.277 | 65.428 | 1477.412 |
| 65.607 | 1424.216 | 65.294 | 1790.741 | 66.806 | 1981.451 | 65.905 | 1260.381 | 65.812 | 1182.996 | 65.539 | 1481.272 |
| 65.720 | 1428.353 | 65.407 | 1795.413 | 66.917 | 1986.966 | 66.017 | 1264.114 | 65.924 | 1186.254 | 65.650 | 1485.817 |
| 65.832 | 1433.497 | 65.520 | 1799.357 | 67.027 | 1989.807 | 66.129 | 1266.620 | 66.036 | 1188.766 | 65.761 | 1490.417 |
| 65.945 | 1437.004 | 65.634 | 1805.546 | 67.138 | 1994.449 | 66.241 | 1269.751 | 66.148 | 1192.617 | 65.873 | 1494.635 |
| 66.057 | 1440.115 | 65.747 | 1809.379 | 67.249 | 2001.262 | 66.353 | 1272.993 | 66.260 | 1195.509 | 65.984 | 1498.529 |
| 66.170 | 1441.176 | 65.860 | 1814.242 | 67.359 | 2006.384 | 66.465 | 1276.174 | 66.372 | 1197.795 | 66.095 | 1502.317 |
| 66.283 | 1445.022 | 65.974 | 1817.765 | 67.470 | 2008.691 | 66.577 | 1280.565 | 66.484 | 1200.485 | 66.206 | 1504.780 |
| 66.395 | 1448.673 | 66.087 | 1821.796 | 67.581 | 2022.922 | 66.689 | 1284.408 | 66.596 | 1204.788 | 66.317 | 1509.381 |
| 66.508 | 1453.920 | 66.200 | 1827.300 | 67.691 | 2019.446 | 66.801 | 1285.470 | 66.708 | 1207.702 | 66.428 | 1513.739 |
| 66.620 | 1458.979 | 66.314 | 1832.606 | 67.802 | 2024.652 | 66.913 | 1290.301 | 66.820 | 1210.801 | 66.539 | 1518.459 |
| 66.733 | 1460.939 | 66.427 | 1836.885 | 67.913 | 2030.692 | 67.025 | 1294.224 | 66.932 | 1213.626 | 66.651 | 1521.893 |
| 66.845 | 1463.758 | 66.540 | 1840.700 | 68.023 | 2043.190 | 67.137 | 1296.933 | 67.044 | 1217.093 | 66.762 | 1526.564 |
| 66.958 | 1467.836 | 66.654 | 1846.286 | 68.134 | 2046.727 | 67.249 | 1299.936 | 67.156 | 1220.320 | 66.873 | 1530.465 |
| 67.071 | 1471.407 | 66.767 | 1852.496 | 68.245 | 2048.991 | 67.361 | 1304.241 | 67.268 | 1223.894 | 66.984 | 1533.873 |
| 67.183 | 1475.080 | 66.880 | 1856.155 | 68.356 | 2054.161 | 67.473 | 1307.053 | 67.379 | 1226.998 | 67.095 | 1537.358 |
| 67.296 | 1479.069 | 66.994 | 1861.739 | 68.466 | 2061.119 | 67.585 | 1310.565 | 67.491 | 1230.241 | 67.206 | 1540.995 |
| 67.408 | 1482.318 | 67.107 | 1866.972 | 68.577 | 2069.915 | 67.697 | 1313.943 | 67.603 | 1233.066 | 67.317 | 1545.048 |
| 67.521 | 1486.657 | 67.220 | 1868.803 | 68.688 | 2080.416 | 67.809 | 1318.197 | 67.715 | 1235.445 | 67.428 | 1548.710 |
| 67.633 | 1490.974 | 67.334 | 1871.200 | 68.798 | 2086.827 | 67.921 | 1321.545 | 67.827 | 1239.096 | 67.540 | 1552.135 |
| 67.746 | 1494.876 | 67.447 | 1878.446 | 68.909 | 2091.273 | 68.033 | 1325.519 | 67.939 | 1244.369 | 67.651 | 1558.401 |
| 67.859 | 1498.635 | 67.560 | 1883.362 | 69.020 | 2096.266 | 68.145 | 1329.393 | 68.051 | 1248.065 | 67.762 | 1563.659 |
| 67.971 | 1504.119 | 67.674 | 1892.411 | 69.130 | 2097.293 | 68.257 | 1332.757 | 68.163 | 1252.067 | 67.873 | 1567.277 |
| 68.084 | 1508.428 | 67.787 | 1895.976 | 69.241 | 2101.208 | 68.369 | 1335.962 | 68.275 | 1254.983 | 67.984 | 1570.243 |
| 68.196 | 1513.814 | 67.900 | 1901.171 | 69.352 | 2110.180 | 68.481 | 1339.840 | 68.387 | 1257.570 | 68.095 | 1572.506 |
| 68.309 | 1518.149 | 68.013 | 1902.992 | 69.463 | 2115.241 | 68.592 | 1343.388 | 68.499 | 1260.515 | 68.206 | 1576.314 |
| 68.421 | 1523.671 | 68.127 | 1910.187 | 69.573 | 2120.036 | 68.704 | 1346.759 | 68.611 | 1263.961 | 68.318 | 1581.143 |
| 68.534 | 1527.746 | 68.240 | 1919.806 | 69.684 | 2124.792 | 68.816 | 1350.705 | 68.723 | 1267.439 | 68.429 | 1587.481 |
| 68.647 | 1531.778 | 68.353 | 1922.880 | 69.795 | 2130.312 | 68.928 | 1354.897 | 68.835 | 1270.670 | 68.540 | 1591.431 |
| 68.759 | 1537.145 | 68.467 | 1929.221 | 69.905 | 2135.434 | 69.040 | 1357.736 | 68.947 | 1274.533 | 68.651 | 1595.844 |
| 68.872 | 1541.653 | 68.580 | 1934.603 | 70.016 | 2140.140 | 69.152 | 1360.961 | 69.059 | 1277.841 | 68.762 | 1598.937 |
| 68.984 | 1547.098 | 68.693 | 1939.862 | 70.127 | 2146.581 | 69.264 | 1365.284 | 69.170 | 1281.047 | 68.873 | 1602.755 |
| 69.097 | 1548.019 | 68.807 | 1945.260 | 70.237 | 2153.942 | 69.376 | 1369.254 | 69.282 | 1284.462 | 68.984 | 1604.456 |
| 69.209 | 1551.460 | 68.920 | 1950.925 | 70.348 | 2159.005 | 69.488 | 1372.640 | 69.394 | 1288.124 | 69.095 | 1608.335 |
| 69.322 | 1555.904 | 69.033 | 1957.211 | 70.459 | 2164.235 | 69.600 | 1374.959 | 69.506 | 1292.769 | 69.207 | 1612.630 |
| 69.435 | 1559.509 | 69.147 | 1963.735 | 70.570 | 2170.166 | 69.712 | 1378.499 | 69.618 | 1295.874 | 69.318 | 1617.082 |
| 69.547 | 1564.392 | 69.260 | 1970.855 | 70.680 | 2175.374 | 69.824 | 1381.258 | 69.730 | 1298.640 | 69.429 | 1621.706 |
| 69.660 | 1571.203 | 69.373 | 1977.721 | 70.791 | 2180.282 | 69.936 | 1384.828 | 69.842 | 1302.579 | 69.540 | 1626.812 |
| 69.772 | 1574.939 | 69.487 | 1981.561 | 70.902 | 2186.305 | 70.048 | 1388.107 | 69.954 | 1306.031 | 69.651 | 1631.565 |
| 69.885 | 1578.009 | 69.600 | 1990.539 | 71.012 | 2191.400 | 70.160 | 1391.795 | 70.066 | 1308.384 | 69.762 | 1635.209 |
| 69.997 | 1582.838 | 69.713 | 2000.316 | 71.123 | 2198.291 | 70.272 | 1395.369 | 70.178 | 1312.550 | 69.873 | 1639.387 |
| 70.110 | 1587.330 | 69.827 | 2003.145 | 71.234 | 2201.759 | 70.384 | 1398.392 | 70.290 | 1316.403 | 69.985 | 1643.489 |
| 70.223 | 1591.765 | 69.940 | 2008.890 | 71.344 | 2206.700 | 70.496 | 1402.523 | 70.402 | 1319.706 | 70.096 | 1647.157 |
| 70.335 | 1594.909 | 70.053 | 2013.777 | 71.455 | 2209.665 | 70.608 | 1406.324 | 70.514 | 1323.452 | 70.207 | 1650.367 |
| 70.448 | 1599.876 | 70.167 | 2019.461 | 71.566 | 2215.400 | 70.720 | 1409.914 | 70.626 | 1327.557 | 70.318 | 1654.117 |
| 70.560 | 1603.263 | 70.280 | 2024.033 | 71.676 | 2224.435 | 70.832 | 1413.452 | 70.738 | 1330.915 | 70.429 | 1659.275 |
| 70.673 | 1608.598 | 70.393 | 2030.126 | 71.787 | 2230.539 | 70.944 | 1416.749 | 70.850 | 1333.809 | 70.540 | 1662.613 |
| 70.785 | 1613.811 | 70.507 | 2036.983 | 71.898 | 2235.323 | 71.056 | 1419.889 | 70.961 | 1337.057 | 70.651 | 1667.899 |
| 70.898 | 1617.949 | 70.620 | 2037.072 | 72.009 | 2240.683 | 71.168 | 1423.944 | 71.073 | 1340.520 | 70.762 | 1672.106 |
| 71.011 | 1621.703 | 70.733 | 2039.975 | 72.119 | 2247.028 | 71.280 | 1427.189 | 71.185 | 1343.996 | 70.874 | 1679.087 |
| 71.123 | 1627.923 | 70.847 | 2045.849 | 72.230 | 2249.853 | 71.392 | 1430.892 | 71.297 | 1347.451 | 70.985 | 1683.104 |
| 71.236 | 1630.887 | 70.960 | 2051.254 | 72.341 | 2255.615 | 71.504 | 1434.140 | 71.409 | 1350.637 | 71.096 | 1687.271 |
| 71.348 | 1634.484 | 71.073 | 2055.261 | 72.451 | 2259.602 | 71.616 | 1439.414 | 71.521 | 1355.093 | 71.207 | 1690.236 |
| 71.461 | 1639.477 | 71.186 | 2060.411 | 72.562 | 2265.765 | 71.728 | 1444.546 | 71.633 | 1358.485 | 71.318 | 1692.988 |
| 71.573 | 1642.188 | 71.300 | 2065.118 | 72.673 | 2273.010 | 71.840 | 1448.927 | 71.745 | 1362.495 | 71.429 | 1696.719 |
| 71.686 | 1645.435 | 71.413 | 2069.691 | 72.783 | 2278.228 | 71.952 | 1451.034 | 71.857 | 1365.390 | 71.540 | 1702.903 |
| 71.799 | 1652.218 | 71.526 | 2075.701 | 72.894 | 2284.130 | 72.064 | 1453.822 | 71.969 | 1369.282 | 71.652 | 1707.146 |
| 71.911 | 1657.202 | 71.640 | 2083.321 | 73.005 | 2288.891 | 72.176 | 1455.892 | 72.081 | 1372.017 | 71.763 | 1710.778 |
| 72.024 | 1662.797 | 71.753 | 2086.901 | 73.116 | 2291.415 | 72.288 | 1459.511 | 72.193 | 1375.376 | 71.874 | 1714.384 |
| 72.136 | 1667.210 | 71.866 | 2095.369 | 73.226 | 2295.603 | 72.400 | 1463.568 | 72.305 | 1377.921 | 71.985 | 1719.719 |
| 72.249 | 1671.174 | 71.980 | 2101.923 | 73.337 | 2304.438 | 72.512 | 1466.980 | 72.417 | 1381.395 | 72.096 | 1725.968 |
| 72.361 | 1674.944 | 72.093 | 2106.912 | 73.448 | 2310.605 | 72.624 | 1471.217 | 72.529 | 1384.552 | 72.207 | 1728.975 |
| 72.474 | 1677.946 | 72.206 | 2111.136 | 73.558 | 2315.693 | 72.736 | 1475.783 | 72.641 | 1388.358 | 72.318 | 1731.217 |
| 72.587 | 1680.638 | 72.320 | 2115.532 | 73.669 | 2321.272 | 72.848 | 1478.975 | 72.752 | 1392.662 | 72.429 | 1737.014 |
| 72.699 | 1685.484 | 72.433 | 2120.234 | 73.780 | 2325.025 | 72.960 | 1482.207 | 72.864 | 1394.728 | 72.541 | 1743.165 |
| 72.812 | 1691.603 | 72.546 | 2127.989 | 73.890 | 2328.913 | 73.072 | 1485.499 | 72.976 | 1396.912 | 72.652 | 1747.079 |
| 72.924 | 1698.182 | 72.660 | 2134.303 | 74.001 | 2331.479 | 73.184 | 1488.395 | 73.088 | 1399.962 | 72.763 | 1751.838 |
| 73.037 | 1702.572 | 72.773 | 2140.011 | 74.112 | 2337.398 | 73.296 | 1490.794 | 73.200 | 1403.863 | 72.874 | 1756.828 |
| 73.149 | 1706.259 | 72.886 | 2145.166 | 74.222 | 2344.447 | 73.408 | 1493.331 | 73.312 | 1408.071 | 72.985 | 1756.907 |
| 73.262 | 1709.204 | 73.000 | 2149.184 | 74.333 | 2352.728 | 73.520 | 1496.912 | 73.424 | 1413.096 | 73.096 | 1762.805 |
| 73.375 | 1713.797 | 73.113 | 2154.486 | 74.444 | 2356.577 | 73.631 | 1500.276 | 73.536 | 1415.267 | 73.207 | 1767.231 |
| 73.487 | 1717.751 | 73.226 | 2160.187 | 74.555 | 2360.963 | 73.743 | 1505.501 | 73.648 | 1418.146 | 73.319 | 1772.754 |
| 73.600 | 1721.064 | 73.340 | 2165.567 | 74.665 | 2367.348 | 73.855 | 1509.556 | 73.760 | 1422.734 | 73.430 | 1778.596 |
| 73.712 | 1725.976 | 73.453 | 2170.403 | 74.776 | 2371.622 | 73.967 | 1512.918 | 73.872 | 1424.791 | 73.541 | 1782.473 |
| 73.825 | 1729.905 | 73.566 | 2176.565 | 74.887 | 2377.221 | 74.079 | 1515.345 | 73.984 | 1428.207 | 73.652 | 1786.110 |
| 73.937 | 1736.041 | 73.680 | 2185.342 | 74.997 | 2382.739 | 74.191 | 1520.259 | 74.096 | 1431.496 | 73.763 | 1790.756 |
| 74.050 | 1741.497 | 73.793 | 2190.510 | 75.108 | 2389.590 | 74.303 | 1522.540 | 74.208 | 1434.503 | 73.874 | 1795.970 |
| 74.163 | 1745.020 | 73.906 | 2193.480 | 75.219 | 2394.010 | 74.415 | 1526.458 | 74.320 | 1439.983 | 73.985 | 1800.610 |
| 74.275 | 1750.311 | 74.020 | 2197.258 | 75.329 | 2401.121 | 74.527 | 1531.144 | 74.432 | 1442.836 | 74.096 | 1803.760 |
| 74.388 | 1753.952 | 74.133 | 2202.609 | 75.440 | 2406.258 | 74.639 | 1534.889 | 74.543 | 1446.691 | 74.208 | 1807.623 |
| 74.500 | 1757.379 | 74.246 | 2209.642 | 75.551 | 2411.309 | 74.751 | 1537.972 | 74.655 | 1452.291 | 74.319 | 1811.187 |
| 74.613 | 1761.246 | 74.360 | 2215.447 | 75.662 | 2416.320 | 74.863 | 1540.687 | 74.767 | 1456.777 | 74.430 | 1814.888 |
| 74.725 | 1766.810 | 74.473 | 2220.332 | 75.772 | 2422.461 | 74.975 | 1544.245 | 74.879 | 1459.424 | 74.541 | 1820.015 |
| 74.838 | 1773.005 | 74.586 | 2226.533 | 75.883 | 2426.950 | 75.087 | 1548.060 | 74.991 | 1462.542 | 74.652 | 1824.052 |
| 74.950 | 1775.806 | 74.699 | 2234.175 | 75.994 | 2431.363 | 75.199 | 1551.487 | 75.103 | 1465.046 | 74.763 | 1828.255 |
| 75.063 | 1779.382 | 74.813 | 2239.492 | 76.104 | 2435.919 | 75.311 | 1555.051 | 75.215 | 1469.155 | 74.874 | 1830.412 |
| 75.176 | 1781.510 | 74.926 | 2245.684 | 76.215 | 2439.811 | 75.423 | 1557.407 | 75.327 | 1473.160 | 74.985 | 1834.435 |
| 75.288 | 1786.370 | 75.039 | 2251.256 | 76.326 | 2446.822 | 75.535 | 1561.317 | 75.439 | 1476.582 | 75.097 | 1840.068 |
| 75.401 | 1792.269 | 75.153 | 2257.867 | 76.436 | 2454.171 | 75.647 | 1565.684 | 75.551 | 1480.586 | 75.208 | 1845.924 |
| 75.513 | 1797.358 | 75.266 | 2265.630 | 76.547 | 2460.354 | 75.759 | 1571.389 | 75.663 | 1485.834 | 75.319 | 1850.293 |
| 75.626 | 1800.597 | 75.379 | 2268.826 | 76.658 | 2466.325 | 75.871 | 1575.333 | 75.775 | 1489.289 | 75.430 | 1855.797 |
| 75.738 | 1804.050 | 75.493 | 2275.127 | 76.768 | 2469.249 | 75.983 | 1577.654 | 75.887 | 1492.503 | 75.541 | 1861.284 |
| 75.851 | 1810.264 | 75.606 | 2278.513 | 76.879 | 2475.204 | 76.095 | 1580.791 | 75.999 | 1496.561 | 75.652 | 1866.071 |
| 75.964 | 1812.757 | 75.719 | 2284.106 | 76.990 | 2476.121 | 76.207 | 1582.790 | 76.111 | 1500.525 | 75.763 | 1869.317 |
| 76.076 | 1816.901 | 75.833 | 2289.517 | 77.101 | 2484.192 | 76.319 | 1587.246 | 76.223 | 1503.568 | 75.875 | 1874.769 |
| 76.189 | 1821.890 | 75.946 | 2296.239 | 77.211 | 2490.833 | 76.431 | 1592.567 | 76.334 | 1506.691 | 75.986 | 1879.212 |
| 76.301 | 1826.717 | 76.059 | 2301.982 | 77.322 | 2498.061 | 76.543 | 1597.867 | 76.446 | 1510.427 | 76.097 | 1884.156 |
| 76.414 | 1831.310 | 76.173 | 2307.928 | 77.433 | 2504.624 | 76.655 | 1602.043 | 76.558 | 1513.285 | 76.208 | 1887.839 |
| 76.526 | 1834.707 | 76.286 | 2313.444 | 77.543 | 2509.365 | 76.767 | 1605.686 | 76.670 | 1516.823 | 76.319 | 1892.747 |
| 76.639 | 1839.339 | 76.399 | 2318.772 | 77.654 | 2514.537 | 76.879 | 1608.502 | 76.782 | 1519.382 | 76.430 | 1896.179 |
| 76.752 | 1844.266 | 76.513 | 2326.800 | 77.765 | 2520.861 | 76.991 | 1611.963 | 76.894 | 1522.029 | 76.541 | 1899.681 |
| 76.864 | 1848.615 | 76.626 | 2333.039 | 77.875 | 2524.677 | 77.103 | 1616.382 | 77.006 | 1526.514 | 76.652 | 1903.745 |
| 76.977 | 1851.464 | 76.739 | 2339.612 | 77.986 | 2529.519 | 77.215 | 1620.645 | 77.118 | 1531.550 | 76.764 | 1908.477 |
| 77.089 | 1852.826 | 76.853 | 2343.079 | 78.097 | 2538.939 | 77.327 | 1624.994 | 77.230 | 1534.859 | 76.875 | 1912.338 |
| 77.202 | 1857.321 | 76.966 | 2346.360 | 78.208 | 2547.339 | 77.439 | 1628.503 | 77.342 | 1537.902 | 76.986 | 1916.280 |
| 77.314 | 1861.743 | 77.079 | 2351.299 | 78.318 | 2551.753 | 77.551 | 1632.745 | 77.454 | 1541.905 | 77.097 | 1921.095 |
| 77.427 | 1866.813 | 77.193 | 2356.987 | 78.429 | 2557.651 | 77.663 | 1636.787 | 77.566 | 1544.919 | 77.208 | 1925.820 |
| 77.540 | 1872.701 | 77.306 | 2366.065 | 78.540 | 2564.636 | 77.775 | 1640.930 | 77.678 | 1549.309 | 77.319 | 1929.479 |
| 77.652 | 1878.066 | 77.419 | 2371.657 | 78.650 | 2570.107 | 77.887 | 1645.057 | 77.790 | 1552.172 | 77.430 | 1933.656 |
| 77.765 | 1882.025 | 77.533 | 2376.485 | 78.761 | 2573.739 | 77.999 | 1649.111 | 77.902 | 1555.294 | 77.542 | 1939.292 |
| 77.877 | 1886.520 | 77.646 | 2382.916 | 78.872 | 2581.243 | 78.111 | 1655.292 | 78.014 | 1558.648 | 77.653 | 1944.678 |
| 77.990 | 1891.638 | 77.759 | 2391.319 | 78.982 | 2587.641 | 78.223 | 1658.033 | 78.125 | 1561.649 | 77.764 | 1949.739 |
| 78.102 | 1897.278 | 77.872 | 2398.362 | 79.093 | 2593.958 | 78.335 | 1661.762 | 78.237 | 1565.142 | 77.875 | 1953.984 |
| 78.215 | 1902.237 | 77.986 | 2403.320 | 79.204 | 2600.526 | 78.447 | 1667.539 | 78.349 | 1568.945 | 77.986 | 1958.842 |
| 78.328 | 1907.200 | 78.099 | 2408.275 | 79.314 | 2603.580 | 78.559 | 1670.907 | 78.461 | 1573.381 | 78.097 | 1961.172 |
| 78.440 | 1910.586 | 78.212 | 2413.753 | 79.425 | 2611.802 | 78.670 | 1675.180 | 78.573 | 1575.750 | 78.208 | 1963.373 |
| 78.553 | 1914.303 | 78.326 | 2419.828 | 79.536 | 2615.944 | 78.782 | 1678.893 | 78.685 | 1578.259 | 78.319 | 1967.101 |
| 78.665 | 1920.878 | 78.439 | 2424.508 | 79.647 | 2623.974 | 78.894 | 1681.357 | 78.797 | 1581.137 | 78.431 | 1973.500 |
| 78.778 | 1924.766 | 78.552 | 2430.772 | 79.757 | 2628.460 | 79.006 | 1684.787 | 78.909 | 1586.571 | 78.542 | 1979.700 |
| 78.890 | 1930.731 | 78.666 | 2437.365 | 79.868 | 2632.299 | 79.118 | 1688.223 | 79.021 | 1589.319 | 78.653 | 1985.147 |
| 79.003 | 1933.732 | 78.779 | 2443.201 | 79.979 | 2640.330 | 79.230 | 1692.153 | 79.133 | 1593.402 | 78.764 | 1991.757 |
| 79.116 | 1936.329 | 78.892 | 2447.619 | 80.089 | 2648.706 | 79.342 | 1696.304 | 79.245 | 1597.540 | 78.875 | 1996.494 |
| 79.228 | 1940.584 | 79.006 | 2453.714 | 80.200 | 2655.054 | 79.454 | 1701.465 | 79.357 | 1601.060 | 78.986 | 1999.533 |
| 79.341 | 1945.748 | 79.119 | 2462.582 | 80.311 | 2659.618 | 79.566 | 1704.596 | 79.469 | 1604.910 | 79.097 | 2005.256 |
| 79.453 | 1953.816 | 79.232 | 2469.231 | 80.421 | 2666.357 | 79.678 | 1708.192 | 79.581 | 1608.794 | 79.209 | 2010.763 |
| 79.566 | 1955.679 | 79.346 | 2476.631 | 80.532 | 2672.420 | 79.790 | 1712.670 | 79.693 | 1611.645 | 79.320 | 2016.136 |
| 79.678 | 1957.611 | 79.459 | 2481.447 | 80.643 | 2679.140 | 79.902 | 1716.574 | 79.805 | 1615.943 | 79.431 | 2021.758 |
| 79.791 | 1964.225 | 79.572 | 2487.597 | 80.754 | 2684.164 | 80.014 | 1721.699 | 79.916 | 1619.522 | 79.542 | 2024.817 |
| 79.904 | 1970.804 | 79.686 | 2493.780 | 80.864 | 2691.869 | 80.126 | 1725.565 | 80.028 | 1623.395 | 79.653 | 2029.089 |
| 80.016 | 1975.820 | 79.799 | 2500.182 | 80.975 | 2698.212 | 80.238 | 1729.327 | 80.140 | 1628.303 | 79.764 | 2034.380 |
| 80.129 | 1979.888 | 79.912 | 2507.327 | 81.086 | 2704.475 | 80.350 | 1734.218 | 80.252 | 1631.966 | 79.875 | 2037.740 |
| 80.241 | 1983.805 | 80.026 | 2511.921 | 81.196 | 2710.422 | 80.462 | 1739.407 | 80.364 | 1635.656 | 79.986 | 2041.997 |
| 80.354 | 1988.690 | 80.139 | 2518.441 | 81.307 | 2714.171 | 80.574 | 1742.140 | 80.476 | 1639.572 | 80.098 | 2047.994 |
| 80.466 | 1989.900 | 80.252 | 2524.430 | 81.418 | 2718.821 | 80.686 | 1745.948 | 80.588 | 1643.555 | 80.209 | 2053.881 |
| 80.579 | 1996.144 | 80.366 | 2530.198 | 81.528 | 2724.409 | 80.798 | 1749.807 | 80.700 | 1648.888 | 80.320 | 2057.813 |
| 80.692 | 2001.940 | 80.479 | 2531.761 | 81.639 | 2729.928 | 80.910 | 1755.014 | 80.812 | 1653.630 | 80.431 | 2061.760 |
| 80.804 | 2008.070 | 80.592 | 2538.824 | 81.750 | 2736.091 | 81.022 | 1759.197 | 80.924 | 1655.249 | 80.542 | 2067.235 |
| 80.917 | 2012.587 | 80.706 | 2544.041 | 81.860 | 2741.696 | 81.134 | 1762.949 | 81.036 | 1658.260 | 80.653 | 2072.253 |
| 81.029 | 2016.220 | 80.819 | 2553.023 | 81.971 | 2748.390 | 81.246 | 1766.284 | 81.148 | 1662.106 | 80.764 | 2078.423 |
| 81.142 | 2023.246 | 80.932 | 2561.976 | 82.082 | 2755.433 | 81.358 | 1769.770 | 81.260 | 1665.867 | 80.876 | 2082.138 |
| 81.254 | 2028.070 | 81.045 | 2566.913 | 82.193 | 2758.449 | 81.470 | 1773.983 | 81.372 | 1669.792 | 80.987 | 2085.393 |
| 81.367 | 2030.925 | 81.159 | 2570.747 | 82.303 | 2764.262 | 81.582 | 1778.335 | 81.484 | 1674.736 | 81.098 | 2090.208 |
| 81.480 | 2035.841 | 81.272 | 2576.250 | 82.414 | 2769.601 | 81.694 | 1781.842 | 81.596 | 1677.605 | 81.209 | 2094.866 |
| 81.592 | 2038.842 | 81.385 | 2582.467 | 82.525 | 2776.046 | 81.806 | 1786.243 | 81.707 | 1680.693 | 81.320 | 2100.696 |
| 81.705 | 2043.013 | 81.499 | 2588.108 | 82.635 | 2780.795 | 81.918 | 1789.929 | 81.819 | 1683.358 | 81.431 | 2105.069 |
| 81.817 | 2047.709 | 81.612 | 2596.900 | 82.746 | 2787.578 | 82.030 | 1794.626 | 81.931 | 1687.180 | 81.542 | 2111.814 |
| 81.930 | 2052.917 | 81.725 | 2603.263 | 82.857 | 2791.268 | 82.142 | 1796.726 | 82.043 | 1692.259 | 81.653 | 2116.887 |
| 82.042 | 2058.165 | 81.839 | 2609.769 | 82.967 | 2798.927 | 82.254 | 1799.768 | 82.155 | 1697.810 | 81.765 | 2121.671 |
| 82.155 | 2063.222 | 81.952 | 2616.725 | 83.078 | 2806.933 | 82.366 | 1803.636 | 82.267 | 1699.923 | 81.876 | 2126.768 |
| 82.268 | 2067.819 | 82.065 | 2621.645 | 83.189 | 2811.555 | 82.478 | 1807.899 | 82.379 | 1704.517 | 81.987 | 2131.195 |
| 82.380 | 2072.120 | 82.179 | 2629.238 | 83.300 | 2815.380 | 82.590 | 1812.060 | 82.491 | 1709.826 | 82.098 | 2133.897 |
| 82.493 | 2076.699 | 82.292 | 2635.185 | 83.410 | 2821.981 | 82.702 | 1817.893 | 82.603 | 1715.165 | 82.209 | 2138.981 |
| 82.605 | 2079.620 | 82.405 | 2641.566 | 83.521 | 2829.291 | 82.814 | 1822.344 | 82.715 | 1717.857 | 82.320 | 2146.076 |
| 82.718 | 2083.907 | 82.519 | 2645.134 | 83.632 | 2833.757 | 82.926 | 1825.736 | 82.827 | 1721.027 | 82.431 | 2149.706 |
| 82.830 | 2089.517 | 82.632 | 2653.434 | 83.742 | 2838.736 | 83.038 | 1829.760 | 82.939 | 1724.103 | 82.543 | 2154.312 |
| 82.943 | 2093.349 | 82.745 | 2659.117 | 83.853 | 2846.090 | 83.150 | 1833.443 | 83.051 | 1729.720 | 82.654 | 2158.615 |
| 83.056 | 2099.656 | 82.859 | 2664.702 | 83.964 | 2851.762 | 83.262 | 1838.622 | 83.163 | 1734.883 | 82.765 | 2163.922 |
| 83.168 | 2104.771 | 82.972 | 2671.934 | 84.074 | 2858.538 | 83.374 | 1841.412 | 83.275 | 1740.001 | 82.876 | 2169.474 |
| 83.281 | 2107.258 | 83.085 | 2678.885 | 84.185 | 2864.391 | 83.486 | 1844.143 | 83.387 | 1743.990 | 82.987 | 2174.565 |
| 83.393 | 2112.810 | 83.199 | 2685.291 | 84.296 | 2871.131 | 83.598 | 1848.349 | 83.498 | 1747.834 | 83.098 | 2178.442 |
| 83.506 | 2117.707 | 83.312 | 2690.297 | 84.406 | 2877.195 | 83.709 | 1852.415 | 83.610 | 1752.836 | 83.209 | 2183.447 |
| 83.618 | 2123.010 | 83.425 | 2697.511 | 84.517 | 2885.434 | 83.821 | 1856.307 | 83.722 | 1757.884 | 83.320 | 2188.889 |
| 83.731 | 2128.931 | 83.539 | 2703.920 | 84.628 | 2888.679 | 83.933 | 1863.877 | 83.834 | 1762.124 | 83.432 | 2193.285 |
| 83.844 | 2134.426 | 83.652 | 2710.411 | 84.739 | 2892.922 | 84.045 | 1868.724 | 83.946 | 1766.376 | 83.543 | 2200.031 |
| 83.956 | 2138.004 | 83.765 | 2714.549 | 84.849 | 2899.311 | 84.157 | 1873.106 | 84.058 | 1769.800 | 83.654 | 2207.459 |
| 84.069 | 2143.423 | 83.879 | 2721.263 | 84.960 | 2907.877 | 84.269 | 1876.345 | 84.170 | 1773.076 | 83.765 | 2211.020 |
| 84.181 | 2146.933 | 83.992 | 2727.740 | 85.071 | 2913.951 | 84.381 | 1879.501 | 84.282 | 1776.834 | 83.876 | 2217.040 |
| 84.294 | 2155.186 | 84.105 | 2736.224 | 85.181 | 2921.271 | 84.493 | 1883.084 | 84.394 | 1780.477 | 83.987 | 2224.452 |
| 84.406 | 2160.808 | 84.218 | 2743.010 | 85.292 | 2926.818 | 84.605 | 1887.145 | 84.506 | 1784.112 | 84.098 | 2227.399 |
| 84.519 | 2165.507 | 84.332 | 2748.015 | 85.403 | 2931.852 | 84.717 | 1890.590 | 84.618 | 1787.927 | 84.210 | 2232.517 |
| 84.632 | 2169.233 | 84.445 | 2753.438 | 85.513 | 2937.717 | 84.829 | 1896.094 | 84.730 | 1791.760 | 84.321 | 2237.404 |
| 84.744 | 2173.573 | 84.558 | 2759.518 | 85.624 | 2941.516 | 84.941 | 1898.750 | 84.842 | 1795.542 | 84.432 | 2241.918 |
| 84.857 | 2179.671 | 84.672 | 2762.981 | 85.735 | 2947.469 | 85.053 | 1905.075 | 84.954 | 1800.116 | 84.543 | 2247.422 |
| 84.969 | 2184.328 | 84.785 | 2766.528 | 85.846 | 2955.081 | 85.165 | 1909.701 | 85.066 | 1803.635 | 84.654 | 2253.032 |
| 85.082 | 2191.343 | 84.898 | 2770.242 | 85.956 | 2960.540 | 85.277 | 1914.114 | 85.178 | 1807.839 | 84.765 | 2258.733 |
| 85.194 | 2193.438 | 85.012 | 2776.099 | 86.067 | 2965.083 | 85.389 | 1919.109 | 85.289 | 1812.450 | 84.876 | 2263.629 |
| 85.307 | 2199.494 | 85.125 | 2785.636 | 86.178 | 2970.594 | 85.501 | 1923.021 | 85.401 | 1816.336 | 84.987 | 2268.955 |
| 85.420 | 2201.138 | 85.238 | 2792.357 | 86.288 | 2975.584 | 85.613 | 1926.362 | 85.513 | 1820.636 | 85.099 | 2275.075 |
| 85.532 | 2207.543 | 85.352 | 2800.170 | 86.399 | 2980.144 | 85.725 | 1930.372 | 85.625 | 1824.557 | 85.210 | 2278.681 |
| 85.645 | 2214.084 | 85.465 | 2808.056 | 86.510 | 2985.828 | 85.837 | 1933.192 | 85.737 | 1828.481 | 85.321 | 2282.884 |
| 85.757 | 2219.741 | 85.578 | 2813.567 | 86.620 | 2988.300 | 85.949 | 1936.731 | 85.849 | 1832.676 | 85.432 | 2287.872 |
| 85.870 | 2225.707 | 85.692 | 2816.354 | 86.731 | 3000.112 | 86.061 | 1941.827 | 85.961 | 1836.337 | 85.543 | 2292.419 |
| 85.982 | 2233.063 | 85.805 | 2823.402 | 86.842 | 3007.712 | 86.173 | 1946.052 | 86.073 | 1839.734 | 85.654 | 2297.111 |
| 86.095 | 2237.161 | 85.918 | 2832.011 | 86.953 | 3015.351 | 86.285 | 1951.786 | 86.185 | 1842.901 | 85.765 | 2301.865 |
| 86.208 | 2239.633 | 86.032 | 2839.354 | 87.063 | 3021.667 | 86.397 | 1955.656 | 86.297 | 1847.387 | 85.876 | 2307.360 |
| 86.320 | 2243.830 | 86.145 | 2848.013 | 87.174 | 3028.618 | 86.509 | 1958.709 | 86.409 | 1852.573 | 85.988 | 2313.609 |
| 86.433 | 2248.778 | 86.258 | 2855.167 | 87.285 | 3035.164 | 86.621 | 1962.475 | 86.521 | 1855.989 | 86.099 | 2319.205 |
| 86.545 | 2254.651 | 86.372 | 2858.475 | 87.395 | 3040.524 | 86.733 | 1966.856 | 86.633 | 1862.310 | 86.210 | 2325.252 |
| 86.658 | 2259.830 | 86.485 | 2865.116 | 87.506 | 3046.642 | 86.845 | 1970.511 | 86.745 | 1866.117 | 86.321 | 2329.683 |
| 86.770 | 2266.346 | 86.598 | 2874.166 | 87.617 | 3051.398 | 86.957 | 1973.995 | 86.857 | 1869.717 | 86.432 | 2334.478 |
| 86.883 | 2271.337 | 86.712 | 2881.792 | 87.727 | 3057.610 | 87.069 | 1977.467 | 86.969 | 1876.005 | 86.543 | 2340.114 |
| 86.995 | 2275.375 | 86.825 | 2890.055 | 87.838 | 3064.675 | 87.181 | 1983.471 | 87.080 | 1878.968 | 86.654 | 2345.297 |
| 87.108 | 2276.948 | 86.938 | 2897.054 | 87.949 | 3072.476 | 87.293 | 1988.548 | 87.192 | 1882.345 | 86.766 | 2350.943 |
| 87.221 | 2283.593 | 87.052 | 2908.092 | 88.059 | 3079.355 | 87.405 | 1992.668 | 87.304 | 1885.888 | 86.877 | 2357.988 |
| 87.333 | 2290.292 | 87.165 | 2913.441 | 88.170 | 3086.011 | 87.517 | 1996.461 | 87.416 | 1889.387 | 86.988 | 2362.102 |
| 87.446 | 2296.896 | 87.278 | 2917.837 | 88.281 | 3091.201 | 87.629 | 2000.103 | 87.528 | 1894.854 | 87.099 | 2366.937 |
| 87.558 | 2300.687 | 87.392 | 2919.726 | 88.392 | 3099.240 | 87.741 | 2006.727 | 87.640 | 1899.983 | 87.210 | 2372.708 |
| 87.671 | 2306.229 | 87.505 | 2926.665 | 88.502 | 3107.773 | 87.853 | 2011.303 | 87.752 | 1903.813 | 87.321 | 2377.884 |
| 87.783 | 2311.969 | 87.618 | 2931.223 | 88.613 | 3117.342 | 87.965 | 2014.537 | 87.864 | 1908.257 | 87.432 | 2383.676 |
| 87.896 | 2316.603 | 87.731 | 2942.215 | 88.724 | 3123.928 | 88.077 | 2017.821 | 87.976 | 1911.841 | 87.543 | 2389.067 |
| 88.009 | 2321.697 | 87.845 | 2949.999 | 88.834 | 3130.610 | 88.189 | 2021.730 | 88.088 | 1915.589 | 87.655 | 2394.640 |
| 88.121 | 2327.872 | 87.958 | 2953.253 | 88.945 | 3137.020 | 88.301 | 2026.704 | 88.200 | 1920.740 | 87.766 | 2398.927 |
| 88.234 | 2334.932 | 88.071 | 2959.837 | 89.056 | 3143.022 | 88.413 | 2031.068 | 88.312 | 1924.302 | 87.877 | 2401.433 |
| 88.346 | 2342.069 | 88.185 | 2967.562 | 89.166 | 3150.043 | 88.525 | 2034.968 | 88.424 | 1928.120 | 87.988 | 2404.778 |
| 88.459 | 2347.590 | 88.298 | 2973.645 | 89.277 | 3153.805 | 88.637 | 2038.667 | 88.536 | 1932.292 | 88.099 | 2409.741 |
| 88.571 | 2352.227 | 88.411 | 2980.296 | 89.388 | 3160.359 | 88.748 | 2043.410 | 88.648 | 1936.403 | 88.210 | 2414.733 |
| 88.684 | 2356.924 | 88.525 | 2986.708 | 89.499 | 3166.248 | 88.860 | 2048.024 | 88.760 | 1940.248 | 88.321 | 2418.649 |
| 88.797 | 2361.181 | 88.638 | 2994.977 | 89.609 | 3174.182 | 88.972 | 2054.412 | 88.871 | 1944.415 | 88.433 | 2424.317 |
| 88.909 | 2365.070 | 88.751 | 2998.975 | 89.720 | 3181.265 | 89.084 | 2058.236 | 88.983 | 1949.405 | 88.544 | 2429.858 |
| 89.022 | 2367.672 | 88.865 | 3007.468 | 89.831 | 3187.487 | 89.196 | 2061.343 | 89.095 | 1953.114 | 88.655 | 2433.443 |
| 89.134 | 2373.772 | 88.978 | 3012.816 | 89.941 | 3198.827 | 89.308 | 2064.373 | 89.207 | 1956.499 | 88.766 | 2439.177 |
| 89.247 | 2378.834 | 89.091 | 3021.727 | 90.052 | 3204.870 | 89.420 | 2068.220 | 89.319 | 1962.447 | 88.877 | 2444.295 |
| 89.359 | 2385.418 | 89.205 | 3028.475 | 90.163 | 3210.773 | 89.532 | 2072.414 | 89.431 | 1967.716 | 88.988 | 2447.650 |
| 89.472 | 2392.099 | 89.318 | 3034.298 | 90.273 | 3218.069 | 89.644 | 2078.044 | 89.543 | 1971.872 | 89.099 | 2452.879 |
| 89.585 | 2398.303 | 89.431 | 3041.206 | 90.384 | 3219.772 | 89.756 | 2083.676 | 89.655 | 1976.092 | 89.210 | 2459.610 |
| 89.697 | 2403.004 | 89.545 | 3049.721 | 90.495 | 3226.761 | 89.868 | 2087.357 | 89.767 | 1980.800 | 89.322 | 2466.166 |
| 89.810 | 2409.331 | 89.658 | 3051.208 | 90.605 | 3235.553 | 89.980 | 2091.739 | 89.879 | 1984.278 | 89.433 | 2472.042 |
| 89.922 | 2412.724 | 89.771 | 3055.418 | 90.716 | 3249.561 | 90.092 | 2096.319 | 89.991 | 1988.173 | 89.544 | 2476.931 |
| 90.035 | 2416.813 | 89.885 | 3062.854 | 90.827 | 3258.221 | 90.204 | 2099.952 | 90.103 | 1991.815 | 89.655 | 2481.362 |
| 90.147 | 2421.075 | 89.998 | 3072.879 | 90.938 | 3262.256 | 90.316 | 2105.864 | 90.215 | 1995.243 | 89.766 | 2483.265 |
| 90.260 | 2428.806 | 90.111 | 3079.702 | 91.048 | 3266.029 | 90.428 | 2109.989 | 90.327 | 1998.731 | 89.877 | 2489.337 |
| 90.373 | 2434.471 | 90.225 | 3083.558 | 91.159 | 3268.726 | 90.540 | 2114.262 | 90.439 | 2001.912 | 89.988 | 2494.667 |
| 90.485 | 2439.142 | 90.338 | 3091.744 | 91.270 | 3275.228 | 90.652 | 2119.833 | 90.551 | 2005.406 | 90.100 | 2499.977 |
| 90.598 | 2443.725 | 90.451 | 3100.159 | 91.380 | 3284.295 | 90.764 | 2124.398 | 90.662 | 2009.905 | 90.211 | 2509.264 |
| 90.710 | 2448.747 | 90.565 | 3106.379 | 91.491 | 3293.623 | 90.876 | 2129.330 | 90.774 | 2015.354 | 90.322 | 2514.113 |
| 90.823 | 2453.930 | 90.678 | 3112.869 | 91.602 | 3303.121 | 90.988 | 2133.172 | 90.886 | 2020.080 | 90.433 | 2517.592 |
| 90.935 | 2457.968 | 90.791 | 3118.939 | 91.712 | 3308.700 | 91.100 | 2137.547 | 90.998 | 2024.191 | 90.544 | 2521.158 |
| 91.048 | 2464.267 | 90.904 | 3126.307 | 91.823 | 3313.931 | 91.212 | 2143.931 | 91.110 | 2028.624 | 90.655 | 2527.219 |
| 91.161 | 2469.466 | 91.018 | 3134.621 | 91.934 | 3320.054 | 91.324 | 2148.975 | 91.222 | 2032.104 | 90.766 | 2536.169 |
| 91.273 | 2476.631 | 91.131 | 3139.271 | 92.045 | 3328.130 | 91.436 | 2155.879 | 91.334 | 2035.306 | 90.877 | 2542.172 |
| 91.386 | 2481.192 | 91.244 | 3141.807 | 92.155 | 3335.593 | 91.548 | 2159.751 | 91.446 | 2038.477 | 90.989 | 2545.169 |
| 91.498 | 2488.391 | 91.358 | 3152.008 | 92.266 | 3340.973 | 91.660 | 2164.180 | 91.558 | 2042.886 | 91.100 | 2549.735 |
| 91.611 | 2489.719 | 91.471 | 3156.242 | 92.377 | 3346.818 | 91.772 | 2167.637 | 91.670 | 2046.250 | 91.211 | 2556.199 |
| 91.723 | 2494.879 | 91.584 | 3162.896 | 92.487 | 3355.149 | 91.884 | 2171.136 | 91.782 | 2050.887 | 91.322 | 2563.083 |
| 91.836 | 2498.888 | 91.698 | 3169.418 | 92.598 | 3361.728 | 91.996 | 2175.156 | 91.894 | 2058.348 | 91.433 | 2569.309 |
| 91.949 | 2504.843 | 91.811 | 3181.894 | 92.709 | 3369.275 | 92.108 | 2179.766 | 92.006 | 2060.512 | 91.544 | 2574.651 |
| 92.061 | 2510.194 | 91.924 | 3187.067 | 92.819 | 3373.367 | 92.220 | 2184.099 | 92.118 | 2064.696 | 91.655 | 2581.840 |
| 92.174 | 2513.668 | 92.038 | 3191.548 | 92.930 | 3379.196 | 92.332 | 2188.535 | 92.230 | 2068.687 | 91.767 | 2585.873 |
| 92.286 | 2519.969 | 92.151 | 3203.806 | 93.041 | 3387.465 | 92.444 | 2192.955 | 92.342 | 2075.116 | 91.878 | 2593.058 |
| 92.399 | 2523.941 | 92.264 | 3210.141 | 93.151 | 3393.318 | 92.556 | 2197.147 | 92.453 | 2078.234 | 91.989 | 2598.358 |
| 92.511 | 2531.753 | 92.378 | 3215.458 | 93.262 | 3400.096 | 92.668 | 2200.103 | 92.565 | 2081.045 | 92.100 | 2604.154 |
| 92.624 | 2536.827 | 92.491 | 3222.583 | 93.373 | 3406.969 | 92.780 | 2205.435 | 92.677 | 2087.542 | 92.211 | 2609.061 |
| 92.737 | 2542.918 | 92.604 | 3228.768 | 93.484 | 3412.127 | 92.892 | 2209.603 | 92.789 | 2091.934 | 92.322 | 2613.880 |
| 92.849 | 2547.282 | 92.718 | 3235.538 | 93.594 | 3418.084 | 93.004 | 2216.118 | 92.901 | 2096.551 | 92.433 | 2618.992 |
| 92.962 | 2553.019 | 92.831 | 3240.929 | 93.705 | 3424.154 | 93.116 | 2220.615 | 93.013 | 2101.901 | 92.544 | 2623.030 |
| 93.074 | 2560.047 | 92.944 | 3246.337 | 93.816 | 3431.152 | 93.228 | 2223.662 | 93.125 | 2106.700 | 92.656 | 2631.180 |
| 93.187 | 2568.415 | 93.058 | 3253.194 | 93.926 | 3439.119 | 93.340 | 2228.241 | 93.237 | 2111.744 | 92.767 | 2636.151 |
| 93.299 | 2574.661 | 93.171 | 3260.520 | 94.037 | 3446.358 | 93.452 | 2231.350 | 93.349 | 2114.806 | 92.878 | 2642.798 |
| 93.412 | 2576.729 | 93.284 | 3267.339 | 94.148 | 3455.238 | 93.564 | 2237.698 | 93.461 | 2119.322 | 92.989 | 2646.389 |
| 93.525 | 2584.313 | 93.398 | 3274.140 | 94.258 | 3454.460 | 93.676 | 2242.781 | 93.573 | 2123.701 | 93.100 | 2651.985 |
| 93.637 | 2589.455 | 93.511 | 3280.510 | 94.369 | 3454.840 | 93.788 | 2247.024 | 93.685 | 2127.955 | 93.211 | 2660.689 |
| 93.750 | 2594.805 | 93.624 | 3286.705 | 94.480 | 3463.495 | 93.899 | 2252.236 | 93.797 | 2132.747 | 93.322 | 2663.837 |
| 93.862 | 2600.135 | 93.738 | 3295.790 | 94.591 | 3470.632 | 94.011 | 2255.480 | 93.909 | 2136.284 | 93.434 | 2669.853 |
| 93.975 | 2605.614 | 93.851 | 3300.030 | 94.701 | 3476.964 | 94.123 | 2260.290 | 94.021 | 2141.635 | 93.545 | 2677.174 |
| 94.087 | 2609.067 | 93.964 | 3308.547 | 94.812 | 3487.824 | 94.235 | 2266.218 | 94.133 | 2146.048 | 93.656 | 2683.192 |
| 94.200 | 2609.882 | 94.077 | 3317.295 | 94.923 | 3495.075 | 94.347 | 2271.647 | 94.244 | 2151.250 | 93.767 | 2689.475 |
| 94.313 | 2616.870 | 94.191 | 3320.764 | 95.033 | 3502.519 | 94.459 | 2276.438 | 94.356 | 2155.344 | 93.878 | 2694.453 |
| 94.425 | 2624.362 | 94.304 | 3323.761 | 95.144 | 3507.033 | 94.571 | 2281.015 | 94.468 | 2159.822 | 93.989 | 2699.275 |
| 94.538 | 2629.338 | 94.417 | 3332.260 | 95.255 | 3514.466 | 94.683 | 2285.955 | 94.580 | 2165.040 | 94.100 | 2704.016 |
| 94.650 | 2635.202 | 94.531 | 3340.668 | 95.365 | 3522.654 | 94.795 | 2290.753 | 94.692 | 2167.988 | 94.211 | 2710.463 |
| 94.763 | 2640.047 | 94.644 | 3347.773 | 95.476 | 3528.157 | 94.907 | 2295.054 | 94.804 | 2172.759 | 94.323 | 2716.404 |
| 94.875 | 2646.407 | 94.757 | 3353.734 | 95.587 | 3538.276 | 95.019 | 2299.117 | 94.916 | 2178.851 | 94.434 | 2721.677 |
| 94.988 | 2650.671 | 94.871 | 3361.811 | 95.697 | 3545.730 | 95.131 | 2303.380 | 95.028 | 2181.720 | 94.545 | 2728.469 |
| 95.101 | 2658.757 | 94.984 | 3364.090 | 95.808 | 3554.097 | 95.243 | 2308.592 | 95.140 | 2187.590 | 94.656 | 2731.169 |
| 95.213 | 2663.599 | 95.097 | 3374.891 | 95.919 | 3562.258 | 95.355 | 2313.044 | 95.252 | 2192.323 | 94.767 | 2738.723 |
| 95.326 | 2668.154 | 95.211 | 3378.770 | 96.030 | 3570.099 | 95.467 | 2318.438 | 95.364 | 2196.963 | 94.878 | 2745.862 |
| 95.438 | 2672.990 | 95.324 | 3383.786 | 96.140 | 3576.887 | 95.579 | 2321.162 | 95.476 | 2201.967 | 94.989 | 2748.919 |
| 95.551 | 2678.549 | 95.437 | 3391.282 | 96.251 | 3582.342 | 95.691 | 2325.324 | 95.588 | 2205.417 | 95.101 | 2752.437 |
| 95.663 | 2684.744 | 95.551 | 3397.949 | 96.362 | 3588.362 | 95.803 | 2330.481 | 95.700 | 2210.750 | 95.212 | 2759.163 |
| 95.776 | 2689.950 | 95.664 | 3403.417 | 96.472 | 3595.555 | 95.915 | 2336.585 | 95.812 | 2216.002 | 95.323 | 2772.175 |
| 95.889 | 2696.830 | 95.777 | 3415.293 | 96.583 | 3599.778 | 96.027 | 2340.551 | 95.924 | 2219.603 | 95.434 | 2777.623 |
| 96.001 | 2701.909 | 95.891 | 3423.708 | 96.694 | 3608.544 | 96.139 | 2345.436 | 96.035 | 2225.073 | 95.545 | 2782.494 |
| 96.114 | 2706.631 | 96.004 | 3428.887 | 96.804 | 3617.113 | 96.251 | 2351.218 | 96.147 | 2229.167 | 95.656 | 2786.711 |
| 96.226 | 2711.400 | 96.117 | 3436.093 | 96.915 | 3628.298 | 96.363 | 2355.460 | 96.259 | 2235.957 | 95.767 | 2793.192 |
| 96.339 | 2715.211 | 96.231 | 3447.958 | 97.026 | 3635.724 | 96.475 | 2359.973 | 96.371 | 2237.873 | 95.878 | 2800.317 |
| 96.451 | 2719.067 | 96.344 | 3450.646 | 97.137 | 3639.002 | 96.587 | 2364.824 | 96.483 | 2243.013 | 95.990 | 2804.760 |
| 96.564 | 2723.827 | 96.457 | 3455.644 | 97.247 | 3642.666 | 96.699 | 2369.225 | 96.595 | 2246.817 | 96.101 | 2809.925 |
| 96.677 | 2730.016 | 96.571 | 3460.686 | 97.358 | 3654.256 | 96.811 | 2373.642 | 96.707 | 2251.576 | 96.212 | 2814.615 |
| 96.789 | 2734.117 | 96.684 | 3471.544 | 97.469 | 3664.079 | 96.923 | 2378.521 | 96.819 | 2256.529 | 96.323 | 2820.416 |
| 96.902 | 2738.889 | 96.797 | 3481.951 | 97.579 | 3670.016 | 97.035 | 2379.940 | 96.931 | 2262.599 | 96.434 | 2827.055 |
| 97.014 | 2745.131 | 96.911 | 3493.460 | 97.690 | 3676.356 | 97.147 | 2385.247 | 97.043 | 2268.342 | 96.545 | 2835.008 |
| 97.127 | 2751.114 | 97.024 | 3501.599 | 97.801 | 3682.490 | 97.259 | 2393.338 | 97.155 | 2272.811 | 96.656 | 2838.667 |
| 97.239 | 2755.712 | 97.137 | 3509.659 | 97.911 | 3690.757 | 97.371 | 2399.007 | 97.267 | 2277.478 | 96.767 | 2843.149 |
| 97.352 | 2760.894 | 97.250 | 3514.227 | 98.022 | 3701.232 | 97.483 | 2403.162 | 97.379 | 2281.342 | 96.879 | 2849.832 |
| 97.465 | 2767.084 | 97.364 | 3523.982 | 98.133 | 3707.288 | 97.595 | 2407.844 | 97.491 | 2286.014 | 96.990 | 2856.470 |
| 97.577 | 2773.162 | 97.477 | 3533.132 | 98.243 | 3712.995 | 97.707 | 2411.412 | 97.603 | 2289.810 | 97.101 | 2862.230 |
| 97.690 | 2777.974 | 97.590 | 3539.210 | 98.354 | 3715.650 | 97.819 | 2415.660 | 97.715 | 2294.114 | 97.212 | 2869.394 |
| 97.802 | 2779.686 | 97.704 | 3545.604 | 98.465 | 3731.042 | 97.931 | 2420.759 | 97.826 | 2297.797 | 97.323 | 2874.115 |
| 97.915 | 2787.303 | 97.817 | 3552.338 | 98.576 | 3741.692 | 98.043 | 2426.731 | 97.938 | 2301.212 | 97.434 | 2880.244 |
| 98.027 | 2796.402 | 97.930 | 3562.553 | 98.686 | 3748.046 | 98.155 | 2431.585 | 98.050 | 2307.863 | 97.545 | 2887.751 |
| 98.140 | 2801.592 | 98.044 | 3574.350 | 98.797 | 3753.886 | 98.267 | 2435.042 | 98.162 | 2311.903 | 97.657 | 2893.147 |
| 98.253 | 2805.088 | 98.157 | 3578.716 | 98.908 | 3761.773 | 98.379 | 2439.395 | 98.274 | 2315.200 | 97.768 | 2900.090 |
| 98.365 | 2806.995 | 98.270 | 3585.354 | 99.018 | 3769.040 | 98.491 | 2445.079 | 98.386 | 2319.161 | 97.879 | 2906.325 |
| 98.478 | 2814.536 | 98.384 | 3591.488 | 99.129 | 3777.527 | 98.603 | 2450.156 | 98.498 | 2324.123 | 97.990 | 2912.640 |
| 98.590 | 2821.616 | 98.497 | 3598.202 | 99.240 | 3783.268 | 98.715 | 2456.028 | 98.610 | 2329.704 | 98.101 | 2918.461 |
| 98.703 | 2830.086 | 98.610 | 3606.653 | 99.350 | 3787.239 | 98.827 | 2462.253 | 98.722 | 2335.001 | 98.212 | 2924.131 |
| 98.815 | 2837.018 | 98.724 | 3613.900 | 99.461 | 3795.651 | 98.938 | 2466.473 | 98.834 | 2340.232 | 98.323 | 2929.474 |
| 98.928 | 2844.115 | 98.837 | 3620.294 | 99.572 | 3804.304 | 99.050 | 2470.638 | 98.946 | 2345.036 | 98.434 | 2936.838 |
| 99.040 | 2849.898 | 98.950 | 3631.500 | 99.683 | 3811.653 | 99.162 | 2476.726 | 99.058 | 2348.686 | 98.546 | 2943.915 |
| 99.153 | 2855.144 | 99.064 | 3638.805 | 99.793 | 3819.992 | 99.274 | 2483.171 | 99.170 | 2353.455 | 98.657 | 2950.093 |
| 99.266 | 2861.035 | 99.177 | 3643.092 | 99.904 | 3826.832 | 99.386 | 2489.604 | 99.282 | 2359.521 | 98.768 | 2957.203 |
| 99.378 | 2870.701 | 99.290 | 3648.174 | 100.015 | 3833.688 | 99.498 | 2495.266 | 99.394 | 2363.605 | 98.879 | 2961.695 |
| 99.491 | 2876.443 | 99.404 | 3656.024 | 100.125 | 3837.057 | 99.610 | 2499.389 | 99.506 | 2366.862 | 98.990 | 2968.164 |
| 99.603 | 2882.540 | 99.517 | 3662.616 | 100.236 | 3843.859 | 99.722 | 2504.033 | 99.617 | 2371.294 | 99.101 | 2974.622 |
| 99.716 | 2884.993 | 99.630 | 3657.441 | 100.347 | 3852.540 | 99.834 | 2507.611 | 99.729 | 2376.082 | 99.212 | 2981.407 |
| 99.828 | 2890.026 | 99.744 | 3657.126 | 100.457 | 3861.930 | 99.946 | 2512.862 | 99.841 | 2380.684 | 99.324 | 2983.931 |
| 99.941 | 2897.535 | 99.857 | 3658.458 | 100.568 | 3870.013 | 100.058 | 2519.430 | 99.953 | 2384.180 | 99.435 | 2992.420 |
| 100.054 | 2905.548 | 99.970 | 3658.304 | 100.679 | 3877.361 | 100.170 | 2521.377 | 100.065 | 2388.969 | 99.546 | 2996.513 |
| 100.166 | 2911.050 | 99.720 | 3551.587 | 100.789 | 3886.845 | 100.282 | 2524.147 | 100.177 | 2393.278 | 99.657 | 3002.428 |
| 100.279 | 2914.190 | 99.595 | 3537.994 | 100.900 | 3895.901 | 100.394 | 2527.237 | 100.289 | 2395.000 | 99.768 | 3011.176 |
| 101.686 | 2927.010 | 99.470 | 3523.131 | 101.011 | 3902.845 | 100.506 | 2531.329 | 100.401 | 2399.481 | 99.879 | 3017.440 |
| 101.597 | 2922.466 | 99.344 | 3504.285 | 101.122 | 3907.416 | 100.618 | 2532.864 | 100.513 | 2402.449 | 99.990 | 3026.368 |
| 101.509 | 2917.480 | 99.219 | 3491.128 | 101.232 | 3912.408 | 100.730 | 2536.338 | 100.625 | 2405.612 | 100.101 | 3032.490 |
| 101.420 | 2916.317 | 99.094 | 3477.564 | 101.343 | 3918.550 | 100.842 | 2537.774 | 100.737 | 2408.272 | 100.213 | 3037.565 |
| 101.332 | 2903.892 | 98.969 | 3463.692 | 101.454 | 3923.709 | 100.770 | 2466.401 | 100.849 | 2411.145 | 100.324 | 3044.732 |
| 101.243 | 2898.941 | 98.844 | 3449.834 | 101.564 | 3928.237 | 100.695 | 2460.351 | 100.961 | 2413.645 | 100.435 | 3049.002 |
| 101.155 | 2890.379 | 98.718 | 3436.126 | 101.675 | 3932.528 | 100.619 | 2449.960 | 101.073 | 2416.181 | 100.546 | 3053.176 |
| 101.066 | 2873.952 | 98.593 | 3421.628 | 101.786 | 3937.513 | 100.544 | 2441.519 | 101.185 | 2417.898 | 100.657 | 3058.656 |
| 100.978 | 2872.264 | 98.468 | 3404.774 | 101.896 | 3942.498 | 100.468 | 2430.526 | 101.297 | 2419.958 | 100.768 | 3062.009 |
| 100.889 | 2859.999 | 98.343 | 3391.068 | 102.007 | 3947.857 | 100.393 | 2429.159 | 101.409 | 2422.118 | 100.879 | 3071.556 |
| 100.801 | 2848.233 | 98.217 | 3378.270 | 102.118 | 3953.027 | 100.317 | 2425.680 | 101.520 | 2423.815 | 100.991 | 3072.761 |
| 100.712 | 2849.083 | 98.092 | 3365.347 | 102.229 | 3958.160 | 100.242 | 2421.121 | 101.632 | 2424.611 | 101.102 | 3072.223 |
| 100.624 | 2840.169 | 97.967 | 3352.441 | 102.339 | 3963.148 | 100.166 | 2414.203 | 101.744 | 2424.669 | 101.213 | 3077.961 |
| 100.535 | 2829.618 | 97.842 | 3339.183 | 102.450 | 3968.188 | 100.090 | 2400.908 | 101.856 | 2431.593 | 101.324 | 3078.837 |
| 100.447 | 2823.878 | 97.717 | 3325.124 | 102.561 | 3972.803 | 100.015 | 2395.075 | 101.968 | 2444.049 | 101.435 | 3085.447 |
| 100.359 | 2815.248 | 97.591 | 3311.852 | 102.671 | 3976.929 | 99.939 | 2387.065 | 102.080 | 2457.391 | 101.546 | 3088.709 |
| 100.270 | 2803.652 | 97.466 | 3299.128 | 102.782 | 3980.951 | 99.864 | 2380.190 | 102.192 | 2470.578 | 101.657 | 3091.846 |
| 100.182 | 2794.326 | 97.341 | 3286.424 | 102.793 | 3989.365 | 99.788 | 2373.055 | 101.726 | 2437.839 | 101.657 | 3091.846 |
| 100.093 | 2786.455 | 97.216 | 3273.558 | 102.673 | 3976.057 | 99.713 | 2364.828 | 101.624 | 2422.157 | 101.353 | 3065.087 |
| 100.005 | 2776.351 | 97.090 | 3261.055 | 102.553 | 3961.906 | 99.637 | 2357.939 | 101.523 | 2405.443 | 101.225 | 3049.331 |
| 99.916 | 2764.929 | 96.965 | 3248.503 | 102.432 | 3948.736 | 99.562 | 2351.365 | 101.421 | 2387.735 | 101.096 | 3041.164 |
| 99.828 | 2754.135 | 96.840 | 3236.072 | 102.312 | 3934.551 | 99.486 | 2344.701 | 101.320 | 2385.814 | 100.968 | 3028.526 |
| 99.739 | 2743.452 | 96.715 | 3224.399 | 102.192 | 3916.954 | 99.411 | 2337.572 | 101.218 | 2383.646 | 100.840 | 3007.721 |
| 99.651 | 2732.922 | 96.590 | 3212.651 | 102.072 | 3902.113 | 99.335 | 2329.644 | 101.117 | 2381.627 | 100.711 | 2987.610 |
| 99.562 | 2723.056 | 96.464 | 3200.979 | 101.951 | 3893.624 | 99.260 | 2320.747 | 101.015 | 2373.496 | 100.583 | 2972.355 |
| 99.474 | 2713.989 | 96.339 | 3189.309 | 101.831 | 3883.054 | 99.184 | 2311.822 | 100.913 | 2369.549 | 100.454 | 2955.843 |
| 99.385 | 2706.142 | 96.214 | 3177.686 | 101.711 | 3864.083 | 99.108 | 2303.776 | 100.812 | 2363.197 | 100.326 | 2941.500 |
| 99.297 | 2697.825 | 96.089 | 3165.626 | 101.590 | 3855.965 | 99.033 | 2297.381 | 100.710 | 2344.465 | 100.198 | 2929.167 |
| 99.208 | 2689.654 | 95.963 | 3153.371 | 101.470 | 3853.680 | 98.957 | 2291.583 | 100.609 | 2336.285 | 100.069 | 2917.142 |
| 99.120 | 2681.506 | 95.838 | 3140.917 | 101.350 | 3849.183 | 98.882 | 2286.153 | 100.507 | 2330.999 | 99.941 | 2904.621 |
| 99.031 | 2673.461 | 95.713 | 3128.463 | 101.230 | 3848.839 | 98.806 | 2280.718 | 100.406 | 2325.277 | 99.812 | 2890.413 |
| 98.943 | 2665.104 | 95.588 | 3115.510 | 101.109 | 3836.512 | 98.731 | 2275.040 | 100.304 | 2316.258 | 99.684 | 2875.035 |
| 98.854 | 2657.253 | 95.463 | 3102.344 | 100.989 | 3820.951 | 98.655 | 2269.444 | 100.203 | 2309.917 | 99.555 | 2861.120 |
| 98.766 | 2649.797 | 95.337 | 3088.959 | 100.869 | 3809.919 | 98.580 | 2264.001 | 100.101 | 2303.401 | 99.427 | 2848.762 |
| 98.677 | 2642.352 | 95.212 | 3074.808 | 100.748 | 3799.247 | 98.504 | 2258.417 | 100.000 | 2294.687 | 99.299 | 2837.252 |
| 98.589 | 2634.788 | 95.087 | 3060.663 | 100.628 | 3784.627 | 98.429 | 2252.879 | 99.898 | 2286.447 | 99.170 | 2826.174 |
| 98.500 | 2627.233 | 94.962 | 3046.309 | 100.508 | 3770.349 | 98.353 | 2247.333 | 99.797 | 2278.718 | 99.042 | 2814.861 |
| 98.412 | 2619.794 | 94.836 | 3031.340 | 100.388 | 3747.830 | 98.278 | 2241.840 | 99.695 | 2270.648 | 98.913 | 2803.174 |
| 98.323 | 2612.682 | 94.711 | 3016.989 | 100.267 | 3727.362 | 98.202 | 2235.876 | 99.593 | 2262.518 | 98.785 | 2790.839 |
| 98.235 | 2605.612 | 94.586 | 3003.661 | 100.147 | 3712.328 | 98.126 | 2229.898 | 99.492 | 2255.211 | 98.657 | 2778.196 |
| 98.146 | 2598.601 | 94.461 | 2990.569 | 100.027 | 3694.277 | 98.051 | 2223.733 | 99.390 | 2247.884 | 98.528 | 2765.425 |
| 98.058 | 2591.855 | 94.336 | 2978.655 | 99.906 | 3677.589 | 97.975 | 2217.464 | 99.289 | 2239.565 | 98.400 | 2752.216 |
| 97.969 | 2585.698 | 94.210 | 2966.726 | 99.786 | 3666.504 | 97.900 | 2210.967 | 99.187 | 2231.027 | 98.271 | 2739.268 |
| 97.881 | 2579.299 | 94.085 | 2953.983 | 99.666 | 3655.624 | 97.824 | 2204.654 | 99.086 | 2222.774 | 98.143 | 2726.618 |
| 97.792 | 2572.771 | 93.960 | 2939.155 | 99.546 | 3643.935 | 97.749 | 2198.324 | 98.984 | 2214.939 | 98.015 | 2713.577 |
| 97.704 | 2566.349 | 93.835 | 2924.140 | 99.425 | 3632.060 | 97.673 | 2191.452 | 98.883 | 2207.126 | 97.886 | 2701.126 |
| 97.615 | 2559.926 | 93.710 | 2909.499 | 99.305 | 3619.406 | 97.598 | 2184.479 | 98.781 | 2199.287 | 97.758 | 2688.818 |
| 97.527 | 2552.981 | 93.584 | 2896.776 | 99.185 | 3607.243 | 97.522 | 2177.733 | 98.680 | 2191.214 | 97.629 | 2677.158 |
| 97.438 | 2545.911 | 93.459 | 2883.812 | 99.064 | 3594.343 | 97.447 | 2170.840 | 98.578 | 2183.587 | 97.501 | 2666.010 |
| 97.350 | 2538.476 | 93.334 | 2871.427 | 98.944 | 3581.347 | 97.371 | 2164.254 | 98.477 | 2176.084 | 97.373 | 2654.778 |
| 97.261 | 2531.228 | 93.209 | 2859.023 | 98.824 | 3567.852 | 97.296 | 2157.833 | 98.375 | 2168.677 | 97.244 | 2643.291 |
| 97.173 | 2524.039 | 93.083 | 2846.451 | 98.704 | 3553.648 | 97.220 | 2151.289 | 98.274 | 2161.492 | 97.116 | 2631.816 |
| 97.084 | 2516.942 | 92.958 | 2834.111 | 98.583 | 3538.495 | 97.144 | 2144.838 | 98.172 | 2154.452 | 96.987 | 2620.856 |
| 96.996 | 2510.163 | 92.833 | 2822.028 | 98.463 | 3524.839 | 97.069 | 2138.583 | 98.070 | 2147.439 | 96.859 | 2610.089 |
| 96.907 | 2503.041 | 92.708 | 2810.388 | 98.343 | 3511.116 | 96.993 | 2132.356 | 97.969 | 2140.713 | 96.731 | 2598.562 |
| 96.819 | 2495.675 | 92.583 | 2798.244 | 98.223 | 3496.563 | 96.918 | 2126.225 | 97.867 | 2134.097 | 96.602 | 2587.013 |
| 96.730 | 2488.143 | 92.457 | 2786.097 | 98.102 | 3482.849 | 96.842 | 2120.298 | 97.766 | 2127.479 | 96.474 | 2574.364 |
| 96.642 | 2480.526 | 92.332 | 2774.088 | 97.982 | 3470.007 | 96.767 | 2114.603 | 97.664 | 2120.906 | 96.345 | 2561.374 |
| 96.553 | 2472.972 | 92.207 | 2761.682 | 97.862 | 3457.801 | 96.691 | 2109.017 | 97.563 | 2114.342 | 96.217 | 2549.493 |
| 96.465 | 2465.417 | 92.082 | 2748.092 | 97.741 | 3445.527 | 96.616 | 2103.509 | 97.461 | 2107.727 | 96.089 | 2538.348 |
| 96.376 | 2457.842 | 91.956 | 2734.816 | 97.621 | 3433.504 | 96.540 | 2098.056 | 97.360 | 2100.975 | 95.960 | 2527.144 |
| 96.288 | 2450.023 | 91.831 | 2722.158 | 97.501 | 3421.200 | 96.465 | 2092.731 | 97.258 | 2093.788 | 95.832 | 2515.890 |
| 96.199 | 2442.511 | 91.706 | 2709.317 | 97.381 | 3405.662 | 96.389 | 2087.605 | 97.157 | 2086.589 | 95.703 | 2504.292 |
| 96.111 | 2435.218 | 91.581 | 2697.162 | 97.260 | 3387.537 | 96.314 | 2082.577 | 97.055 | 2079.402 | 95.575 | 2492.408 |
| 96.022 | 2428.199 | 91.456 | 2686.093 | 97.140 | 3373.757 | 96.238 | 2077.553 | 96.954 | 2071.877 | 95.447 | 2480.359 |
| 95.934 | 2421.383 | 91.330 | 2675.071 | 97.020 | 3359.582 | 96.163 | 2072.388 | 96.852 | 2064.219 | 95.318 | 2468.044 |
| 95.845 | 2414.567 | 91.205 | 2664.220 | 96.899 | 3345.338 | 96.087 | 2067.099 | 96.751 | 2056.794 | 95.190 | 2456.328 |
| 95.757 | 2407.751 | 91.080 | 2653.369 | 96.779 | 3330.887 | 96.011 | 2061.804 | 96.649 | 2049.524 | 95.061 | 2444.810 |
| 95.668 | 2400.935 | 90.955 | 2642.005 | 96.659 | 3313.074 | 95.936 | 2056.450 | 96.547 | 2042.230 | 94.933 | 2433.199 |
| 95.580 | 2394.354 | 90.829 | 2630.412 | 96.539 | 3298.283 | 95.860 | 2051.027 | 96.446 | 2035.283 | 94.805 | 2421.853 |
| 95.491 | 2387.546 | 90.704 | 2618.432 | 96.418 | 3284.750 | 95.785 | 2045.296 | 96.344 | 2028.348 | 94.676 | 2410.886 |
| 95.403 | 2380.873 | 90.579 | 2607.202 | 96.298 | 3271.916 | 95.709 | 2039.580 | 96.243 | 2021.549 | 94.548 | 2399.960 |
| 95.314 | 2374.239 | 90.454 | 2596.508 | 96.178 | 3259.405 | 95.634 | 2033.863 | 96.141 | 2014.715 | 94.419 | 2389.052 |
| 95.226 | 2367.573 | 90.329 | 2585.772 | 96.057 | 3246.617 | 95.558 | 2028.188 | 96.040 | 2007.630 | 94.291 | 2378.388 |
| 95.137 | 2360.782 | 90.203 | 2574.507 | 95.937 | 3233.404 | 95.483 | 2022.575 | 95.938 | 2000.607 | 94.163 | 2367.689 |
| 95.049 | 2354.004 | 90.078 | 2562.863 | 95.817 | 3220.525 | 95.407 | 2016.946 | 95.837 | 1993.581 | 94.034 | 2356.376 |
| 94.960 | 2347.083 | 89.953 | 2551.289 | 95.697 | 3207.889 | 95.332 | 2011.230 | 95.735 | 1986.612 | 93.906 | 2344.885 |
| 94.872 | 2339.890 | 89.828 | 2539.805 | 95.576 | 3195.321 | 95.256 | 2005.464 | 95.634 | 1979.684 | 93.777 | 2333.090 |
| 94.783 | 2332.684 | 89.702 | 2528.061 | 95.456 | 3183.323 | 95.181 | 1999.473 | 95.532 | 1972.747 | 93.649 | 2321.175 |
| 94.695 | 2325.493 | 89.577 | 2515.002 | 95.336 | 3172.041 | 95.105 | 1993.472 | 95.431 | 1965.699 | 93.520 | 2309.417 |
| 94.606 | 2318.164 | 89.452 | 2502.408 | 95.216 | 3161.077 | 95.029 | 1986.987 | 95.329 | 1958.646 | 93.392 | 2298.072 |
| 94.518 | 2310.799 | 89.327 | 2490.600 | 95.095 | 3149.785 | 94.954 | 1980.280 | 95.227 | 1951.670 | 93.264 | 2287.326 |
| 94.429 | 2303.436 | 89.202 | 2478.567 | 94.975 | 3137.072 | 94.878 | 1973.573 | 95.126 | 1944.699 | 93.135 | 2276.901 |
| 94.341 | 2296.298 | 89.076 | 2468.013 | 94.855 | 3124.138 | 94.803 | 1966.867 | 95.024 | 1937.744 | 93.007 | 2266.563 |
| 94.252 | 2289.167 | 88.951 | 2457.226 | 94.734 | 3111.196 | 94.727 | 1960.039 | 94.923 | 1930.742 | 92.878 | 2255.986 |
| 94.164 | 2282.088 | 88.826 | 2445.160 | 94.614 | 3097.559 | 94.652 | 1953.087 | 94.821 | 1923.599 | 92.750 | 2244.928 |
| 94.075 | 2274.687 | 88.701 | 2432.841 | 94.494 | 3084.053 | 94.576 | 1946.567 | 94.720 | 1916.504 | 92.622 | 2233.557 |
| 93.987 | 2267.227 | 88.576 | 2420.334 | 94.374 | 3070.847 | 94.501 | 1940.204 | 94.618 | 1909.457 | 92.493 | 2221.808 |
| 93.898 | 2260.243 | 88.450 | 2408.176 | 94.253 | 3057.269 | 94.425 | 1934.019 | 94.517 | 1902.518 | 92.365 | 2210.009 |
| 93.810 | 2253.267 | 88.325 | 2396.327 | 94.133 | 3043.387 | 94.350 | 1928.016 | 94.415 | 1895.528 | 92.236 | 2198.519 |
| 93.721 | 2246.063 | 88.200 | 2384.605 | 94.013 | 3030.040 | 94.274 | 1922.002 | 94.314 | 1888.582 | 92.108 | 2187.019 |
| 93.633 | 2238.680 | 88.075 | 2372.354 | 93.892 | 3016.542 | 94.199 | 1915.994 | 94.212 | 1881.476 | 91.980 | 2176.076 |
| 93.544 | 2231.470 | 87.949 | 2360.382 | 93.772 | 3002.737 | 94.123 | 1910.091 | 94.111 | 1874.601 | 91.851 | 2165.735 |
| 93.456 | 2224.499 | 87.824 | 2348.416 | 93.652 | 2988.683 | 94.047 | 1904.411 | 94.009 | 1867.927 | 91.723 | 2155.502 |
| 93.367 | 2217.655 | 87.699 | 2336.683 | 93.532 | 2974.629 | 93.972 | 1898.710 | 93.908 | 1861.130 | 91.594 | 2145.360 |
| 93.279 | 2210.654 | 87.574 | 2325.644 | 93.411 | 2961.060 | 93.896 | 1893.084 | 93.806 | 1854.327 | 91.466 | 2135.362 |
| 93.190 | 2203.591 | 87.449 | 2313.628 | 93.291 | 2949.042 | 93.821 | 1887.479 | 93.704 | 1847.708 | 91.338 | 2125.336 |
| 93.102 | 2196.599 | 87.323 | 2301.016 | 93.171 | 2937.136 | 93.745 | 1881.937 | 93.603 | 1841.251 | 91.209 | 2115.001 |
| 93.013 | 2189.872 | 87.198 | 2288.343 | 93.050 | 2925.319 | 93.670 | 1876.507 | 93.501 | 1834.810 | 91.081 | 2104.205 |
| 92.925 | 2183.158 | 87.073 | 2275.670 | 92.930 | 2912.797 | 93.594 | 1871.162 | 93.400 | 1828.354 | 90.952 | 2092.916 |
| 92.836 | 2176.490 | 86.948 | 2263.760 | 92.810 | 2900.274 | 93.519 | 1865.791 | 93.298 | 1821.852 | 90.824 | 2081.346 |
| 92.748 | 2170.062 | 86.822 | 2252.588 | 92.690 | 2887.751 | 93.443 | 1860.431 | 93.197 | 1815.315 | 90.696 | 2069.534 |
| 92.659 | 2163.639 | 86.697 | 2241.718 | 92.569 | 2875.229 | 93.368 | 1855.015 | 93.095 | 1808.764 | 90.567 | 2058.152 |
| 92.571 | 2157.351 | 86.572 | 2230.530 | 92.449 | 2862.785 | 93.292 | 1849.597 | 92.994 | 1802.148 | 90.439 | 2047.309 |
| 92.482 | 2151.167 | 86.447 | 2219.045 | 92.329 | 2851.230 | 93.217 | 1844.126 | 92.892 | 1795.661 | 90.310 | 2036.443 |
| 92.394 | 2145.115 | 86.322 | 2207.583 | 92.209 | 2840.196 | 93.141 | 1838.654 | 92.791 | 1788.942 | 90.182 | 2025.390 |
| 92.305 | 2139.088 | 86.196 | 2195.406 | 92.088 | 2828.945 | 93.065 | 1833.183 | 92.689 | 1782.046 | 90.054 | 2014.533 |
| 92.217 | 2133.025 | 86.071 | 2183.280 | 91.968 | 2816.863 | 92.990 | 1827.710 | 92.588 | 1775.154 | 89.925 | 2003.609 |
| 92.128 | 2126.908 | 85.946 | 2170.645 | 91.848 | 2804.351 | 92.914 | 1822.117 | 92.486 | 1768.276 | 89.797 | 1992.565 |
| 92.040 | 2120.782 | 85.821 | 2158.326 | 91.727 | 2791.653 | 92.839 | 1816.658 | 92.384 | 1761.271 | 89.668 | 1981.779 |
| 91.951 | 2114.343 | 85.695 | 2143.961 | 91.607 | 2778.768 | 92.763 | 1811.217 | 92.283 | 1754.289 | 89.540 | 1971.007 |
| 91.863 | 2107.857 | 85.570 | 2129.661 | 91.487 | 2765.919 | 92.688 | 1805.730 | 92.181 | 1747.543 | 89.412 | 1960.260 |
| 91.774 | 2101.357 | 85.445 | 2115.735 | 91.367 | 2753.149 | 92.612 | 1800.144 | 92.080 | 1741.047 | 89.283 | 1949.235 |
| 91.686 | 2094.871 | 85.320 | 2103.921 | 91.246 | 2740.246 | 92.537 | 1794.353 | 91.978 | 1734.626 | 89.155 | 1938.578 |
| 91.597 | 2088.439 | 85.195 | 2092.272 | 91.126 | 2727.456 | 92.461 | 1788.509 | 91.877 | 1728.224 | 89.026 | 1928.484 |
| 91.509 | 2082.007 | 85.069 | 2080.631 | 91.006 | 2712.803 | 92.386 | 1782.626 | 91.775 | 1721.883 | 88.898 | 1918.501 |
| 91.420 | 2075.535 | 84.944 | 2069.094 | 90.885 | 2695.966 | 92.310 | 1776.593 | 91.674 | 1715.458 | 88.770 | 1908.541 |
| 91.332 | 2068.908 | 84.819 | 2058.707 | 90.765 | 2678.936 | 92.235 | 1770.500 | 91.572 | 1708.952 | 88.641 | 1898.581 |
| 91.243 | 2061.551 | 84.694 | 2048.552 | 90.645 | 2663.630 | 92.159 | 1764.353 | 91.471 | 1702.344 | 88.513 | 1888.912 |
| 91.155 | 2053.994 | 84.568 | 2039.534 | 90.525 | 2650.006 | 92.083 | 1758.168 | 91.369 | 1695.716 | 88.384 | 1879.118 |
| 91.066 | 2046.124 | 84.443 | 2030.393 | 90.404 | 2637.881 | 92.008 | 1751.971 | 91.268 | 1688.955 | 88.256 | 1869.163 |
| 90.978 | 2038.226 | 84.318 | 2021.176 | 90.284 | 2625.948 | 91.932 | 1745.807 | 91.166 | 1682.212 | 88.128 | 1859.275 |
| 90.889 | 2030.257 | 84.193 | 2012.125 | 90.164 | 2613.317 | 91.857 | 1739.740 | 91.065 | 1675.455 | 87.999 | 1848.985 |
| 90.801 | 2022.943 | 84.068 | 2003.045 | 90.043 | 2600.685 | 91.781 | 1733.610 | 90.963 | 1668.558 | 87.871 | 1838.690 |
| 90.712 | 2015.274 | 83.942 | 1993.723 | 89.923 | 2588.167 | 91.706 | 1727.627 | 90.861 | 1661.865 | 87.742 | 1828.256 |
| 90.624 | 2007.911 | 83.817 | 1984.306 | 89.803 | 2575.034 | 91.630 | 1721.682 | 90.760 | 1655.219 | 87.614 | 1817.874 |
| 90.535 | 2000.751 | 83.692 | 1974.856 | 89.683 | 2562.339 | 91.555 | 1715.745 | 90.658 | 1648.418 | 87.485 | 1807.704 |
| 90.447 | 1993.763 | 83.567 | 1964.901 | 89.562 | 2549.995 | 91.479 | 1709.909 | 90.557 | 1641.643 | 87.357 | 1797.660 |
| 90.358 | 1987.114 | 83.442 | 1955.112 | 89.442 | 2538.282 | 91.404 | 1704.063 | 90.455 | 1634.946 | 87.229 | 1787.527 |
| 90.270 | 1980.465 | 83.316 | 1945.278 | 89.322 | 2526.763 | 91.328 | 1698.208 | 90.354 | 1628.169 | 87.100 | 1777.111 |
| 90.181 | 1973.250 | 83.191 | 1935.077 | 89.201 | 2515.567 | 91.253 | 1692.610 | 90.252 | 1621.691 | 86.972 | 1766.620 |
| 90.093 | 1966.119 | 83.066 | 1924.666 | 89.081 | 2504.102 | 91.177 | 1687.087 | 90.151 | 1615.407 | 86.843 | 1756.129 |
| 90.004 | 1959.363 | 82.941 | 1914.005 | 88.961 | 2492.636 | 91.101 | 1681.522 | 90.049 | 1609.118 | 86.715 | 1745.618 |
| 89.916 | 1952.800 | 82.815 | 1903.141 | 88.841 | 2481.167 | 91.026 | 1675.981 | 89.948 | 1603.029 | 86.587 | 1734.699 |
| 89.827 | 1946.305 | 82.690 | 1892.098 | 88.720 | 2469.595 | 90.950 | 1670.607 | 89.846 | 1597.122 | 86.458 | 1724.164 |
| 89.739 | 1939.908 | 82.565 | 1881.056 | 88.600 | 2458.435 | 90.875 | 1665.348 | 89.745 | 1591.348 | 86.330 | 1713.714 |
| 89.650 | 1933.510 | 82.440 | 1870.013 | 88.480 | 2447.396 | 90.799 | 1660.088 | 89.643 | 1585.622 | 86.201 | 1703.148 |
| 89.562 | 1927.068 | 82.315 | 1859.306 | 88.360 | 2436.322 | 90.724 | 1654.826 | 89.541 | 1579.890 | 86.073 | 1693.079 |
| 89.474 | 1920.832 | 82.189 | 1848.859 | 88.239 | 2424.997 | 90.648 | 1649.578 | 89.440 | 1573.831 | 85.945 | 1683.209 |
| 89.385 | 1914.663 | 82.064 | 1838.835 | 88.119 | 2413.736 | 90.573 | 1644.354 | 89.338 | 1567.699 | 85.816 | 1673.333 |
| 89.297 | 1908.472 | 81.939 | 1828.285 | 87.999 | 2402.178 | 90.497 | 1639.068 | 89.237 | 1561.569 | 85.688 | 1663.490 |
| 89.208 | 1902.211 | 81.814 | 1817.968 | 87.878 | 2389.755 | 90.422 | 1633.703 | 89.135 | 1555.363 | 85.559 | 1653.993 |
| 89.120 | 1895.938 | 81.688 | 1807.654 | 87.758 | 2377.020 | 90.346 | 1628.374 | 89.034 | 1548.912 | 85.431 | 1644.821 |
| 89.031 | 1889.661 | 81.563 | 1797.340 | 87.638 | 2364.426 | 90.271 | 1623.232 | 88.932 | 1542.067 | 85.303 | 1635.642 |
| 88.943 | 1883.435 | 81.438 | 1787.026 | 87.518 | 2352.147 | 90.195 | 1618.142 | 88.831 | 1535.145 | 85.174 | 1626.099 |
| 88.854 | 1877.292 | 81.313 | 1776.951 | 87.397 | 2339.598 | 90.119 | 1613.087 | 88.729 | 1528.286 | 85.046 | 1616.592 |
| 88.766 | 1871.179 | 81.188 | 1767.214 | 87.277 | 2327.378 | 90.044 | 1607.799 | 88.628 | 1521.451 | 84.917 | 1606.687 |
| 88.677 | 1865.065 | 81.062 | 1757.649 | 87.157 | 2314.761 | 89.968 | 1602.462 | 88.526 | 1514.658 | 84.789 | 1596.369 |
| 88.589 | 1858.997 | 80.937 | 1748.362 | 87.036 | 2302.123 | 89.893 | 1597.125 | 88.425 | 1507.867 | 84.661 | 1586.305 |
| 88.500 | 1853.020 | 80.812 | 1739.211 | 86.916 | 2289.539 | 89.817 | 1591.787 | 88.323 | 1501.119 | 84.532 | 1576.280 |
| 88.412 | 1846.865 | 80.687 | 1729.910 | 86.796 | 2276.962 | 89.742 | 1586.403 | 88.222 | 1494.565 | 84.404 | 1566.362 |
| 88.323 | 1840.635 | 80.561 | 1720.609 | 86.676 | 2264.804 | 89.666 | 1581.045 | 88.120 | 1488.172 | 84.275 | 1555.654 |
| 88.235 | 1834.397 | 80.436 | 1711.356 | 86.555 | 2253.183 | 89.591 | 1575.629 | 88.018 | 1481.987 | 84.147 | 1545.019 |
| 88.146 | 1827.631 | 80.311 | 1702.392 | 86.435 | 2242.015 | 89.515 | 1570.231 | 87.917 | 1475.772 | 84.019 | 1534.128 |
| 88.058 | 1820.836 | 80.186 | 1693.428 | 86.315 | 2231.045 | 89.440 | 1564.723 | 87.815 | 1469.423 | 83.890 | 1523.353 |
| 87.969 | 1813.821 | 80.061 | 1684.481 | 86.194 | 2220.603 | 89.364 | 1559.178 | 87.714 | 1463.185 | 83.762 | 1512.924 |
| 87.881 | 1806.545 | 79.935 | 1675.343 | 86.074 | 2210.290 | 89.289 | 1553.549 | 87.612 | 1456.938 | 83.633 | 1502.895 |
| 87.792 | 1799.349 | 79.810 | 1665.998 | 85.954 | 2199.496 | 89.213 | 1547.859 | 87.511 | 1450.649 | 83.505 | 1492.785 |
| 87.704 | 1792.239 | 79.685 | 1656.546 | 85.834 | 2188.641 | 89.138 | 1542.189 | 87.409 | 1444.092 | 83.377 | 1482.817 |
| 87.615 | 1785.115 | 79.560 | 1646.836 | 85.713 | 2177.697 | 89.062 | 1536.303 | 87.308 | 1437.535 | 83.248 | 1473.181 |
| 87.527 | 1777.977 | 79.434 | 1637.017 | 85.593 | 2166.744 | 88.986 | 1530.313 | 87.206 | 1431.056 | 83.120 | 1463.057 |
| 87.438 | 1770.792 | 79.309 | 1627.197 | 85.473 | 2156.072 | 88.911 | 1524.446 | 87.105 | 1424.610 | 82.991 | 1453.214 |
| 87.350 | 1763.340 | 79.184 | 1617.444 | 85.353 | 2145.442 | 88.835 | 1518.761 | 87.003 | 1418.320 | 82.863 | 1443.426 |
| 87.261 | 1755.709 | 79.059 | 1607.668 | 85.232 | 2134.428 | 88.760 | 1513.161 | 86.902 | 1412.081 | 82.735 | 1433.927 |
| 87.173 | 1748.123 | 78.934 | 1598.038 | 85.112 | 2123.290 | 88.684 | 1507.631 | 86.800 | 1405.966 | 82.606 | 1424.581 |
| 87.084 | 1740.589 | 78.808 | 1588.933 | 84.992 | 2112.153 | 88.609 | 1502.068 | 86.699 | 1400.002 | 82.478 | 1415.540 |
| 86.996 | 1733.204 | 78.683 | 1579.735 | 84.871 | 2100.904 | 88.533 | 1496.311 | 86.597 | 1393.933 | 82.349 | 1406.534 |
| 86.907 | 1726.120 | 78.558 | 1570.655 | 84.751 | 2088.485 | 88.458 | 1490.527 | 86.495 | 1387.799 | 82.221 | 1397.383 |
| 86.819 | 1719.055 | 78.433 | 1561.584 | 84.631 | 2075.164 | 88.382 | 1484.722 | 86.394 | 1381.664 | 82.093 | 1388.062 |
| 86.730 | 1712.299 | 78.308 | 1552.980 | 84.511 | 2062.129 | 88.307 | 1478.965 | 86.292 | 1375.568 | 81.964 | 1378.718 |
| 86.642 | 1705.874 | 78.182 | 1544.397 | 84.390 | 2049.720 | 88.231 | 1473.304 | 86.191 | 1369.478 | 81.836 | 1369.439 |
| 86.553 | 1699.410 | 78.057 | 1535.814 | 84.270 | 2037.412 | 88.156 | 1467.631 | 86.089 | 1363.429 | 81.707 | 1360.394 |
| 86.465 | 1692.886 | 77.932 | 1527.558 | 84.150 | 2026.331 | 88.080 | 1461.957 | 85.988 | 1357.668 | 81.579 | 1351.415 |
| 86.376 | 1686.368 | 77.807 | 1519.793 | 84.029 | 2015.636 | 88.004 | 1456.326 | 85.886 | 1351.925 | 81.451 | 1342.121 |
| 86.288 | 1680.024 | 77.681 | 1512.022 | 83.909 | 2004.913 | 87.929 | 1450.679 | 85.785 | 1346.125 | 81.322 | 1332.905 |
| 86.199 | 1673.680 | 77.556 | 1504.384 | 83.789 | 1994.358 | 87.853 | 1445.162 | 85.683 | 1340.264 | 81.194 | 1323.186 |
| 86.111 | 1667.335 | 77.431 | 1496.607 | 83.669 | 1983.873 | 87.778 | 1439.915 | 85.582 | 1334.298 | 81.065 | 1313.173 |
| 86.022 | 1661.258 | 77.306 | 1488.841 | 83.548 | 1973.527 | 87.702 | 1434.747 | 85.480 | 1328.320 | 80.937 | 1302.976 |
| 85.934 | 1655.140 | 77.181 | 1480.999 | 83.428 | 1963.517 | 87.627 | 1429.622 | 85.379 | 1322.124 | 80.808 | 1293.266 |
| 85.845 | 1649.328 | 77.055 | 1472.742 | 83.308 | 1953.568 | 87.551 | 1424.564 | 85.277 | 1315.644 | 80.680 | 1284.021 |
| 85.757 | 1643.515 | 76.930 | 1464.609 | 83.187 | 1943.169 | 87.476 | 1419.473 | 85.175 | 1309.223 | 80.552 | 1274.672 |
| 85.668 | 1637.821 | 76.805 | 1456.537 | 83.067 | 1932.133 | 87.400 | 1414.600 | 85.074 | 1302.843 | 80.423 | 1265.588 |
| 85.580 | 1632.155 | 76.680 | 1448.381 | 82.947 | 1921.146 | 87.325 | 1409.759 | 84.972 | 1296.449 | 80.295 | 1256.745 |
| 85.491 | 1626.580 | 76.554 | 1440.243 | 82.827 | 1910.223 | 87.249 | 1404.929 | 84.871 | 1290.011 | 80.166 | 1247.855 |
| 85.403 | 1620.996 | 76.429 | 1431.730 | 82.706 | 1899.417 | 87.174 | 1400.221 | 84.769 | 1283.673 | 80.038 | 1238.811 |
| 85.314 | 1615.194 | 76.304 | 1422.445 | 82.586 | 1888.615 | 87.098 | 1395.513 | 84.668 | 1277.403 | 79.910 | 1229.584 |
| 85.226 | 1609.356 | 76.179 | 1412.901 | 82.466 | 1877.939 | 87.022 | 1390.805 | 84.566 | 1271.056 | 79.781 | 1220.849 |
| 85.137 | 1603.614 | 76.054 | 1403.379 | 82.346 | 1867.684 | 86.947 | 1386.037 | 84.465 | 1264.692 | 79.653 | 1212.186 |
| 85.049 | 1597.983 | 75.928 | 1393.964 | 82.225 | 1864.725 | 86.871 | 1381.225 | 84.363 | 1258.331 | 79.524 | 1203.520 |
| 84.960 | 1592.320 | 75.803 | 1384.605 | 82.105 | 1856.457 | 86.796 | 1376.370 | 84.262 | 1252.122 | 79.396 | 1195.165 |
| 84.872 | 1586.558 | 75.678 | 1375.165 | 81.985 | 1845.237 | 86.720 | 1371.435 | 84.160 | 1246.437 | 79.268 | 1187.243 |
| 84.783 | 1580.796 | 75.553 | 1366.084 | 81.864 | 1833.647 | 86.645 | 1366.310 | 84.059 | 1242.709 | 79.139 | 1179.128 |
| 84.695 | 1574.992 | 75.427 | 1357.947 | 81.744 | 1821.436 | 86.569 | 1360.060 | 83.957 | 1237.226 | 79.011 | 1171.077 |
| 84.606 | 1568.179 | 75.302 | 1349.837 | 81.624 | 1808.535 | 86.494 | 1353.808 | 83.856 | 1231.262 | 78.882 | 1163.128 |
| 84.518 | 1561.195 | 75.177 | 1341.552 | 81.504 | 1795.244 | 86.418 | 1347.708 | 83.754 | 1225.304 | 78.754 | 1155.180 |
| 84.429 | 1554.406 | 75.052 | 1333.290 | 81.383 | 1782.248 | 86.343 | 1342.490 | 83.652 | 1219.283 | 78.626 | 1147.137 |
| 84.341 | 1547.481 | 74.927 | 1325.583 | 81.263 | 1769.691 | 86.267 | 1337.207 | 83.551 | 1213.311 | 78.497 | 1139.020 |
| 84.252 | 1540.472 | 74.801 | 1317.875 | 81.143 | 1758.137 | 86.192 | 1331.901 | 83.449 | 1207.412 | 78.369 | 1130.701 |
| 84.164 | 1533.276 | 74.676 | 1310.179 | 81.022 | 1746.768 | 86.116 | 1326.577 | 83.348 | 1201.567 | 78.240 | 1122.438 |
| 84.075 | 1526.152 | 74.551 | 1302.786 | 80.902 | 1735.707 | 86.040 | 1321.275 | 83.246 | 1195.735 | 78.112 | 1113.790 |
| 83.987 | 1519.381 | 74.426 | 1295.693 | 80.782 | 1724.970 | 85.965 | 1316.127 | 83.145 | 1189.923 | 77.984 | 1104.444 |
| 83.898 | 1513.170 | 74.301 | 1288.596 | 80.662 | 1714.400 | 85.889 | 1311.121 | 83.043 | 1184.246 | 77.855 | 1095.024 |
| 83.810 | 1507.034 | 74.175 | 1281.469 | 80.541 | 1703.917 | 85.814 | 1306.059 | 82.942 | 1178.770 | 77.727 | 1085.576 |
| 83.721 | 1500.757 | 74.050 | 1274.336 | 80.421 | 1694.303 | 85.738 | 1300.872 | 82.840 | 1173.097 | 77.598 | 1076.192 |
| 83.633 | 1494.716 | 73.925 | 1266.974 | 80.301 | 1685.174 | 85.663 | 1295.530 | 82.739 | 1167.346 | 77.470 | 1066.786 |
| 83.544 | 1488.684 | 73.800 | 1259.429 | 80.180 | 1676.045 | 85.587 | 1290.025 | 82.637 | 1161.360 | 77.342 | 1057.720 |
| 83.456 | 1482.696 | 73.674 | 1251.826 | 80.060 | 1667.290 | 85.512 | 1284.489 | 82.536 | 1155.353 | 77.213 | 1048.799 |
| 83.367 | 1476.791 | 73.549 | 1244.130 | 79.940 | 1658.288 | 85.436 | 1278.953 | 82.434 | 1149.261 | 77.085 | 1039.764 |
| 83.279 | 1470.857 | 73.424 | 1236.309 | 79.820 | 1649.262 | 85.361 | 1273.435 | 82.332 | 1143.246 | 76.956 | 1030.916 |
| 83.190 | 1464.920 | 73.299 | 1228.223 | 79.699 | 1640.232 | 85.285 | 1267.952 | 82.231 | 1137.320 | 76.828 | 1022.827 |
| 83.102 | 1458.986 | 73.174 | 1220.121 | 79.579 | 1631.197 | 85.210 | 1262.385 | 82.129 | 1131.541 | 76.700 | 1014.881 |
| 83.013 | 1453.155 | 73.048 | 1212.007 | 79.459 | 1622.188 | 85.134 | 1256.963 | 82.028 | 1125.822 | 76.571 | 1007.118 |
| 82.925 | 1447.322 | 72.923 | 1204.022 | 79.339 | 1613.202 | 85.058 | 1251.789 | 81.926 | 1120.193 | 76.443 | 999.518 |
| 82.836 | 1441.489 | 72.798 | 1196.134 | 79.218 | 1604.866 | 84.983 | 1246.616 | 81.825 | 1114.639 | 76.314 | 992.410 |
| 82.748 | 1435.782 | 72.673 | 1188.083 | 79.098 | 1596.191 | 84.907 | 1241.618 | 81.723 | 1109.086 | 76.186 | 985.326 |
| 82.659 | 1430.140 | 72.547 | 1180.241 | 78.978 | 1587.460 | 84.832 | 1236.685 | 81.622 | 1103.577 | 76.058 | 978.225 |
| 82.571 | 1424.394 | 72.422 | 1172.591 | 78.857 | 1578.498 | 84.756 | 1231.737 | 81.520 | 1097.940 | 75.929 | 971.011 |
| 82.482 | 1418.694 | 72.297 | 1164.909 | 78.737 | 1569.466 | 84.681 | 1226.789 | 81.419 | 1092.274 | 75.801 | 963.682 |
| 82.394 | 1413.163 | 72.172 | 1157.431 | 78.617 | 1560.182 | 84.605 | 1221.841 | 81.317 | 1086.655 | 75.672 | 956.354 |
| 82.305 | 1407.723 | 72.047 | 1149.996 | 78.497 | 1550.409 | 84.530 | 1216.894 | 81.216 | 1081.046 | 75.544 | 949.019 |
| 82.217 | 1401.954 | 71.921 | 1142.995 | 78.376 | 1540.304 | 84.454 | 1212.014 | 81.114 | 1075.616 | 75.416 | 941.515 |
| 82.128 | 1395.457 | 71.796 | 1136.049 | 78.256 | 1530.066 | 84.379 | 1207.219 | 81.013 | 1070.279 | 75.287 | 934.040 |
| 82.040 | 1388.904 | 71.671 | 1129.156 | 78.136 | 1520.163 | 84.303 | 1202.446 | 80.911 | 1064.810 | 75.159 | 926.520 |
| 81.951 | 1382.385 | 71.546 | 1122.263 | 78.015 | 1510.319 | 84.228 | 1197.675 | 80.809 | 1059.410 | 75.030 | 918.567 |
| 81.863 | 1375.953 | 71.420 | 1115.470 | 77.895 | 1500.105 | 84.152 | 1192.785 | 80.708 | 1054.024 | 74.902 | 910.252 |
| 81.774 | 1370.328 | 71.295 | 1108.670 | 77.775 | 1489.670 | 84.076 | 1187.545 | 80.606 | 1048.608 | 74.773 | 901.860 |
| 81.686 | 1364.650 | 71.170 | 1102.086 | 77.655 | 1479.170 | 84.001 | 1182.334 | 80.505 | 1043.142 | 74.645 | 893.129 |
| 81.597 | 1359.319 | 71.045 | 1095.512 | 77.534 | 1468.865 | 83.925 | 1177.134 | 80.403 | 1037.676 | 74.517 | 884.564 |
| 81.509 | 1353.915 | 70.920 | 1088.538 | 77.414 | 1458.700 | 83.850 | 1171.936 | 80.302 | 1032.143 | 74.388 | 876.434 |
| 81.420 | 1348.414 | 70.794 | 1081.557 | 77.294 | 1449.147 | 83.774 | 1166.579 | 80.200 | 1026.542 | 74.260 | 868.394 |
| 81.332 | 1342.510 | 70.669 | 1074.718 | 77.173 | 1440.150 | 83.699 | 1161.136 | 80.099 | 1020.953 | 74.131 | 859.990 |
| 81.243 | 1336.381 | 70.544 | 1067.881 | 77.053 | 1431.360 | 83.623 | 1155.851 | 79.997 | 1015.455 | 74.003 | 851.684 |
| 81.155 | 1330.246 | 70.419 | 1061.027 | 76.933 | 1422.770 | 83.548 | 1150.567 | 79.896 | 1009.860 | 73.875 | 843.515 |
| 81.066 | 1324.128 | 70.293 | 1054.693 | 76.813 | 1414.374 | 83.472 | 1145.375 | 79.794 | 1004.258 | 73.746 | 835.556 |
| 80.978 | 1318.061 | 70.168 | 1048.680 | 76.692 | 1405.927 | 83.397 | 1140.181 | 79.693 | 998.787 | 73.618 | 827.858 |
| 80.889 | 1311.982 | 70.043 | 1042.731 | 76.572 | 1397.448 | 83.321 | 1134.973 | 79.591 | 993.262 | 73.489 | 820.798 |
| 80.801 | 1306.064 | 69.918 | 1036.783 | 76.452 | 1389.184 | 83.246 | 1129.766 | 79.490 | 987.882 | 73.361 | 813.997 |
| 80.712 | 1300.243 | 69.793 | 1030.774 | 76.331 | 1380.971 | 83.170 | 1124.559 | 79.388 | 982.642 | 73.233 | 806.698 |
| 80.624 | 1294.766 | 69.667 | 1024.448 | 76.211 | 1372.791 | 83.094 | 1119.359 | 79.286 | 977.403 | 73.104 | 799.373 |
| 80.535 | 1288.874 | 69.542 | 1018.111 | 76.091 | 1364.696 | 83.019 | 1114.396 | 79.185 | 972.164 | 72.976 | 792.143 |
| 80.447 | 1282.894 | 69.417 | 1011.760 | 75.971 | 1356.822 | 82.943 | 1109.525 | 79.083 | 966.962 | 72.847 | 785.140 |
| 80.358 | 1276.969 | 69.292 | 1005.608 | 75.850 | 1349.227 | 82.868 | 1104.598 | 78.982 | 961.845 | 72.719 | 777.983 |
| 80.270 | 1270.896 | 69.167 | 999.570 | 75.730 | 1341.535 | 82.792 | 1099.503 | 78.880 | 956.684 | 72.591 | 770.839 |
| 80.181 | 1264.808 | 69.041 | 993.484 | 75.610 | 1333.810 | 82.717 | 1094.360 | 78.779 | 951.275 | 72.462 | 763.669 |
| 80.093 | 1258.760 | 68.916 | 987.347 | 75.490 | 1325.654 | 82.641 | 1089.217 | 78.677 | 945.841 | 72.334 | 756.797 |
| 80.004 | 1252.712 | 68.791 | 981.300 | 75.369 | 1317.359 | 82.566 | 1084.007 | 78.576 | 940.297 | 72.205 | 749.867 |
| 79.916 | 1246.689 | 68.666 | 975.364 | 75.249 | 1309.064 | 82.490 | 1078.776 | 78.474 | 934.685 | 72.077 | 742.953 |
| 79.827 | 1240.623 | 68.540 | 969.473 | 75.129 | 1300.937 | 82.415 | 1073.500 | 78.373 | 929.136 | 71.949 | 735.378 |
| 79.739 | 1235.022 | 68.415 | 963.640 | 75.008 | 1292.957 | 82.339 | 1068.212 | 78.271 | 923.817 | 71.820 | 727.799 |
| 79.650 | 1229.948 | 68.290 | 957.889 | 74.888 | 1284.913 | 82.264 | 1063.061 | 78.170 | 918.586 | 71.692 | 719.964 |
| 79.562 | 1224.883 | 68.165 | 952.138 | 74.768 | 1276.765 | 82.188 | 1057.913 | 78.068 | 913.568 | 71.563 | 712.076 |
| 79.473 | 1219.820 | 68.040 | 946.552 | 74.648 | 1268.324 | 82.113 | 1052.653 | 77.966 | 908.537 | 71.435 | 704.589 |
| 79.385 | 1214.757 | 67.914 | 941.029 | 74.527 | 1259.752 | 82.037 | 1047.577 | 77.865 | 903.625 | 71.307 | 696.502 |
| 79.296 | 1209.320 | 67.789 | 935.502 | 74.407 | 1251.163 | 81.961 | 1042.575 | 77.763 | 898.812 | 71.178 | 688.490 |
| 79.208 | 1204.191 | 67.664 | 929.954 | 74.287 | 1242.575 | 81.886 | 1037.594 | 77.662 | 894.040 | 71.050 | 681.264 |
| 79.119 | 1199.130 | 67.539 | 924.422 | 74.166 | 1234.090 | 81.810 | 1032.580 | 77.560 | 889.240 | 70.921 | 674.335 |
| 79.031 | 1193.969 | 67.413 | 918.859 | 74.046 | 1226.143 | 81.735 | 1027.569 | 77.459 | 884.394 | 70.793 | 667.706 |
| 78.942 | 1188.765 | 67.288 | 913.275 | 73.926 | 1218.705 | 81.659 | 1022.545 | 77.357 | 879.519 | 70.665 | 661.254 |
| 78.854 | 1183.335 | 67.163 | 907.692 | 73.806 | 1211.350 | 81.584 | 1017.594 | 77.256 | 874.561 | 70.536 | 655.011 |
| 78.765 | 1177.905 | 67.038 | 902.320 | 73.685 | 1204.195 | 81.508 | 1012.602 | 77.154 | 869.534 | 70.408 | 648.752 |
| 78.677 | 1172.464 | 66.913 | 897.126 | 73.565 | 1197.168 | 81.433 | 1007.687 | 77.053 | 864.415 | 70.279 | 642.490 |
| 78.588 | 1166.461 | 66.787 | 891.875 | 73.445 | 1189.924 | 81.357 | 1002.828 | 76.951 | 859.264 | 70.151 | 636.258 |
| 78.500 | 1159.626 | 66.662 | 886.514 | 73.324 | 1182.616 | 81.282 | 997.968 | 76.850 | 854.139 | 70.023 | 629.986 |
| 78.412 | 1152.709 | 66.537 | 881.061 | 73.204 | 1175.510 | 81.206 | 993.107 | 76.748 | 849.046 | 69.894 | 623.758 |
| 78.323 | 1145.673 | 66.412 | 875.715 | 73.084 | 1168.613 | 81.131 | 988.359 | 76.647 | 844.019 | 69.766 | 617.574 |
| 78.235 | 1138.983 | 66.286 | 870.368 | 72.964 | 1161.716 | 81.055 | 983.665 | 76.545 | 839.005 | 69.637 | 611.310 |
| 78.146 | 1132.551 | 66.161 | 864.618 | 72.843 | 1154.875 | 80.979 | 979.100 | 76.443 | 834.107 | 69.509 | 605.045 |
| 78.058 | 1126.206 | 66.036 | 858.748 | 72.723 | 1148.138 | 80.904 | 974.527 | 76.342 | 829.207 | 69.381 | 598.797 |
| 77.969 | 1119.771 | 65.911 | 853.113 | 72.603 | 1140.889 | 80.828 | 969.954 | 76.240 | 824.289 | 69.252 | 592.580 |
| 77.881 | 1113.466 | 65.786 | 847.494 | 72.483 | 1133.482 | 80.753 | 965.377 | 76.139 | 819.129 | 69.124 | 586.244 |
| 77.792 | 1107.352 | 65.660 | 842.048 | 72.362 | 1125.911 | 80.677 | 960.929 | 76.037 | 813.926 | 68.995 | 580.036 |
| 77.704 | 1101.555 | 65.535 | 836.634 | 72.242 | 1118.339 | 80.602 | 956.129 | 75.936 | 808.720 | 68.867 | 573.695 |
| 77.615 | 1095.851 | 65.410 | 831.047 | 72.122 | 1110.400 | 80.526 | 951.212 | 75.834 | 803.527 | 68.738 | 567.246 |
| 77.527 | 1090.148 | 65.285 | 825.673 | 72.001 | 1102.535 | 80.451 | 946.422 | 75.733 | 798.341 | 68.610 | 560.466 |
| 77.438 | 1084.433 | 65.159 | 820.559 | 71.881 | 1094.778 | 80.375 | 941.671 | 75.631 | 791.693 | 68.482 | 553.242 |
| 77.350 | 1078.719 | 65.034 | 815.454 | 71.761 | 1087.007 | 80.300 | 936.920 | 75.530 | 786.230 | 68.353 | 546.031 |
| 77.261 | 1073.088 | 64.909 | 810.350 | 71.641 | 1079.183 | 80.224 | 932.159 | 75.428 | 781.128 | 68.225 | 541.757 |
| 77.173 | 1067.505 | 64.784 | 805.275 | 71.520 | 1071.939 | 80.149 | 927.388 | 75.327 | 776.102 | 68.096 | 535.356 |
| 77.084 | 1061.927 | 64.659 | 800.265 | 71.400 | 1064.645 | 80.073 | 922.692 | 75.225 | 771.056 | 67.968 | 528.989 |
| 76.996 | 1056.477 | 64.533 | 795.322 | 71.280 | 1057.246 | 79.997 | 917.934 | 75.123 | 766.187 | 67.840 | 522.721 |
| 76.907 | 1051.264 | 64.408 | 790.519 | 71.159 | 1049.835 | 79.922 | 912.984 | 75.022 | 761.319 | 67.711 | 516.841 |
| 76.819 | 1045.962 | 64.283 | 785.847 | 71.039 | 1042.416 | 79.846 | 908.094 | 74.920 | 756.480 | 67.583 | 510.959 |
| 76.730 | 1040.660 | 64.158 | 781.193 | 70.919 | 1034.990 | 79.771 | 903.334 | 74.819 | 751.841 | 67.454 | 505.027 |
| 76.642 | 1035.519 | 64.033 | 776.539 | 70.799 | 1027.643 | 79.695 | 898.571 | 74.717 | 747.309 | 67.326 | 499.454 |
| 76.553 | 1030.219 | 63.907 | 771.795 | 70.678 | 1020.411 | 79.620 | 893.718 | 74.616 | 742.722 | 67.198 | 494.257 |
| 76.465 | 1024.838 | 63.782 | 766.884 | 70.558 | 1013.573 | 79.544 | 888.822 | 74.514 | 738.086 | 67.069 | 488.899 |
| 76.376 | 1019.458 | 63.657 | 761.974 | 70.438 | 1006.918 | 79.469 | 884.110 | 74.413 | 733.377 | 66.941 | 483.560 |
| 76.288 | 1014.305 | 63.532 | 757.064 | 70.317 | 1000.374 | 79.393 | 879.597 | 74.311 | 728.655 | 66.812 | 478.166 |
| 76.199 | 1009.159 | 63.406 | 752.237 | 70.197 | 994.118 | 79.318 | 875.175 | 74.210 | 723.945 | 66.684 | 472.664 |
| 76.111 | 1003.936 | 63.281 | 747.539 | 70.077 | 987.862 | 79.242 | 870.937 | 74.108 | 719.324 | 66.556 | 467.361 |
| 76.022 | 998.572 | 63.156 | 742.913 | 69.957 | 981.624 | 79.167 | 866.737 | 74.007 | 714.672 | 66.427 | 462.169 |
| 75.934 | 993.200 | 63.031 | 738.287 | 69.836 | 975.453 | 79.091 | 862.537 | 73.905 | 710.077 | 66.299 | 456.958 |
| 75.845 | 988.043 | 62.906 | 733.560 | 69.716 | 969.372 | 79.015 | 858.334 | 73.804 | 705.555 | 66.170 | 451.769 |
| 75.757 | 982.794 | 62.780 | 728.873 | 69.596 | 963.377 | 78.940 | 854.264 | 73.702 | 701.017 | 66.042 | 446.673 |
| 75.668 | 977.517 | 62.655 | 724.239 | 69.476 | 957.562 | 78.864 | 850.202 | 73.600 | 696.398 | 65.914 | 441.626 |
| 75.580 | 972.118 | 62.530 | 719.606 | 69.355 | 951.852 | 78.789 | 846.123 | 73.499 | 691.770 | 65.785 | 436.773 |
| 75.491 | 966.637 | 62.405 | 714.972 | 69.235 | 946.084 | 78.713 | 841.990 | 73.397 | 686.979 | 65.657 | 431.961 |
| 75.403 | 961.155 | 62.279 | 710.455 | 69.115 | 940.402 | 78.638 | 837.793 | 73.296 | 682.082 | 65.528 | 427.100 |
| 75.314 | 955.652 | 62.154 | 706.263 | 68.994 | 934.528 | 78.562 | 833.511 | 73.194 | 677.196 | 65.400 | 422.226 |
| 75.226 | 950.040 | 62.029 | 702.018 | 68.874 | 928.653 | 78.487 | 829.292 | 73.093 | 672.384 | 65.272 | 417.218 |
| 75.137 | 944.481 | 61.904 | 697.742 | 68.754 | 922.805 | 78.411 | 825.110 | 72.991 | 667.571 | 65.143 | 412.172 |
| 75.049 | 938.908 | 61.779 | 693.477 | 68.634 | 916.968 | 78.336 | 820.998 | 72.890 | 662.930 | 65.015 | 407.056 |
| 74.960 | 933.418 | 61.653 | 689.238 | 68.513 | 911.160 | 78.260 | 816.903 | 72.788 | 658.433 | 64.886 | 402.029 |
| 74.872 | 927.955 | 61.528 | 684.729 | 68.393 | 905.403 | 78.185 | 812.865 | 72.687 | 654.031 | 64.758 | 397.283 |
| 74.783 | 922.201 | 61.403 | 680.252 | 68.273 | 899.313 | 78.109 | 808.696 | 72.585 | 649.792 | 64.630 | 392.455 |
| 74.695 | 916.448 | 61.278 | 675.793 | 68.152 | 893.097 | 78.033 | 804.393 | 72.484 | 645.665 | 64.501 | 387.743 |
| 74.606 | 910.961 | 61.152 | 671.474 | 68.032 | 886.892 | 77.958 | 800.164 | 72.382 | 641.513 | 64.373 | 383.173 |
| 74.518 | 905.208 | 61.027 | 667.293 | 67.912 | 880.561 | 77.882 | 795.959 | 72.280 | 637.418 | 64.244 | 378.683 |
| 74.429 | 899.392 | 60.902 | 663.105 | 67.792 | 874.015 | 77.807 | 791.656 | 72.179 | 633.453 | 64.116 | 374.023 |
| 74.341 | 893.516 | 60.777 | 659.037 | 67.671 | 867.770 | 77.731 | 787.078 | 72.077 | 629.442 | 63.988 | 369.256 |
| 74.252 | 888.202 | 60.652 | 655.068 | 67.551 | 861.630 | 77.656 | 782.377 | 71.976 | 625.309 | 63.859 | 364.422 |
| 74.164 | 882.935 | 60.526 | 651.069 | 67.431 | 855.790 | 77.580 | 777.652 | 71.874 | 621.149 | 63.731 | 359.490 |
| 74.075 | 877.538 | 60.401 | 647.053 | 67.310 | 850.071 | 77.505 | 773.065 | 71.773 | 616.943 | 63.602 | 354.457 |
| 73.987 | 872.084 | 60.276 | 643.066 | 67.190 | 844.706 | 77.429 | 768.461 | 71.671 | 612.518 | 63.474 | 349.483 |
| 73.898 | 867.010 | 60.151 | 639.079 | 67.070 | 839.852 | 77.354 | 763.767 | 71.570 | 608.065 | 63.346 | 344.522 |
| 73.810 | 861.985 | 60.025 | 635.050 | 66.950 | 835.158 | 77.278 | 759.083 | 71.468 | 603.539 | 63.217 | 339.970 |
| 73.721 | 857.076 | 59.900 | 631.022 | 66.829 | 830.491 | 77.203 | 754.543 | 71.367 | 599.044 | 63.089 | 335.395 |
| 73.633 | 852.490 | 59.775 | 626.922 | 66.709 | 825.901 | 77.127 | 749.957 | 71.265 | 594.598 | 62.960 | 330.760 |
| 73.544 | 847.905 | 59.650 | 622.788 | 66.589 | 821.340 | 77.051 | 745.558 | 71.164 | 590.133 | 62.832 | 326.215 |
| 73.456 | 843.253 | 59.525 | 618.795 | 66.468 | 816.641 | 76.976 | 741.205 | 71.062 | 585.902 | 62.703 | 321.810 |
| 73.367 | 838.655 | 59.399 | 614.918 | 66.348 | 811.749 | 76.900 | 736.862 | 70.961 | 581.756 | 62.575 | 317.509 |
| 73.279 | 834.153 | 59.274 | 611.004 | 66.228 | 806.857 | 76.825 | 732.473 | 70.859 | 577.545 | 62.447 | 313.246 |
| 73.190 | 829.698 | 59.149 | 606.895 | 66.108 | 801.831 | 76.749 | 728.038 | 70.757 | 573.221 | 62.318 | 309.107 |
| 73.102 | 825.079 | 59.024 | 602.766 | 65.987 | 796.750 | 76.674 | 723.758 | 70.656 | 568.886 | 62.190 | 304.983 |
| 73.013 | 820.350 | 58.899 | 598.637 | 65.867 | 791.659 | 76.598 | 719.529 | 70.554 | 564.572 | 62.061 | 301.012 |
| 72.925 | 815.631 | 58.773 | 594.681 | 65.747 | 786.237 | 76.523 | 715.376 | 70.453 | 560.258 | 61.933 | 297.167 |
| 72.836 | 810.953 | 58.648 | 590.749 | 65.627 | 780.959 | 76.447 | 711.178 | 70.351 | 556.082 | 61.805 | 293.405 |
| 72.748 | 806.161 | 58.523 | 586.858 | 65.506 | 775.864 | 76.372 | 706.920 | 70.250 | 551.970 | 61.676 | 289.688 |
| 72.659 | 801.291 | 58.398 | 583.264 | 65.386 | 770.768 | 76.296 | 702.793 | 70.148 | 547.839 | 61.548 | 286.019 |
| 72.571 | 796.436 | 58.272 | 579.642 | 65.266 | 765.858 | 76.221 | 698.819 | 70.047 | 543.736 | 61.419 | 282.400 |
| 72.482 | 791.504 | 58.147 | 576.172 | 65.145 | 761.284 | 76.145 | 694.902 | 69.945 | 539.617 | 61.291 | 278.733 |
| 72.394 | 786.450 | 58.022 | 572.732 | 65.025 | 756.603 | 76.069 | 690.972 | 69.844 | 535.431 | 61.163 | 274.979 |
| 72.305 | 781.482 | 57.897 | 569.240 | 64.905 | 751.922 | 75.994 | 686.953 | 69.742 | 531.154 | 61.034 | 271.229 |
| 72.217 | 776.353 | 57.772 | 565.743 | 64.785 | 747.570 | 75.918 | 682.886 | 69.641 | 526.951 | 60.906 | 267.700 |
| 72.128 | 771.383 | 57.646 | 562.246 | 64.664 | 743.327 | 75.843 | 678.871 | 69.539 | 522.763 | 60.777 | 264.046 |
| 72.040 | 766.298 | 57.521 | 558.749 | 64.544 | 739.084 | 75.767 | 675.050 | 69.438 | 518.678 | 60.649 | 260.086 |
| 71.951 | 761.129 | 57.396 | 555.205 | 64.424 | 734.816 | 75.692 | 671.267 | 69.336 | 514.651 | 60.521 | 256.023 |
| 71.863 | 755.949 | 57.271 | 551.657 | 64.303 | 730.282 | 75.616 | 667.392 | 69.234 | 510.605 | 60.392 | 251.754 |
| 71.774 | 750.900 | 57.145 | 548.235 | 64.183 | 725.721 | 75.541 | 663.479 | 69.133 | 506.644 | 60.264 | 247.597 |
| 71.686 | 745.922 | 57.020 | 544.848 | 64.063 | 721.079 | 75.465 | 659.564 | 69.031 | 502.659 | 60.135 | 243.771 |
| 71.597 | 741.021 | 56.895 | 541.394 | 63.943 | 716.411 | 75.390 | 655.572 | 68.930 | 498.587 | 60.007 | 240.184 |
| 71.509 | 736.215 | 56.770 | 537.933 | 63.822 | 711.742 | 75.314 | 651.422 | 68.828 | 494.549 | 59.879 | 236.598 |
| 71.420 | 731.210 | 56.645 | 534.450 | 63.702 | 707.096 | 75.239 | 647.274 | 68.727 | 490.424 | 59.750 | 233.037 |
| 71.332 | 725.983 | 56.519 | 530.994 | 63.582 | 702.564 | 75.163 | 643.125 | 68.625 | 486.272 | 59.622 | 229.570 |
| 71.243 | 720.660 | 56.394 | 527.620 | 63.461 | 698.190 | 75.088 | 638.923 | 68.524 | 482.113 | 59.493 | 226.040 |
| 71.155 | 715.353 | 56.269 | 524.252 | 63.341 | 694.377 | 75.012 | 634.646 | 68.422 | 477.832 | 59.365 | 222.604 |
| 71.066 | 710.043 | 56.144 | 520.833 | 63.221 | 690.680 | 74.936 | 630.303 | 68.321 | 473.426 | 59.237 | 219.268 |
| 70.978 | 704.710 | 56.018 | 517.326 | 63.101 | 686.970 | 74.861 | 626.130 | 68.219 | 468.949 | 59.108 | 215.938 |
| 70.889 | 699.390 | 55.893 | 513.711 | 62.980 | 683.362 | 74.785 | 622.093 | 68.118 | 464.417 | 58.980 | 212.618 |
| 70.801 | 694.525 | 55.768 | 510.092 | 62.860 | 679.800 | 74.710 | 617.890 | 68.016 | 460.245 | 58.851 | 209.299 |
| 70.712 | 689.865 | 55.643 | 506.438 | 62.740 | 676.235 | 74.634 | 613.684 | 67.914 | 455.988 | 58.723 | 205.969 |
| 70.624 | 685.185 | 55.518 | 502.722 | 62.620 | 672.550 | 74.559 | 609.438 | 67.813 | 451.694 | 58.595 | 202.854 |
| 70.535 | 680.636 | 55.392 | 499.060 | 62.499 | 668.864 | 74.483 | 605.165 | 67.711 | 447.386 | 58.466 | 199.983 |
| 70.447 | 676.116 | 55.267 | 495.636 | 62.379 | 665.179 | 74.408 | 600.915 | 67.610 | 443.073 | 58.338 | 197.084 |
| 70.358 | 671.626 | 55.142 | 492.230 | 62.259 | 661.580 | 74.332 | 596.666 | 67.508 | 438.790 | 58.209 | 194.067 |
| 70.270 | 667.140 | 55.017 | 488.753 | 62.138 | 658.029 | 74.257 | 592.424 | 67.407 | 434.725 | 58.081 | 190.780 |
| 70.181 | 662.463 | 54.891 | 485.186 | 62.018 | 654.467 | 74.181 | 588.165 | 67.305 | 430.783 | 57.953 | 187.413 |
| 70.093 | 657.759 | 54.766 | 481.601 | 61.898 | 650.834 | 74.106 | 583.992 | 67.204 | 426.924 | 57.824 | 184.475 |
| 70.004 | 653.051 | 54.641 | 478.025 | 61.778 | 647.200 | 74.030 | 579.912 | 67.102 | 423.098 | 57.696 | 181.738 |
| 69.916 | 648.399 | 54.516 | 474.496 | 61.657 | 643.644 | 73.954 | 575.933 | 67.001 | 419.354 | 57.567 | 179.060 |
| 69.827 | 643.783 | 54.391 | 471.213 | 61.537 | 640.065 | 73.879 | 571.935 | 66.899 | 415.648 | 57.439 | 176.257 |
| 69.739 | 639.206 | 54.265 | 467.943 | 61.417 | 636.488 | 73.803 | 567.963 | 66.798 | 411.951 | 57.311 | 173.515 |
| 69.650 | 634.637 | 54.140 | 464.814 | 61.296 | 632.885 | 73.728 | 564.070 | 66.696 | 408.233 | 57.182 | 170.749 |
| 69.562 | 630.073 | 54.015 | 461.713 | 61.176 | 629.282 | 73.652 | 559.853 | 66.595 | 404.534 | 57.054 | 167.946 |
| 69.473 | 625.535 | 53.890 | 458.629 | 61.056 | 625.679 | 73.577 | 555.706 | 66.493 | 400.894 | 56.925 | 164.840 |
| 69.385 | 620.947 | 53.765 | 455.546 | 60.936 | 622.076 | 73.501 | 551.768 | 66.391 | 397.325 | 56.797 | 161.778 |
| 69.296 | 616.304 | 53.639 | 452.491 | 60.815 | 618.445 | 73.426 | 547.824 | 66.290 | 393.765 | 56.669 | 158.923 |
| 69.208 | 611.664 | 53.514 | 449.487 | 60.695 | 614.860 | 73.350 | 544.291 | 66.188 | 390.380 | 56.540 | 156.238 |
| 69.119 | 607.023 | 53.389 | 446.493 | 60.575 | 611.285 | 73.275 | 540.777 | 66.087 | 387.045 | 56.412 | 153.449 |
| 69.031 | 602.466 | 53.264 | 443.618 | 60.454 | 607.626 | 73.199 | 537.275 | 65.985 | 383.537 | 56.283 | 150.921 |
| 68.942 | 598.120 | 53.138 | 440.796 | 60.334 | 603.927 | 73.124 | 533.860 | 65.884 | 379.960 | 56.155 | 148.351 |
| 68.854 | 593.807 | 53.013 | 438.123 | 60.214 | 600.316 | 73.048 | 530.438 | 65.782 | 376.336 | 56.026 | 145.750 |
| 68.765 | 589.466 | 52.888 | 435.462 | 60.094 | 596.645 | 72.972 | 526.931 | 65.681 | 372.660 | 55.898 | 143.266 |
| 68.677 | 585.242 | 52.763 | 432.798 | 59.973 | 592.908 | 72.897 | 523.423 | 65.579 | 368.975 | 55.770 | 140.701 |
| 68.588 | 580.865 | 52.638 | 429.800 | 59.853 | 589.216 | 72.821 | 519.895 | 65.478 | 365.345 | 55.641 | 138.048 |
| 68.500 | 576.228 | 52.512 | 426.800 | 59.733 | 585.587 | 72.746 | 516.296 | 65.376 | 361.929 | 55.513 | 135.223 |
| 68.411 | 571.461 | 52.387 | 423.749 | 59.613 | 582.125 | 72.670 | 512.519 | 65.275 | 358.585 | 55.384 | 132.439 |
| 68.323 | 566.673 | 52.262 | 420.639 | 59.492 | 578.806 | 72.595 | 508.649 | 65.173 | 355.323 | 55.256 | 129.674 |
| 68.234 | 561.963 | 52.137 | 417.574 | 59.372 | 575.517 | 72.519 | 504.742 | 65.071 | 352.058 | 55.128 | 127.139 |
| 68.146 | 557.568 | 52.011 | 414.610 | 59.252 | 572.249 | 72.444 | 500.857 | 64.970 | 348.738 | 54.999 | 124.667 |
| 68.057 | 553.342 | 51.886 | 411.689 | 59.131 | 568.942 | 72.368 | 497.096 | 64.868 | 345.367 | 54.871 | 122.371 |
| 67.969 | 549.148 | 51.761 | 408.594 | 59.011 | 565.651 | 72.293 | 493.321 | 64.767 | 342.005 | 54.742 | 120.240 |
| 67.880 | 544.931 | 51.636 | 405.475 | 58.891 | 562.355 | 72.217 | 489.747 | 64.665 | 338.753 | 54.614 | 118.118 |
| 67.792 | 540.737 | 51.511 | 402.363 | 58.771 | 558.971 | 72.142 | 486.322 | 64.564 | 335.487 | 54.486 | 116.032 |
| 67.703 | 536.573 | 51.385 | 399.109 | 58.650 | 555.590 | 72.066 | 482.812 | 64.462 | 332.187 | 54.357 | 113.958 |
| 67.615 | 532.492 | 51.260 | 395.915 | 58.530 | 552.209 | 71.990 | 479.336 | 64.361 | 328.896 | 54.229 | 112.020 |
| 67.527 | 528.431 | 51.135 | 392.722 | 58.410 | 548.793 | 71.915 | 475.851 | 64.259 | 325.645 | 54.100 | 110.134 |
| 67.438 | 524.472 | 51.010 | 389.733 | 58.289 | 545.386 | 71.839 | 472.346 | 64.158 | 322.454 | 53.972 | 108.315 |
| 67.350 | 520.551 | 50.884 | 386.816 | 58.169 | 541.963 | 71.764 | 468.767 | 64.056 | 319.125 | 53.844 | 106.472 |
| 67.261 | 516.478 | 50.759 | 383.727 | 58.049 | 538.556 | 71.688 | 465.152 | 63.955 | 315.755 | 53.715 | 104.587 |
| 67.173 | 512.242 | 50.634 | 380.669 | 57.929 | 535.238 | 71.613 | 461.585 | 63.853 | 312.428 | 53.587 | 102.615 |
| 67.084 | 508.049 | 50.509 | 377.589 | 57.808 | 532.031 | 71.537 | 458.180 | 63.752 | 309.169 | 53.458 | 100.653 |
| 66.996 | 503.818 | 50.384 | 374.549 | 57.688 | 528.844 | 71.462 | 454.822 | 63.650 | 305.873 | 53.330 | 98.719 |
| 66.907 | 499.698 | 50.258 | 371.532 | 57.568 | 525.646 | 71.386 | 451.380 | 63.548 | 302.523 | 53.202 | 96.779 |
| 66.819 | 495.722 | 50.133 | 368.518 | 57.447 | 522.535 | 71.311 | 447.913 | 63.447 | 299.367 | 53.073 | 94.815 |
| 66.730 | 492.014 | 50.008 | 365.971 | 57.327 | 519.431 | 71.235 | 444.466 | 63.345 | 296.272 | 52.945 | 92.808 |
| 66.642 | 488.195 | 49.883 | 363.447 | 57.207 | 516.270 | 71.160 | 440.871 | 63.244 | 293.127 | 52.816 | 90.585 |
| 66.553 | 484.286 | 49.757 | 360.862 | 57.087 | 513.129 | 71.084 | 437.273 | 63.142 | 289.962 | 52.688 | 88.347 |
| 66.465 | 480.377 | 49.632 | 358.231 | 56.966 | 509.866 | 71.008 | 433.664 | 63.041 | 286.841 | 52.560 | 86.166 |
| 66.376 | 476.468 | 49.507 | 355.597 | 56.846 | 506.497 | 70.933 | 430.051 | 62.939 | 283.730 | 52.431 | 84.282 |
| 66.288 | 472.545 | 49.382 | 352.916 | 56.726 | 503.103 | 70.857 | 426.443 | 62.838 | 280.559 | 52.303 | 82.483 |
| 66.199 | 468.406 | 49.257 | 350.164 | 56.605 | 499.700 | 70.782 | 422.825 | 62.736 | 277.436 | 52.174 | 80.762 |
| 66.111 | 464.248 | 49.131 | 347.395 | 56.485 | 496.270 | 70.706 | 418.892 | 62.635 | 274.281 | 52.046 | 79.026 |
| 66.022 | 460.089 | 49.006 | 344.573 | 56.365 | 492.950 | 70.631 | 414.803 | 62.533 | 271.096 | 51.918 | 77.268 |
| 65.934 | 455.740 | 48.881 | 341.790 | 56.245 | 489.879 | 70.555 | 410.610 | 62.432 | 267.831 | 51.789 | 75.555 |
| 65.845 | 451.249 | 48.756 | 338.980 | 56.124 | 486.796 | 70.480 | 406.680 | 62.330 | 264.569 | 51.661 | 73.855 |
| 65.757 | 446.429 | 48.631 | 336.011 | 56.004 | 483.740 | 70.404 | 403.048 | 62.229 | 261.348 | 51.532 | 72.233 |
| 65.668 | 441.610 | 48.505 | 332.866 | 55.884 | 480.746 | 70.329 | 399.616 | 62.127 | 258.240 | 51.404 | 70.580 |
| 65.580 | 437.016 | 48.380 | 329.564 | 55.764 | 477.745 | 70.253 | 396.248 | 62.025 | 255.397 | 51.276 | 68.936 |
| 65.491 | 432.686 | 48.255 | 326.167 | 55.643 | 474.730 | 70.178 | 392.938 | 61.924 | 252.818 | 51.147 | 67.377 |
| 65.403 | 428.431 | 48.130 | 322.810 | 55.523 | 471.707 | 70.102 | 389.416 | 61.822 | 250.302 | 51.019 | 65.828 |
| 65.314 | 424.440 | 48.004 | 319.465 | 55.403 | 468.626 | 70.026 | 385.939 | 61.721 | 247.810 | 50.890 | 64.353 |
| 65.226 | 420.494 | 47.879 | 316.364 | 55.282 | 465.515 | 69.951 | 382.462 | 61.619 | 245.322 | 50.762 | 62.929 |
| 65.137 | 416.621 | 47.754 | 313.441 | 55.162 | 462.557 | 69.875 | 378.997 | 61.518 | 242.750 | 50.634 | 61.594 |
| 65.049 | 412.812 | 47.629 | 310.517 | 55.042 | 459.641 | 69.800 | 375.539 | 61.416 | 240.148 | 50.505 | 60.259 |
| 64.960 | 408.911 | 47.504 | 307.583 | 54.922 | 456.729 | 69.724 | 372.065 | 61.315 | 237.448 | 50.377 | 58.894 |
| 64.872 | 404.825 | 47.378 | 304.776 | 54.801 | 453.812 | 69.649 | 368.618 | 61.213 | 234.724 | 50.248 | 57.569 |
| 64.783 | 400.869 | 47.253 | 301.971 | 54.681 | 450.939 | 69.573 | 365.179 | 61.112 | 231.926 | 50.120 | 56.175 |
| 64.695 | 394.383 | 47.128 | 299.274 | 54.561 | 448.128 | 69.498 | 361.776 | 61.010 | 229.213 | 49.991 | 54.692 |
| 64.606 | 390.613 | 47.003 | 296.637 | 54.440 | 445.256 | 69.422 | 358.441 | 60.909 | 226.494 | 49.863 | 53.178 |
| 64.518 | 386.905 | 46.877 | 294.027 | 54.320 | 442.265 | 69.347 | 354.991 | 60.807 | 223.360 | 49.735 | 51.648 |
| 64.429 | 383.437 | 46.752 | 291.471 | 54.200 | 439.308 | 69.271 | 351.544 | 60.705 | 220.244 | 49.606 | 50.278 |
| 64.341 | 379.936 | 46.627 | 289.034 | 54.080 | 436.375 | 69.196 | 348.102 | 60.604 | 217.509 | 49.478 | 49.090 |
| 64.252 | 376.321 | 46.502 | 286.721 | 53.959 | 433.161 | 69.120 | 344.817 | 60.502 | 214.867 | 49.349 | 47.854 |
| 64.164 | 372.883 | 46.377 | 284.408 | 53.839 | 429.813 | 69.044 | 341.702 | 60.401 | 212.196 | 49.221 | 46.629 |
| 64.075 | 369.614 | 46.251 | 281.981 | 53.719 | 426.559 | 68.969 | 338.672 | 60.299 | 209.529 | 49.093 | 45.368 |
| 63.987 | 366.311 | 46.126 | 279.628 | 53.598 | 423.232 | 68.893 | 335.744 | 60.198 | 206.856 | 48.964 | 44.127 |
| 63.898 | 362.919 | 46.001 | 277.363 | 53.478 | 419.881 | 68.818 | 332.875 | 60.096 | 204.192 | 48.836 | 42.893 |
| 63.810 | 359.527 | 45.876 | 274.980 | 53.358 | 416.730 | 68.742 | 329.981 | 59.995 | 201.495 | 48.707 | 41.675 |
| 63.721 | 356.271 | 45.750 | 272.591 | 53.238 | 413.732 | 68.667 | 327.047 | 59.893 | 198.768 | 48.579 | 40.542 |
| 63.633 | 353.029 | 45.625 | 270.241 | 53.117 | 410.706 | 68.591 | 324.230 | 59.792 | 196.056 | 48.451 | 39.442 |
| 63.544 | 349.463 | 45.500 | 267.820 | 52.997 | 407.669 | 68.516 | 321.448 | 59.690 | 193.422 | 48.322 | 38.288 |
| 63.456 | 345.684 | 45.375 | 265.064 | 52.877 | 404.631 | 68.440 | 318.673 | 59.589 | 190.768 | 48.194 | 37.134 |
| 63.367 | 342.106 | 45.250 | 262.226 | 52.757 | 401.727 | 68.365 | 316.014 | 59.487 | 187.985 | 48.065 | 35.982 |
| 63.279 | 338.528 | 45.124 | 259.420 | 52.636 | 398.871 | 68.289 | 313.287 | 59.386 | 185.143 | 47.937 | 34.870 |
| 63.190 | 334.841 | 44.999 | 256.616 | 52.516 | 396.113 | 68.214 | 310.562 | 59.284 | 182.341 | 47.809 | 33.795 |
| 63.102 | 331.114 | 44.874 | 254.010 | 52.396 | 393.354 | 68.138 | 307.869 | 59.182 | 179.588 | 47.680 | 32.818 |
| 63.013 | 327.522 | 44.749 | 251.444 | 52.275 | 390.508 | 68.063 | 305.189 | 59.081 | 176.965 | 47.552 | 31.862 |
| 62.925 | 324.032 | 44.623 | 248.747 | 52.155 | 387.640 | 67.987 | 302.535 | 58.979 | 174.351 | 47.423 | 30.855 |
| 62.836 | 320.636 | 44.498 | 246.018 | 52.035 | 384.703 | 67.911 | 299.828 | 58.878 | 171.908 | 47.295 | 29.710 |
| 62.748 | 317.293 | 44.373 | 243.331 | 51.915 | 381.938 | 67.836 | 297.037 | 58.776 | 169.441 | 47.167 | 28.871 |
| 62.659 | 314.088 | 44.248 | 240.708 | 51.794 | 379.096 | 67.760 | 294.128 | 58.675 | 166.964 | 47.038 | 28.001 |
| 62.571 | 310.909 | 44.123 | 238.085 | 51.674 | 376.246 | 67.685 | 291.204 | 58.573 | 164.557 | 46.910 | 27.129 |
| 62.482 | 307.555 | 43.997 | 235.516 | 51.554 | 373.440 | 67.609 | 288.252 | 58.472 | 162.240 | 46.781 | 26.284 |
| 62.394 | 304.080 | 43.872 | 233.193 | 51.433 | 370.673 | 67.534 | 285.300 | 58.370 | 159.939 | 46.653 | 25.548 |
| 62.305 | 300.599 | 43.747 | 230.712 | 51.313 | 367.914 | 67.458 | 282.218 | 58.269 | 157.640 | 46.525 | 24.818 |
| 62.217 | 297.013 | 43.622 | 228.145 | 51.193 | 364.763 | 67.383 | 279.135 | 58.167 | 155.415 | 46.396 | 24.100 |
| 62.128 | 293.475 | 43.497 | 225.597 | 51.073 | 361.695 | 67.307 | 276.230 | 58.066 | 153.289 | 46.268 | 23.352 |
| 62.040 | 290.083 | 43.371 | 223.001 | 50.952 | 358.623 | 67.232 | 273.345 | 57.964 | 151.275 | 46.139 | 22.566 |
| 61.951 | 286.728 | 43.246 | 220.354 | 50.832 | 355.492 | 67.156 | 270.489 | 57.862 | 149.220 | 46.011 | 21.735 |
| 61.863 | 283.353 | 43.121 | 217.812 | 50.712 | 352.611 | 67.081 | 267.533 | 57.761 | 147.128 | 45.883 | 20.970 |
| 61.774 | 280.028 | 42.996 | 215.272 | 50.591 | 349.791 | 67.005 | 264.710 | 57.659 | 145.053 | 45.754 | 20.200 |
| 61.686 | 279.036 | 42.870 | 212.646 | 50.471 | 346.971 | 66.929 | 261.887 | 57.558 | 143.020 | 45.626 | 19.423 |
| 61.597 | 274.987 | 42.745 | 209.983 | 50.351 | 344.100 | 66.854 | 259.064 | 57.456 | 140.989 | 45.497 | 18.620 |
| 61.509 | 270.820 | 42.620 | 207.372 | 50.231 | 341.271 | 66.778 | 256.257 | 57.355 | 138.948 | 45.369 | 17.853 |
| 61.420 | 266.763 | 42.495 | 204.771 | 50.110 | 338.641 | 66.703 | 253.552 | 57.253 | 136.911 | 45.241 | 17.243 |
| 61.332 | 262.702 | 42.370 | 202.172 | 49.990 | 335.828 | 66.627 | 250.884 | 57.152 | 134.804 | 45.112 | 16.655 |
| 61.243 | 259.113 | 42.244 | 199.501 | 49.870 | 332.785 | 66.552 | 248.321 | 57.050 | 132.621 | 44.984 | 16.113 |
| 61.155 | 255.518 | 42.119 | 196.963 | 49.750 | 329.718 | 66.476 | 245.846 | 56.949 | 130.265 | 44.855 | 15.516 |
| 61.066 | 251.836 | 41.994 | 194.352 | 49.629 | 326.688 | 66.401 | 243.148 | 56.847 | 127.910 | 44.727 | 14.920 |
| 60.978 | 248.111 | 41.869 | 191.718 | 49.509 | 324.108 | 66.325 | 240.388 | 56.746 | 125.563 | 44.599 | 14.355 |
| 60.889 | 244.426 | 41.743 | 189.053 | 49.389 | 321.644 | 66.250 | 237.463 | 56.644 | 123.167 | 44.470 | 13.815 |
| 60.801 | 245.121 | 41.618 | 186.646 | 49.268 | 319.277 | 66.174 | 234.419 | 56.543 | 120.803 | 44.342 | 13.289 |
| 60.712 | 241.645 | 41.493 | 184.315 | 49.148 | 316.909 | 66.099 | 231.463 | 56.441 | 118.621 | 44.213 | 12.719 |
| 60.624 | 238.319 | 41.368 | 181.984 | 49.028 | 314.495 | 66.023 | 228.571 | 56.339 | 116.417 | 44.085 | 12.202 |
| 60.535 | 234.924 | 41.243 | 179.828 | 48.908 | 311.946 | 65.947 | 225.863 | 56.238 | 114.233 | 43.956 | 11.659 |
| 60.447 | 231.342 | 41.117 | 177.548 | 48.787 | 309.396 | 65.872 | 223.314 | 56.136 | 112.292 | 43.828 | 11.159 |
| 60.358 | 227.753 | 40.992 | 175.232 | 48.667 | 306.855 | 65.796 | 220.714 | 56.035 | 110.408 | 43.700 | 10.698 |
| 60.270 | 224.420 | 40.867 | 172.871 | 48.547 | 304.052 | 65.721 | 218.093 | 55.933 | 108.616 | 43.571 | 10.239 |
| 60.181 | 221.328 | 40.742 | 170.545 | 48.426 | 301.177 | 65.645 | 215.435 | 55.832 | 106.799 | 43.443 | 9.789 |
| 60.093 | 218.254 | 40.616 | 168.371 | 48.306 | 298.506 | 65.570 | 212.732 | 55.730 | 104.955 | 43.314 | 9.355 |
| 60.004 | 215.154 | 40.491 | 166.221 | 48.186 | 295.887 | 65.494 | 210.010 | 55.629 | 103.125 | 43.186 | 8.964 |
| 59.916 | 212.149 | 40.366 | 164.071 | 48.066 | 293.446 | 65.419 | 207.314 | 55.527 | 101.323 | 43.058 | 8.584 |
| 59.827 | 209.331 | 40.241 | 161.822 | 47.945 | 290.960 | 65.343 | 204.556 | 55.426 | 99.499 | 42.929 | 8.211 |
| 59.739 | 206.441 | 40.116 | 159.369 | 47.825 | 288.482 | 65.268 | 201.813 | 55.324 | 97.654 | 42.801 | 7.844 |
| 59.650 | 203.421 | 39.990 | 156.981 | 47.705 | 286.026 | 65.192 | 199.171 | 55.223 | 95.897 | 42.672 | 7.523 |
| 59.562 | 200.448 | 39.865 | 154.592 | 47.584 | 283.680 | 65.117 | 196.417 | 55.121 | 94.253 | 42.544 | 7.228 |
| 59.473 | 197.650 | 39.740 | 152.244 | 47.464 | 281.280 | 65.041 | 193.678 | 55.020 | 92.698 | 42.416 | 6.872 |
| 59.385 | 194.866 | 39.615 | 149.993 | 47.344 | 278.855 | 64.965 | 190.911 | 54.918 | 91.153 | 42.287 | 6.486 |
| 59.296 | 192.122 | 39.489 | 147.792 | 47.224 | 276.220 | 64.890 | 188.170 | 54.816 | 89.625 | 42.159 | 6.207 |
| 59.208 | 189.488 | 39.364 | 145.648 | 47.103 | 273.603 | 64.814 | 185.503 | 54.715 | 88.139 | 42.030 | 6.081 |
| 59.119 | 186.926 | 39.239 | 143.503 | 46.983 | 270.911 | 64.739 | 182.832 | 54.613 | 86.596 | 41.902 | 5.999 |
| 59.031 | 184.448 | 39.114 | 141.353 | 46.863 | 268.096 | 64.663 | 180.187 | 54.512 | 85.018 | 41.774 | 5.857 |
| 58.942 | 181.982 | 38.989 | 139.313 | 46.743 | 265.148 | 64.588 | 177.667 | 54.410 | 83.508 | 41.645 | 5.694 |
| 58.854 | 179.432 | 38.863 | 137.184 | 46.622 | 262.237 | 64.512 | 175.279 | 54.309 | 81.993 | 41.517 | 5.679 |
| 58.765 | 176.775 | 38.738 | 135.068 | 46.502 | 259.446 | 64.437 | 172.940 | 54.207 | 80.439 | 41.388 | 5.575 |
| 58.677 | 173.696 | 38.613 | 132.972 | 46.382 | 256.769 | 64.361 | 170.768 | 54.106 | 78.888 |  |  |
| 58.588 | 170.560 | 38.488 | 131.015 | 46.261 | 254.276 | 64.286 | 168.724 | 54.004 | 77.229 |  |  |
| 58.500 | 166.119 | 38.363 | 129.055 | 46.141 | 251.808 | 64.210 | 166.488 | 53.903 | 75.619 |  |  |
| 58.411 | 163.359 | 38.237 | 127.065 | 46.021 | 249.388 | 64.135 | 164.195 | 53.801 | 74.004 |  |  |
| 58.323 | 160.687 | 38.112 | 125.072 | 45.901 | 246.993 | 64.059 | 161.903 | 53.700 | 72.509 |  |  |
| 58.234 | 158.050 | 37.987 | 123.146 | 45.780 | 244.558 | 63.983 | 159.628 | 53.598 | 71.075 |  |  |
| 58.146 | 155.322 | 37.862 | 121.196 | 45.660 | 242.114 | 63.908 | 157.455 | 53.496 | 69.634 |  |  |
| 58.057 | 152.445 | 37.736 | 119.169 | 45.540 | 239.721 | 63.832 | 154.371 | 53.395 | 68.167 |  |  |
| 57.969 | 149.576 | 37.611 | 117.178 | 45.419 | 237.366 | 63.757 | 152.303 | 53.293 | 66.720 |  |  |
| 57.880 | 147.167 | 37.486 | 115.115 | 45.299 | 235.034 | 63.681 | 150.213 | 53.192 | 65.285 |  |  |
| 57.792 | 145.111 | 37.361 | 113.030 | 45.179 | 232.745 | 63.606 | 148.123 | 53.090 | 63.834 |  |  |
| 57.703 | 143.070 | 37.236 | 110.921 | 45.059 | 230.576 | 63.530 | 146.008 | 52.989 | 62.390 |  |  |
| 57.615 | 141.018 | 37.110 | 108.736 | 44.938 | 228.440 | 63.455 | 143.794 | 52.887 | 61.097 |  |  |
| 57.526 | 138.822 | 36.985 | 106.386 | 44.818 | 226.305 | 63.379 | 141.579 | 52.786 | 59.788 |  |  |
| 57.438 | 136.522 | 36.860 | 104.127 | 44.698 | 224.117 | 63.304 | 139.461 | 52.684 | 58.320 |  |  |
| 57.349 | 134.211 | 36.735 | 102.006 | 44.577 | 221.800 | 63.228 | 137.079 | 52.583 | 56.857 |  |  |
| 57.261 | 131.879 | 36.609 | 100.095 | 44.457 | 219.468 | 63.153 | 134.696 | 52.481 | 55.542 |  |  |
| 57.172 | 129.772 | 36.484 | 98.387 | 44.337 | 217.136 | 63.077 | 132.737 | 52.380 | 54.368 |  |  |
| 57.084 | 127.765 | 36.359 | 96.762 | 44.217 | 214.561 | 63.001 | 130.730 | 52.278 | 53.193 |  |  |
| 56.995 | 125.729 | 36.234 | 95.038 | 44.096 | 211.727 | 62.926 | 128.696 | 52.177 | 52.044 |  |  |
| 56.907 | 123.497 | 36.109 | 93.245 | 43.976 | 208.801 | 62.850 | 126.548 | 52.075 | 50.925 |  |  |
| 56.818 | 121.030 | 35.983 | 91.459 | 43.856 | 206.042 | 62.775 | 124.198 | 51.973 | 49.802 |  |  |
| 56.730 | 118.236 | 35.858 | 89.635 | 43.735 | 203.131 | 62.699 | 121.834 | 51.872 | 48.687 |  |  |
| 56.641 | 115.710 | 35.733 | 87.675 | 43.615 | 200.424 | 62.624 | 119.648 | 51.770 | 47.615 |  |  |
| 56.553 | 113.410 | 35.608 | 85.726 | 43.495 | 197.759 | 62.548 | 117.344 | 51.669 | 46.523 |  |  |
| 56.465 | 110.673 | 35.482 | 83.779 | 43.375 | 195.420 | 62.473 | 114.995 | 51.567 | 45.436 |  |  |
| 56.376 | 107.935 | 35.357 | 81.851 | 43.254 | 192.966 | 62.397 | 112.758 | 51.466 | 44.338 |  |  |
| 56.288 | 105.487 | 35.232 | 79.674 | 43.134 | 190.427 | 62.322 | 110.806 | 51.364 | 43.244 |  |  |
| 56.199 | 103.244 | 35.107 | 77.334 | 43.014 | 187.827 | 62.246 | 108.899 | 51.263 | 42.165 |  |  |
| 56.111 | 101.074 | 34.982 | 75.111 | 42.894 | 185.385 | 62.171 | 107.013 | 51.161 | 41.128 |  |  |
| 56.022 | 98.677 | 34.856 | 73.293 | 42.773 | 183.270 | 62.095 | 105.137 | 51.060 | 40.105 |  |  |
| 55.934 | 96.173 | 34.731 | 71.717 | 42.653 | 181.039 | 62.019 | 103.361 | 50.958 | 39.089 |  |  |
| 55.845 | 93.876 | 34.606 | 70.105 | 42.533 | 178.788 | 61.944 | 101.608 | 50.857 | 38.068 |  |  |
| 55.757 | 91.757 | 34.481 | 68.442 | 42.412 | 176.517 | 61.868 | 99.905 | 50.755 | 37.105 |  |  |
| 55.668 | 89.739 | 34.355 | 66.952 | 42.292 | 174.274 | 61.793 | 98.208 | 50.653 | 36.174 |  |  |
| 55.580 | 88.064 | 34.230 | 65.463 | 42.172 | 172.111 | 61.717 | 96.510 | 50.552 | 35.281 |  |  |
| 55.491 | 86.401 | 34.105 | 63.304 | 42.052 | 170.116 | 61.642 | 94.713 | 50.450 | 34.439 |  |  |
| 55.403 | 84.791 | 33.980 | 61.877 | 41.931 | 168.102 | 61.566 | 92.889 | 50.349 | 33.561 |  |  |
| 55.314 | 83.137 | 33.855 | 60.212 | 41.811 | 166.029 | 61.491 | 91.034 | 50.247 | 32.703 |  |  |
| 55.226 | 81.502 | 33.729 | 58.383 | 41.691 | 163.976 | 61.415 | 89.168 | 50.146 | 31.838 |  |  |
| 55.137 | 79.867 | 33.604 | 56.538 | 41.570 | 161.925 | 61.340 | 87.355 | 50.044 | 30.971 |  |  |
| 55.049 | 78.195 | 33.479 | 54.864 | 41.450 | 159.863 | 61.264 | 85.569 | 49.943 | 30.183 |  |  |
| 54.960 | 76.584 | 33.354 | 53.206 | 41.330 | 157.871 | 61.189 | 83.837 | 49.841 | 29.389 |  |  |
| 54.872 | 75.064 | 33.229 | 51.521 | 41.210 | 155.951 | 61.113 | 82.198 | 49.740 | 28.613 |  |  |
| 54.783 | 73.525 | 33.103 | 49.893 | 41.089 | 154.085 | 61.038 | 80.588 | 49.638 | 27.905 |  |  |
| 54.695 | 71.958 | 32.978 | 48.006 | 40.969 | 152.218 | 60.962 | 78.985 | 49.537 | 27.196 |  |  |
| 54.606 | 70.160 | 32.853 | 46.410 | 40.849 | 150.352 | 60.886 | 77.259 | 49.435 | 26.491 |  |  |
| 54.518 | 67.865 | 32.728 | 45.193 | 40.728 | 148.485 | 60.811 | 75.145 | 49.334 | 25.810 |  |  |
| 54.429 | 66.053 | 32.602 | 43.981 | 40.608 | 146.259 | 60.735 | 73.015 | 49.232 | 25.171 |  |  |
| 54.341 | 64.231 | 32.477 | 42.804 | 40.488 | 143.763 | 60.660 | 70.879 | 49.130 | 24.542 |  |  |
| 54.252 | 62.472 | 32.352 | 41.577 | 40.368 | 141.020 | 60.584 | 68.993 | 49.029 | 23.907 |  |  |
| 54.164 | 60.981 | 32.227 | 40.344 | 40.247 | 138.505 | 60.509 | 67.596 | 48.927 | 23.221 |  |  |
| 54.075 | 59.287 | 32.102 | 39.056 | 40.127 | 136.319 | 60.433 | 66.032 | 48.826 | 22.559 |  |  |
| 53.987 | 57.429 | 31.976 | 37.844 | 40.007 | 134.224 | 60.358 | 64.111 | 48.724 | 21.894 |  |  |
| 53.898 | 55.817 | 31.851 | 36.567 | 39.887 | 132.233 | 60.282 | 62.026 | 48.623 | 21.229 |  |  |
| 53.810 | 54.239 | 31.726 | 35.205 | 39.766 | 130.243 | 60.207 | 60.160 | 48.521 | 20.586 |  |  |
| 53.721 | 52.666 | 31.601 | 33.704 | 39.646 | 127.835 | 60.131 | 58.258 | 48.420 | 20.009 |  |  |
| 53.633 | 51.281 | 31.475 | 32.376 | 39.526 | 125.100 | 60.056 | 56.325 | 48.318 | 19.398 |  |  |
| 53.544 | 49.776 | 31.350 | 31.203 | 39.405 | 123.235 | 59.980 | 54.424 | 48.217 | 18.804 |  |  |
| 53.456 | 47.831 | 31.225 | 30.057 | 39.285 | 121.327 | 59.904 | 52.805 | 48.115 | 18.234 |  |  |
| 53.367 | 46.498 | 31.100 | 29.111 | 39.165 | 119.383 | 59.829 | 51.495 | 48.014 | 17.669 |  |  |
| 53.279 | 45.027 | 30.975 | 28.133 | 39.045 | 117.453 | 59.753 | 50.223 | 47.912 | 17.162 |  |  |
| 53.190 | 43.314 | 30.849 | 27.017 | 38.924 | 115.581 | 59.678 | 48.963 | 47.810 | 16.638 |  |  |
| 53.102 | 41.801 | 30.724 | 25.908 | 38.804 | 113.996 | 59.602 | 47.704 | 47.709 | 16.165 |  |  |
| 53.013 | 40.037 | 30.599 | 24.741 | 38.684 | 112.522 | 59.527 | 46.324 | 47.607 | 15.706 |  |  |
| 52.925 | 38.883 | 30.474 | 23.502 | 38.563 | 110.983 | 59.451 | 44.892 | 47.506 | 15.237 |  |  |
| 52.836 | 37.759 | 30.348 | 22.277 | 38.443 | 109.285 | 59.376 | 43.443 | 47.404 | 14.708 |  |  |
| 52.748 | 36.577 | 30.223 | 21.126 | 38.323 | 107.550 | 59.300 | 42.124 | 47.303 | 14.204 |  |  |
| 52.659 | 35.396 | 30.098 | 20.180 | 38.203 | 105.762 | 59.225 | 40.787 | 47.201 | 13.706 |  |  |
| 52.571 | 33.950 | 29.973 | 19.272 | 38.082 | 103.942 | 59.149 | 39.313 | 47.100 | 13.219 |  |  |
| 52.482 | 32.162 | 29.848 | 18.037 | 37.962 | 102.105 | 59.074 | 37.766 | 46.998 | 12.741 |  |  |
| 52.394 | 31.072 | 29.722 | 17.111 | 37.842 | 100.207 | 58.998 | 36.284 | 46.897 | 12.343 |  |  |
| 52.305 | 29.913 | 29.597 | 16.110 | 37.721 | 98.203 | 58.922 | 34.932 | 46.795 | 11.962 |  |  |
| 52.217 | 28.723 | 29.472 | 15.368 | 37.601 | 96.202 | 58.847 | 33.606 | 46.694 | 11.609 |  |  |
| 52.128 | 27.610 | 29.347 | 14.296 | 37.481 | 94.251 | 58.771 | 32.304 | 46.592 | 11.269 |  |  |
| 52.040 | 26.629 | 29.222 | 13.322 | 37.361 | 92.463 | 58.696 | 30.958 | 46.491 | 10.934 |  |  |
| 51.951 | 25.665 | 29.096 | 12.349 | 37.240 | 90.695 | 58.620 | 29.752 | 46.389 | 10.633 |  |  |
| 51.863 | 24.668 | 28.971 | 11.756 | 37.120 | 88.966 | 58.545 | 28.587 | 46.287 | 10.120 |  |  |
| 51.774 | 23.767 | 28.846 | 11.221 | 37.000 | 87.228 | 58.469 | 27.456 | 46.186 | 9.831 |  |  |
| 51.686 | 22.739 | 28.721 | 10.675 | 36.880 | 85.494 | 58.394 | 26.280 | 46.084 | 9.561 |  |  |
| 51.597 | 21.882 | 28.595 | 9.831 | 36.759 | 83.735 | 58.318 | 25.100 | 45.983 | 9.298 |  |  |
| 51.509 | 21.012 | 28.470 | 9.401 | 36.639 | 81.972 | 58.243 | 23.974 | 45.881 | 9.039 |  |  |
| 51.420 | 20.060 | 28.345 | 8.800 | 36.519 | 80.213 | 58.167 | 22.946 | 45.780 | 8.785 |  |  |
| 51.332 | 19.121 | 28.220 | 8.006 | 36.398 | 78.644 | 58.092 | 22.025 | 45.678 | 8.537 |  |  |
| 51.243 | 18.164 | 28.095 | 7.416 | 36.278 | 76.887 | 58.016 | 21.013 | 45.577 | 8.305 |  |  |
| 51.155 | 17.242 | 27.969 | 6.927 | 36.158 | 75.139 | 57.940 | 19.936 | 45.475 | 8.112 |  |  |
| 51.066 | 16.359 | 27.844 | 6.260 | 36.038 | 73.357 | 57.865 | 18.980 | 45.374 | 7.821 |  |  |
| 50.978 | 15.505 | 27.719 | 5.717 | 35.917 | 71.484 | 57.789 | 18.099 | 45.272 | 7.551 |  |  |
| 50.889 | 14.689 | 27.594 | 5.288 | 35.797 | 69.781 | 57.714 | 17.229 | 45.171 | 7.294 |  |  |
| 50.801 | 13.995 | 27.468 | 4.807 | 35.677 | 68.137 | 57.638 | 16.391 | 45.069 | 7.032 |  |  |
| 50.712 | 13.177 | 27.343 | 4.509 | 35.556 | 66.243 | 57.563 | 15.273 | 44.968 | 6.791 |  |  |
| 50.624 | 12.735 | 27.218 | 4.268 | 35.436 | 64.294 | 57.487 | 14.331 | 44.866 | 6.594 |  |  |
| 50.535 | 12.179 | 27.093 | 3.943 | 35.316 | 62.457 | 57.412 | 13.364 | 44.764 | 6.369 |  |  |
| 50.447 | 11.633 | 26.968 | 3.626 | 35.196 | 60.928 | 57.336 | 12.298 | 44.663 | 6.137 |  |  |
| 50.358 | 11.122 | 26.842 | 3.275 | 35.075 | 59.425 | 57.261 | 11.277 | 44.561 | 5.947 |  |  |
| 50.270 | 10.636 | 26.717 | 2.987 | 34.955 | 58.110 | 57.185 | 10.447 | 44.460 | 5.741 |  |  |
| 50.181 | 10.009 | 26.592 | 2.678 | 34.835 | 56.864 | 57.110 | 9.803 | 44.358 | 5.537 |  |  |
| 50.093 | 9.362 | 26.467 | 2.511 | 34.714 | 55.594 | 57.034 | 9.164 | 44.257 | 5.359 |  |  |
| 50.004 | 8.755 | 26.341 | 2.358 | 34.594 | 54.319 | 56.958 | 8.399 | 44.155 | 5.224 |  |  |
| 49.916 | 8.325 | 26.216 | 2.074 | 34.474 | 53.060 | 56.883 | 7.602 | 44.054 | 5.099 |  |  |
| 49.827 | 7.915 | 26.091 | 2.037 | 34.354 | 51.697 | 56.807 | 6.962 | 43.952 | 4.951 |  |  |
| 49.739 | 7.535 | 25.966 | 1.939 | 34.233 | 50.295 | 56.732 | 6.398 | 43.851 | 4.823 |  |  |
| 49.650 | 7.155 | 25.841 | 1.903 | 34.113 | 48.805 | 56.656 | 5.841 | 43.749 | 4.687 |  |  |
| 49.562 | 6.774 | 25.715 | 1.913 | 33.993 | 47.228 | 56.581 | 5.329 | 43.648 | 4.566 |  |  |
| 49.473 | 6.408 | 25.590 | 1.836 | 33.872 | 45.760 | 56.505 | 4.792 | 43.546 | 4.450 |  |  |
| 49.385 | 6.045 | 25.465 | 1.627 | 33.752 | 44.334 | 56.430 | 4.255 | 43.444 | 4.338 |  |  |
| 49.296 | 5.679 | 25.340 | 1.552 | 33.632 | 42.881 | 56.354 | 3.781 | 43.343 | 4.233 |  |  |
| 49.208 | 5.256 | 25.214 | 1.719 | 33.512 | 41.579 | 56.279 | 3.393 | 43.241 | 4.130 |  |  |
| 49.119 | 4.843 | 25.089 | 1.595 | 33.391 | 40.258 | 56.203 | 3.082 | 43.140 | 3.997 |  |  |
| 49.031 | 4.499 | 24.964 | 1.480 | 33.271 | 38.837 | 56.128 | 2.553 | 43.038 | 3.869 |  |  |
| 48.942 | 4.055 | 24.839 | 1.488 | 33.151 | 37.521 | 56.052 | 2.182 | 42.937 | 3.748 |  |  |
| 48.854 | 3.680 | 24.714 | 1.518 | 33.031 | 36.353 | 55.976 | 1.849 | 42.835 | 3.662 |  |  |
| 48.765 | 3.309 | 24.588 | 1.803 | 32.910 | 35.295 | 55.901 | 1.573 | 42.734 | 3.574 |  |  |
| 48.677 | 2.932 | 24.463 | 1.828 | 32.790 | 34.283 | 55.825 | 1.244 | 42.632 | 3.502 |  |  |
| 48.588 | 2.568 | 24.338 | 1.868 | 32.670 | 33.167 | 55.750 | 0.924 | 42.531 | 3.429 |  |  |
| 48.500 | 2.246 | 24.213 | 2.068 | 32.549 | 32.073 | 55.674 | 0.582 | 42.429 | 3.364 |  |  |
| 48.411 | 1.904 | 24.088 | 2.156 | 32.429 | 30.927 | 55.599 | 0.317 | 42.328 | 3.290 |  |  |
| 48.323 | 1.616 | 23.962 | 2.304 | 32.309 | 29.699 | 55.523 | 0.123 | 42.226 | 3.216 |  |  |
| 48.234 | 1.438 | 23.837 | 2.432 | 32.189 | 28.566 | 55.448 | -0.005 | 42.125 | 3.136 |  |  |
| 48.146 | 1.267 | 23.712 | 2.532 | 32.068 | 27.474 | 55.372 | -0.132 | 42.023 | 3.060 |  |  |
| 48.057 | 1.078 | 23.587 | 2.633 | 31.948 | 26.560 | 55.297 | -0.215 | 41.921 | 2.980 |  |  |
| 47.969 | 0.921 | 23.461 | 2.733 | 31.828 | 25.664 | 55.221 | -0.366 | 41.820 | 2.890 |  |  |
| 47.880 | 0.830 | 23.336 | 2.834 | 31.707 | 24.776 | 55.146 | -0.267 | 41.718 | 2.795 |  |  |
| 47.792 | 0.781 | 23.211 | 2.934 | 31.587 | 23.943 | 55.070 | -0.362 | 41.617 | 2.713 |  |  |
| 47.703 | 0.624 | 23.086 | 3.035 | 31.467 | 23.067 | 54.994 | -0.418 | 41.515 | 2.629 |  |  |
| 47.615 | 0.535 | 22.961 | 3.135 | 31.347 | 22.229 | 54.919 | -0.451 | 41.414 | 2.545 |  |  |
| 47.526 | 0.277 | 22.835 | 3.236 | 31.226 | 21.313 | 54.843 | -0.485 | 41.312 | 2.470 |  |  |
| 47.438 | 0.115 |  |  | 31.106 | 20.420 | 54.768 | -0.484 | 41.211 | 2.395 |  |  |
| 47.349 | 0.080 |  |  | 30.986 | 19.243 | 54.692 | -0.490 | 41.109 | 2.325 |  |  |
| 47.261 | 0.009 |  |  | 30.865 | 18.244 | 54.617 | -0.477 | 41.008 | 2.255 |  |  |
| 47.172 | -0.030 |  |  | 30.745 | 17.234 | 54.541 | -0.464 | 40.906 | 2.184 |  |  |
| 47.084 | -0.079 |  |  | 30.625 | 16.153 | 54.466 | -0.482 | 40.805 | 2.108 |  |  |
| 46.995 | -0.128 |  |  | 30.505 | 15.082 | 54.390 | -0.503 | 40.703 | 2.045 |  |  |
| 46.907 | -0.191 |  |  | 30.384 | 14.273 | 54.315 | -0.609 | 40.601 | 1.999 |  |  |
| 46.818 | -0.221 |  |  | 30.264 | 13.521 | 54.239 | -0.785 | 40.500 | 1.944 |  |  |
| 46.730 | -0.204 |  |  | 30.144 | 12.730 | 54.164 | -0.934 | 40.398 | 1.897 |  |  |
| 46.641 | -0.205 |  |  | 30.024 | 12.135 | 54.088 | -1.081 | 40.297 | 1.849 |  |  |
| 46.553 | -0.194 |  |  | 29.903 | 11.554 | 54.013 | -1.253 | 40.195 | 1.805 |  |  |
| 46.464 | 0.046 |  |  | 29.783 | 10.978 | 53.937 | -1.411 | 40.094 | 1.747 |  |  |
| 46.376 | 0.092 |  |  | 29.663 | 10.093 | 53.861 | -1.586 | 39.992 | 1.694 |  |  |
| 46.287 | 0.059 |  |  | 29.542 | 9.372 | 53.786 | -1.751 | 39.891 | 1.653 |  |  |
| 46.199 | 0.026 |  |  | 29.422 | 8.131 | 53.710 | -1.914 | 39.789 | 1.626 |  |  |
| 46.110 | 0.122 |  |  | 29.302 | 7.397 | 53.635 | -2.070 | 39.688 | 1.608 |  |  |
| 46.022 | 0.141 |  |  | 29.182 | 7.034 | 53.559 | -2.223 | 39.586 | 1.594 |  |  |
| 45.933 | 0.193 |  |  | 29.061 | 6.344 | 53.484 | -2.380 | 39.485 | 1.582 |  |  |
| 45.845 | 0.245 |  |  | 28.941 | 5.606 | 53.408 | -2.544 | 39.383 | 1.575 |  |  |
| 45.756 | 0.293 |  |  | 28.821 | 4.885 | 53.333 | -2.712 | 39.282 | 1.586 |  |  |
| 45.668 | 0.352 |  |  | 28.700 | 4.151 | 53.257 | -2.885 | 39.180 | 1.578 |  |  |
| 45.580 | 0.399 |  |  | 28.580 | 3.439 | 53.182 | -3.049 | 39.078 | 1.574 |  |  |
| 45.491 | 0.462 |  |  | 28.460 | 2.884 | 53.106 | -3.216 | 38.977 | 1.567 |  |  |
| 45.403 | 0.516 |  |  | 28.340 | 2.400 | 53.031 | -3.379 | 38.875 | 1.593 |  |  |
| 45.314 | 0.574 |  |  | 28.219 | 1.872 | 52.955 | -3.542 | 38.774 | 1.619 |  |  |
| 45.226 | 0.629 |  |  | 28.099 | 1.326 | 52.879 | -3.709 | 38.672 | 1.648 |  |  |
| 45.137 | 0.688 |  |  | 27.979 | 0.877 | 52.804 | -3.875 | 38.571 | 1.689 |  |  |
| 45.049 | 0.739 |  |  | 27.858 | 0.448 |  |  | 38.469 | 1.726 |  |  |
| 44.960 | 0.793 |  |  | 27.738 | 0.066 |  |  | 38.368 | 1.769 |  |  |
|  |  |  |  | 27.618 | -0.381 |  |  | 38.266 | 1.802 |  |  |
|  |  |  |  | 27.498 | -0.792 |  |  | 38.165 | 1.839 |  |  |
|  |  |  |  | 27.377 | -0.916 |  |  | 38.063 | 1.875 |  |  |
|  |  |  |  | 27.257 | -1.189 |  |  | 37.962 | 1.919 |  |  |
|  |  |  |  | 27.137 | -1.416 |  |  | 37.860 | 1.966 |  |  |
|  |  |  |  | 27.017 | -1.650 |  |  | 37.759 | 2.005 |  |  |
|  |  |  |  | 26.896 | -1.850 |  |  | 37.657 | 2.044 |  |  |
|  |  |  |  | 26.776 | -2.201 |  |  | 37.555 | 2.087 |  |  |
|  |  |  |  | 26.656 | -2.414 |  |  | 37.454 | 2.122 |  |  |
|  |  |  |  | 26.535 | -2.778 |  |  | 37.352 | 2.166 |  |  |
|  |  |  |  | 26.415 | -2.818 |  |  | 37.251 | 2.208 |  |  |
|  |  |  |  | 26.295 | -2.884 |  |  | 37.149 | 2.246 |  |  |
|  |  |  |  | 26.175 | -2.943 |  |  | 37.048 | 2.282 |  |  |
|  |  |  |  | 26.054 | -3.001 |  |  | 36.946 | 2.318 |  |  |
|  |  |  |  | 25.934 | -2.916 |  |  | 36.845 | 2.354 |  |  |
|  |  |  |  | 25.814 | -3.012 |  |  | 36.743 | 2.404 |  |  |

Table 4. Raw data of the silicon coating (see Fig. 4).

| silicon | | | | | |
| --- | --- | --- | --- | --- | --- |
| as-coated | | 10 h | | 100 h | |
| E_r_ (GPa) | H (GPa) | E_r_ (GPa) | H (GPa) | E_r_ (GPa) | H (GPa) |
| 115.86337 | 7.79262 | 141.12552 | 9.17164 | 167.83901 | 9.34245 |
| 132.01941 | 8.27063 | 151.61410 | 9.29607 | 171.09261 | 10.02209 |
| 141.44223 | 8.33438 | 159.97905 | 9.82198 | 193.05238 | 10.90853 |
| 135.21584 | 8.92391 | 155.90578 | 9.76614 | 190.77161 | 10.52207 |
| 128.30752 | 8.06296 | 189.41248 | 10.32983 | 180.22349 | 9.87046 |
| 123.46994 | 7.95759 | 218.07020 | 11.49881 | 196.64122 | 10.81920 |
| 125.44837 | 8.42332 | 185.67737 | 9.61788 | 192.07751 | 10.36244 |
| 118.25812 | 8.02244 | 175.97168 | 10.18267 | 181.67868 | 9.47271 |
| 111.21479 | 7.20024 | 181.50548 | 10.41557 | 198.57655 | 10.95143 |
| 135.37293 | 8.32333 | 190.93893 | 10.22320 | 210.25888 | 11.40171 |

Table 5. Raw data of the 36 mol% HfO_2_-doped silicon coating (see Fig. 4).

| 36 mol% HfO_2_-doped silicon | | | | | |
| --- | --- | --- | --- | --- | --- |
| as-coated | | 10 h | | 100 h | |
| E_r_ (GPa) | H (GPa) | E_r_ (GPa) | H (GPa) | E_r_ (GPa) | H (GPa) |
| 112.40762 | 6.18539 | 109.81473 | 6.12188 | 140.55182 | 9.23777 |
| 122.14612 | 6.64719 | 102.90394 | 6.37184 | 140.61553 | 8.06262 |
| 123.37931 | 6.60987 | 113.48205 | 7.18728 | 148.65996 | 9.15573 |
| 129.10326 | 6.73312 | 102.43867 | 5.73937 | 152.58144 | 8.90324 |
| 121.58088 | 6.61760 | 104.85219 | 6.34267 | 138.19811 | 7.87415 |
| 119.85768 | 6.42967 | 129.21715 | 7.48987 | 133.06002 | 8.02814 |
| 118.42288 | 6.72870 | 123.12733 | 7.41805 | 138.79758 | 8.09650 |
| 121.88119 | 6.50184 | 120.21241 | 7.22610 | 137.27744 | 8.23965 |
| 123.11569 | 6.72442 | 89.09335 | 5.78327 | 143.62630 | 7.91153 |
| 120.75882 | 6.55273 | 97.22784 | 5.36168 | 156.30666 | 8.85277 |

Table 6. Raw data of Fig. 5.

| silicon (100 h) | | 36 mol% HfO_2_-doped silicon (100 h) | | 60 mol% HfO_2_-doped silicon (100 h) | |
| --- | --- | --- | --- | --- | --- |
| depth (nm) | load (µN) | depth (nm) | load (µN) | depth (nm) | load (µN) |
| -9.875 | 1.540 | -9.920 | 5.293 | -5.296 | 2.135 |
| -9.701 | 1.470 | -9.747 | 4.884 | -5.128 | 2.103 |
| -9.527 | 1.409 | -9.575 | 4.478 | -4.960 | 1.969 |
| -9.353 | 1.349 | -9.402 | 4.081 | -4.793 | 1.869 |
| -9.179 | 1.283 | -9.229 | 3.672 | -4.625 | 1.881 |
| -9.005 | 1.259 | -9.057 | 3.264 | -4.457 | 1.766 |
| -8.831 | 1.206 | -8.884 | 2.875 | -4.290 | 1.700 |
| -8.657 | 1.106 | -8.711 | 2.458 | -4.122 | 1.618 |
| -8.483 | 1.099 | -8.539 | 2.074 | -3.954 | 1.554 |
| -8.309 | 1.019 | -8.366 | 1.694 | -3.787 | 1.406 |
| -8.135 | 0.991 | -8.193 | 1.328 | -3.619 | 1.301 |
| -7.961 | 0.922 | -8.020 | 0.895 | -3.451 | 1.207 |
| -7.787 | 0.840 | -7.848 | 0.555 | -3.283 | 1.145 |
| -7.613 | 0.839 | -7.675 | 0.474 | -3.116 | 0.964 |
| -7.439 | 0.732 | -7.502 | 0.462 | -2.948 | 1.031 |
| -7.264 | 0.730 | -7.330 | 0.434 | -2.780 | 0.911 |
| -7.090 | 0.621 | -7.157 | 0.468 | -2.613 | 0.732 |
| -6.916 | 0.625 | -6.984 | 0.393 | -2.445 | 0.752 |
| -6.742 | 0.613 | -6.811 | 0.450 | -2.277 | 0.642 |
| -6.568 | 0.543 | -6.639 | 0.337 | -2.109 | 0.531 |
| -6.394 | 0.455 | -6.466 | 0.227 | -1.942 | 0.399 |
| -6.220 | 0.486 | -6.293 | 0.195 | -1.774 | 0.476 |
| -6.046 | 0.425 | -6.121 | 0.172 | -1.606 | 0.437 |
| -5.872 | 0.412 | -5.948 | 0.030 | -1.439 | 0.269 |
| -5.698 | 0.359 | -5.775 | 0.013 | -1.271 | 0.293 |
| -5.524 | 0.404 | -5.603 | -0.048 | -1.103 | 0.113 |
| -5.350 | 0.433 | -5.430 | -0.107 | -0.935 | 0.092 |
| -5.176 | 0.401 | -5.257 | -0.226 | -0.768 | -0.108 |
| -5.002 | 0.440 | -5.084 | -0.224 | -0.600 | 0.058 |
| -4.828 | 0.387 | -4.912 | -0.265 | -0.432 | -0.025 |
| -4.654 | 0.275 | -4.739 | -0.327 | -0.265 | 0.009 |
| -4.480 | 0.350 | -4.566 | -0.378 | -0.097 | -0.201 |
| -4.306 | 0.335 | -4.394 | -0.505 | 0.071 | -0.406 |
| -4.132 | 0.233 | -4.221 | -0.479 | 0.238 | 0.136 |
| -3.958 | 0.231 | -4.048 | -0.678 | 0.406 | 0.646 |
| -3.784 | 0.205 | -3.875 | -0.686 | 0.574 | 0.308 |
| -3.609 | 0.225 | -3.703 | -0.771 | 0.742 | 0.864 |
| -3.435 | 0.238 | -3.530 | -0.752 | 0.909 | 0.949 |
| -3.261 | 0.218 | -3.357 | -0.663 | 1.077 | 1.694 |
| -3.087 | 0.199 | -3.185 | -0.524 | 1.245 | 2.813 |
| -2.913 | 0.120 | -3.012 | -0.560 | 1.412 | 2.559 |
| -2.739 | 0.110 | -2.839 | -0.548 | 1.580 | 3.256 |
| -2.565 | 0.186 | -2.667 | -0.527 | 1.748 | 3.663 |
| -2.391 | 0.069 | -2.494 | -0.371 | 1.916 | 5.964 |
| -2.217 | -0.093 | -2.321 | -0.276 | 2.083 | 4.827 |
| -2.043 | 0.018 | -2.148 | -0.217 | 2.251 | 4.866 |
| -1.869 | -0.012 | -1.976 | -0.117 | 2.419 | 6.016 |
| -1.695 | -0.091 | -1.803 | -0.134 | 2.586 | 8.067 |
| -1.521 | -0.061 | -1.630 | -0.033 | 2.754 | 9.751 |
| -1.347 | -0.050 | -1.458 | -0.016 | 2.922 | 9.265 |
| -1.173 | -0.033 | -1.285 | -0.048 | 3.090 | 11.327 |
| -0.999 | -0.088 | -1.112 | 0.056 | 3.257 | 12.863 |
| -0.825 | -0.140 | -0.939 | -0.057 | 3.425 | 13.320 |
| -0.651 | -0.164 | -0.767 | 0.273 | 3.593 | 13.481 |
| -0.477 | 0.346 | -0.594 | 0.314 | 3.760 | 14.772 |
| -0.303 | 0.003 | -0.421 | 0.419 | 3.928 | 17.405 |
| -0.129 | 0.797 | -0.249 | 0.766 | 4.096 | 19.568 |
| 0.046 | 0.833 | -0.076 | 0.092 | 4.264 | 20.236 |
| 0.220 | 0.918 | 0.097 | 1.379 | 4.431 | 21.294 |
| 0.394 | 1.835 | 0.269 | 1.585 | 4.599 | 23.001 |
| 0.568 | 3.873 | 0.442 | 1.752 | 4.767 | 23.859 |
| 0.742 | 4.469 | 0.615 | 2.127 | 4.934 | 25.163 |
| 0.916 | 4.485 | 0.788 | 3.270 | 5.102 | 26.626 |
| 1.090 | 5.264 | 0.960 | 3.486 | 5.270 | 27.460 |
| 1.264 | 7.131 | 1.133 | 4.508 | 5.437 | 28.724 |
| 1.438 | 7.829 | 1.306 | 4.958 | 5.605 | 29.776 |
| 1.612 | 12.204 | 1.478 | 6.283 | 5.773 | 30.169 |
| 1.786 | 10.050 | 1.651 | 7.234 | 5.941 | 31.060 |
| 1.960 | 13.162 | 1.824 | 7.303 | 6.108 | 32.568 |
| 2.134 | 11.692 | 1.997 | 7.352 | 6.276 | 34.659 |
| 2.308 | 13.492 | 2.169 | 9.219 | 6.444 | 35.868 |
| 2.482 | 17.791 | 2.342 | 10.656 | 6.611 | 37.448 |
| 2.656 | 18.154 | 2.515 | 11.948 | 6.779 | 39.118 |
| 2.830 | 23.643 | 2.687 | 13.134 | 6.947 | 40.650 |
| 3.004 | 25.305 | 2.860 | 14.709 | 7.115 | 41.583 |
| 3.178 | 26.657 | 3.033 | 15.698 | 7.282 | 42.630 |
| 3.352 | 28.324 | 3.205 | 16.880 | 7.450 | 44.760 |
| 3.526 | 32.536 | 3.378 | 18.097 | 7.618 | 46.284 |
| 3.701 | 34.748 | 3.551 | 19.461 | 7.785 | 47.539 |
| 3.875 | 35.905 | 3.724 | 20.786 | 7.953 | 48.761 |
| 4.049 | 37.676 | 3.896 | 21.253 | 8.121 | 50.086 |
| 4.223 | 39.424 | 4.069 | 22.242 | 8.289 | 51.839 |
| 4.397 | 42.489 | 4.242 | 24.676 | 8.456 | 52.525 |
| 4.571 | 46.334 | 4.414 | 26.315 | 8.624 | 55.081 |
| 4.745 | 48.482 | 4.587 | 27.691 | 8.792 | 56.148 |
| 4.919 | 50.448 | 4.760 | 28.823 | 8.959 | 57.270 |
| 5.093 | 52.425 | 4.933 | 30.757 | 9.127 | 58.179 |
| 5.267 | 54.080 | 5.105 | 32.503 | 9.295 | 59.624 |
| 5.441 | 57.254 | 5.278 | 34.461 | 9.462 | 60.612 |
| 5.615 | 59.948 | 5.451 | 36.227 | 9.630 | 61.826 |
| 5.789 | 62.335 | 5.623 | 38.374 | 9.798 | 62.946 |
| 5.963 | 65.168 | 5.796 | 39.771 | 9.966 | 63.777 |
| 6.137 | 66.200 | 5.969 | 41.196 | 10.133 | 64.745 |
| 6.311 | 68.847 | 6.141 | 44.429 | 10.301 | 67.748 |
| 6.485 | 71.425 | 6.314 | 45.831 | 10.469 | 69.915 |
| 6.659 | 74.981 | 6.487 | 47.926 | 10.636 | 74.103 |
| 6.833 | 78.043 | 6.660 | 50.281 | 10.804 | 73.975 |
| 7.007 | 81.521 | 6.832 | 51.872 | 10.972 | 74.175 |
| 7.181 | 83.622 | 7.005 | 53.609 | 11.140 | 79.376 |
| 7.356 | 86.989 | 7.178 | 56.688 | 11.307 | 79.588 |
| 7.530 | 90.375 | 7.350 | 58.306 | 11.475 | 84.175 |
| 7.704 | 95.144 | 7.523 | 60.557 | 11.643 | 85.900 |
| 7.878 | 96.387 | 7.696 | 62.858 | 11.810 | 87.240 |
| 8.052 | 97.902 | 7.869 | 65.334 | 11.978 | 88.704 |
| 8.226 | 101.371 | 8.041 | 67.283 | 12.146 | 90.142 |
| 8.400 | 105.235 | 8.214 | 68.834 | 12.314 | 93.438 |
| 8.574 | 109.821 | 8.387 | 70.009 | 12.481 | 95.008 |
| 8.748 | 112.520 | 8.559 | 72.490 | 12.649 | 96.497 |
| 8.922 | 115.030 | 8.732 | 74.326 | 12.817 | 98.354 |
| 9.096 | 117.719 | 8.905 | 76.877 | 12.984 | 100.452 |
| 9.270 | 121.279 | 9.077 | 79.576 | 13.152 | 101.902 |
| 9.444 | 126.415 | 9.250 | 81.262 | 13.320 | 104.366 |
| 9.618 | 129.045 | 9.423 | 83.076 | 13.487 | 106.258 |
| 9.792 | 133.693 | 9.596 | 84.325 | 13.655 | 107.383 |
| 9.966 | 136.888 | 9.768 | 86.386 | 13.823 | 108.482 |
| 10.140 | 140.055 | 9.941 | 88.438 | 13.991 | 109.582 |
| 10.314 | 146.993 | 10.114 | 90.862 | 14.158 | 112.030 |
| 10.488 | 151.651 | 10.286 | 93.662 | 14.326 | 113.569 |
| 10.662 | 154.456 | 10.459 | 96.453 | 14.494 | 115.428 |
| 10.836 | 157.481 | 10.632 | 99.065 | 14.661 | 117.500 |
| 11.011 | 161.732 | 10.805 | 101.057 | 14.829 | 118.952 |
| 11.185 | 164.721 | 10.977 | 102.343 | 14.997 | 121.957 |
| 11.359 | 168.757 | 11.150 | 104.812 | 15.165 | 124.981 |
| 11.533 | 173.215 | 11.323 | 107.223 | 15.332 | 125.943 |
| 11.707 | 177.387 | 11.495 | 110.075 | 15.500 | 128.180 |
| 11.881 | 180.725 | 11.668 | 112.797 | 15.668 | 130.407 |
| 12.055 | 184.198 | 11.841 | 115.058 | 15.835 | 132.842 |
| 12.229 | 188.328 | 12.013 | 117.377 | 16.003 | 134.330 |
| 12.403 | 191.895 | 12.186 | 119.705 | 16.171 | 135.625 |
| 12.577 | 195.042 | 12.359 | 122.156 | 16.339 | 136.782 |
| 12.751 | 198.671 | 12.532 | 125.281 | 16.506 | 139.371 |
| 12.925 | 205.325 | 12.704 | 128.091 | 16.674 | 141.414 |
| 13.099 | 208.210 | 12.877 | 130.046 | 16.842 | 143.948 |
| 13.273 | 209.635 | 13.050 | 132.851 | 17.009 | 145.201 |
| 13.447 | 215.126 | 13.222 | 135.349 | 17.177 | 147.553 |
| 13.621 | 222.566 | 13.395 | 137.940 | 17.345 | 148.651 |
| 13.795 | 225.619 | 13.568 | 139.779 | 17.512 | 150.270 |
| 13.969 | 229.023 | 13.741 | 141.945 | 17.680 | 152.196 |
| 14.143 | 233.659 | 13.913 | 144.667 | 17.848 | 155.128 |
| 14.317 | 237.485 | 14.086 | 147.842 | 18.016 | 156.806 |
| 14.492 | 241.543 | 14.259 | 150.115 | 18.183 | 158.236 |
| 14.666 | 244.968 | 14.431 | 152.009 | 18.351 | 159.495 |
| 14.840 | 248.378 | 14.604 | 154.041 | 18.519 | 160.868 |
| 15.014 | 253.308 | 14.777 | 156.642 | 18.686 | 162.354 |
| 15.188 | 257.850 | 14.950 | 159.760 | 18.854 | 163.744 |
| 15.362 | 261.667 | 15.122 | 163.395 | 19.022 | 166.435 |
| 15.536 | 264.983 | 15.295 | 165.521 | 19.190 | 169.171 |
| 15.710 | 268.304 | 15.468 | 167.691 | 19.357 | 170.398 |
| 15.884 | 272.072 | 15.640 | 171.020 | 19.525 | 172.415 |
| 16.058 | 276.131 | 15.813 | 175.208 | 19.693 | 172.564 |
| 16.232 | 280.119 | 15.986 | 178.218 | 19.860 | 174.672 |
| 16.406 | 284.125 | 16.158 | 181.138 | 20.028 | 177.761 |
| 16.580 | 288.034 | 16.331 | 183.800 | 20.196 | 180.230 |
| 16.754 | 291.488 | 16.504 | 186.324 | 20.364 | 181.196 |
| 16.928 | 294.912 | 16.677 | 189.523 | 20.531 | 186.495 |
| 17.102 | 298.774 | 16.849 | 192.334 | 20.699 | 199.156 |
| 17.276 | 302.908 | 17.022 | 196.535 | 20.867 | 193.304 |
| 17.450 | 307.477 | 17.195 | 200.478 | 21.034 | 201.795 |
| 17.624 | 311.184 | 17.367 | 204.112 | 21.202 | 198.585 |
| 17.798 | 314.924 | 17.540 | 207.133 | 21.370 | 202.718 |
| 17.972 | 318.243 | 17.713 | 209.231 | 21.537 | 208.195 |
| 18.147 | 321.130 | 17.886 | 212.306 | 21.705 | 200.327 |
| 18.321 | 324.650 | 18.058 | 215.785 | 21.873 | 204.847 |
| 18.495 | 329.477 | 18.231 | 218.556 | 22.041 | 211.000 |
| 18.669 | 335.295 | 18.404 | 221.461 | 22.208 | 217.786 |
| 18.843 | 339.694 | 18.576 | 224.498 | 22.376 | 220.819 |
| 19.017 | 344.593 | 18.749 | 227.625 | 22.544 | 222.905 |
| 19.191 | 347.686 | 18.922 | 230.755 | 22.711 | 227.496 |
| 19.365 | 351.097 | 19.094 | 234.744 | 22.879 | 229.376 |
| 19.539 | 354.956 | 19.267 | 239.008 | 23.047 | 231.148 |
| 19.713 | 359.862 | 19.440 | 242.757 | 23.215 | 233.209 |
| 19.887 | 364.282 | 19.613 | 245.045 | 23.382 | 236.848 |
| 20.061 | 367.863 | 19.785 | 247.366 | 23.550 | 241.550 |
| 20.235 | 371.144 | 19.958 | 250.242 | 23.718 | 244.857 |
| 20.409 | 374.414 | 20.131 | 253.321 | 23.885 | 249.147 |
| 20.583 | 378.443 | 20.303 | 257.621 | 24.053 | 249.805 |
| 20.757 | 382.674 | 20.476 | 261.130 | 24.221 | 252.584 |
| 20.931 | 386.867 | 20.649 | 266.030 | 24.389 | 255.853 |
| 21.105 | 391.385 | 20.822 | 268.437 | 24.556 | 261.541 |
| 21.279 | 396.929 | 20.994 | 271.757 | 24.724 | 264.899 |
| 21.453 | 400.215 | 21.167 | 275.125 | 24.892 | 267.343 |
| 21.627 | 403.367 | 21.340 | 277.919 | 25.059 | 269.758 |
| 21.802 | 406.720 | 21.512 | 280.995 | 25.227 | 272.094 |
| 21.976 | 411.102 | 21.685 | 285.197 | 25.395 | 274.690 |
| 22.150 | 416.736 | 21.858 | 288.632 | 25.563 | 277.143 |
| 22.324 | 421.069 | 22.030 | 291.365 | 25.730 | 282.302 |
| 22.498 | 424.461 | 22.203 | 294.281 | 25.898 | 286.027 |
| 22.672 | 427.345 | 22.376 | 297.709 | 26.066 | 288.232 |
| 22.846 | 430.632 | 22.549 | 300.558 | 26.233 | 290.035 |
| 23.020 | 435.003 | 22.721 | 303.847 | 26.401 | 294.150 |
| 23.194 | 438.438 | 22.894 | 307.125 | 26.569 | 298.083 |
| 23.368 | 444.194 | 23.067 | 310.394 | 26.736 | 300.081 |
| 23.542 | 450.550 | 23.239 | 313.736 | 26.904 | 302.655 |
| 23.716 | 456.307 | 23.412 | 317.046 | 27.072 | 305.130 |
| 23.890 | 460.360 | 23.585 | 320.952 | 27.240 | 308.883 |
| 24.064 | 463.799 | 23.758 | 324.874 | 27.407 | 311.092 |
| 24.238 | 468.315 | 23.930 | 328.880 | 27.575 | 315.080 |
| 24.412 | 472.062 | 24.103 | 332.443 | 27.743 | 318.085 |
| 24.586 | 476.713 | 24.276 | 336.828 | 27.910 | 321.354 |
| 24.760 | 481.462 | 24.448 | 341.007 | 28.078 | 324.537 |
| 24.934 | 485.616 | 24.621 | 344.703 | 28.246 | 327.319 |
| 25.108 | 489.973 | 24.794 | 348.272 | 28.414 | 329.881 |
| 25.282 | 494.289 | 24.966 | 351.097 | 28.581 | 333.535 |
| 25.457 | 499.229 | 25.139 | 354.456 | 28.749 | 335.256 |
| 25.631 | 504.643 | 25.312 | 358.137 | 28.917 | 335.819 |
| 25.805 | 511.340 | 25.485 | 361.745 | 29.084 | 336.232 |
| 25.979 | 515.933 | 25.657 | 365.189 | 29.252 | 340.480 |
| 26.153 | 519.761 | 25.830 | 368.172 | 29.420 | 347.786 |
| 26.327 | 526.268 | 26.003 | 371.335 | 29.588 | 351.344 |
| 26.501 | 530.603 | 26.175 | 375.124 | 29.755 | 355.404 |
| 26.675 | 534.616 | 26.348 | 378.989 | 29.923 | 359.575 |
| 26.849 | 539.020 | 26.521 | 381.846 | 30.091 | 362.007 |
| 27.023 | 543.653 | 26.694 | 386.973 | 30.258 | 364.258 |
| 27.197 | 550.447 | 26.866 | 389.197 | 30.426 | 366.710 |
| 27.371 | 555.988 | 27.039 | 393.023 | 30.594 | 370.461 |
| 27.545 | 560.682 | 27.212 | 397.358 | 30.761 | 374.032 |
| 27.719 | 566.076 | 27.384 | 402.803 | 30.929 | 376.753 |
| 27.893 | 571.477 | 27.557 | 406.124 | 31.097 | 379.384 |
| 28.067 | 576.009 | 27.730 | 409.306 | 31.265 | 382.818 |
| 28.241 | 581.989 | 27.902 | 413.558 | 31.432 | 387.935 |
| 28.415 | 587.913 | 28.075 | 417.625 | 31.600 | 389.242 |
| 28.589 | 593.480 | 28.248 | 420.878 | 31.768 | 395.420 |
| 28.763 | 598.966 | 28.421 | 424.296 | 31.935 | 395.859 |
| 28.937 | 602.354 | 28.593 | 428.778 | 32.103 | 398.575 |
| 29.112 | 605.873 | 28.766 | 434.242 | 32.271 | 401.743 |
| 29.286 | 611.644 | 28.939 | 437.742 | 32.439 | 404.925 |
| 29.460 | 619.849 | 29.111 | 442.244 | 32.606 | 413.444 |
| 29.634 | 623.780 | 29.284 | 445.101 | 32.774 | 417.666 |
| 29.808 | 627.889 | 29.457 | 449.292 | 32.942 | 420.190 |
| 29.982 | 632.017 | 29.630 | 453.093 | 33.109 | 426.343 |
| 30.156 | 636.409 | 29.802 | 457.495 | 33.277 | 426.606 |
| 30.330 | 644.302 | 29.975 | 462.207 | 33.445 | 427.362 |
| 30.504 | 650.086 | 30.148 | 465.243 | 33.613 | 433.779 |
| 30.678 | 653.013 | 30.320 | 468.812 | 33.780 | 439.058 |
| 30.852 | 661.650 | 30.493 | 472.919 | 33.948 | 444.021 |
| 31.026 | 666.088 | 30.666 | 477.219 | 34.116 | 447.492 |
| 31.200 | 671.426 | 30.838 | 480.991 | 34.283 | 450.270 |
| 31.374 | 677.408 | 31.011 | 484.745 | 34.451 | 454.330 |
| 31.548 | 684.880 | 31.184 | 489.685 | 34.619 | 456.619 |
| 31.722 | 686.498 | 31.357 | 493.395 | 34.786 | 458.894 |
| 31.896 | 692.372 | 31.529 | 499.032 | 34.954 | 461.454 |
| 32.070 | 698.790 | 31.702 | 504.571 | 35.122 | 466.716 |
| 32.244 | 701.771 | 31.875 | 508.521 | 35.290 | 471.751 |
| 32.418 | 705.060 | 32.047 | 512.464 | 35.457 | 475.377 |
| 32.592 | 708.902 | 32.220 | 515.969 | 35.625 | 478.266 |
| 32.767 | 710.810 | 32.393 | 519.537 | 35.793 | 480.595 |
| 32.941 | 715.343 | 32.566 | 523.444 | 35.960 | 483.249 |
| 33.115 | 722.241 | 32.738 | 528.068 | 36.128 | 488.832 |
| 33.289 | 726.260 | 32.911 | 531.186 | 36.296 | 491.353 |
| 33.463 | 730.008 | 33.084 | 535.990 | 36.464 | 493.769 |
| 33.637 | 735.415 | 33.256 | 542.449 | 36.631 | 498.177 |
| 33.811 | 737.716 | 33.429 | 546.568 | 36.799 | 501.432 |
| 33.985 | 743.486 | 33.602 | 550.116 | 36.967 | 504.550 |
| 34.159 | 747.105 | 33.774 | 553.749 | 37.134 | 509.264 |
| 34.333 | 752.228 | 33.947 | 560.119 | 37.302 | 510.309 |
| 34.507 | 759.668 | 34.120 | 564.690 | 37.470 | 514.317 |
| 34.681 | 764.064 | 34.293 | 569.623 | 37.638 | 516.926 |
| 34.855 | 770.358 | 34.465 | 573.010 | 37.805 | 522.036 |
| 35.029 | 776.625 | 34.638 | 577.771 | 37.973 | 526.312 |
| 35.203 | 784.315 | 34.811 | 582.635 | 38.141 | 531.133 |
| 35.377 | 787.022 | 34.983 | 586.374 | 38.308 | 534.438 |
| 35.551 | 790.764 | 35.156 | 589.286 | 38.476 | 537.915 |
| 35.725 | 797.353 | 35.329 | 593.482 | 38.644 | 538.340 |
| 35.899 | 803.666 | 35.502 | 597.600 | 38.811 | 541.207 |
| 36.073 | 809.122 | 35.674 | 606.006 | 38.979 | 545.616 |
| 36.247 | 815.102 | 35.847 | 608.704 | 39.147 | 548.718 |
| 36.422 | 819.718 | 36.020 | 612.704 | 39.315 | 553.597 |
| 36.596 | 825.812 | 36.192 | 617.650 | 39.482 | 556.827 |
| 36.770 | 832.953 | 36.365 | 622.702 | 39.650 | 564.014 |
| 36.944 | 837.529 | 36.538 | 627.486 | 39.818 | 566.970 |
| 37.118 | 840.395 | 36.710 | 631.759 | 39.985 | 569.090 |
| 37.292 | 849.887 | 36.883 | 636.959 | 40.153 | 573.017 |
| 37.466 | 853.151 | 37.056 | 641.811 | 40.321 | 576.706 |
| 37.640 | 863.145 | 37.229 | 645.097 | 40.489 | 582.887 |
| 37.814 | 866.558 | 37.401 | 649.529 | 40.656 | 584.770 |
| 37.988 | 870.527 | 37.574 | 654.533 | 40.824 | 589.998 |
| 38.162 | 878.115 | 37.747 | 659.902 | 40.992 | 593.989 |
| 38.336 | 883.604 | 37.919 | 663.644 | 41.159 | 598.228 |
| 38.510 | 890.378 | 38.092 | 668.930 | 41.327 | 600.949 |
| 38.684 | 895.093 | 38.265 | 673.659 | 41.495 | 601.946 |
| 38.858 | 903.971 | 38.438 | 678.315 | 41.663 | 609.067 |
| 39.032 | 911.663 | 38.610 | 682.787 | 41.830 | 612.836 |
| 39.206 | 918.391 | 38.783 | 686.927 | 41.998 | 616.361 |
| 39.380 | 924.280 | 38.956 | 690.651 | 42.166 | 621.251 |
| 39.554 | 931.315 | 39.128 | 694.018 | 42.333 | 624.778 |
| 39.728 | 935.679 | 39.301 | 697.461 | 42.501 | 629.341 |
| 39.902 | 940.103 | 39.474 | 702.178 | 42.669 | 638.049 |
| 40.077 | 946.535 | 39.646 | 708.253 | 42.836 | 642.841 |
| 40.251 | 953.101 | 39.819 | 712.955 | 43.004 | 642.118 |
| 40.425 | 958.482 | 39.992 | 716.690 | 43.172 | 648.845 |
| 40.599 | 961.736 | 40.165 | 720.145 | 43.340 | 648.961 |
| 40.773 | 967.218 | 40.337 | 723.901 | 43.507 | 653.284 |
| 40.947 | 973.424 | 40.510 | 726.576 | 43.675 | 658.604 |
| 41.121 | 978.151 | 40.683 | 730.011 | 43.843 | 663.738 |
| 41.295 | 983.069 | 40.855 | 734.889 | 44.010 | 669.381 |
| 41.469 | 989.254 | 41.028 | 740.631 | 44.178 | 672.832 |
| 41.643 | 994.164 | 41.201 | 744.355 | 44.346 | 678.293 |
| 41.817 | 999.473 | 41.374 | 748.469 | 44.514 | 684.893 |
| 41.991 | 1005.932 | 41.546 | 754.220 | 44.681 | 690.553 |
| 42.165 | 1010.425 | 41.719 | 759.979 | 44.849 | 694.469 |
| 42.339 | 1014.430 | 41.892 | 763.861 | 45.017 | 698.884 |
| 42.513 | 1018.696 | 42.064 | 768.093 | 45.184 | 702.131 |
| 42.687 | 1023.355 | 42.237 | 771.539 | 45.352 | 705.905 |
| 42.861 | 1029.912 | 42.410 | 775.095 | 45.520 | 710.474 |
| 43.035 | 1035.536 | 42.583 | 779.742 | 45.688 | 717.895 |
| 43.209 | 1043.177 | 42.755 | 785.242 | 45.855 | 721.060 |
| 43.383 | 1047.815 | 42.928 | 787.597 | 46.023 | 724.994 |
| 43.558 | 1054.260 | 43.101 | 794.234 | 46.191 | 730.356 |
| 43.732 | 1060.183 | 43.273 | 798.637 | 46.358 | 735.157 |
| 43.906 | 1067.436 | 43.446 | 806.083 | 46.526 | 739.017 |
| 44.080 | 1072.804 | 43.619 | 815.974 | 46.694 | 742.919 |
| 44.254 | 1081.042 | 43.791 | 815.541 | 46.861 | 748.318 |
| 44.428 | 1086.152 | 43.964 | 818.719 | 47.029 | 754.003 |
| 44.602 | 1092.333 | 44.137 | 826.359 | 47.197 | 758.573 |
| 44.776 | 1097.403 | 44.310 | 827.956 | 47.365 | 762.227 |
| 44.950 | 1103.787 | 44.482 | 832.778 | 47.532 | 764.741 |
| 45.124 | 1113.046 | 44.655 | 837.964 | 47.700 | 767.255 |
| 45.298 | 1118.681 | 44.828 | 844.935 | 47.868 | 772.483 |
| 45.472 | 1126.078 | 45.000 | 853.610 | 48.035 | 777.104 |
| 45.646 | 1130.674 | 45.173 | 852.743 | 48.203 | 780.681 |
| 45.820 | 1137.664 | 45.346 | 858.075 | 48.371 | 784.971 |
| 45.994 | 1144.546 | 45.519 | 872.764 | 48.539 | 789.719 |
| 46.168 | 1147.457 | 45.691 | 877.287 | 48.706 | 794.714 |
| 46.342 | 1151.802 | 45.864 | 882.495 | 48.874 | 800.857 |
| 46.516 | 1163.064 | 46.037 | 887.744 | 49.042 | 807.200 |
| 46.690 | 1170.312 | 46.209 | 893.080 | 49.209 | 812.311 |
| 46.864 | 1175.552 | 46.382 | 898.143 | 49.377 | 812.306 |
| 47.038 | 1182.697 | 46.555 | 903.057 | 49.545 | 818.190 |
| 47.213 | 1188.925 | 46.727 | 908.236 | 49.713 | 823.461 |
| 47.387 | 1194.809 | 46.900 | 913.019 | 49.880 | 826.082 |
| 47.561 | 1202.103 | 47.073 | 917.531 | 50.048 | 834.841 |
| 47.735 | 1208.502 | 47.246 | 921.777 | 50.216 | 840.075 |
| 47.909 | 1213.052 | 47.418 | 925.659 | 50.383 | 842.811 |
| 48.083 | 1220.792 | 47.591 | 929.472 | 50.551 | 845.063 |
| 48.257 | 1227.510 | 47.764 | 936.024 | 50.719 | 848.462 |
| 48.431 | 1235.012 | 47.936 | 940.977 | 50.887 | 852.592 |
| 48.605 | 1241.542 | 48.109 | 946.250 | 51.054 | 855.121 |
| 48.779 | 1245.445 | 48.282 | 950.490 | 51.222 | 861.688 |
| 48.953 | 1254.497 | 48.455 | 956.377 | 51.390 | 867.740 |
| 49.127 | 1259.981 | 48.627 | 961.488 | 51.557 | 871.911 |
| 49.301 | 1271.926 | 48.800 | 967.974 | 51.725 | 877.658 |
| 49.475 | 1279.295 | 48.973 | 973.628 | 51.893 | 880.594 |
| 49.649 | 1283.499 | 49.145 | 978.357 | 52.060 | 883.841 |
| 49.823 | 1288.845 | 49.318 | 982.548 | 52.228 | 889.786 |
| 49.997 | 1295.801 | 49.491 | 988.688 | 52.396 | 893.867 |
| 50.171 | 1299.615 | 49.663 | 993.307 | 52.564 | 899.652 |
| 50.345 | 1307.877 | 49.836 | 998.102 | 52.731 | 907.574 |
| 50.519 | 1316.184 | 50.009 | 1002.274 | 52.899 | 911.396 |
| 50.693 | 1323.761 | 50.182 | 1008.431 | 53.067 | 914.497 |
| 50.868 | 1332.882 | 50.354 | 1013.725 | 53.234 | 917.593 |
| 51.042 | 1338.111 | 50.527 | 1020.289 | 53.402 | 922.211 |
| 51.216 | 1340.042 | 50.700 | 1025.131 | 53.570 | 925.625 |
| 51.390 | 1346.355 | 50.872 | 1031.933 | 53.738 | 931.738 |
| 51.564 | 1355.271 | 51.045 | 1036.159 | 53.905 | 938.506 |
| 51.738 | 1361.045 | 51.218 | 1040.423 | 54.073 | 942.521 |
| 51.912 | 1369.309 | 51.391 | 1046.466 | 54.241 | 947.982 |
| 52.086 | 1379.440 | 51.563 | 1054.301 | 54.408 | 950.301 |
| 52.260 | 1386.130 | 51.736 | 1060.010 | 54.576 | 952.267 |
| 52.434 | 1389.924 | 51.909 | 1064.552 | 54.744 | 959.132 |
| 52.608 | 1394.714 | 52.081 | 1069.884 | 54.912 | 961.999 |
| 52.782 | 1400.265 | 52.254 | 1074.027 | 55.079 | 966.152 |
| 52.956 | 1409.670 | 52.427 | 1078.824 | 55.247 | 968.489 |
| 53.130 | 1414.807 | 52.599 | 1084.605 | 55.415 | 973.870 |
| 53.304 | 1422.565 | 52.772 | 1090.298 | 55.582 | 980.277 |
| 53.478 | 1428.246 | 52.945 | 1095.955 | 55.750 | 982.077 |
| 53.652 | 1430.032 | 53.118 | 1099.915 | 55.918 | 987.228 |
| 53.826 | 1434.563 | 53.290 | 1104.964 | 56.085 | 990.508 |
| 54.000 | 1439.498 | 53.463 | 1111.485 | 56.253 | 996.710 |
| 54.174 | 1447.402 | 53.636 | 1116.227 | 56.421 | 1000.849 |
| 54.348 | 1454.269 | 53.808 | 1119.981 | 56.589 | 1007.007 |
| 54.523 | 1460.341 | 53.981 | 1124.987 | 56.756 | 1010.253 |
| 54.697 | 1467.580 | 54.154 | 1131.328 | 56.924 | 1016.745 |
| 54.871 | 1474.082 | 54.327 | 1136.153 | 57.092 | 1022.955 |
| 55.045 | 1478.729 | 54.499 | 1142.457 | 57.259 | 1026.616 |
| 55.219 | 1484.756 | 54.672 | 1147.006 | 57.427 | 1033.710 |
| 55.393 | 1490.780 | 54.845 | 1152.373 | 57.595 | 1039.803 |
| 55.567 | 1500.060 | 55.017 | 1158.247 | 57.763 | 1045.654 |
| 55.741 | 1506.902 | 55.190 | 1163.267 | 57.930 | 1049.930 |
| 55.915 | 1510.952 | 55.363 | 1169.595 | 58.098 | 1053.533 |
| 56.089 | 1516.604 | 55.535 | 1173.883 | 58.266 | 1056.988 |
| 56.263 | 1525.592 | 55.708 | 1179.730 | 58.433 | 1063.729 |
| 56.437 | 1540.105 | 55.881 | 1185.788 | 58.601 | 1063.909 |
| 56.611 | 1540.920 | 56.054 | 1190.056 | 58.769 | 1069.447 |
| 56.785 | 1552.445 | 56.226 | 1195.340 | 58.937 | 1080.217 |
| 56.959 | 1566.383 | 56.399 | 1200.991 | 59.104 | 1083.601 |
| 57.133 | 1567.139 | 56.572 | 1207.230 | 59.272 | 1086.985 |
| 57.307 | 1570.520 | 56.744 | 1213.300 | 59.440 | 1092.728 |
| 57.481 | 1585.870 | 56.917 | 1218.256 | 59.607 | 1101.790 |
| 57.655 | 1596.367 | 57.090 | 1222.608 | 59.775 | 1103.534 |
| 57.829 | 1603.829 | 57.263 | 1227.670 | 59.943 | 1111.158 |
| 58.003 | 1609.456 | 57.435 | 1234.308 | 60.110 | 1116.799 |
| 58.178 | 1615.818 | 57.608 | 1240.358 | 60.278 | 1122.888 |
| 58.352 | 1623.394 | 57.781 | 1244.844 | 60.446 | 1130.392 |
| 58.526 | 1634.974 | 57.953 | 1249.150 | 60.614 | 1134.451 |
| 58.700 | 1643.619 | 58.126 | 1256.900 | 60.781 | 1137.363 |
| 58.874 | 1651.325 | 58.299 | 1262.390 | 60.949 | 1141.515 |
| 59.048 | 1654.903 | 58.471 | 1266.597 | 61.117 | 1148.628 |
| 59.222 | 1664.011 | 58.644 | 1270.583 | 61.284 | 1155.675 |
| 59.396 | 1674.347 | 58.817 | 1274.922 | 61.452 | 1163.633 |
| 59.570 | 1683.621 | 58.990 | 1280.804 | 61.620 | 1167.503 |
| 59.744 | 1689.341 | 59.162 | 1285.973 | 61.788 | 1171.034 |
| 59.918 | 1699.052 | 59.335 | 1290.982 | 61.955 | 1174.894 |
| 60.092 | 1712.852 | 59.508 | 1296.547 | 62.123 | 1179.041 |
| 60.266 | 1723.673 | 59.680 | 1302.242 | 62.291 | 1186.575 |
| 60.440 | 1732.544 | 59.853 | 1306.844 | 62.458 | 1194.697 |
| 60.614 | 1742.347 | 60.026 | 1313.434 | 62.626 | 1197.446 |
| 60.788 | 1749.129 | 60.199 | 1318.766 | 62.794 | 1202.532 |
| 60.962 | 1755.706 | 60.371 | 1322.415 | 62.962 | 1213.375 |
| 61.136 | 1759.543 | 60.544 | 1329.987 | 63.129 | 1216.487 |
| 61.310 | 1767.007 | 60.717 | 1339.346 | 63.297 | 1219.775 |
| 61.484 | 1774.046 | 60.889 | 1340.954 | 63.465 | 1225.204 |
| 61.658 | 1779.849 | 61.062 | 1349.753 | 63.632 | 1227.069 |
| 61.833 | 1790.570 | 61.235 | 1352.083 | 63.800 | 1231.968 |
| 62.007 | 1798.125 | 61.407 | 1360.959 | 63.968 | 1242.581 |
| 62.181 | 1806.832 | 61.580 | 1367.097 | 64.135 | 1246.412 |
| 62.355 | 1814.329 | 61.753 | 1371.148 | 64.303 | 1248.994 |
| 62.529 | 1820.490 | 61.926 | 1378.924 | 64.471 | 1252.677 |
| 62.703 | 1826.790 | 62.098 | 1384.310 | 64.639 | 1255.329 |
| 62.877 | 1833.494 | 62.271 | 1391.793 | 64.806 | 1257.723 |
| 63.051 | 1847.179 | 62.444 | 1397.717 | 64.974 | 1259.975 |
| 63.225 | 1855.889 | 62.616 | 1401.058 | 65.142 | 1263.883 |
| 63.399 | 1861.616 | 62.789 | 1407.045 | 65.309 | 1267.873 |
| 63.573 | 1866.417 | 62.962 | 1412.327 | 65.477 | 1270.040 |
| 63.747 | 1872.254 | 63.135 | 1418.497 | 65.645 | 1273.465 |
| 63.921 | 1880.959 | 63.307 | 1424.412 | 65.813 | 1278.796 |
| 64.095 | 1886.624 | 63.480 | 1430.673 | 65.980 | 1287.313 |
| 64.269 | 1893.389 | 63.653 | 1437.383 | 66.148 | 1290.007 |
| 64.443 | 1907.357 | 63.825 | 1444.094 | 66.316 | 1292.085 |
| 64.617 | 1913.942 | 63.998 | 1448.852 | 66.483 | 1301.016 |
| 64.791 | 1916.114 | 64.171 | 1454.697 | 66.651 | 1308.229 |
| 64.965 | 1925.649 | 64.343 | 1461.624 | 66.819 | 1312.766 |
| 65.139 | 1938.763 | 64.516 | 1469.316 | 66.987 | 1320.201 |
| 65.313 | 1947.283 | 64.689 | 1473.933 | 67.154 | 1325.567 |
| 65.488 | 1956.847 | 64.862 | 1478.937 | 67.322 | 1332.044 |
| 65.662 | 1966.432 | 65.034 | 1489.143 | 67.490 | 1336.117 |
| 65.836 | 1972.817 | 65.207 | 1494.205 | 67.657 | 1343.592 |
| 66.010 | 1978.036 | 65.380 | 1500.334 | 67.825 | 1352.774 |
| 66.184 | 1984.009 | 65.552 | 1504.229 | 67.993 | 1360.000 |
| 66.358 | 1995.732 | 65.725 | 1507.808 | 68.160 | 1364.708 |
| 66.532 | 2006.603 | 65.898 | 1510.388 | 68.328 | 1368.990 |
| 66.706 | 2020.177 | 66.071 | 1517.529 | 68.496 | 1373.298 |
| 66.880 | 2026.634 | 66.243 | 1524.079 | 68.664 | 1376.449 |
| 67.054 | 2037.743 | 66.416 | 1531.504 | 68.831 | 1379.618 |
| 67.228 | 2044.090 | 66.589 | 1534.123 | 68.999 | 1382.914 |
| 67.402 | 2054.781 | 66.761 | 1542.951 | 69.167 | 1393.227 |
| 67.576 | 2064.368 | 66.934 | 1549.299 | 69.334 | 1404.679 |
| 67.750 | 2074.306 | 67.107 | 1557.724 | 69.502 | 1405.999 |
| 67.924 | 2081.708 | 67.279 | 1564.763 | 69.670 | 1413.969 |
| 68.098 | 2089.271 | 67.452 | 1570.529 | 69.838 | 1409.318 |
| 68.272 | 2095.746 | 67.625 | 1574.381 | 70.005 | 1422.855 |
| 68.446 | 2101.125 | 67.798 | 1581.071 | 70.173 | 1427.562 |
| 68.620 | 2107.756 | 67.970 | 1588.147 | 70.341 | 1438.226 |
| 68.794 | 2115.046 | 68.143 | 1593.798 | 70.508 | 1450.448 |
| 68.968 | 2121.531 | 68.316 | 1602.181 | 70.676 | 1456.345 |
| 69.143 | 2127.220 | 68.488 | 1609.826 | 70.844 | 1466.180 |
| 69.317 | 2132.567 | 68.661 | 1614.106 | 71.012 | 1473.116 |
| 69.491 | 2139.140 | 68.834 | 1618.301 | 71.179 | 1478.527 |
| 69.665 | 2158.109 | 69.007 | 1621.903 | 71.347 | 1482.555 |
| 69.839 | 2163.762 | 69.179 | 1627.090 | 71.515 | 1489.896 |
| 70.013 | 2173.370 | 69.352 | 1634.064 | 71.682 | 1495.262 |
| 70.187 | 2187.546 | 69.525 | 1639.620 | 71.850 | 1504.110 |
| 70.361 | 2199.864 | 69.697 | 1645.139 | 72.018 | 1509.367 |
| 70.535 | 2204.173 | 69.870 | 1651.520 | 72.186 | 1518.695 |
| 70.709 | 2214.070 | 70.043 | 1660.150 | 72.353 | 1518.959 |
| 70.883 | 2220.875 | 70.216 | 1666.874 | 72.521 | 1527.812 |
| 71.057 | 2229.494 | 70.388 | 1674.076 | 72.689 | 1535.868 |
| 71.231 | 2236.060 | 70.561 | 1680.339 | 72.856 | 1539.283 |
| 71.405 | 2243.699 | 70.734 | 1685.049 | 73.024 | 1547.789 |
| 71.579 | 2251.220 | 70.906 | 1693.168 | 73.192 | 1551.869 |
| 71.753 | 2259.492 | 71.079 | 1698.055 | 73.359 | 1556.666 |
| 71.927 | 2269.580 | 71.252 | 1704.579 | 73.527 | 1562.133 |
| 72.101 | 2273.456 | 71.424 | 1712.174 | 73.695 | 1567.148 |
| 72.275 | 2284.925 | 71.597 | 1718.187 | 73.863 | 1571.373 |
| 72.449 | 2293.035 | 71.770 | 1723.552 | 74.030 | 1576.698 |
| 72.624 | 2300.447 | 71.943 | 1731.802 | 74.198 | 1585.916 |
| 72.798 | 2312.992 | 72.115 | 1738.021 | 74.366 | 1594.487 |
| 72.972 | 2322.241 | 72.288 | 1745.859 | 74.533 | 1605.267 |
| 73.146 | 2334.190 | 72.461 | 1751.182 | 74.701 | 1605.193 |
| 73.320 | 2338.876 | 72.633 | 1757.937 | 74.869 | 1611.061 |
| 73.494 | 2345.603 | 72.806 | 1767.182 | 75.037 | 1619.952 |
| 73.668 | 2356.744 | 72.979 | 1773.120 | 75.204 | 1625.014 |
| 73.842 | 2363.034 | 73.152 | 1779.119 | 75.372 | 1629.119 |
| 74.016 | 2371.618 | 73.324 | 1784.261 | 75.540 | 1631.727 |
| 74.190 | 2381.531 | 73.497 | 1790.202 | 75.707 | 1634.205 |
| 74.364 | 2386.634 | 73.670 | 1793.242 | 75.875 | 1638.090 |
| 74.538 | 2400.750 | 73.842 | 1800.104 | 76.043 | 1643.376 |
| 74.712 | 2409.644 | 74.015 | 1807.094 | 76.211 | 1647.340 |
| 74.886 | 2419.289 | 74.188 | 1817.261 | 76.378 | 1651.221 |
| 75.060 | 2429.658 | 74.360 | 1822.045 | 76.546 | 1657.433 |
| 75.234 | 2433.790 | 74.533 | 1827.393 | 76.714 | 1664.089 |
| 75.408 | 2449.550 | 74.706 | 1835.016 | 76.881 | 1665.094 |
| 75.582 | 2460.904 | 74.879 | 1842.443 | 77.049 | 1673.356 |
| 75.756 | 2472.892 | 75.051 | 1851.176 | 77.217 | 1678.259 |
| 75.930 | 2484.963 | 75.224 | 1857.513 | 77.384 | 1687.313 |
| 76.104 | 2488.142 | 75.397 | 1862.935 | 77.552 | 1693.964 |
| 76.279 | 2495.292 | 75.569 | 1871.023 | 77.720 | 1699.048 |
| 76.453 | 2502.414 | 75.742 | 1878.076 | 77.888 | 1710.155 |
| 76.627 | 2508.270 | 75.915 | 1886.289 | 78.055 | 1717.848 |
| 76.801 | 2515.590 | 76.088 | 1895.088 | 78.223 | 1724.316 |
| 76.975 | 2529.747 | 76.260 | 1898.418 | 78.391 | 1728.705 |
| 77.149 | 2546.705 | 76.433 | 1904.018 | 78.558 | 1733.371 |
| 77.323 | 2550.964 | 76.606 | 1911.634 | 78.726 | 1738.687 |
| 77.497 | 2562.153 | 76.778 | 1921.255 | 78.894 | 1744.711 |
| 77.671 | 2571.200 | 76.951 | 1927.195 | 79.062 | 1750.023 |
| 77.845 | 2581.126 | 77.124 | 1933.885 | 79.229 | 1762.685 |
| 78.019 | 2593.118 | 77.296 | 1939.933 | 79.397 | 1779.901 |
| 78.193 | 2604.296 | 77.469 | 1947.013 | 79.565 | 1766.455 |
| 78.367 | 2616.247 | 77.642 | 1951.784 | 79.732 | 1771.913 |
| 78.541 | 2626.449 | 77.815 | 1956.495 | 79.900 | 1777.191 |
| 78.715 | 2631.980 | 77.987 | 1962.152 | 80.068 | 1788.780 |
| 78.889 | 2640.990 | 78.160 | 1972.604 | 80.236 | 1805.260 |
| 79.063 | 2647.071 | 78.333 | 1979.766 | 80.403 | 1815.198 |
| 79.237 | 2652.058 | 78.505 | 1985.526 | 80.571 | 1819.942 |
| 79.411 | 2661.518 | 78.678 | 1994.721 | 80.739 | 1828.690 |
| 79.585 | 2670.512 | 78.851 | 1998.864 | 80.906 | 1834.677 |
| 79.759 | 2678.360 | 79.024 | 2006.951 | 81.074 | 1832.892 |
| 79.934 | 2685.713 | 79.196 | 2013.134 | 81.242 | 1841.728 |
| 80.108 | 2695.906 | 79.369 | 2020.208 | 81.409 | 1847.692 |
| 80.282 | 2704.972 | 79.542 | 2030.408 | 81.577 | 1859.957 |
| 80.456 | 2713.139 | 79.714 | 2038.299 | 81.745 | 1866.625 |
| 80.630 | 2721.209 | 79.887 | 2043.608 | 81.913 | 1871.002 |
| 80.804 | 2728.136 | 80.060 | 2051.200 | 82.080 | 1881.315 |
| 80.978 | 2735.864 | 80.232 | 2057.702 | 82.248 | 1886.713 |
| 81.152 | 2746.320 | 80.405 | 2066.687 | 82.416 | 1891.225 |
| 81.326 | 2756.603 | 80.578 | 2072.684 | 82.583 | 1897.186 |
| 81.500 | 2781.326 | 80.751 | 2081.498 | 82.751 | 1903.264 |
| 81.674 | 2786.447 | 80.923 | 2086.709 | 82.919 | 1915.053 |
| 81.848 | 2802.954 | 81.096 | 2095.228 | 83.087 | 1919.377 |
| 82.022 | 2800.155 | 81.269 | 2102.536 | 83.254 | 1926.824 |
| 82.196 | 2812.534 | 81.441 | 2107.948 | 83.422 | 1934.914 |
| 82.370 | 2826.730 | 81.614 | 2116.261 | 83.590 | 1940.234 |
| 82.544 | 2839.700 | 81.787 | 2125.942 | 83.757 | 1944.148 |
| 82.718 | 2848.979 | 81.960 | 2132.333 | 83.925 | 1949.528 |
| 82.892 | 2859.276 | 82.132 | 2140.149 | 84.093 | 1959.023 |
| 83.066 | 2869.387 | 82.305 | 2148.089 | 84.261 | 1968.013 |
| 83.240 | 2877.109 | 82.478 | 2156.066 | 84.428 | 1975.010 |
| 83.414 | 2889.895 | 82.650 | 2161.944 | 84.596 | 1981.857 |
| 83.589 | 2895.074 | 82.823 | 2172.886 | 84.764 | 1989.557 |
| 83.763 | 2908.417 | 82.996 | 2181.640 | 84.931 | 1995.219 |
| 83.937 | 2918.981 | 83.168 | 2188.343 | 85.099 | 1999.580 |
| 84.111 | 2923.787 | 83.341 | 2196.389 | 85.267 | 2006.992 |
| 84.285 | 2936.125 | 83.514 | 2201.814 | 85.434 | 2014.648 |
| 84.459 | 2954.356 | 83.687 | 2206.929 | 85.602 | 2023.839 |
| 84.633 | 2960.919 | 83.859 | 2213.975 | 85.770 | 2029.816 |
| 84.807 | 2969.504 | 84.032 | 2222.540 | 85.938 | 2038.042 |
| 84.981 | 2980.793 | 84.205 | 2230.053 | 86.105 | 2041.939 |
| 85.155 | 2992.394 | 84.377 | 2241.579 | 86.273 | 2046.244 |
| 85.329 | 2999.938 | 84.550 | 2248.108 | 86.441 | 2050.255 |
| 85.503 | 3009.028 | 84.723 | 2253.304 | 86.608 | 2058.154 |
| 85.677 | 3016.968 | 84.896 | 2259.429 | 86.776 | 2068.873 |
| 85.851 | 3029.633 | 85.068 | 2268.918 | 86.944 | 2073.496 |
| 86.025 | 3041.180 | 85.241 | 2276.444 | 87.112 | 2082.225 |
| 86.199 | 3053.121 | 85.414 | 2285.338 | 87.279 | 2092.251 |
| 86.373 | 3059.629 | 85.586 | 2290.824 | 87.447 | 2105.233 |
| 86.547 | 3066.679 | 85.759 | 2298.860 | 87.615 | 2112.380 |
| 86.721 | 3076.531 | 85.932 | 2303.686 | 87.782 | 2119.777 |
| 86.895 | 3084.913 | 86.104 | 2313.382 | 87.950 | 2125.437 |
| 87.069 | 3098.367 | 86.277 | 2322.467 | 88.118 | 2130.713 |
| 87.244 | 3108.581 | 86.450 | 2328.377 | 88.286 | 2141.042 |
| 87.418 | 3119.862 | 86.623 | 2334.023 | 88.453 | 2145.987 |
| 87.592 | 3128.390 | 86.795 | 2342.112 | 88.621 | 2150.887 |
| 87.766 | 3141.248 | 86.968 | 2350.364 | 88.789 | 2156.143 |
| 87.940 | 3156.303 | 87.141 | 2357.213 | 88.956 | 2160.676 |
| 88.114 | 3169.105 | 87.313 | 2364.596 | 89.124 | 2167.949 |
| 88.288 | 3177.207 | 87.486 | 2375.309 | 89.292 | 2176.413 |
| 88.462 | 3188.833 | 87.659 | 2383.398 | 89.459 | 2186.787 |
| 88.636 | 3201.200 | 87.832 | 2387.838 | 89.627 | 2194.818 |
| 88.810 | 3211.694 | 88.004 | 2397.418 | 89.795 | 2201.309 |
| 88.984 | 3222.203 | 88.177 | 2406.823 | 89.963 | 2209.339 |
| 89.158 | 3233.246 | 88.350 | 2411.987 | 90.130 | 2213.651 |
| 89.332 | 3242.603 | 88.522 | 2420.244 | 90.298 | 2217.397 |
| 89.506 | 3250.770 | 88.695 | 2424.309 | 90.466 | 2225.517 |
| 89.680 | 3258.816 | 88.868 | 2433.528 | 90.633 | 2235.941 |
| 89.854 | 3274.657 | 89.040 | 2440.805 | 90.801 | 2247.858 |
| 90.028 | 3283.884 | 89.213 | 2448.875 | 90.969 | 2254.813 |
| 90.202 | 3292.157 | 89.386 | 2456.160 | 91.137 | 2262.532 |
| 90.376 | 3300.226 | 89.559 | 2463.783 | 91.304 | 2271.548 |
| 90.550 | 3307.756 | 89.731 | 2472.293 | 91.472 | 2278.294 |
| 90.724 | 3315.228 | 89.904 | 2477.878 | 91.640 | 2285.299 |
| 90.899 | 3330.407 | 90.077 | 2485.929 | 91.807 | 2285.485 |
| 91.073 | 3339.547 | 90.249 | 2493.754 | 91.975 | 2292.338 |
| 91.247 | 3352.498 | 90.422 | 2503.458 | 92.143 | 2306.643 |
| 91.421 | 3357.393 | 90.595 | 2512.121 | 92.311 | 2314.862 |
| 91.595 | 3368.891 | 90.768 | 2517.039 | 92.478 | 2320.950 |
| 91.769 | 3381.295 | 90.940 | 2524.680 | 92.646 | 2325.168 |
| 91.943 | 3391.280 | 91.113 | 2534.297 | 92.814 | 2332.599 |
| 92.117 | 3403.996 | 91.286 | 2542.286 | 92.981 | 2342.137 |
| 92.291 | 3415.348 | 91.458 | 2548.740 | 93.149 | 2348.720 |
| 92.465 | 3428.958 | 91.631 | 2554.689 | 93.317 | 2357.063 |
| 92.639 | 3438.786 | 91.804 | 2562.226 | 93.485 | 2363.680 |
| 92.813 | 3448.832 | 91.976 | 2570.208 | 93.652 | 2368.036 |
| 92.987 | 3457.852 | 92.149 | 2579.342 | 93.820 | 2372.802 |
| 93.161 | 3468.314 | 92.322 | 2585.288 | 93.988 | 2381.252 |
| 93.335 | 3477.371 | 92.495 | 2590.189 | 94.155 | 2387.457 |
| 93.509 | 3487.371 | 92.667 | 2606.742 | 94.323 | 2389.690 |
| 93.683 | 3498.151 | 92.840 | 2616.657 | 94.491 | 2405.858 |
| 93.857 | 3507.045 | 93.013 | 2625.820 | 94.658 | 2415.229 |
| 94.031 | 3516.132 | 93.185 | 2635.109 | 94.826 | 2421.245 |
| 94.205 | 3523.681 | 93.358 | 2641.234 | 94.994 | 2427.301 |
| 94.379 | 3534.808 | 93.531 | 2646.526 | 95.162 | 2434.392 |
| 94.554 | 3548.028 | 93.704 | 2656.479 | 95.329 | 2441.969 |
| 94.728 | 3563.540 | 93.876 | 2665.431 | 95.497 | 2448.005 |
| 94.902 | 3575.924 | 94.049 | 2674.289 | 95.665 | 2450.340 |
| 95.076 | 3590.905 | 94.222 | 2680.273 | 95.832 | 2459.028 |
| 95.250 | 3602.470 | 94.394 | 2688.326 | 96.000 | 2470.117 |
| 95.424 | 3610.318 | 94.567 | 2695.360 | 96.168 | 2476.790 |
| 95.598 | 3623.582 | 94.740 | 2701.342 | 96.336 | 2483.025 |
| 95.772 | 3626.993 | 94.912 | 2711.746 | 96.503 | 2490.054 |
| 95.946 | 3637.915 | 95.085 | 2724.659 | 96.671 | 2496.243 |
| 96.120 | 3648.613 | 95.258 | 2731.972 | 96.839 | 2504.882 |
| 96.294 | 3661.453 | 95.431 | 2740.481 | 97.006 | 2511.717 |
| 96.468 | 3673.383 | 95.603 | 2751.303 | 97.174 | 2516.772 |
| 96.642 | 3682.065 | 95.776 | 2762.630 | 97.342 | 2520.220 |
| 96.816 | 3690.956 | 95.949 | 2764.994 | 97.510 | 2527.269 |
| 96.990 | 3699.751 | 96.121 | 2775.525 | 97.677 | 2533.244 |
| 97.164 | 3710.731 | 96.294 | 2783.683 | 97.845 | 2541.817 |
| 97.338 | 3733.154 | 96.467 | 2792.402 | 98.013 | 2556.444 |
| 97.512 | 3748.499 | 96.640 | 2800.357 | 98.180 | 2560.637 |
| 97.686 | 3757.681 | 96.812 | 2808.842 | 98.348 | 2562.027 |
| 97.860 | 3767.304 | 96.985 | 2818.624 | 98.516 | 2571.768 |
| 98.035 | 3776.867 | 97.158 | 2824.970 | 98.683 | 2577.529 |
| 98.209 | 3787.356 | 97.330 | 2836.070 | 98.851 | 2583.638 |
| 98.383 | 3796.634 | 97.503 | 2849.063 | 99.019 | 2596.266 |
| 98.557 | 3808.625 | 97.676 | 2858.870 | 99.187 | 2605.248 |
| 98.731 | 3816.945 | 97.849 | 2867.260 | 99.354 | 2614.954 |
| 98.905 | 3827.508 | 98.021 | 2875.828 | 99.522 | 2615.807 |
| 99.079 | 3844.252 | 98.194 | 2884.859 | 99.690 | 2632.357 |
| 99.253 | 3853.138 | 98.367 | 2893.135 | 99.857 | 2641.022 |
| 99.427 | 3862.931 | 98.539 | 2903.165 | 100.025 | 2647.115 |
| 99.601 | 3886.646 | 98.712 | 2914.177 | 100.193 | 2653.415 |
| 99.775 | 3889.889 | 98.885 | 2924.896 | 100.361 | 2659.347 |
| 99.949 | 3896.700 | 99.057 | 2933.762 | 100.528 | 2666.886 |
| 100.123 | 3918.132 | 99.230 | 2938.205 | 100.696 | 2673.589 |
| 100.297 | 3927.132 | 99.403 | 2944.854 | 100.864 | 2679.348 |
| 100.471 | 3937.635 | 99.576 | 2952.088 | 101.031 | 2683.529 |
| 100.645 | 3949.409 | 99.748 | 2961.680 | 101.199 | 2687.034 |
| 100.819 | 3959.029 | 99.921 | 2969.231 | 101.367 | 2692.518 |
| 100.993 | 3966.541 | 100.094 | 2974.887 | 101.535 | 2695.232 |
| 101.167 | 3978.395 | 100.266 | 2984.031 | 101.702 | 2702.319 |
| 101.341 | 3994.392 | 100.439 | 2994.088 | 101.870 | 2713.440 |
| 101.515 | 4002.189 | 100.612 | 3004.233 | 102.038 | 2721.242 |
| 101.690 | 4009.486 | 100.785 | 3011.427 | 102.205 | 2732.727 |
| 101.864 | 4017.243 | 100.957 | 3021.768 | 102.373 | 2738.778 |
| 102.038 | 4025.522 | 101.130 | 3031.336 | 102.541 | 2747.782 |
| 102.212 | 4036.732 | 101.303 | 3039.579 | 102.708 | 2754.833 |
| 102.386 | 4048.771 | 101.475 | 3046.717 | 102.876 | 2783.017 |
| 102.560 | 4060.160 | 101.648 | 3054.699 | 103.044 | 2776.226 |
| 102.734 | 4070.413 | 101.821 | 3066.528 | 103.212 | 2784.187 |
| 102.908 | 4080.335 | 101.993 | 3073.944 | 103.379 | 2806.355 |
| 103.082 | 4089.113 | 102.166 | 3083.270 | 103.547 | 2804.186 |
| 103.256 | 4099.206 | 102.339 | 3091.016 | 103.715 | 2827.594 |
| 103.430 | 4108.175 | 102.512 | 3100.810 | 103.882 | 2835.291 |
| 103.604 | 4124.477 | 102.684 | 3110.736 | 104.050 | 2842.767 |
| 103.778 | 4129.938 | 102.857 | 3119.459 | 104.218 | 2848.453 |
| 103.952 | 4139.068 | 103.030 | 3127.317 | 104.386 | 2855.872 |
| 104.126 | 4149.343 | 103.202 | 3138.305 | 104.553 | 2865.740 |
| 104.300 | 4157.754 | 103.375 | 3143.006 | 104.721 | 2872.556 |
| 104.474 | 4171.716 | 103.548 | 3154.352 | 104.889 | 2882.106 |
| 104.648 | 4184.913 | 103.721 | 3161.753 | 105.056 | 2889.422 |
| 104.822 | 4201.968 | 103.893 | 3169.470 | 105.224 | 2901.256 |
| 104.996 | 4209.843 | 104.066 | 3178.688 | 105.392 | 2907.803 |
| 105.170 | 4236.446 | 104.239 | 3189.945 | 105.560 | 2920.237 |
| 105.345 | 4245.117 | 104.411 | 3198.135 | 105.727 | 2929.001 |
| 105.519 | 4253.975 | 104.584 | 3204.371 | 105.895 | 2937.275 |
| 105.693 | 4263.493 | 104.757 | 3210.703 | 106.063 | 2948.313 |
| 105.867 | 4273.918 | 104.929 | 3221.508 | 106.230 | 2956.545 |
| 106.041 | 4280.556 | 105.102 | 3229.535 | 106.398 | 2964.510 |
| 106.215 | 4286.206 | 105.275 | 3239.588 | 106.566 | 2971.453 |
| 106.389 | 4291.908 | 105.448 | 3250.869 | 106.733 | 2974.924 |
| 106.563 | 4298.071 | 105.620 | 3258.879 | 106.901 | 2984.779 |
| 106.737 | 4311.094 | 105.793 | 3270.078 | 107.069 | 2998.609 |
| 106.911 | 4324.792 | 105.966 | 3281.702 | 107.237 | 3011.419 |
| 107.085 | 4338.185 | 106.138 | 3290.020 | 107.404 | 3017.267 |
| 107.259 | 4353.083 | 106.311 | 3298.854 | 107.572 | 3021.567 |
| 107.433 | 4360.459 | 106.484 | 3303.382 | 107.740 | 3028.564 |
| 107.607 | 4373.647 | 106.657 | 3318.188 | 107.907 | 3037.118 |
| 107.781 | 4391.012 | 106.829 | 3329.284 | 108.075 | 3046.624 |
| 107.955 | 4410.144 | 107.002 | 3336.087 | 108.243 | 3058.155 |
| 108.129 | 4424.727 | 107.175 | 3348.342 | 108.411 | 3065.663 |
| 108.303 | 4427.127 | 107.347 | 3354.092 | 108.578 | 3074.851 |
| 108.477 | 4451.959 | 107.520 | 3359.343 | 108.746 | 3080.898 |
| 108.651 | 4462.997 | 107.693 | 3365.214 | 108.914 | 3086.292 |
| 108.825 | 4476.304 | 107.865 | 3373.262 | 109.081 | 3091.707 |
| 109.000 | 4493.521 | 108.038 | 3380.757 | 109.249 | 3101.981 |
| 109.174 | 4512.291 | 108.211 | 3391.516 | 109.417 | 3105.398 |
| 109.348 | 4520.460 | 108.384 | 3402.376 | 109.585 | 3117.074 |
| 109.522 | 4529.174 | 108.556 | 3410.430 | 109.752 | 3129.502 |
| 109.696 | 4541.029 | 108.729 | 3418.706 | 109.920 | 3141.792 |
| 109.870 | 4553.742 | 108.902 | 3429.550 | 110.088 | 3147.485 |
| 110.044 | 4583.968 | 109.074 | 3439.410 | 110.255 | 3153.178 |
| 110.218 | 4580.683 | 109.247 | 3450.160 | 110.423 | 3161.016 |
| 110.392 | 4606.293 | 109.420 | 3455.174 | 110.591 | 3162.385 |
| 110.566 | 4627.522 | 109.593 | 3466.671 | 110.758 | 3168.252 |
| 110.740 | 4635.106 | 109.765 | 3476.670 | 110.926 | 3180.226 |
| 110.914 | 4644.769 | 109.938 | 3483.911 | 111.094 | 3183.719 |
| 111.088 | 4656.700 | 110.111 | 3491.501 | 111.262 | 3197.456 |
| 111.262 | 4668.088 | 110.283 | 3501.172 | 111.429 | 3204.797 |
| 111.436 | 4681.569 | 110.456 | 3509.894 | 111.597 | 3215.502 |
| 111.610 | 4696.325 | 110.629 | 3516.217 | 111.765 | 3223.974 |
| 111.784 | 4706.319 | 110.801 | 3526.267 | 111.932 | 3236.951 |
| 111.958 | 4718.467 | 110.974 | 3534.114 | 112.100 | 3250.819 |
| 112.132 | 4737.161 | 111.147 | 3544.701 | 112.268 | 3253.785 |
| 112.306 | 4741.222 | 111.320 | 3555.572 | 112.436 | 3262.061 |
| 112.480 | 4761.471 | 111.492 | 3564.789 | 112.603 | 3270.700 |
| 112.655 | 4769.133 | 111.665 | 3573.098 | 112.771 | 3278.429 |
| 112.829 | 4776.532 | 111.838 | 3581.482 | 112.939 | 3284.779 |
| 113.003 | 4787.688 | 112.010 | 3590.648 | 113.106 | 3292.761 |
| 113.177 | 4793.862 | 112.183 | 3605.214 | 113.274 | 3306.367 |
| 113.351 | 4811.428 | 112.356 | 3612.922 | 113.442 | 3313.840 |
| 113.525 | 4821.784 | 112.529 | 3622.861 | 113.610 | 3321.585 |
| 113.699 | 4833.082 | 112.701 | 3629.444 | 113.777 | 3329.204 |
| 113.873 | 4840.111 | 112.874 | 3634.523 | 113.945 | 3335.849 |
| 114.047 | 4843.894 | 113.047 | 3645.501 | 114.113 | 3343.758 |
| 114.221 | 4855.310 | 113.219 | 3655.891 | 114.280 | 3353.664 |
| 114.395 | 4868.417 | 113.392 | 3667.273 | 114.448 | 3367.370 |
| 114.569 | 4877.593 | 113.565 | 3674.896 | 114.616 | 3371.054 |
| 114.743 | 4891.856 | 113.737 | 3685.329 | 114.783 | 3378.636 |
| 114.917 | 4904.802 | 113.910 | 3696.661 | 114.951 | 3392.382 |
| 115.091 | 4910.997 | 114.083 | 3705.520 | 115.119 | 3399.689 |
| 115.265 | 4917.217 | 114.256 | 3718.575 | 115.287 | 3417.577 |
| 115.439 | 4927.093 | 114.428 | 3725.145 | 115.454 | 3426.011 |
| 115.613 | 4938.048 | 114.601 | 3734.496 | 115.622 | 3435.395 |
| 115.787 | 4951.365 | 114.774 | 3749.606 | 115.790 | 3444.797 |
| 115.961 | 4967.042 | 114.946 | 3758.144 | 115.957 | 3449.941 |
| 116.135 | 4983.787 | 115.119 | 3765.384 | 116.125 | 3454.553 |
| 116.310 | 4993.684 | 115.292 | 3777.211 | 116.293 | 3460.585 |
| 116.484 | 5007.184 | 115.465 | 3794.013 | 116.461 | 3467.158 |
| 116.658 | 5018.039 | 115.637 | 3800.552 | 116.628 | 3476.454 |
| 116.832 | 5028.557 | 115.810 | 3811.226 | 116.796 | 3488.095 |
| 117.006 | 5039.254 | 115.983 | 3820.162 | 116.964 | 3492.004 |
| 117.180 | 5054.464 | 116.155 | 3834.249 | 117.131 | 3507.405 |
| 117.354 | 5063.995 | 116.328 | 3843.842 | 117.299 | 3523.295 |
| 117.528 | 5076.691 | 116.501 | 3856.528 | 117.467 | 3528.040 |
| 117.702 | 5091.565 | 116.673 | 3867.723 | 117.635 | 3539.130 |
| 117.876 | 5102.362 | 116.846 | 3878.819 | 117.802 | 3543.638 |
| 118.050 | 5112.226 | 117.019 | 3886.633 | 117.970 | 3556.426 |
| 118.224 | 5128.545 | 117.192 | 3896.649 | 118.138 | 3562.595 |
| 118.398 | 5163.077 | 117.364 | 3903.324 | 118.305 | 3577.851 |
| 118.572 | 5162.821 | 117.537 | 3910.581 | 118.473 | 3584.179 |
| 118.746 | 5157.589 | 117.710 | 3918.970 | 118.641 | 3591.245 |
| 118.920 | 5185.950 | 117.882 | 3933.612 | 118.809 | 3597.289 |
| 119.094 | 5183.378 | 118.055 | 3944.521 | 118.976 | 3603.207 |
| 119.268 | 5212.550 | 118.228 | 3952.429 | 119.144 | 3610.046 |
| 119.442 | 5252.755 | 118.401 | 3962.029 | 119.312 | 3621.339 |
| 119.616 | 5256.893 | 118.573 | 3973.282 | 119.479 | 3631.673 |
| 119.790 | 5274.523 | 118.746 | 3983.374 | 119.647 | 3640.319 |
| 119.965 | 5288.369 | 118.919 | 3988.672 | 119.815 | 3654.336 |
| 120.139 | 5303.454 | 119.091 | 3998.101 | 119.982 | 3662.399 |
| 120.313 | 5315.176 | 119.264 | 4006.452 | 120.150 | 3666.818 |
| 120.487 | 5327.088 | 119.437 | 4014.244 | 120.318 | 3671.142 |
| 120.661 | 5338.568 | 119.609 | 4025.735 | 120.486 | 3676.177 |
| 120.835 | 5360.380 | 119.782 | 4037.778 | 120.653 | 3682.222 |
| 121.009 | 5370.445 | 119.955 | 4046.878 | 120.821 | 3689.319 |
| 121.183 | 5387.882 | 120.128 | 4054.089 | 120.989 | 3694.191 |
| 121.357 | 5397.981 | 120.300 | 4059.287 | 121.156 | 3704.941 |
| 121.531 | 5416.397 | 120.473 | 4066.779 | 121.324 | 3716.636 |
| 121.705 | 5429.798 | 120.646 | 4086.823 | 121.492 | 3728.412 |
| 121.879 | 5440.806 | 120.818 | 4099.081 | 121.660 | 3741.318 |
| 122.053 | 5452.085 | 120.991 | 4109.809 | 121.827 | 3737.967 |
| 122.227 | 5470.311 | 121.164 | 4121.414 | 121.995 | 3751.228 |
| 122.401 | 5489.152 | 121.337 | 4131.359 | 122.163 | 3767.253 |
| 122.575 | 5504.221 | 121.509 | 4142.137 | 122.330 | 3782.102 |
| 122.749 | 5516.694 | 121.682 | 4152.118 | 122.498 | 3790.415 |
| 122.923 | 5519.877 | 121.855 | 4161.458 | 122.666 | 3802.254 |
| 123.097 | 5535.723 | 122.027 | 4169.981 | 122.834 | 3805.576 |
| 123.271 | 5546.724 | 122.200 | 4178.082 | 123.001 | 3811.774 |
| 123.445 | 5563.363 | 122.373 | 4185.692 | 123.169 | 3826.953 |
| 123.620 | 5577.085 | 122.545 | 4193.970 | 123.337 | 3835.161 |
| 123.794 | 5589.089 | 122.718 | 4205.785 | 123.504 | 3842.595 |
| 123.968 | 5605.214 | 122.891 | 4213.349 | 123.672 | 3856.389 |
| 124.142 | 5617.018 | 123.064 | 4223.351 | 123.840 | 3865.427 |
| 124.316 | 5623.706 | 123.236 | 4230.106 | 124.007 | 3873.941 |
| 124.490 | 5634.032 | 123.409 | 4241.570 | 124.175 | 3881.857 |
| 124.664 | 5647.228 | 123.582 | 4249.510 | 124.343 | 3882.783 |
| 124.838 | 5660.396 | 123.754 | 4259.917 | 124.511 | 3888.472 |
| 125.012 | 5673.701 | 123.927 | 4270.117 | 124.678 | 3899.522 |
| 125.186 | 5690.945 | 124.100 | 4282.906 | 124.846 | 3914.581 |
| 125.360 | 5699.699 | 124.273 | 4293.398 | 125.014 | 3919.547 |
| 125.534 | 5717.532 | 124.445 | 4304.429 | 125.181 | 3924.512 |
| 125.708 | 5727.658 | 124.618 | 4315.785 | 125.349 | 3934.588 |
| 125.882 | 5743.611 | 124.791 | 4323.624 | 125.517 | 3949.272 |
| 126.056 | 5760.962 | 124.963 | 4335.667 | 125.685 | 3957.897 |
| 126.230 | 5778.606 | 125.136 | 4344.618 | 125.852 | 3963.827 |
| 126.404 | 5780.237 | 125.309 | 4355.162 | 126.020 | 3974.849 |
| 126.578 | 5802.779 | 125.481 | 4361.023 | 126.188 | 3986.772 |
| 126.752 | 5818.120 | 125.654 | 4374.208 | 126.355 | 3997.908 |
| 126.926 | 5830.861 | 125.827 | 4389.236 | 126.523 | 4008.859 |
| 127.101 | 5842.554 | 126.000 | 4393.739 | 126.691 | 4017.462 |
| 127.275 | 5849.288 | 126.172 | 4409.835 | 126.859 | 4028.192 |
| 127.449 | 5857.679 | 126.345 | 4422.147 | 127.026 | 4034.681 |
| 127.623 | 5881.447 | 126.518 | 4435.816 | 127.194 | 4041.420 |
| 127.797 | 5883.111 | 126.690 | 4447.004 | 127.362 | 4059.443 |
| 127.971 | 5895.690 | 126.863 | 4455.143 | 127.529 | 4073.196 |
| 128.145 | 5906.190 | 127.036 | 4465.775 | 127.697 | 4086.301 |
| 128.319 | 5914.068 | 127.209 | 4480.787 | 127.865 | 4093.402 |
| 128.493 | 5924.186 | 127.381 | 4492.220 | 128.032 | 4101.032 |
| 128.667 | 5927.847 | 127.554 | 4507.494 | 128.200 | 4113.361 |
| 128.841 | 5937.467 | 127.727 | 4515.982 | 128.368 | 4123.570 |
| 129.015 | 5948.600 | 127.899 | 4521.246 | 128.536 | 4131.480 |
| 129.189 | 5968.212 | 128.072 | 4535.338 | 128.703 | 4143.559 |
| 129.363 | 6015.030 | 128.245 | 4548.587 | 128.871 | 4149.962 |
| 129.537 | 6006.978 | 128.418 | 4557.953 | 129.039 | 4157.771 |
| 129.711 | 6027.429 | 128.590 | 4567.116 | 129.206 | 4167.740 |
| 129.885 | 6043.126 | 128.763 | 4575.920 | 129.374 | 4175.590 |
| 130.059 | 6068.416 | 128.936 | 4583.637 | 129.542 | 4187.516 |
| 130.233 | 6069.468 | 129.108 | 4594.391 | 129.710 | 4187.514 |
| 130.407 | 6102.164 | 129.281 | 4604.824 | 129.877 | 4200.250 |
| 130.581 | 6129.751 | 129.454 | 4613.816 | 130.045 | 4217.548 |
| 130.756 | 6143.180 | 129.626 | 4622.470 | 130.213 | 4224.990 |
| 130.930 | 6155.412 | 129.799 | 4632.190 | 130.380 | 4230.390 |
| 131.104 | 6159.073 | 129.972 | 4640.457 | 130.548 | 4242.021 |
| 131.278 | 6175.902 | 130.145 | 4650.172 | 130.716 | 4263.214 |
| 131.452 | 6191.276 | 130.317 | 4666.357 | 130.884 | 4261.794 |
| 131.626 | 6206.205 | 130.490 | 4676.458 | 131.051 | 4310.531 |
| 131.800 | 6218.979 | 130.663 | 4685.549 | 131.219 | 4291.876 |
| 131.974 | 6232.449 | 130.835 | 4698.742 | 131.387 | 4292.202 |
| 132.148 | 6244.593 | 131.008 | 4707.787 | 131.554 | 4300.669 |
| 132.322 | 6260.587 | 131.181 | 4722.263 | 131.722 | 4308.547 |
| 132.496 | 6276.510 | 131.354 | 4737.034 | 131.890 | 4342.733 |
| 132.670 | 6288.755 | 131.526 | 4749.858 | 132.057 | 4340.220 |
| 132.844 | 6309.699 | 131.699 | 4763.597 | 132.225 | 4371.416 |
| 133.018 | 6317.675 | 131.872 | 4770.330 | 132.393 | 4393.743 |
| 133.192 | 6342.343 | 132.044 | 4779.267 | 132.561 | 4403.400 |
| 133.366 | 6363.764 | 132.217 | 4789.840 | 132.728 | 4412.310 |
| 133.540 | 6376.804 | 132.390 | 4799.570 | 132.896 | 4420.453 |
| 133.714 | 6383.268 | 132.562 | 4812.385 | 133.064 | 4427.558 |
| 133.888 | 6401.849 | 132.735 | 4826.850 | 133.231 | 4437.037 |
| 134.062 | 6418.463 | 132.908 | 4838.933 | 133.399 | 4444.513 |
| 134.236 | 6431.011 | 133.081 | 4850.197 | 133.567 | 4459.688 |
| 134.411 | 6460.418 | 133.253 | 4860.004 | 133.735 | 4472.191 |
| 134.585 | 6468.623 | 133.426 | 4868.200 | 133.902 | 4482.448 |
| 134.759 | 6480.295 | 133.599 | 4877.879 | 134.070 | 4481.655 |
| 134.933 | 6495.827 | 133.771 | 4887.530 | 134.238 | 4499.044 |
| 135.107 | 6521.510 | 133.944 | 4897.474 | 134.405 | 4505.948 |
| 135.281 | 6538.155 | 134.117 | 4909.533 | 134.573 | 4518.245 |
| 135.455 | 6550.096 | 134.290 | 4916.556 | 134.741 | 4522.248 |
| 135.629 | 6568.141 | 134.462 | 4929.973 | 134.909 | 4543.067 |
| 135.803 | 6585.383 | 134.635 | 4943.082 | 135.076 | 4554.228 |
| 135.977 | 6594.612 | 134.808 | 4956.278 | 135.244 | 4562.580 |
| 136.151 | 6606.345 | 134.980 | 4966.537 | 135.412 | 4570.288 |
| 136.325 | 6623.480 | 135.153 | 4982.946 | 135.579 | 4574.173 |
| 136.499 | 6636.500 | 135.326 | 4997.240 | 135.747 | 4588.381 |
| 136.673 | 6656.393 | 135.498 | 5008.187 | 135.915 | 4597.695 |
| 136.847 | 6664.970 | 135.671 | 5018.659 | 136.082 | 4608.092 |
| 137.021 | 6677.309 | 135.844 | 5028.944 | 136.250 | 4610.099 |
| 137.195 | 6693.127 | 136.017 | 5040.286 | 136.418 | 4619.763 |
| 137.369 | 6717.823 | 136.189 | 5047.874 | 136.586 | 4631.111 |
| 137.543 | 6732.737 | 136.362 | 5062.603 | 136.753 | 4642.022 |
| 137.717 | 6745.498 | 136.535 | 5070.445 | 136.921 | 4652.370 |
| 137.891 | 6754.224 | 136.707 | 5085.622 | 137.089 | 4665.358 |
| 138.066 | 6767.755 | 136.880 | 5098.146 | 137.256 | 4681.549 |
| 138.240 | 6779.759 | 137.053 | 5111.299 | 137.424 | 4675.278 |
| 138.414 | 6809.878 | 137.226 | 5120.652 | 137.592 | 4687.041 |
| 138.588 | 6819.557 | 137.398 | 5132.077 | 137.760 | 4700.343 |
| 138.762 | 6832.955 | 137.571 | 5139.417 | 137.927 | 4719.049 |
| 138.936 | 6856.631 | 137.744 | 5151.890 | 138.095 | 4741.101 |
| 139.110 | 6869.362 | 137.916 | 5167.150 | 138.263 | 4748.712 |
| 139.284 | 6874.326 | 138.089 | 5176.907 | 138.430 | 4746.471 |
| 139.458 | 6906.382 | 138.262 | 5182.622 | 138.598 | 4760.067 |
| 139.632 | 6912.275 | 138.434 | 5195.169 | 138.766 | 4767.281 |
| 139.806 | 6924.614 | 138.607 | 5205.638 | 138.934 | 4780.249 |
| 139.980 | 6940.946 | 138.780 | 5216.365 | 139.101 | 4791.016 |
| 140.154 | 6955.024 | 138.953 | 5227.896 | 139.269 | 4800.451 |
| 140.328 | 6969.047 | 139.125 | 5238.041 | 139.437 | 4810.922 |
| 140.502 | 6987.439 | 139.298 | 5248.068 | 139.604 | 4823.167 |
| 140.676 | 6989.577 | 139.471 | 5257.692 | 139.772 | 4837.384 |
| 140.850 | 7022.918 | 139.643 | 5269.384 | 139.940 | 4843.028 |
| 141.024 | 7037.527 | 139.816 | 5278.463 | 140.108 | 4850.046 |
| 141.198 | 7051.054 | 139.989 | 5293.524 | 140.275 | 4859.960 |
| 141.372 | 7066.359 | 140.162 | 5304.766 | 140.443 | 4868.271 |
| 141.546 | 7085.608 | 140.334 | 5317.814 | 140.611 | 4878.946 |
| 141.721 | 7098.832 | 140.507 | 5333.463 | 140.778 | 4877.251 |
| 141.895 | 7101.671 | 140.680 | 5345.380 | 140.946 | 4893.542 |
| 142.069 | 7127.162 | 140.852 | 5355.041 | 141.114 | 4908.688 |
| 142.243 | 7131.567 | 141.025 | 5371.933 | 141.281 | 4925.874 |
| 142.417 | 7157.854 | 141.198 | 5382.237 | 141.449 | 4933.078 |
| 142.591 | 7170.899 | 141.370 | 5392.192 | 141.617 | 4937.048 |
| 142.765 | 7195.170 | 141.543 | 5407.157 | 141.785 | 4946.390 |
| 142.939 | 7214.539 | 141.716 | 5419.560 | 141.952 | 4954.290 |
| 143.113 | 7228.305 | 141.889 | 5430.242 | 142.120 | 4970.816 |
| 143.287 | 7233.501 | 142.061 | 5439.506 | 142.288 | 4979.702 |
| 143.461 | 7246.273 | 142.234 | 5449.461 | 142.455 | 4999.087 |
| 143.635 | 7253.715 | 142.407 | 5462.098 | 142.623 | 5005.224 |
| 143.809 | 7270.387 | 142.579 | 5471.282 | 142.791 | 5011.425 |
| 143.983 | 7290.170 | 142.752 | 5479.854 | 142.959 | 5018.309 |
| 144.157 | 7314.123 | 142.925 | 5494.022 | 143.126 | 5026.961 |
| 144.331 | 7337.265 | 143.098 | 5505.983 | 143.294 | 5042.873 |
| 144.505 | 7350.178 | 143.270 | 5516.882 | 143.462 | 5060.269 |
| 144.679 | 7357.887 | 143.443 | 5524.433 | 143.629 | 5070.537 |
| 144.853 | 7373.013 | 143.616 | 5536.805 | 143.797 | 5080.865 |
| 145.027 | 7388.543 | 143.788 | 5543.397 | 143.965 | 5089.148 |
| 145.201 | 7402.185 | 143.961 | 5559.783 | 144.133 | 5095.425 |
| 145.376 | 7423.691 | 144.134 | 5574.777 | 144.300 | 5105.413 |
| 145.550 | 7433.464 | 144.306 | 5590.506 | 144.468 | 5116.929 |
| 145.724 | 7449.614 | 144.479 | 5600.865 | 144.636 | 5130.020 |
| 145.898 | 7465.335 | 144.652 | 5614.083 | 144.803 | 5141.493 |
| 146.072 | 7479.641 | 144.825 | 5618.879 | 144.971 | 5152.337 |
| 146.246 | 7497.073 | 144.997 | 5630.667 | 145.139 | 5158.110 |
| 146.420 | 7502.207 | 145.170 | 5646.310 | 145.306 | 5165.621 |
| 146.594 | 7513.458 | 145.343 | 5661.806 | 145.474 | 5173.231 |
| 146.768 | 7527.002 | 145.515 | 5678.244 | 145.642 | 5182.767 |
| 146.942 | 7546.633 | 145.688 | 5684.671 | 145.810 | 5193.131 |
| 147.116 | 7566.553 | 145.861 | 5695.735 | 145.977 | 5202.648 |
| 147.290 | 7583.758 | 146.034 | 5709.147 | 146.145 | 5221.613 |
| 147.464 | 7599.575 | 146.206 | 5723.321 | 146.313 | 5226.505 |
| 147.638 | 7617.989 | 146.379 | 5735.475 | 146.480 | 5234.477 |
| 147.812 | 7636.232 | 146.552 | 5749.539 | 146.648 | 5253.549 |
| 147.986 | 7655.713 | 146.724 | 5759.787 | 146.816 | 5266.533 |
| 148.160 | 7671.731 | 146.897 | 5770.765 | 146.984 | 5278.661 |
| 148.334 | 7687.074 | 147.070 | 5779.347 | 147.151 | 5298.898 |
| 148.508 | 7699.056 | 147.242 | 5785.513 | 147.319 | 5312.955 |
| 148.682 | 7716.976 | 147.415 | 5801.889 | 147.487 | 5324.248 |
| 148.856 | 7732.412 | 147.588 | 5818.367 | 147.654 | 5332.021 |
| 149.031 | 7756.658 | 147.761 | 5828.310 | 147.822 | 5339.122 |
| 149.205 | 7757.615 | 147.933 | 5843.359 | 147.990 | 5340.302 |
| 149.379 | 7770.712 | 148.106 | 5855.683 | 148.158 | 5355.621 |
| 149.553 | 7789.796 | 148.279 | 5864.973 | 148.325 | 5369.821 |
| 149.727 | 7807.947 | 148.451 | 5876.393 | 148.493 | 5381.375 |
| 149.901 | 7824.583 | 148.624 | 5899.239 | 148.661 | 5390.462 |
| 150.075 | 7831.719 | 148.797 | 5904.515 | 148.828 | 5403.697 |
| 150.249 | 7842.331 | 148.970 | 5916.142 | 148.996 | 5413.556 |
| 150.423 | 7864.481 | 149.142 | 5929.583 | 149.164 | 5422.443 |
| 150.597 | 7877.880 | 149.315 | 5944.326 | 149.331 | 5424.427 |
| 150.771 | 7887.270 | 149.488 | 5957.690 | 149.499 | 5442.463 |
| 150.945 | 7895.165 | 149.660 | 5969.958 | 149.667 | 5457.043 |
| 151.119 | 7913.042 | 149.833 | 5979.667 | 149.835 | 5457.756 |
| 151.293 | 7920.490 | 150.006 | 5992.235 | 150.002 | 5483.395 |
| 151.467 | 7943.049 | 150.178 | 6001.924 | 150.170 | 5498.632 |
| 151.641 | 7969.216 | 150.351 | 6014.045 | 150.338 | 5509.095 |
| 151.815 | 7984.879 | 150.524 | 6026.493 | 150.505 | 5523.149 |
| 151.989 | 7998.630 | 150.697 | 6040.479 | 150.673 | 5527.414 |
| 152.163 | 8021.622 | 150.869 | 6052.628 | 150.841 | 5536.005 |
| 152.337 | 8037.443 | 151.042 | 6066.352 | 151.009 | 5553.321 |
| 152.512 | 8055.482 | 151.215 | 6076.751 | 151.176 | 5571.768 |
| 152.686 | 8070.955 | 151.387 | 6090.802 | 151.344 | 5584.823 |
| 152.860 | 8081.896 | 151.560 | 6101.370 | 151.512 | 5595.892 |
| 153.034 | 8100.015 | 151.733 | 6111.455 | 151.679 | 5603.478 |
| 153.208 | 8116.268 | 151.906 | 6129.770 | 151.847 | 5610.200 |
| 153.382 | 8133.499 | 152.078 | 6143.765 | 152.015 | 5629.146 |
| 153.556 | 8143.918 | 152.251 | 6152.673 | 152.183 | 5638.092 |
| 153.730 | 8154.117 | 152.424 | 6166.653 | 152.350 | 5651.237 |
| 153.904 | 8170.124 | 152.596 | 6180.837 | 152.518 | 5667.444 |
| 154.078 | 8184.394 | 152.769 | 6186.726 | 152.686 | 5672.011 |
| 154.252 | 8207.316 | 152.942 | 6200.608 | 152.853 | 5689.175 |
| 154.426 | 8218.557 | 153.114 | 6214.204 | 153.021 | 5703.664 |
| 154.600 | 8239.377 | 153.287 | 6225.688 | 153.189 | 5712.490 |
| 154.774 | 8245.576 | 153.460 | 6236.300 | 153.356 | 5723.457 |
| 154.948 | 8267.517 | 153.633 | 6250.524 | 153.524 | 5725.681 |
| 155.122 | 8288.443 | 153.805 | 6260.974 | 153.692 | 5750.651 |
| 155.296 | 8310.479 | 153.978 | 6279.123 | 153.860 | 5759.336 |
| 155.470 | 8320.162 | 154.151 | 6293.009 | 154.027 | 5764.071 |
| 155.644 | 8332.543 | 154.323 | 6307.659 | 154.195 | 5768.807 |
| 155.818 | 8348.481 | 154.496 | 6320.583 | 154.363 | 5781.235 |
| 155.992 | 8366.851 | 154.669 | 6332.770 | 154.530 | 5791.625 |
| 156.167 | 8383.881 | 154.842 | 6349.807 | 154.698 | 5807.939 |
| 156.341 | 8417.943 | 155.014 | 6359.784 | 154.866 | 5820.333 |
| 156.515 | 8445.753 | 155.187 | 6370.234 | 155.034 | 5836.528 |
| 156.689 | 8442.814 | 155.360 | 6382.366 | 155.201 | 5843.917 |
| 156.863 | 8461.380 | 155.532 | 6402.311 | 155.369 | 5853.832 |
| 157.037 | 8485.816 | 155.705 | 6416.123 | 155.537 | 5857.621 |
| 157.211 | 8509.254 | 155.878 | 6429.069 | 155.704 | 5867.608 |
| 157.385 | 8525.907 | 156.051 | 6442.843 | 155.872 | 5883.965 |
| 157.559 | 8546.553 | 156.223 | 6452.883 | 156.040 | 5884.689 |
| 157.733 | 8566.631 | 156.396 | 6463.655 | 156.208 | 5896.452 |
| 157.907 | 8581.745 | 156.569 | 6475.259 | 156.375 | 5908.511 |
| 158.081 | 8597.894 | 156.741 | 6489.906 | 156.543 | 5912.976 |
| 158.255 | 8617.186 | 156.914 | 6507.075 | 156.711 | 5919.631 |
| 158.429 | 8629.755 | 157.087 | 6518.731 | 156.878 | 5931.259 |
| 158.603 | 8646.620 | 157.259 | 6535.119 | 157.046 | 5945.324 |
| 158.777 | 8667.492 | 157.432 | 6550.482 | 157.214 | 5959.425 |
| 158.951 | 8684.119 | 157.605 | 6565.008 | 157.381 | 5974.442 |
| 159.125 | 8708.789 | 157.778 | 6577.326 | 157.549 | 5989.154 |
| 159.299 | 8718.357 | 157.950 | 6591.145 | 157.717 | 6002.249 |
| 159.473 | 8716.034 | 158.123 | 6604.070 | 157.885 | 6011.571 |
| 159.647 | 8724.675 | 158.296 | 6613.422 | 158.052 | 6019.339 |
| 159.822 | 8756.812 | 158.468 | 6626.002 | 158.220 | 6029.741 |
| 159.996 | 8811.942 | 158.641 | 6635.403 | 158.388 | 6043.671 |
| 160.170 | 8813.407 | 158.814 | 6651.213 | 158.555 | 6063.088 |
| 160.344 | 8817.943 | 158.987 | 6661.837 | 158.723 | 6071.187 |
| 160.518 | 8835.716 | 159.159 | 6672.590 | 158.891 | 6080.054 |
| 160.692 | 8873.759 | 159.332 | 6681.013 | 159.059 | 6091.812 |
| 160.866 | 8853.037 | 159.505 | 6693.345 | 159.226 | 6102.636 |
| 161.040 | 8817.047 | 159.677 | 6709.753 | 159.394 | 6112.000 |
| 161.214 | 8823.575 | 159.850 | 6714.848 | 159.562 | 6127.487 |
| 161.179 | 8745.635 | 160.023 | 6729.526 | 159.729 | 6139.037 |
| 161.179 | 8745.635 | 160.195 | 6741.765 | 159.897 | 6161.229 |
| 161.051 | 8750.897 | 160.368 | 6754.727 | 160.065 | 6173.649 |
| 160.922 | 8765.264 | 160.541 | 6764.996 | 160.233 | 6196.123 |
| 160.794 | 8720.545 | 160.714 | 6777.771 | 160.400 | 6200.952 |
| 160.666 | 8708.889 | 160.886 | 6789.064 | 160.568 | 6218.881 |
| 160.537 | 8702.063 | 161.059 | 6807.595 | 160.736 | 6230.274 |
| 160.409 | 8712.211 | 161.232 | 6827.670 | 160.903 | 6242.200 |
| 160.281 | 8624.458 | 161.404 | 6828.698 | 161.071 | 6243.661 |
| 160.152 | 8581.774 | 161.577 | 6834.715 | 161.071 | 6243.661 |
| 160.024 | 8554.512 | 161.750 | 6849.952 | 161.660 | 6217.739 |
| 159.895 | 8527.277 | 161.923 | 6857.779 | 161.549 | 6194.458 |
| 159.767 | 8504.222 | 162.095 | 6865.419 | 161.438 | 6210.695 |
| 159.639 | 8480.764 | 162.268 | 6867.280 | 161.327 | 6171.154 |
| 159.510 | 8453.096 | 162.441 | 6867.668 | 161.216 | 6156.481 |
| 159.382 | 8424.857 | 162.441 | 6867.668 | 161.105 | 6144.221 |
| 159.254 | 8396.870 | 162.624 | 6889.979 | 160.994 | 6154.211 |
| 159.125 | 8372.902 | 162.517 | 6802.916 | 160.883 | 6121.343 |
| 158.997 | 8349.951 | 162.411 | 6787.781 | 160.772 | 6132.070 |
| 158.869 | 8326.226 | 162.304 | 6771.399 | 160.661 | 6098.194 |
| 158.740 | 8303.076 | 162.198 | 6750.863 | 160.550 | 6077.766 |
| 158.612 | 8279.931 | 162.092 | 6747.223 | 160.439 | 6066.255 |
| 158.484 | 8256.794 | 161.985 | 6730.698 | 160.328 | 6054.745 |
| 158.355 | 8233.657 | 161.879 | 6728.170 | 160.217 | 6043.234 |
| 158.227 | 8211.542 | 161.772 | 6710.348 | 160.106 | 6031.788 |
| 158.098 | 8189.997 | 161.666 | 6686.900 | 159.995 | 6019.798 |
| 157.970 | 8168.904 | 161.560 | 6674.298 | 159.884 | 6006.847 |
| 157.842 | 8144.608 | 161.453 | 6669.754 | 159.773 | 5993.896 |
| 157.713 | 8119.425 | 161.347 | 6670.196 | 159.662 | 5980.945 |
| 157.585 | 8093.685 | 161.240 | 6671.554 | 159.551 | 5967.994 |
| 157.457 | 8067.945 | 161.134 | 6679.251 | 159.440 | 5955.043 |
| 157.328 | 8039.907 | 161.027 | 6668.343 | 159.329 | 5938.193 |
| 157.200 | 8010.704 | 160.921 | 6654.819 | 159.218 | 5920.592 |
| 157.072 | 7981.501 | 160.815 | 6642.873 | 159.107 | 5903.021 |
| 156.943 | 7955.538 | 160.708 | 6614.673 | 158.996 | 5888.162 |
| 156.815 | 7926.675 | 160.602 | 6581.451 | 158.885 | 5878.299 |
| 156.687 | 7887.245 | 160.495 | 6567.834 | 158.774 | 5868.436 |
| 156.558 | 7859.606 | 160.389 | 6547.640 | 158.663 | 5858.573 |
| 156.430 | 7835.256 | 160.283 | 6539.795 | 158.552 | 5848.710 |
| 156.301 | 7810.905 | 160.176 | 6525.154 | 158.441 | 5838.847 |
| 156.173 | 7784.700 | 160.070 | 6498.789 | 158.330 | 5829.455 |
| 156.045 | 7757.959 | 159.963 | 6465.272 | 158.219 | 5820.074 |
| 155.916 | 7731.234 | 159.857 | 6449.103 | 158.108 | 5810.318 |
| 155.788 | 7704.838 | 159.751 | 6432.716 | 157.997 | 5799.742 |
| 155.660 | 7676.609 | 159.644 | 6416.071 | 157.886 | 5789.165 |
| 155.531 | 7649.491 | 159.538 | 6399.752 | 157.775 | 5778.589 |
| 155.403 | 7623.625 | 159.431 | 6384.376 | 157.664 | 5768.013 |
| 155.275 | 7597.758 | 159.325 | 6369.300 | 157.553 | 5757.437 |
| 155.146 | 7572.356 | 159.219 | 6354.225 | 157.442 | 5746.513 |
| 155.018 | 7549.357 | 159.112 | 6339.149 | 157.331 | 5735.136 |
| 154.889 | 7526.823 | 159.006 | 6323.772 | 157.220 | 5724.405 |
| 154.761 | 7504.359 | 158.899 | 6308.268 | 157.109 | 5713.749 |
| 154.633 | 7481.827 | 158.793 | 6293.561 | 156.998 | 5703.093 |
| 154.504 | 7459.187 | 158.686 | 6278.286 | 156.887 | 5692.436 |
| 154.376 | 7436.547 | 158.580 | 6263.304 | 156.776 | 5681.780 |
| 154.248 | 7413.270 | 158.474 | 6248.340 | 156.665 | 5671.123 |
| 154.119 | 7390.804 | 158.367 | 6233.376 | 156.554 | 5660.467 |
| 153.991 | 7368.550 | 158.261 | 6218.427 | 156.443 | 5649.810 |
| 153.863 | 7346.296 | 158.154 | 6203.608 | 156.332 | 5638.689 |
| 153.734 | 7323.777 | 158.048 | 6188.103 | 156.221 | 5626.050 |
| 153.606 | 7301.893 | 157.942 | 6171.588 | 156.110 | 5613.412 |
| 153.478 | 7280.194 | 157.835 | 6154.864 | 155.999 | 5600.975 |
| 153.349 | 7258.496 | 157.729 | 6137.217 | 155.888 | 5588.673 |
| 153.221 | 7237.069 | 157.622 | 6119.539 | 155.777 | 5576.370 |
| 153.092 | 7215.659 | 157.516 | 6101.595 | 155.666 | 5564.068 |
| 152.964 | 7193.089 | 157.410 | 6084.947 | 155.555 | 5551.765 |
| 152.836 | 7168.855 | 157.303 | 6069.389 | 155.444 | 5540.817 |
| 152.707 | 7144.051 | 157.197 | 6053.622 | 155.333 | 5531.232 |
| 152.579 | 7117.791 | 157.090 | 6038.323 | 155.222 | 5521.973 |
| 152.451 | 7091.031 | 156.984 | 6023.725 | 155.111 | 5512.714 |
| 152.322 | 7063.848 | 156.877 | 6008.975 | 155.000 | 5503.455 |
| 152.194 | 7037.838 | 156.771 | 5994.159 | 154.889 | 5494.195 |
| 152.066 | 7012.152 | 156.665 | 5979.550 | 154.778 | 5484.936 |
| 151.937 | 6987.715 | 156.558 | 5964.942 | 154.667 | 5475.543 |
| 151.809 | 6964.372 | 156.452 | 5950.333 | 154.556 | 5465.760 |
| 151.680 | 6940.634 | 156.345 | 5934.892 | 154.445 | 5455.977 |
| 151.552 | 6916.651 | 156.239 | 5918.391 | 154.334 | 5446.194 |
| 151.424 | 6892.809 | 156.133 | 5901.193 | 154.223 | 5435.917 |
| 151.295 | 6871.302 | 156.026 | 5884.065 | 154.112 | 5425.468 |
| 151.167 | 6850.898 | 155.920 | 5866.702 | 154.001 | 5415.018 |
| 151.039 | 6830.494 | 155.813 | 5849.136 | 153.890 | 5404.559 |
| 150.910 | 6809.730 | 155.707 | 5832.391 | 153.779 | 5394.097 |
| 150.782 | 6787.805 | 155.601 | 5815.528 | 153.668 | 5383.636 |
| 150.654 | 6766.763 | 155.494 | 5798.475 | 153.557 | 5372.701 |
| 150.525 | 6746.793 | 155.388 | 5781.381 | 153.446 | 5360.786 |
| 150.397 | 6726.823 | 155.281 | 5764.487 | 153.335 | 5348.871 |
| 150.269 | 6706.852 | 155.175 | 5747.537 | 153.224 | 5337.359 |
| 150.140 | 6686.882 | 155.068 | 5730.379 | 153.113 | 5326.271 |
| 150.012 | 6666.922 | 154.962 | 5713.889 | 153.002 | 5315.182 |
| 149.883 | 6646.989 | 154.856 | 5697.164 | 152.891 | 5304.094 |
| 149.755 | 6625.101 | 154.749 | 5680.933 | 152.780 | 5293.005 |
| 149.627 | 6602.468 | 154.643 | 5664.701 | 152.669 | 5283.353 |
| 149.498 | 6579.766 | 154.536 | 5648.469 | 152.558 | 5273.232 |
| 149.370 | 6556.657 | 154.430 | 5632.382 | 152.447 | 5262.733 |
| 149.242 | 6533.159 | 154.324 | 5615.882 | 152.336 | 5252.233 |
| 149.113 | 6509.794 | 154.217 | 5600.234 | 152.225 | 5241.733 |
| 148.985 | 6489.171 | 154.111 | 5585.297 | 152.114 | 5231.234 |
| 148.857 | 6468.660 | 154.004 | 5570.712 | 152.003 | 5220.734 |
| 148.728 | 6448.148 | 153.898 | 5556.125 | 151.892 | 5209.536 |
| 148.600 | 6427.636 | 153.792 | 5541.537 | 151.781 | 5198.191 |
| 148.471 | 6407.125 | 153.685 | 5526.949 | 151.670 | 5186.846 |
| 148.343 | 6386.610 | 153.579 | 5512.232 | 151.559 | 5176.723 |
| 148.215 | 6364.576 | 153.472 | 5497.479 | 151.448 | 5166.728 |
| 148.086 | 6338.899 | 153.366 | 5482.516 | 151.337 | 5155.423 |
| 147.958 | 6313.415 | 153.259 | 5467.106 | 151.226 | 5143.266 |
| 147.830 | 6287.972 | 153.153 | 5451.471 | 151.115 | 5131.109 |
| 147.701 | 6262.528 | 153.047 | 5435.804 | 151.004 | 5118.953 |
| 147.573 | 6236.685 | 152.940 | 5419.469 | 150.892 | 5106.846 |
| 147.445 | 6213.065 | 152.834 | 5403.293 | 150.781 | 5094.777 |
| 147.316 | 6191.157 | 152.727 | 5387.833 | 150.670 | 5082.707 |
| 147.188 | 6168.802 | 152.621 | 5372.373 | 150.559 | 5070.862 |
| 147.060 | 6146.120 | 152.515 | 5356.913 | 150.448 | 5059.483 |
| 146.931 | 6123.439 | 152.408 | 5341.453 | 150.337 | 5048.104 |
| 146.803 | 6100.757 | 152.302 | 5324.343 | 150.226 | 5036.660 |
| 146.674 | 6078.075 | 152.195 | 5306.900 | 150.115 | 5023.846 |
| 146.546 | 6054.473 | 152.089 | 5288.181 | 150.004 | 5011.032 |
| 146.418 | 6030.250 | 151.983 | 5269.481 | 149.893 | 4998.218 |
| 146.289 | 6007.591 | 151.876 | 5250.864 | 149.782 | 4985.969 |
| 146.161 | 5985.305 | 151.770 | 5233.660 | 149.671 | 4973.889 |
| 146.033 | 5963.310 | 151.663 | 5216.114 | 149.560 | 4962.496 |
| 145.904 | 5941.566 | 151.557 | 5200.065 | 149.449 | 4952.113 |
| 145.776 | 5919.822 | 151.451 | 5182.838 | 149.338 | 4941.730 |
| 145.648 | 5898.117 | 151.344 | 5160.049 | 149.227 | 4931.453 |
| 145.519 | 5877.187 | 151.238 | 5138.730 | 149.116 | 4921.235 |
| 145.391 | 5857.694 | 151.131 | 5121.780 | 149.005 | 4911.017 |
| 145.262 | 5838.384 | 151.025 | 5105.450 | 148.894 | 4900.799 |
| 145.134 | 5819.026 | 150.918 | 5089.244 | 148.783 | 4890.582 |
| 145.006 | 5799.294 | 150.812 | 5072.800 | 148.672 | 4880.364 |
| 144.877 | 5779.154 | 150.706 | 5055.988 | 148.561 | 4870.146 |
| 144.749 | 5758.662 | 150.599 | 5039.356 | 148.450 | 4859.531 |
| 144.621 | 5735.958 | 150.493 | 5024.225 | 148.339 | 4848.901 |
| 144.492 | 5713.262 | 150.386 | 5009.094 | 148.228 | 4838.787 |
| 144.364 | 5690.565 | 150.280 | 4993.963 | 148.117 | 4829.229 |
| 144.236 | 5667.752 | 150.174 | 4978.981 | 148.006 | 4818.984 |
| 144.107 | 5644.869 | 150.067 | 4964.051 | 147.895 | 4807.958 |
| 143.979 | 5621.564 | 149.961 | 4949.167 | 147.784 | 4796.933 |
| 143.851 | 5599.848 | 149.854 | 4935.501 | 147.673 | 4785.907 |
| 143.722 | 5578.189 | 149.748 | 4921.943 | 147.562 | 4774.881 |
| 143.594 | 5556.606 | 149.642 | 4908.245 | 147.451 | 4763.856 |
| 143.465 | 5535.619 | 149.535 | 4894.324 | 147.340 | 4752.560 |
| 143.337 | 5514.632 | 149.429 | 4880.403 | 147.229 | 4741.133 |
| 143.209 | 5493.680 | 149.322 | 4866.144 | 147.118 | 4729.705 |
| 143.080 | 5472.958 | 149.216 | 4852.032 | 147.007 | 4718.229 |
| 142.952 | 5451.668 | 149.109 | 4837.954 | 146.896 | 4706.732 |
| 142.824 | 5430.407 | 149.003 | 4823.861 | 146.785 | 4695.297 |
| 142.695 | 5409.860 | 148.897 | 4809.768 | 146.674 | 4683.862 |
| 142.567 | 5389.179 | 148.790 | 4795.324 | 146.563 | 4672.427 |
| 142.439 | 5368.498 | 148.684 | 4779.996 | 146.452 | 4660.991 |
| 142.310 | 5347.638 | 148.577 | 4764.438 | 146.341 | 4649.776 |
| 142.182 | 5327.570 | 148.471 | 4748.359 | 146.230 | 4639.209 |
| 142.053 | 5308.424 | 148.365 | 4732.346 | 146.119 | 4628.643 |
| 141.925 | 5289.109 | 148.258 | 4716.436 | 146.008 | 4618.922 |
| 141.797 | 5269.681 | 148.152 | 4700.545 | 145.897 | 4609.830 |
| 141.668 | 5250.253 | 148.045 | 4685.820 | 145.786 | 4600.738 |
| 141.540 | 5229.379 | 147.939 | 4671.345 | 145.675 | 4591.646 |
| 141.412 | 5207.221 | 147.833 | 4656.870 | 145.564 | 4582.552 |
| 141.283 | 5183.780 | 147.726 | 4643.075 | 145.453 | 4571.880 |
| 141.155 | 5159.385 | 147.620 | 4629.078 | 145.342 | 4561.207 |
| 141.027 | 5134.980 | 147.513 | 4614.909 | 145.231 | 4550.534 |
| 140.898 | 5110.563 | 147.407 | 4600.421 | 145.120 | 4539.861 |
| 140.770 | 5086.917 | 147.300 | 4585.469 | 145.009 | 4529.452 |
| 140.642 | 5064.499 | 147.194 | 4570.060 | 144.898 | 4519.585 |
| 140.513 | 5043.336 | 147.088 | 4554.652 | 144.787 | 4509.719 |
| 140.385 | 5022.976 | 146.981 | 4539.243 | 144.676 | 4500.010 |
| 140.256 | 5002.800 | 146.875 | 4523.795 | 144.565 | 4491.124 |
| 140.128 | 4983.520 | 146.768 | 4508.350 | 144.454 | 4481.680 |
| 140.000 | 4964.241 | 146.662 | 4493.318 | 144.343 | 4472.237 |
| 139.871 | 4945.022 | 146.556 | 4478.774 | 144.232 | 4462.793 |
| 139.743 | 4926.123 | 146.449 | 4464.883 | 144.121 | 4453.349 |
| 139.615 | 4907.284 | 146.343 | 4451.265 | 144.010 | 4443.905 |
| 139.486 | 4888.235 | 146.236 | 4437.743 | 143.899 | 4434.462 |
| 139.358 | 4867.929 | 146.130 | 4424.351 | 143.788 | 4425.018 |
| 139.230 | 4847.877 | 146.024 | 4410.958 | 143.677 | 4415.574 |
| 139.101 | 4827.824 | 145.917 | 4397.565 | 143.566 | 4406.131 |
| 138.973 | 4807.771 | 145.811 | 4384.172 | 143.455 | 4397.257 |
| 138.844 | 4787.293 | 145.704 | 4370.147 | 143.344 | 4387.625 |
| 138.716 | 4767.279 | 145.598 | 4356.119 | 143.233 | 4376.772 |
| 138.588 | 4748.039 | 145.491 | 4341.967 | 143.122 | 4365.919 |
| 138.459 | 4728.799 | 145.385 | 4327.584 | 143.011 | 4355.066 |
| 138.331 | 4709.562 | 145.279 | 4312.875 | 142.900 | 4344.212 |
| 138.203 | 4690.329 | 145.172 | 4297.239 | 142.789 | 4333.359 |
| 138.074 | 4670.836 | 145.066 | 4281.519 | 142.678 | 4322.506 |
| 137.946 | 4650.826 | 144.959 | 4265.829 | 142.567 | 4311.653 |
| 137.818 | 4632.170 | 144.853 | 4250.609 | 142.456 | 4301.560 |
| 137.689 | 4614.198 | 144.747 | 4235.390 | 142.345 | 4292.490 |
| 137.561 | 4596.227 | 144.640 | 4220.170 | 142.234 | 4283.318 |
| 137.433 | 4578.255 | 144.534 | 4204.837 | 142.123 | 4274.132 |
| 137.304 | 4560.283 | 144.427 | 4188.979 | 142.012 | 4264.946 |
| 137.176 | 4542.171 | 144.321 | 4173.444 | 141.901 | 4255.285 |
| 137.047 | 4523.866 | 144.215 | 4158.122 | 141.790 | 4245.281 |
| 136.919 | 4506.099 | 144.108 | 4142.817 | 141.679 | 4235.278 |
| 136.791 | 4488.932 | 144.002 | 4127.953 | 141.568 | 4225.275 |
| 136.662 | 4471.674 | 143.895 | 4113.210 | 141.457 | 4215.272 |
| 136.534 | 4454.417 | 143.789 | 4098.810 | 141.346 | 4205.269 |
| 136.406 | 4437.160 | 143.682 | 4084.869 | 141.235 | 4195.433 |
| 136.277 | 4419.903 | 143.576 | 4070.928 | 141.124 | 4186.076 |
| 136.149 | 4402.380 | 143.470 | 4056.987 | 141.013 | 4176.481 |
| 136.021 | 4385.643 | 143.363 | 4043.449 | 140.902 | 4166.615 |
| 135.892 | 4368.715 | 143.257 | 4030.556 | 140.791 | 4156.505 |
| 135.764 | 4351.198 | 143.150 | 4017.704 | 140.680 | 4146.139 |
| 135.635 | 4333.661 | 143.044 | 4004.611 | 140.569 | 4135.773 |
| 135.507 | 4315.951 | 142.938 | 3991.494 | 140.458 | 4125.406 |
| 135.379 | 4298.242 | 142.831 | 3978.444 | 140.347 | 4115.040 |
| 135.250 | 4280.532 | 142.725 | 3965.348 | 140.236 | 4104.673 |
| 135.122 | 4262.822 | 142.618 | 3951.971 | 140.125 | 4094.307 |
| 134.994 | 4245.112 | 142.512 | 3938.472 | 140.013 | 4083.940 |
| 134.865 | 4227.091 | 142.406 | 3924.550 | 139.902 | 4073.182 |
| 134.737 | 4208.812 | 142.299 | 3910.629 | 139.791 | 4061.332 |
| 134.609 | 4189.994 | 142.193 | 3896.852 | 139.680 | 4049.995 |
| 134.480 | 4170.802 | 142.086 | 3882.936 | 139.569 | 4038.659 |
| 134.352 | 4150.615 | 141.980 | 3868.843 | 139.458 | 4027.322 |
| 134.224 | 4130.428 | 141.874 | 3854.674 | 139.347 | 4015.985 |
| 134.095 | 4110.241 | 141.767 | 3840.784 | 139.236 | 4004.648 |
| 133.967 | 4090.054 | 141.661 | 3827.059 | 139.125 | 3993.311 |
| 133.838 | 4070.179 | 141.554 | 3812.401 | 139.014 | 3982.966 |
| 133.710 | 4050.861 | 141.448 | 3797.808 | 138.903 | 3973.429 |
| 133.582 | 4032.675 | 141.341 | 3783.216 | 138.792 | 3963.685 |
| 133.453 | 4015.438 | 141.235 | 3768.623 | 138.681 | 3953.942 |
| 133.325 | 3998.089 | 141.129 | 3754.392 | 138.570 | 3944.198 |
| 133.197 | 3980.741 | 141.022 | 3740.324 | 138.459 | 3934.455 |
| 133.068 | 3963.392 | 140.916 | 3726.467 | 138.348 | 3923.761 |
| 132.940 | 3946.044 | 140.809 | 3713.441 | 138.237 | 3912.871 |
| 132.812 | 3928.436 | 140.703 | 3699.759 | 138.126 | 3901.981 |
| 132.683 | 3910.555 | 140.597 | 3685.300 | 138.015 | 3891.090 |
| 132.555 | 3892.537 | 140.490 | 3671.033 | 137.904 | 3880.200 |
| 132.426 | 3874.999 | 140.384 | 3657.221 | 137.793 | 3869.713 |
| 132.298 | 3858.561 | 140.277 | 3643.380 | 137.682 | 3859.548 |
| 132.170 | 3841.573 | 140.171 | 3629.496 | 137.571 | 3849.578 |
| 132.041 | 3824.527 | 140.065 | 3615.623 | 137.460 | 3839.731 |
| 131.913 | 3808.164 | 139.958 | 3602.539 | 137.349 | 3830.730 |
| 131.785 | 3792.588 | 139.852 | 3589.530 | 137.238 | 3821.728 |
| 131.656 | 3777.438 | 139.745 | 3576.519 | 137.127 | 3812.726 |
| 131.528 | 3762.648 | 139.639 | 3563.577 | 137.016 | 3803.724 |
| 131.400 | 3747.859 | 139.532 | 3550.715 | 136.905 | 3794.723 |
| 131.271 | 3733.069 | 139.426 | 3537.852 | 136.794 | 3785.721 |
| 131.143 | 3718.157 | 139.320 | 3524.675 | 136.683 | 3776.694 |
| 131.015 | 3703.070 | 139.213 | 3511.244 | 136.572 | 3767.590 |
| 130.886 | 3687.393 | 139.107 | 3497.121 | 136.461 | 3758.486 |
| 130.758 | 3671.715 | 139.000 | 3483.571 | 136.350 | 3749.383 |
| 130.629 | 3656.038 | 138.894 | 3470.451 | 136.239 | 3739.618 |
| 130.501 | 3640.361 | 138.788 | 3457.332 | 136.128 | 3729.534 |
| 130.373 | 3624.512 | 138.681 | 3444.260 | 136.017 | 3719.511 |
| 130.244 | 3608.564 | 138.575 | 3431.190 | 135.906 | 3709.489 |
| 130.116 | 3593.267 | 138.468 | 3419.351 | 135.795 | 3699.467 |
| 129.988 | 3578.342 | 138.362 | 3407.838 | 135.684 | 3689.444 |
| 129.859 | 3563.061 | 138.256 | 3395.473 | 135.573 | 3679.422 |
| 129.731 | 3547.781 | 138.149 | 3382.998 | 135.462 | 3669.873 |
| 129.603 | 3531.521 | 138.043 | 3370.534 | 135.351 | 3660.336 |
| 129.474 | 3515.062 | 137.936 | 3358.224 | 135.240 | 3650.325 |
| 129.346 | 3498.602 | 137.830 | 3345.914 | 135.129 | 3639.588 |
| 129.217 | 3482.021 | 137.723 | 3333.602 | 135.018 | 3628.852 |
| 129.089 | 3465.067 | 137.617 | 3321.220 | 134.907 | 3618.115 |
| 128.961 | 3448.710 | 137.511 | 3308.497 | 134.796 | 3606.933 |
| 128.832 | 3432.269 | 137.404 | 3295.426 | 134.685 | 3595.453 |
| 128.704 | 3416.478 | 137.298 | 3282.744 | 134.574 | 3583.973 |
| 128.576 | 3400.825 | 137.191 | 3270.157 | 134.463 | 3572.615 |
| 128.447 | 3384.591 | 137.085 | 3257.616 | 134.352 | 3563.126 |
| 128.319 | 3368.358 | 136.979 | 3245.287 | 134.241 | 3553.685 |
| 128.191 | 3351.782 | 136.872 | 3233.083 | 134.130 | 3544.345 |
| 128.062 | 3335.048 | 136.766 | 3220.879 | 134.019 | 3535.004 |
| 127.934 | 3318.279 | 136.659 | 3209.083 | 133.908 | 3525.664 |
| 127.806 | 3301.923 | 136.553 | 3197.337 | 133.797 | 3515.918 |
| 127.677 | 3286.033 | 136.447 | 3184.883 | 133.686 | 3505.710 |
| 127.549 | 3270.134 | 136.340 | 3172.360 | 133.575 | 3495.502 |
| 127.420 | 3254.136 | 136.234 | 3159.984 | 133.464 | 3485.294 |
| 127.292 | 3238.816 | 136.127 | 3147.704 | 133.353 | 3475.086 |
| 127.164 | 3223.495 | 136.021 | 3135.235 | 133.242 | 3464.878 |
| 127.035 | 3208.604 | 135.914 | 3122.780 | 133.131 | 3454.658 |
| 126.907 | 3193.902 | 135.808 | 3110.062 | 133.020 | 3444.115 |
| 126.779 | 3179.200 | 135.702 | 3096.848 | 132.909 | 3435.006 |
| 126.650 | 3164.498 | 135.595 | 3084.415 | 132.798 | 3425.381 |
| 126.522 | 3149.986 | 135.489 | 3072.441 | 132.687 | 3415.756 |
| 126.394 | 3135.263 | 135.382 | 3060.490 | 132.576 | 3406.130 |
| 126.265 | 3120.257 | 135.276 | 3048.540 | 132.465 | 3396.505 |
| 126.137 | 3104.528 | 135.170 | 3036.589 | 132.354 | 3386.879 |
| 126.008 | 3088.839 | 135.063 | 3024.706 | 132.243 | 3377.254 |
| 125.880 | 3073.484 | 134.957 | 3012.900 | 132.132 | 3367.629 |
| 125.752 | 3058.597 | 134.850 | 3000.491 | 132.021 | 3357.875 |
| 125.623 | 3043.710 | 134.744 | 2988.048 | 131.910 | 3347.479 |
| 125.495 | 3028.922 | 134.638 | 2975.605 | 131.799 | 3336.852 |
| 125.367 | 3014.434 | 134.531 | 2963.166 | 131.688 | 3326.225 |
| 125.238 | 3000.361 | 134.425 | 2950.493 | 131.577 | 3315.598 |
| 125.110 | 2986.289 | 134.318 | 2937.235 | 131.466 | 3304.971 |
| 124.982 | 2972.216 | 134.212 | 2923.901 | 131.355 | 3294.246 |
| 124.853 | 2958.133 | 134.106 | 2911.292 | 131.244 | 3283.451 |
| 124.725 | 2943.557 | 133.999 | 2898.413 | 131.133 | 3273.633 |
| 124.597 | 2928.980 | 133.893 | 2885.638 | 131.022 | 3264.361 |
| 124.468 | 2914.481 | 133.786 | 2872.927 | 130.911 | 3255.089 |
| 124.340 | 2900.161 | 133.680 | 2860.733 | 130.800 | 3246.522 |
| 124.211 | 2886.323 | 133.573 | 2848.802 | 130.689 | 3237.995 |
| 124.083 | 2872.323 | 133.467 | 2836.825 | 130.578 | 3229.468 |
| 123.955 | 2858.323 | 133.361 | 2824.711 | 130.467 | 3220.942 |
| 123.826 | 2844.295 | 133.254 | 2812.736 | 130.356 | 3212.415 |
| 123.698 | 2830.161 | 133.148 | 2800.790 | 130.245 | 3203.888 |
| 123.570 | 2816.027 | 133.041 | 2788.843 | 130.134 | 3195.361 |
| 123.441 | 2801.893 | 132.935 | 2776.895 | 130.023 | 3186.790 |
| 123.313 | 2788.555 | 132.829 | 2764.742 | 129.912 | 3178.082 |
| 123.185 | 2775.603 | 132.722 | 2752.391 | 129.801 | 3169.673 |
| 123.056 | 2762.725 | 132.616 | 2739.949 | 129.690 | 3161.265 |
| 122.928 | 2749.354 | 132.509 | 2728.097 | 129.579 | 3150.806 |
| 122.799 | 2736.170 | 132.403 | 2716.539 | 129.468 | 3136.219 |
| 122.671 | 2723.154 | 132.297 | 2705.225 | 129.357 | 3121.633 |
| 122.543 | 2710.137 | 132.190 | 2693.974 | 129.246 | 3107.047 |
| 122.414 | 2697.121 | 132.084 | 2682.770 | 129.135 | 3095.308 |
| 122.286 | 2684.104 | 131.977 | 2671.565 | 129.023 | 3085.888 |
| 122.158 | 2671.206 | 131.871 | 2660.169 | 128.912 | 3076.468 |
| 122.029 | 2659.388 | 131.764 | 2648.716 | 128.801 | 3067.094 |
| 121.901 | 2647.099 | 131.658 | 2637.068 | 128.690 | 3057.749 |
| 121.773 | 2634.720 | 131.552 | 2624.918 | 128.579 | 3048.405 |
| 121.644 | 2621.552 | 131.445 | 2612.407 | 128.468 | 3038.265 |
| 121.516 | 2608.349 | 131.339 | 2599.917 | 128.357 | 3027.770 |
| 121.388 | 2595.145 | 131.232 | 2587.169 | 128.246 | 3017.274 |
| 121.259 | 2581.942 | 131.126 | 2574.036 | 128.135 | 3007.128 |
| 121.131 | 2568.739 | 131.020 | 2561.216 | 128.024 | 2997.910 |
| 121.002 | 2555.535 | 130.913 | 2548.258 | 127.913 | 2988.693 |
| 120.874 | 2542.240 | 130.807 | 2535.954 | 127.802 | 2979.475 |
| 120.746 | 2529.056 | 130.700 | 2524.169 | 127.691 | 2970.258 |
| 120.617 | 2515.818 | 130.594 | 2512.385 | 127.580 | 2960.810 |
| 120.489 | 2502.474 | 130.488 | 2500.849 | 127.469 | 2951.867 |
| 120.361 | 2487.807 | 130.381 | 2489.642 | 127.358 | 2942.923 |
| 120.232 | 2473.141 | 130.275 | 2478.118 | 127.247 | 2933.980 |
| 120.104 | 2458.475 | 130.168 | 2466.123 | 127.136 | 2925.036 |
| 119.976 | 2444.571 | 130.062 | 2453.733 | 127.025 | 2916.093 |
| 119.847 | 2431.283 | 129.955 | 2441.334 | 126.914 | 2907.149 |
| 119.719 | 2419.365 | 129.849 | 2429.186 | 126.803 | 2898.206 |
| 119.590 | 2408.034 | 129.743 | 2417.294 | 126.692 | 2889.268 |
| 119.462 | 2396.223 | 129.636 | 2405.257 | 126.581 | 2880.374 |
| 119.334 | 2383.965 | 129.530 | 2393.759 | 126.470 | 2871.211 |
| 119.205 | 2371.836 | 129.423 | 2382.128 | 126.359 | 2862.265 |
| 119.077 | 2359.708 | 129.317 | 2371.086 | 126.248 | 2853.380 |
| 118.949 | 2347.579 | 129.211 | 2360.129 | 126.137 | 2844.496 |
| 118.820 | 2335.489 | 129.104 | 2349.036 | 126.026 | 2835.612 |
| 118.692 | 2323.692 | 128.998 | 2337.947 | 125.915 | 2826.727 |
| 118.564 | 2312.124 | 128.891 | 2327.443 | 125.804 | 2817.843 |
| 118.435 | 2300.813 | 128.785 | 2316.938 | 125.693 | 2808.959 |
| 118.307 | 2289.596 | 128.679 | 2306.183 | 125.582 | 2800.455 |
| 118.179 | 2278.366 | 128.572 | 2295.098 | 125.471 | 2792.105 |
| 118.050 | 2267.135 | 128.466 | 2283.957 | 125.360 | 2783.755 |
| 117.922 | 2255.905 | 128.359 | 2272.771 | 125.249 | 2775.405 |
| 117.793 | 2244.505 | 128.253 | 2261.375 | 125.138 | 2764.944 |
| 117.665 | 2233.097 | 128.146 | 2249.635 | 125.027 | 2754.113 |
| 117.537 | 2222.158 | 128.040 | 2237.874 | 124.916 | 2743.282 |
| 117.408 | 2211.673 | 127.934 | 2226.494 | 124.805 | 2732.451 |
| 117.280 | 2201.120 | 127.827 | 2215.227 | 124.694 | 2721.619 |
| 117.152 | 2190.806 | 127.721 | 2204.382 | 124.583 | 2710.788 |
| 117.023 | 2180.500 | 127.614 | 2193.527 | 124.472 | 2700.119 |
| 116.895 | 2170.193 | 127.508 | 2182.221 | 124.361 | 2690.386 |
| 116.767 | 2159.887 | 127.402 | 2171.162 | 124.250 | 2681.119 |
| 116.638 | 2149.580 | 127.295 | 2160.143 | 124.139 | 2672.392 |
| 116.510 | 2139.272 | 127.189 | 2148.875 | 124.028 | 2663.968 |
| 116.381 | 2128.965 | 127.082 | 2136.610 | 123.917 | 2655.545 |
| 116.253 | 2118.743 | 126.976 | 2124.737 | 123.806 | 2647.121 |
| 116.125 | 2108.883 | 126.870 | 2113.627 | 123.695 | 2638.698 |
| 115.996 | 2099.068 | 126.763 | 2102.490 | 123.584 | 2630.274 |
| 115.868 | 2089.275 | 126.657 | 2091.346 | 123.473 | 2621.850 |
| 115.740 | 2078.906 | 126.550 | 2081.021 | 123.362 | 2613.427 |
| 115.611 | 2068.536 | 126.444 | 2071.418 | 123.251 | 2605.366 |
| 115.483 | 2058.200 | 126.337 | 2062.079 | 123.140 | 2597.569 |
| 115.355 | 2048.236 | 126.231 | 2052.652 | 123.029 | 2589.772 |
| 115.226 | 2038.272 | 126.125 | 2043.049 | 122.918 | 2581.975 |
| 115.098 | 2028.308 | 126.018 | 2033.447 | 122.807 | 2574.166 |
| 114.970 | 2018.381 | 125.912 | 2024.012 | 122.696 | 2566.348 |
| 114.841 | 2008.681 | 125.805 | 2014.578 | 122.585 | 2558.531 |
| 114.713 | 1999.296 | 125.699 | 2004.928 | 122.474 | 2550.713 |
| 114.584 | 1990.006 | 125.593 | 1994.649 | 122.363 | 2542.895 |
| 114.456 | 1980.808 | 125.486 | 1983.612 | 122.252 | 2535.078 |
| 114.328 | 1971.609 | 125.380 | 1972.575 | 122.141 | 2527.260 |
| 114.199 | 1962.410 | 125.273 | 1961.032 | 122.030 | 2519.148 |
| 114.071 | 1953.211 | 125.167 | 1949.168 | 121.919 | 2510.972 |
| 113.943 | 1944.093 | 125.061 | 1937.243 | 121.808 | 2502.400 |
| 113.814 | 1934.763 | 124.954 | 1925.523 | 121.697 | 2493.633 |
| 113.686 | 1925.859 | 124.848 | 1913.876 | 121.586 | 2484.451 |
| 113.558 | 1917.109 | 124.741 | 1902.573 | 121.475 | 2473.911 |
| 113.429 | 1908.510 | 124.635 | 1891.735 | 121.364 | 2463.370 |
| 113.301 | 1899.852 | 124.529 | 1881.400 | 121.253 | 2452.830 |
| 113.173 | 1891.229 | 124.422 | 1871.065 | 121.142 | 2442.289 |
| 113.044 | 1882.658 | 124.316 | 1860.892 | 121.031 | 2431.734 |
| 112.916 | 1874.290 | 124.209 | 1851.236 | 120.920 | 2421.170 |
| 112.787 | 1866.227 | 124.103 | 1840.900 | 120.809 | 2410.815 |
| 112.659 | 1858.163 | 123.996 | 1830.482 | 120.698 | 2400.699 |
| 112.531 | 1850.099 | 123.890 | 1820.369 | 120.587 | 2390.566 |
| 112.402 | 1841.954 | 123.784 | 1810.320 | 120.476 | 2380.432 |
| 112.274 | 1833.799 | 123.677 | 1800.356 | 120.365 | 2370.299 |
| 112.146 | 1825.577 | 123.571 | 1790.391 | 120.254 | 2360.165 |
| 112.017 | 1817.488 | 123.464 | 1780.618 | 120.143 | 2350.032 |
| 111.889 | 1809.548 | 123.358 | 1770.819 | 120.032 | 2340.377 |
| 111.761 | 1801.617 | 123.252 | 1761.429 | 119.921 | 2331.771 |
| 111.632 | 1794.021 | 123.145 | 1752.226 | 119.810 | 2323.808 |
| 111.504 | 1786.448 | 123.039 | 1743.023 | 119.699 | 2315.898 |
| 111.375 | 1778.874 | 122.932 | 1733.744 | 119.588 | 2308.276 |
| 111.247 | 1771.301 | 122.826 | 1724.112 | 119.477 | 2300.654 |
| 111.119 | 1763.848 | 122.720 | 1714.684 | 119.366 | 2293.032 |
| 110.990 | 1756.506 | 122.613 | 1705.514 | 119.255 | 2285.410 |
| 110.862 | 1749.149 | 122.507 | 1696.344 | 119.144 | 2277.788 |
| 110.734 | 1741.695 | 122.400 | 1687.080 | 119.033 | 2270.166 |
| 110.605 | 1734.470 | 122.294 | 1677.927 | 118.922 | 2262.544 |
| 110.477 | 1727.381 | 122.187 | 1668.782 | 118.811 | 2254.921 |
| 110.349 | 1720.547 | 122.081 | 1659.662 | 118.700 | 2247.293 |
| 110.220 | 1713.799 | 121.975 | 1650.578 | 118.589 | 2238.232 |
| 110.092 | 1706.921 | 121.868 | 1641.557 | 118.478 | 2228.552 |
| 109.964 | 1700.044 | 121.762 | 1632.594 | 118.367 | 2218.585 |
| 109.835 | 1693.166 | 121.655 | 1623.537 | 118.256 | 2208.617 |
| 109.707 | 1686.289 | 121.549 | 1614.047 | 118.144 | 2198.650 |
| 109.578 | 1679.723 | 121.443 | 1605.099 | 118.033 | 2188.683 |
| 109.450 | 1673.174 | 121.336 | 1596.204 | 117.922 | 2178.715 |
| 109.322 | 1666.625 | 121.230 | 1587.327 | 117.811 | 2168.823 |
| 109.193 | 1660.076 | 121.123 | 1578.457 | 117.700 | 2159.420 |
| 109.065 | 1653.538 | 121.017 | 1569.831 | 117.589 | 2150.841 |
| 108.937 | 1646.958 | 120.911 | 1561.283 | 117.478 | 2142.408 |
| 108.808 | 1639.963 | 120.804 | 1552.669 | 117.367 | 2133.930 |
| 108.680 | 1632.942 | 120.698 | 1544.055 | 117.256 | 2125.453 |
| 108.552 | 1625.749 | 120.591 | 1535.440 | 117.145 | 2116.975 |
| 108.423 | 1618.516 | 120.485 | 1526.826 | 117.034 | 2108.498 |
| 108.295 | 1611.283 | 120.378 | 1518.282 | 116.923 | 2100.020 |
| 108.166 | 1604.123 | 120.272 | 1509.800 | 116.812 | 2091.542 |
| 108.038 | 1597.052 | 120.166 | 1501.459 | 116.701 | 2082.895 |
| 107.910 | 1589.989 | 120.059 | 1492.558 | 116.590 | 2074.159 |
| 107.781 | 1583.303 | 119.953 | 1483.593 | 116.479 | 2065.423 |
| 107.653 | 1576.892 | 119.846 | 1474.654 | 116.368 | 2056.401 |
| 107.525 | 1570.478 | 119.740 | 1465.527 | 116.257 | 2047.372 |
| 107.396 | 1564.064 | 119.634 | 1456.164 | 116.146 | 2038.344 |
| 107.268 | 1557.747 | 119.527 | 1446.800 | 116.035 | 2029.315 |
| 107.140 | 1551.653 | 119.421 | 1437.542 | 115.924 | 2020.287 |
| 107.011 | 1545.559 | 119.314 | 1428.656 | 115.813 | 2011.272 |
| 106.883 | 1539.488 | 119.208 | 1420.287 | 115.702 | 2002.563 |
| 106.755 | 1533.643 | 119.102 | 1411.570 | 115.591 | 1993.853 |
| 106.626 | 1527.798 | 118.995 | 1402.788 | 115.480 | 1985.613 |
| 106.498 | 1521.464 | 118.889 | 1393.825 | 115.369 | 1977.506 |
| 106.369 | 1515.051 | 118.782 | 1384.798 | 115.258 | 1969.399 |
| 106.241 | 1508.819 | 118.676 | 1375.753 | 115.147 | 1961.236 |
| 106.113 | 1502.588 | 118.569 | 1366.707 | 115.036 | 1952.992 |
| 105.984 | 1496.356 | 118.463 | 1357.821 | 114.925 | 1944.749 |
| 105.856 | 1490.124 | 118.357 | 1349.114 | 114.814 | 1936.505 |
| 105.728 | 1483.892 | 118.250 | 1340.831 | 114.703 | 1928.636 |
| 105.599 | 1477.859 | 118.144 | 1332.642 | 114.592 | 1921.234 |
| 105.471 | 1472.037 | 118.037 | 1324.723 | 114.481 | 1913.840 |
| 105.343 | 1466.183 | 117.931 | 1316.780 | 114.370 | 1906.532 |
| 105.214 | 1460.328 | 117.825 | 1308.685 | 114.259 | 1899.223 |
| 105.086 | 1454.393 | 117.718 | 1300.376 | 114.148 | 1891.915 |
| 104.957 | 1448.596 | 117.612 | 1292.031 | 114.037 | 1884.607 |
| 104.829 | 1442.800 | 117.505 | 1283.731 | 113.926 | 1876.471 |
| 104.701 | 1437.003 | 117.399 | 1275.917 | 113.815 | 1868.260 |
| 104.572 | 1431.094 | 117.293 | 1268.248 | 113.704 | 1860.048 |
| 104.444 | 1424.859 | 117.186 | 1260.599 | 113.593 | 1851.837 |
| 104.316 | 1418.623 | 117.080 | 1252.957 | 113.482 | 1843.626 |
| 104.187 | 1412.411 | 116.973 | 1245.231 | 113.371 | 1835.495 |
| 104.059 | 1406.261 | 116.867 | 1237.539 | 113.260 | 1827.400 |
| 103.931 | 1400.180 | 116.761 | 1230.054 | 113.149 | 1818.894 |
| 103.802 | 1394.116 | 116.654 | 1222.700 | 113.038 | 1811.152 |
| 103.674 | 1387.989 | 116.548 | 1215.345 | 112.927 | 1803.409 |
| 103.546 | 1381.835 | 116.441 | 1208.105 | 112.816 | 1795.666 |
| 103.417 | 1375.851 | 116.335 | 1200.941 | 112.705 | 1787.923 |
| 103.289 | 1369.868 | 116.228 | 1193.778 | 112.594 | 1780.180 |
| 103.160 | 1363.884 | 116.122 | 1186.670 | 112.483 | 1772.437 |
| 103.032 | 1357.901 | 116.016 | 1179.460 | 112.372 | 1764.694 |
| 102.904 | 1351.918 | 115.909 | 1171.954 | 112.261 | 1756.899 |
| 102.775 | 1345.993 | 115.803 | 1164.600 | 112.150 | 1749.017 |
| 102.647 | 1340.096 | 115.696 | 1157.083 | 112.039 | 1741.064 |
| 102.519 | 1334.245 | 115.590 | 1149.537 | 111.928 | 1732.796 |
| 102.390 | 1328.454 | 115.484 | 1141.990 | 111.817 | 1724.154 |
| 102.262 | 1322.894 | 115.377 | 1134.330 | 111.706 | 1715.511 |
| 102.134 | 1317.419 | 115.271 | 1125.772 | 111.595 | 1706.868 |
| 102.005 | 1311.944 | 115.164 | 1117.083 | 111.484 | 1698.225 |
| 101.877 | 1306.470 | 115.058 | 1108.243 | 111.373 | 1689.508 |
| 101.748 | 1300.995 | 114.952 | 1099.264 | 111.262 | 1680.373 |
| 101.620 | 1295.520 | 114.845 | 1090.373 | 111.151 | 1671.238 |
| 101.492 | 1289.959 | 114.739 | 1081.084 | 111.040 | 1661.917 |
| 101.363 | 1284.399 | 114.632 | 1072.007 | 110.929 | 1652.228 |
| 101.235 | 1278.956 | 114.526 | 1063.696 | 110.818 | 1642.538 |
| 101.107 | 1273.611 | 114.419 | 1055.649 | 110.707 | 1632.848 |
| 100.978 | 1268.266 | 114.313 | 1047.619 | 110.596 | 1623.158 |
| 100.850 | 1262.843 | 114.207 | 1039.638 | 110.485 | 1614.752 |
| 100.722 | 1257.362 | 114.100 | 1032.560 | 110.374 | 1606.424 |
| 100.593 | 1251.883 | 113.994 | 1025.992 | 110.263 | 1598.095 |
| 100.465 | 1246.855 | 113.887 | 1019.425 | 110.152 | 1591.293 |
| 100.337 | 1241.827 | 113.781 | 1012.902 | 110.041 | 1584.458 |
| 100.208 | 1236.798 | 113.675 | 1006.273 | 109.930 | 1577.464 |
| 100.080 | 1231.729 | 113.568 | 999.678 | 109.819 | 1570.470 |
| 99.951 | 1226.623 | 113.462 | 993.410 | 109.708 | 1563.476 |
| 99.823 | 1221.470 | 113.355 | 987.231 | 109.597 | 1556.482 |
| 99.695 | 1216.090 | 113.249 | 981.051 | 109.486 | 1549.488 |
| 99.566 | 1210.689 | 113.143 | 974.865 | 109.375 | 1542.494 |
| 99.438 | 1205.275 | 113.036 | 968.674 | 109.264 | 1535.437 |
| 99.310 | 1199.860 | 112.930 | 962.473 | 109.153 | 1528.223 |
| 99.181 | 1194.446 | 112.823 | 955.817 | 109.042 | 1521.809 |
| 99.053 | 1189.072 | 112.717 | 949.169 | 108.931 | 1515.395 |
| 98.925 | 1183.707 | 112.610 | 942.521 | 108.820 | 1508.982 |
| 98.796 | 1178.384 | 112.504 | 935.870 | 108.709 | 1501.895 |
| 98.668 | 1173.056 | 112.398 | 929.075 | 108.598 | 1494.578 |
| 98.539 | 1167.525 | 112.291 | 922.252 | 108.487 | 1487.262 |
| 98.411 | 1161.725 | 112.185 | 915.653 | 108.376 | 1479.945 |
| 98.283 | 1156.060 | 112.078 | 909.580 | 108.265 | 1472.628 |
| 98.154 | 1150.385 | 111.972 | 903.558 | 108.154 | 1465.402 |
| 98.026 | 1144.709 | 111.866 | 897.446 | 108.043 | 1458.407 |
| 97.898 | 1139.034 | 111.759 | 891.408 | 107.932 | 1451.413 |
| 97.769 | 1133.453 | 111.653 | 885.434 | 107.821 | 1443.031 |
| 97.641 | 1127.796 | 111.546 | 879.495 | 107.710 | 1432.570 |
| 97.513 | 1122.249 | 111.440 | 873.110 | 107.599 | 1422.109 |
| 97.384 | 1116.701 | 111.334 | 866.680 | 107.488 | 1411.649 |
| 97.256 | 1111.153 | 111.227 | 860.170 | 107.377 | 1403.356 |
| 97.128 | 1105.721 | 111.121 | 853.557 | 107.265 | 1397.107 |
| 96.999 | 1100.420 | 111.014 | 847.077 | 107.154 | 1390.858 |
| 96.871 | 1095.385 | 110.908 | 840.673 | 107.043 | 1384.609 |
| 96.742 | 1090.061 | 110.801 | 834.650 | 106.932 | 1378.164 |
| 96.614 | 1084.795 | 110.695 | 828.743 | 106.821 | 1370.737 |
| 96.486 | 1079.746 | 110.589 | 822.942 | 106.710 | 1362.589 |
| 96.357 | 1074.746 | 110.482 | 817.094 | 106.599 | 1354.374 |
| 96.229 | 1069.738 | 110.376 | 810.772 | 106.488 | 1346.158 |
| 96.101 | 1064.730 | 110.269 | 804.385 | 106.377 | 1337.942 |
| 95.972 | 1059.721 | 110.163 | 797.943 | 106.266 | 1329.727 |
| 95.844 | 1054.870 | 110.057 | 791.763 | 106.155 | 1321.632 |
| 95.716 | 1050.209 | 109.950 | 785.238 | 106.044 | 1313.882 |
| 95.587 | 1045.552 | 109.844 | 778.787 | 105.933 | 1306.328 |
| 95.459 | 1040.880 | 109.737 | 772.671 | 105.822 | 1299.134 |
| 95.330 | 1036.209 | 109.631 | 766.578 | 105.711 | 1291.940 |
| 95.202 | 1031.464 | 109.525 | 760.708 | 105.600 | 1284.745 |
| 95.074 | 1026.643 | 109.418 | 755.229 | 105.489 | 1277.551 |
| 94.945 | 1021.770 | 109.312 | 749.750 | 105.378 | 1270.357 |
| 94.817 | 1017.015 | 109.205 | 744.271 | 105.267 | 1263.198 |
| 94.689 | 1012.354 | 109.099 | 738.793 | 105.156 | 1256.898 |
| 94.560 | 1007.693 | 108.992 | 733.398 | 105.045 | 1250.598 |
| 94.432 | 1003.033 | 108.886 | 727.968 | 104.934 | 1244.291 |
| 94.304 | 997.982 | 108.780 | 722.507 | 104.823 | 1237.393 |
| 94.175 | 992.723 | 108.673 | 716.941 | 104.712 | 1230.242 |
| 94.047 | 987.554 | 108.567 | 711.284 | 104.601 | 1223.090 |
| 93.919 | 982.430 | 108.460 | 705.544 | 104.490 | 1215.939 |
| 93.790 | 977.306 | 108.354 | 699.544 | 104.379 | 1208.788 |
| 93.662 | 972.120 | 108.248 | 693.487 | 104.268 | 1201.637 |
| 93.533 | 966.630 | 108.141 | 687.683 | 104.157 | 1194.486 |
| 93.405 | 961.294 | 108.035 | 682.108 | 104.046 | 1187.403 |
| 93.277 | 956.189 | 107.928 | 676.871 | 103.935 | 1181.490 |
| 93.148 | 951.083 | 107.822 | 671.692 | 103.824 | 1175.910 |
| 93.020 | 946.007 | 107.716 | 666.570 | 103.713 | 1170.329 |
| 92.892 | 940.959 | 107.609 | 661.413 | 103.602 | 1164.749 |
| 92.763 | 935.969 | 107.503 | 656.424 | 103.491 | 1159.168 |
| 92.635 | 931.266 | 107.396 | 651.387 | 103.380 | 1153.588 |
| 92.507 | 926.485 | 107.290 | 646.349 | 103.269 | 1148.007 |
| 92.378 | 921.626 | 107.184 | 641.311 | 103.158 | 1142.427 |
| 92.250 | 916.841 | 107.077 | 636.257 | 103.047 | 1136.847 |
| 92.121 | 912.132 | 106.971 | 631.149 | 102.936 | 1131.266 |
| 91.993 | 907.426 | 106.864 | 626.364 | 102.825 | 1125.686 |
| 91.865 | 902.697 | 106.758 | 621.690 | 102.714 | 1118.702 |
| 91.736 | 897.767 | 106.651 | 616.980 | 102.603 | 1110.829 |
| 91.608 | 893.110 | 106.545 | 612.230 | 102.492 | 1102.957 |
| 91.480 | 888.194 | 106.439 | 607.195 | 102.381 | 1095.084 |
| 91.351 | 883.236 | 106.332 | 602.122 | 102.270 | 1087.212 |
| 91.223 | 878.278 | 106.226 | 597.097 | 102.159 | 1080.356 |
| 91.095 | 873.320 | 106.119 | 592.102 | 102.048 | 1074.329 |
| 90.966 | 868.214 | 106.013 | 587.108 | 101.937 | 1068.301 |
| 90.838 | 863.153 | 105.907 | 582.158 | 101.826 | 1062.274 |
| 90.710 | 858.208 | 105.800 | 577.248 | 101.715 | 1056.282 |
| 90.581 | 853.374 | 105.694 | 572.352 | 101.604 | 1050.551 |
| 90.453 | 848.528 | 105.587 | 567.455 | 101.493 | 1044.821 |
| 90.324 | 843.703 | 105.481 | 562.558 | 101.382 | 1038.460 |
| 90.196 | 838.934 | 105.375 | 553.951 | 101.271 | 1031.809 |
| 90.068 | 834.154 | 105.268 | 549.108 | 101.160 | 1025.157 |
| 89.939 | 829.374 | 105.162 | 543.498 | 101.049 | 1018.132 |
| 89.811 | 824.594 | 105.055 | 537.922 | 100.938 | 1011.035 |
| 89.683 | 819.954 | 104.949 | 532.404 | 100.827 | 1003.939 |
| 89.554 | 815.545 | 104.842 | 527.082 | 100.716 | 996.843 |
| 89.426 | 811.194 | 104.736 | 522.074 | 100.605 | 990.009 |
| 89.298 | 807.216 | 104.630 | 517.502 | 100.494 | 983.445 |
| 89.169 | 803.166 | 104.523 | 512.736 | 100.383 | 976.881 |
| 89.041 | 799.092 | 104.417 | 507.970 | 100.272 | 970.392 |
| 88.912 | 794.998 | 104.310 | 503.204 | 100.161 | 964.439 |
| 88.784 | 790.903 | 104.204 | 498.448 | 100.050 | 958.485 |
| 88.656 | 786.809 | 104.098 | 493.818 | 99.939 | 952.532 |
| 88.527 | 782.714 | 103.991 | 489.287 | 99.828 | 946.578 |
| 88.399 | 778.480 | 103.885 | 485.053 | 99.717 | 940.625 |
| 88.271 | 774.190 | 103.778 | 480.820 | 99.606 | 934.339 |
| 88.142 | 769.484 | 103.672 | 476.610 | 99.495 | 927.182 |
| 88.014 | 764.778 | 103.566 | 472.614 | 99.384 | 919.546 |
| 87.886 | 759.893 | 103.459 | 468.619 | 99.273 | 911.910 |
| 87.757 | 754.898 | 103.353 | 464.636 | 99.162 | 904.248 |
| 87.629 | 749.904 | 103.246 | 460.758 | 99.051 | 896.457 |
| 87.501 | 745.149 | 103.140 | 456.917 | 98.940 | 888.666 |
| 87.372 | 740.612 | 103.033 | 453.025 | 98.829 | 867.279 |
| 87.244 | 736.223 | 102.927 | 449.162 | 98.718 | 860.892 |
| 87.115 | 731.753 | 102.821 | 445.317 | 98.607 | 855.269 |
| 86.987 | 727.230 | 102.714 | 441.472 | 98.496 | 849.647 |
| 86.859 | 722.697 | 102.608 | 437.621 | 98.385 | 844.024 |
| 86.730 | 717.972 | 102.501 | 433.503 | 98.274 | 838.402 |
| 86.602 | 713.266 | 102.395 | 429.402 | 98.163 | 832.867 |
| 86.474 | 708.735 | 102.289 | 425.433 | 98.052 | 827.543 |
| 86.345 | 704.127 | 102.182 | 421.307 | 97.941 | 822.219 |
| 86.217 | 699.368 | 102.076 | 417.111 | 97.830 | 816.895 |
| 86.089 | 694.805 | 101.969 | 412.937 | 97.719 | 811.691 |
| 85.960 | 690.510 | 101.863 | 408.894 | 97.608 | 806.488 |
| 85.832 | 685.923 | 101.757 | 404.214 | 97.497 | 801.284 |
| 85.703 | 681.336 | 101.650 | 399.534 | 97.386 | 796.080 |
| 85.575 | 676.749 | 101.544 | 394.855 | 97.275 | 790.877 |
| 85.447 | 672.323 | 101.437 | 391.058 | 97.164 | 785.673 |
| 85.318 | 667.786 | 101.331 | 387.434 | 97.053 | 780.197 |
| 85.190 | 663.422 | 101.224 | 383.684 | 96.942 | 774.175 |
| 85.062 | 659.202 | 101.118 | 379.935 | 96.831 | 768.153 |
| 84.933 | 654.999 | 101.012 | 376.185 | 96.720 | 762.278 |
| 84.805 | 650.386 | 100.905 | 372.450 | 96.609 | 756.424 |
| 84.677 | 645.327 | 100.799 | 368.701 | 96.498 | 750.570 |
| 84.548 | 640.316 | 100.692 | 365.427 | 96.386 | 744.430 |
| 84.420 | 635.551 | 100.586 | 362.207 | 96.275 | 738.319 |
| 84.292 | 630.787 | 100.480 | 359.005 | 96.164 | 732.318 |
| 84.163 | 625.663 | 100.373 | 355.800 | 96.053 | 726.317 |
| 84.035 | 620.634 | 100.267 | 352.370 | 95.942 | 720.316 |
| 83.906 | 615.846 | 100.160 | 348.930 | 95.831 | 714.329 |
| 83.778 | 612.047 | 100.054 | 345.539 | 95.720 | 708.494 |
| 83.650 | 608.248 | 99.948 | 342.300 | 95.609 | 702.660 |
| 83.521 | 604.448 | 99.841 | 339.072 | 95.498 | 696.966 |
| 83.393 | 600.649 | 99.735 | 335.865 | 95.387 | 692.012 |
| 83.265 | 596.827 | 99.628 | 332.665 | 95.276 | 687.059 |
| 83.136 | 593.000 | 99.522 | 329.320 | 95.165 | 682.105 |
| 83.008 | 589.189 | 99.415 | 325.838 | 95.054 | 677.152 |
| 82.880 | 585.427 | 99.309 | 322.157 | 94.943 | 672.198 |
| 82.751 | 581.422 | 99.203 | 318.476 | 94.832 | 667.245 |
| 82.623 | 577.352 | 99.096 | 314.851 | 94.721 | 661.874 |
| 82.494 | 573.239 | 98.990 | 311.391 | 94.610 | 656.425 |
| 82.366 | 569.125 | 98.883 | 307.931 | 94.499 | 651.023 |
| 82.238 | 564.988 | 98.777 | 304.768 | 94.388 | 645.635 |
| 82.109 | 560.866 | 98.671 | 301.696 | 94.277 | 639.902 |
| 81.981 | 556.741 | 98.564 | 298.596 | 94.166 | 633.854 |
| 81.853 | 552.548 | 98.458 | 295.521 | 94.055 | 627.806 |
| 81.724 | 548.306 | 98.351 | 292.533 | 93.944 | 621.936 |
| 81.596 | 544.031 | 98.245 | 289.545 | 93.833 | 616.085 |
| 81.468 | 539.601 | 98.139 | 286.557 | 93.722 | 610.235 |
| 81.339 | 535.128 | 98.032 | 283.569 | 93.611 | 604.191 |
| 81.211 | 530.628 | 97.926 | 280.587 | 93.500 | 597.417 |
| 81.083 | 526.338 | 97.819 | 277.690 | 93.389 | 590.676 |
| 80.954 | 522.093 | 97.713 | 274.981 | 93.278 | 583.935 |
| 80.826 | 517.992 | 97.607 | 272.301 | 93.167 | 577.370 |
| 80.697 | 513.661 | 97.500 | 269.710 | 93.056 | 570.985 |
| 80.569 | 509.450 | 97.394 | 267.116 | 92.945 | 565.901 |
| 80.441 | 505.430 | 97.287 | 264.514 | 92.834 | 560.873 |
| 80.312 | 501.411 | 97.181 | 261.884 | 92.723 | 556.014 |
| 80.184 | 497.373 | 97.074 | 259.139 | 92.612 | 551.366 |
| 80.056 | 493.431 | 96.968 | 256.394 | 92.501 | 546.718 |
| 79.927 | 489.603 | 96.862 | 253.784 | 92.390 | 542.070 |
| 79.799 | 485.763 | 96.755 | 251.173 | 92.279 | 537.423 |
| 79.671 | 481.924 | 96.649 | 248.553 | 92.168 | 532.648 |
| 79.542 | 478.084 | 96.542 | 245.860 | 92.057 | 527.867 |
| 79.414 | 474.244 | 96.436 | 243.180 | 91.946 | 523.086 |
| 79.285 | 470.389 | 96.330 | 240.429 | 91.835 | 517.981 |
| 79.157 | 466.519 | 96.223 | 237.561 | 91.724 | 512.081 |
| 79.029 | 462.601 | 96.117 | 234.347 | 91.613 | 506.182 |
| 78.900 | 458.628 | 96.010 | 231.001 | 91.502 | 500.282 |
| 78.772 | 454.548 | 95.904 | 227.735 | 91.391 | 494.312 |
| 78.644 | 450.425 | 95.798 | 224.462 | 91.280 | 488.213 |
| 78.515 | 446.303 | 95.691 | 221.292 | 91.169 | 482.594 |
| 78.387 | 442.142 | 95.585 | 218.355 | 91.058 | 477.340 |
| 78.259 | 437.799 | 95.478 | 215.710 | 90.947 | 471.729 |
| 78.130 | 433.441 | 95.372 | 213.296 | 90.836 | 466.118 |
| 78.002 | 428.937 | 95.265 | 210.920 | 90.725 | 460.508 |
| 77.874 | 424.299 | 95.159 | 208.544 | 90.614 | 454.955 |
| 77.745 | 419.661 | 95.053 | 206.175 | 90.503 | 449.421 |
| 77.617 | 415.650 | 94.946 | 203.834 | 90.392 | 444.079 |
| 77.488 | 411.752 | 94.840 | 201.525 | 90.281 | 438.971 |
| 77.360 | 407.854 | 94.733 | 199.240 | 90.170 | 434.034 |
| 77.232 | 404.136 | 94.627 | 197.032 | 90.059 | 429.107 |
| 77.103 | 400.496 | 94.521 | 194.904 | 89.948 | 424.301 |
| 76.975 | 396.935 | 94.414 | 192.787 | 89.837 | 420.136 |
| 76.847 | 393.373 | 94.308 | 190.667 | 89.726 | 415.971 |
| 76.718 | 389.856 | 94.201 | 188.554 | 89.615 | 412.151 |
| 76.590 | 386.341 | 94.095 | 186.442 | 89.504 | 408.579 |
| 76.462 | 382.738 | 93.989 | 184.262 | 89.393 | 405.008 |
| 76.333 | 379.090 | 93.882 | 181.911 | 89.282 | 401.436 |
| 76.205 | 375.250 | 93.776 | 179.555 | 89.171 | 397.765 |
| 76.076 | 371.175 | 93.669 | 177.199 | 89.060 | 394.051 |
| 75.948 | 367.086 | 93.563 | 174.743 | 88.949 | 390.337 |
| 75.820 | 363.057 | 93.456 | 172.282 | 88.838 | 386.623 |
| 75.691 | 359.068 | 93.350 | 169.796 | 88.727 | 381.271 |
| 75.563 | 355.250 | 93.244 | 167.222 | 88.616 | 375.878 |
| 75.435 | 351.890 | 93.137 | 164.694 | 88.505 | 370.278 |
| 75.306 | 348.248 | 93.031 | 162.193 | 88.394 | 364.474 |
| 75.178 | 344.381 | 92.924 | 159.740 | 88.283 | 359.217 |
| 75.050 | 340.515 | 92.818 | 157.370 | 88.172 | 353.966 |
| 74.921 | 336.649 | 92.712 | 155.146 | 88.061 | 348.715 |
| 74.793 | 332.811 | 92.605 | 152.943 | 87.950 | 343.465 |
| 74.665 | 329.635 | 92.499 | 150.400 | 87.839 | 338.214 |
| 74.536 | 326.505 | 92.392 | 147.837 | 87.728 | 333.237 |
| 74.408 | 323.403 | 92.286 | 145.254 | 87.617 | 329.159 |
| 74.279 | 320.301 | 92.180 | 143.101 | 87.506 | 325.342 |
| 74.151 | 317.198 | 92.073 | 141.107 | 87.395 | 321.524 |
| 74.023 | 314.096 | 91.967 | 139.113 | 87.284 | 317.707 |
| 73.894 | 310.994 | 91.860 | 137.191 | 87.173 | 313.889 |
| 73.766 | 307.509 | 91.754 | 135.310 | 87.062 | 310.072 |
| 73.638 | 303.923 | 91.647 | 133.405 | 86.951 | 306.701 |
| 73.509 | 300.298 | 91.541 | 131.489 | 86.840 | 303.569 |
| 73.381 | 296.442 | 91.435 | 129.573 | 86.729 | 300.579 |
| 73.253 | 292.930 | 91.328 | 127.674 | 86.618 | 297.555 |
| 73.124 | 289.422 | 91.222 | 125.828 | 86.507 | 294.200 |
| 72.996 | 285.914 | 91.115 | 123.965 | 86.396 | 290.846 |
| 72.867 | 282.401 | 91.009 | 122.112 | 86.285 | 287.492 |
| 72.739 | 278.649 | 90.903 | 120.116 | 86.174 | 284.138 |
| 72.611 | 274.479 | 90.796 | 117.974 | 86.063 | 280.783 |
| 72.482 | 270.370 | 90.690 | 115.812 | 85.952 | 277.429 |
| 72.354 | 266.406 | 90.583 | 113.651 | 85.841 | 273.431 |
| 72.226 | 262.655 | 90.477 | 111.514 | 85.730 | 268.387 |
| 72.097 | 258.933 | 90.371 | 109.550 | 85.619 | 263.303 |
| 71.969 | 255.610 | 90.264 | 107.558 | 85.507 | 258.220 |
| 71.841 | 252.574 | 90.158 | 105.649 | 85.396 | 253.313 |
| 71.712 | 249.538 | 90.051 | 103.704 | 85.285 | 249.324 |
| 71.584 | 246.568 | 89.945 | 101.791 | 85.174 | 246.065 |
| 71.456 | 243.638 | 89.839 | 99.903 | 85.063 | 242.807 |
| 71.327 | 240.781 | 89.732 | 98.015 | 84.952 | 238.698 |
| 71.199 | 237.964 | 89.626 | 96.207 | 84.841 | 234.306 |
| 71.070 | 235.229 | 89.519 | 94.516 | 84.730 | 229.913 |
| 70.942 | 232.514 | 89.413 | 92.871 | 84.619 | 226.052 |
| 70.814 | 229.576 | 89.306 | 91.251 | 84.508 | 223.170 |
| 70.685 | 226.637 | 89.200 | 89.611 | 84.397 | 220.431 |
| 70.557 | 223.596 | 89.094 | 87.973 | 84.286 | 217.672 |
| 70.429 | 220.569 | 88.987 | 86.419 | 84.175 | 214.749 |
| 70.300 | 217.582 | 88.881 | 85.030 | 84.064 | 211.826 |
| 70.172 | 214.256 | 88.774 | 83.643 | 83.953 | 208.902 |
| 70.044 | 210.410 | 88.668 | 82.256 | 83.842 | 205.979 |
| 69.915 | 206.524 | 88.562 | 80.794 | 83.731 | 203.074 |
| 69.787 | 202.017 | 88.455 | 79.337 | 83.620 | 200.218 |
| 69.659 | 197.468 | 88.349 | 77.898 | 83.509 | 197.176 |
| 69.530 | 194.432 | 88.242 | 76.555 | 83.398 | 192.832 |
| 69.402 | 191.548 | 88.136 | 75.269 | 83.287 | 188.487 |
| 69.273 | 188.455 | 88.030 | 74.003 | 83.176 | 184.142 |
| 69.145 | 185.425 | 87.923 | 72.824 | 83.065 | 179.797 |
| 69.017 | 182.642 | 87.817 | 71.667 | 82.954 | 176.606 |
| 68.888 | 179.858 | 87.710 | 70.525 | 82.843 | 173.740 |
| 68.760 | 177.267 | 87.604 | 69.398 | 82.732 | 169.951 |
| 68.632 | 174.694 | 87.497 | 68.161 | 82.621 | 166.162 |
| 68.503 | 172.052 | 87.391 | 66.683 | 82.510 | 162.373 |
| 68.375 | 169.305 | 87.285 | 65.197 | 82.399 | 159.501 |
| 68.247 | 166.544 | 87.178 | 63.821 | 82.288 | 156.802 |
| 68.118 | 163.704 | 87.072 | 62.395 | 82.177 | 154.371 |
| 67.990 | 160.810 | 86.965 | 60.801 | 82.066 | 151.940 |
| 67.861 | 157.916 | 86.859 | 59.188 | 81.955 | 149.541 |
| 67.733 | 154.845 | 86.753 | 57.604 | 81.844 | 147.179 |
| 67.605 | 151.738 | 86.646 | 56.296 | 81.733 | 145.041 |
| 67.476 | 148.762 | 86.540 | 54.962 | 81.622 | 143.004 |
| 67.348 | 146.107 | 86.433 | 53.712 | 81.511 | 140.966 |
| 67.220 | 143.737 | 86.327 | 52.487 | 81.400 | 138.928 |
| 67.091 | 141.428 | 86.221 | 51.261 | 81.289 | 136.814 |
| 66.963 | 139.111 | 86.114 | 50.054 | 81.178 | 134.158 |
| 66.835 | 136.857 | 86.008 | 48.773 | 81.067 | 131.503 |
| 66.706 | 134.657 | 85.901 | 47.523 | 80.956 | 128.647 |
| 66.578 | 132.456 | 85.795 | 46.359 | 80.845 | 125.492 |
| 66.450 | 129.942 | 85.688 | 45.234 | 80.734 | 121.903 |
| 66.321 | 127.311 | 85.582 | 44.109 | 80.623 | 118.089 |
| 66.193 | 124.656 | 85.476 | 43.013 | 80.512 | 114.274 |
| 66.064 | 121.932 | 85.369 | 41.934 | 80.401 | 110.557 |
| 65.936 | 119.395 | 85.263 | 40.837 | 80.290 | 107.653 |
| 65.808 | 117.012 | 85.156 | 39.736 | 80.179 | 105.494 |
| 65.679 | 114.741 | 85.050 | 38.620 | 80.068 | 103.334 |
| 65.551 | 112.413 | 84.944 | 37.513 | 79.957 | 101.175 |
| 65.423 | 110.139 | 84.837 | 36.413 | 79.846 | 99.016 |
| 65.294 | 107.864 | 84.731 | 35.406 | 79.735 | 97.257 |
| 65.166 | 105.590 | 84.624 | 34.415 | 79.624 | 95.537 |
| 65.038 | 103.258 | 84.518 | 33.435 | 79.513 | 93.816 |
| 64.909 | 100.987 | 84.412 | 32.466 | 79.402 | 92.169 |
| 64.781 | 98.762 | 84.305 | 31.560 | 79.291 | 90.539 |
| 64.652 | 96.604 | 84.199 | 30.657 | 79.180 | 88.809 |
| 64.524 | 94.446 | 84.092 | 29.710 | 79.069 | 87.077 |
| 64.396 | 92.327 | 83.986 | 28.781 | 78.958 | 85.345 |
| 64.267 | 90.138 | 83.879 | 27.828 | 78.847 | 83.611 |
| 64.139 | 87.959 | 83.773 | 26.955 | 78.736 | 81.841 |
| 64.011 | 85.780 | 83.667 | 26.217 | 78.625 | 80.049 |
| 63.882 | 83.601 | 83.560 | 25.507 | 78.514 | 78.095 |
| 63.754 | 81.630 | 83.454 | 24.793 | 78.403 | 75.784 |
| 63.626 | 79.786 | 83.347 | 24.060 | 78.292 | 73.125 |
| 63.497 | 77.912 | 83.241 | 23.309 | 78.181 | 70.466 |
| 63.369 | 75.952 | 83.135 | 22.557 | 78.070 | 67.852 |
| 63.241 | 74.003 | 83.028 | 21.802 | 77.959 | 65.423 |
| 63.112 | 71.419 | 82.922 | 21.031 | 77.848 | 63.088 |
| 62.984 | 68.552 | 82.815 | 20.288 | 77.737 | 60.752 |
| 62.855 | 66.631 | 82.709 | 19.533 | 77.626 | 58.835 |
| 62.727 | 64.757 | 82.603 | 18.765 | 77.515 | 57.360 |
| 62.599 | 62.896 | 82.496 | 18.018 | 77.404 | 56.086 |
| 62.470 | 61.091 | 82.390 | 17.346 | 77.293 | 54.812 |
| 62.342 | 59.437 | 82.283 | 16.674 | 77.182 | 53.538 |
| 62.214 | 57.787 | 82.177 | 15.902 | 77.071 | 52.263 |
| 62.085 | 56.113 | 82.070 | 15.051 | 76.960 | 50.988 |
| 61.957 | 54.403 | 81.964 | 14.403 | 76.849 | 49.699 |
| 61.829 | 52.684 | 81.858 | 13.801 | 76.738 | 48.399 |
| 61.700 | 50.988 | 81.751 | 13.249 | 76.627 | 47.049 |
| 61.572 | 49.403 | 81.645 | 12.703 | 76.516 | 45.678 |
| 61.443 | 47.845 | 81.538 | 12.158 | 76.405 | 44.307 |
| 61.315 | 46.182 | 81.432 | 11.615 | 76.294 | 42.936 |
| 61.187 | 44.521 | 81.326 | 11.102 | 76.183 | 41.565 |
| 61.058 | 42.873 | 81.219 | 10.617 | 76.072 | 40.192 |
| 60.930 | 41.239 | 81.113 | 10.161 | 75.961 | 38.931 |
| 60.802 | 39.745 | 81.006 | 9.705 | 75.850 | 37.652 |
| 60.673 | 38.206 | 80.900 | 9.283 | 75.739 | 36.374 |
| 60.545 | 36.675 | 80.794 | 8.905 | 75.628 | 35.096 |
| 60.417 | 35.470 | 80.687 | 8.524 | 75.517 | 33.817 |
| 60.288 | 35.557 | 80.581 | 8.193 | 75.406 | 32.647 |
| 60.160 | 35.454 | 80.474 | 7.861 | 75.295 | 31.716 |
| 60.032 | 35.279 | 80.368 | 7.581 | 75.184 | 30.785 |
| 59.903 | 35.273 | 80.262 | 7.272 | 75.073 | 29.855 |
| 59.775 | 35.573 | 80.155 | 6.940 | 74.962 | 28.924 |
| 59.646 | 35.855 | 80.049 | 6.684 | 74.851 | 27.963 |
| 59.518 | 36.137 | 79.942 | 6.441 | 74.740 | 27.030 |
| 59.390 | 36.423 | 79.836 | 6.226 | 74.628 | 26.157 |
| 59.261 | 36.709 | 79.729 | 5.991 | 74.517 | 25.270 |
| 59.133 | 36.900 | 79.623 | 5.757 | 74.406 | 24.122 |
| 59.005 | 37.054 | 79.517 | 5.537 | 74.295 | 22.974 |
| 58.876 | 37.200 | 79.410 | 5.311 | 74.184 | 21.827 |
| 58.748 | 37.524 | 79.304 | 5.092 | 74.073 | 20.708 |
| 58.620 | 37.894 | 79.197 | 4.882 | 73.962 | 19.860 |
| 58.491 | 38.265 | 79.091 | 4.614 | 73.851 | 18.998 |
| 58.363 | 38.508 | 78.985 | 4.360 | 73.740 | 18.137 |
| 58.234 | 38.642 | 78.878 | 4.080 | 73.629 | 17.275 |
| 58.106 | 38.856 | 78.772 | 3.785 | 73.518 | 16.413 |
| 57.978 | 39.238 | 78.665 | 3.480 | 73.407 | 15.635 |
| 57.849 | 39.689 | 78.559 | 3.174 | 73.296 | 15.000 |
| 57.721 | 40.122 | 78.453 | 2.877 | 73.185 | 14.366 |
| 57.593 | 40.527 | 78.346 | 2.576 | 73.074 | 13.731 |
| 57.464 | 41.219 | 78.240 | 2.268 | 72.963 | 13.100 |
| 57.336 | 41.942 | 78.133 | 1.961 | 72.852 | 12.499 |
| 57.208 | 42.656 | 78.027 | 1.653 | 72.741 | 11.898 |
| 57.079 | 43.371 | 77.920 | 1.346 | 72.630 | 11.407 |
| 56.951 | 44.118 | 77.814 | 1.107 | 72.519 | 10.953 |
| 56.823 | 44.919 | 77.708 | 0.880 | 72.408 | 10.458 |
| 56.694 | 45.527 | 77.601 | 0.639 | 72.297 | 9.972 |
| 56.566 | 46.170 | 77.495 | 0.394 | 72.186 | 9.504 |
| 56.437 | 47.126 | 77.388 | 0.148 | 72.075 | 9.135 |
| 56.309 | 48.097 | 77.282 | -0.098 | 71.964 | 8.737 |
| 56.181 | 49.061 | 77.176 | -0.344 | 71.853 | 8.372 |
| 56.052 | 50.080 | 77.069 | -0.589 | 71.742 | 8.028 |
| 55.924 | 51.144 | 76.963 | -0.839 | 71.631 | 7.704 |
| 55.796 | 52.140 | 76.856 | -1.091 | 71.520 | 7.412 |
| 55.667 | 53.136 | 76.750 | -1.336 | 71.409 | 7.120 |
| 55.539 | 54.122 | 76.644 | -1.581 | 71.298 | 6.829 |
| 55.411 | 55.143 | 76.537 | -1.825 | 71.187 | 6.537 |
| 55.282 | 56.451 | 76.431 | -2.097 | 71.076 | 6.246 |
| 55.154 | 57.413 | 76.324 | -2.347 | 70.965 | 5.960 |
| 55.025 | 58.562 | 76.218 | -2.555 | 70.854 | 5.680 |
| 54.897 | 59.697 | 76.111 | -2.768 | 70.743 | 5.434 |
| 54.769 | 60.776 | 76.005 | -2.965 | 70.632 | 5.201 |
| 54.640 | 61.748 | 75.899 | -3.167 | 70.521 | 4.968 |
| 54.512 | 62.725 | 75.792 | -3.365 | 70.410 | 4.736 |
| 54.384 | 63.795 | 75.686 | -3.563 | 70.299 | 4.503 |
| 54.255 | 64.551 | 75.579 | -3.771 | 70.188 | 4.270 |
| 54.127 | 65.043 | 75.473 | -3.955 | 70.077 | 4.037 |
| 53.999 | 66.540 | 75.367 | -4.117 | 69.966 | 3.802 |
| 53.870 | 67.901 | 75.260 | -4.267 | 69.855 | 3.520 |
| 53.742 | 68.754 | 75.154 | -4.433 | 69.744 | 3.240 |
| 53.614 | 69.947 | 75.047 | -4.583 | 69.633 | 2.960 |
| 53.485 | 71.235 | 74.941 | -4.746 | 69.522 | 2.680 |
| 53.357 | 72.557 | 74.835 | -4.926 | 69.411 | 2.400 |
| 53.228 | 73.890 | 74.728 | -5.103 | 69.300 | 2.210 |
| 53.100 | 75.111 | 74.622 | -5.278 | 69.189 | 2.094 |
| 52.972 | 76.348 | 74.515 | -5.428 | 69.078 | 1.934 |
| 52.843 | 77.579 | 74.409 | -5.585 | 68.967 | 1.783 |
| 52.715 | 78.819 | 74.302 | -5.707 | 68.856 | 1.669 |
| 52.587 | 80.060 | 74.196 | -5.249 | 68.745 | 1.545 |
| 52.458 | 81.405 | 74.090 | -4.175 | 68.634 | 1.431 |
| 52.330 | 82.750 | 73.983 | -3.101 | 68.523 | 1.272 |
| 52.202 | 84.107 | 73.877 | -2.027 | 68.412 | 1.155 |
| 52.073 | 85.470 | 73.770 | -0.954 | 68.301 | 1.047 |
| 51.945 | 86.834 |  |  | 68.190 | 0.978 |
| 51.816 | 88.187 |  |  | 68.079 | 0.916 |
| 51.688 | 89.547 |  |  | 67.968 | 0.777 |
| 51.560 | 90.906 |  |  | 67.857 | 0.637 |
| 51.431 | 92.262 |  |  | 67.746 | 0.497 |
| 51.303 | 93.623 |  |  | 67.635 | 0.357 |
| 51.175 | 94.993 |  |  | 67.524 | 0.217 |
| 51.046 | 96.346 |  |  | 67.413 | 0.077 |
| 50.918 | 97.706 |  |  | 67.302 | -0.066 |
|  |  |  |  | 67.191 | -0.234 |
|  |  |  |  | 67.080 | -0.402 |
|  |  |  |  | 66.969 | -0.570 |
|  |  |  |  | 66.858 | -0.738 |
|  |  |  |  | 66.747 | -0.878 |
|  |  |  |  | 66.636 | -0.968 |
|  |  |  |  | 66.525 | -1.058 |
|  |  |  |  | 66.414 | -1.147 |
|  |  |  |  | 66.303 | -1.237 |
|  |  |  |  | 66.192 | -1.325 |
|  |  |  |  | 66.081 | -1.413 |
|  |  |  |  | 65.970 | -1.501 |
|  |  |  |  | 65.859 | -1.549 |
|  |  |  |  | 65.748 | -1.618 |
|  |  |  |  | 65.637 | -1.691 |
|  |  |  |  | 65.526 | -1.765 |
|  |  |  |  | 65.415 | -1.816 |
|  |  |  |  | 65.304 | -1.886 |
|  |  |  |  | 65.193 | -1.933 |
|  |  |  |  | 65.082 | -1.996 |
|  |  |  |  | 64.971 | -2.029 |
|  |  |  |  | 64.860 | -2.110 |
|  |  |  |  | 64.749 | -2.175 |
|  |  |  |  | 64.638 | -2.223 |
|  |  |  |  | 64.527 | -2.293 |
|  |  |  |  | 64.416 | -2.355 |
|  |  |  |  | 64.305 | -2.404 |

Table 7. Raw data of Fig. 6.

| silicon (100 h) | | 36 mol% HfO_2_-doped silicon (100 h) | | 60 mol% HfO_2_-doped silicon (100 h) | |
| --- | --- | --- | --- | --- | --- |
| E_r_ (GPa) | H (GPa) | E_r_ (GPa) | H (GPa) | E_r_ (GPa) | H (GPa) |
| 217.532304 | 10.861417 | 159.69963 | 7.81764 | 101.130316 | 8.801451 |
| 232.018225 | 11.037162 | 166.147189 | 8.447629 | 110.793267 | 9.17202 |
| 193.217461 | 10.320329 | 162.151428 | 8.57956 | 126.424621 | 8.039774 |
| 203.301176 | 9.977692 | 147.121144 | 7.219124 | 115.863371 | 7.792622 |
| 196.452247 | 9.729563 | 161.525282 | 7.917845 | 132.01941 | 8.270634 |
| 188.498935 | 9.996762 | 144.751915 | 8.112183 | 141.442231 | 8.334383 |
